# Developing a Best Practice Guideline for Clinical Practice in a Digital Health Environment: Systematic Reviews Based on the Grading of Recommendations, Assessment, Development, and Evaluation Approach

**DOI:** 10.2196/74942

**Published:** 2026-01-23

**Authors:** Lauren Bailey, Lyndsay Howitt, Nafsin Nizum, Christine Buchanan, Maureen Charlebois, Jennifer Yoon, RNAO Expert Panel, Rob Beleno

**Affiliations:** 1 International Affairs and Best Practice Guidelines Centre, Registered Nurses' Association of Ontario, 4211 Yonge Street #500, Toronto, ON, M4G 2C5, Canada, 1 416-599-1925; 2 Bayshore HealthCare, Toronto, ON, Canada; 3 Humber River Health, Toronto, ON, Canada; 4 See Acknowledgement

**Keywords:** digital health, nursing, electronic health, health informatics, clinical guidelines

## Abstract

**Background:**

Digital health refers to the field of knowledge and practice associated with the development and use of digital technologies to improve clinical practice and health outcomes. Knowledge of digital health technology is becoming essential for all nurses and health providers.

**Objective:**

This study aims to present the results of the systematic reviews that were used to inform the recommendations in a best practice guideline (BPG) following the GRADE (Grading of Recommendations, Assessment, Development, and Evaluation) approach. Reviews focused on digital health education for nurses and health providers, peer champion models, and the use of predictive analytics in digital health environments.

**Methods:**

The BPG team, in collaboration with a panel of 17 experts, conducted 5 systematic reviews to address 5 recommendation questions. Systematic searches looked for relevant studies published in English from January 2017 to July 2022 from 10 databases. The GRADE approach was used to synthesize and evaluate the quality of evidence, ensuring the guideline aligned with international reporting standards.

**Results:**

A total of 18 articles across 4 systematic reviews met the inclusion criteria. From these reviews, 4 corresponding recommendations were drafted for nurses and health providers. The strength of the recommendations was determined through discussion and consensus by the expert panel using the GRADE approach. Among all, 1 systematic review resulted in no recommendation due to insufficient evidence.

**Conclusions:**

The BPG on digital health provides 4 evidence-based recommendations for nurses and health providers on how to incorporate digital health technologies into clinical practice. This BPG is intended to be used across all health care settings.

## Introduction

Over the last decade, there has been an increased uptake of digital health technologies across global health care systems [1]. Digital health is a broad term that refers to the field of knowledge and practice associated with the development and use of digital technologies to improve health [2]. Digital health technologies refer to tools, systems, or devices that can generate, create, store, or process data, enabled through microprocesses that are programmed to perform specific functions [3]. Specifically in health care settings (or digital health environments), digital health technologies may encompass eHealth, mHealth, health informatics, artificial intelligence (AI), machine learning, big data, robotics, and advanced computing sciences [2]. A digital health environment refers to any setting where health providers, informatics professionals, administrators, managers, and persons or families receiving care work in supportive teams to leverage digital tools, technologies, and services to optimize care delivery and empower and activate people to manage their health and wellness [4]. Nurses and health providers use a variety of digital health technologies in practice, including electronic health records, clinical decision support systems (CDSSs) that use predictive analytics, robotics, mobile apps, virtual care platforms, wearable devices, remote monitoring systems, smart home technologies, and others [4]. As nursing practice continues to evolve across all settings and sectors to incorporate these technologies, ongoing education is essential for nurses and health providers to deliver comprehensive clinical care [4,5].

Digital health technologies are advancing at a rapid pace; however, challenges remain in supporting nurses and health providers in using these technologies safely and effectively [6]. Educators and health systems leaders must work to evolve the understanding of novel nurse-patient interactions involving digital health technologies, alongside other core nursing topics [5]. Through further education and training, nurses will have a greater understanding of how both new and existing digital health technologies may impact clinical processes and communication patterns between patients, caregivers, and the interprofessional team [7]. Furthermore, nurses in clinical practice will require initial and ongoing professional development opportunities to aid in the use of digital health technologies [8,9]. Effective training will enable nurses to use these technologies both safely and effectively. Many of the good practice statements, recommendations, and resources within this best practice guideline (BPG) provide guidance on education for nurses and health providers to address this growing need.

The Registered Nurses’ Association of Ontario (RNAO) published a new BPG entitled *Clinical Practice in a Digital Health Environment* in March 2024 [4]. The BPG was developed with an expert panel, which included 17 digital health experts representing diverse backgrounds including nursing, education, research, allied health, and people with lived experience. The purpose of the BPG is to provide evidence-based recommendations that foster nurses’ ability to maintain, advance, and strengthen professional practice in the context of a digital health environment [4]. The guideline is intended for all nurses (registered nurses, nurse practitioners, and registered practical nurses), nursing students, as well as members of the interprofessional health care team, educators, administrators, executives, policymakers, researchers, and people with lived experience. Within the context of this BPG, people with lived experience refer to patients and family within health systems wherein digital health is used.

The aim of this paper is to describe the BPG development process and the results from 4 systematic reviews that were used to inform the recommendations in the BPG, following the GRADE (Grading of Recommendations, Assessment, Development, and Evaluation) approach [10]. Additionally, this paper will reflect on the health equity considerations, research gaps, and limitations noted during guideline development, related to the integration of digital health technologies in clinical practice.

## Methods

### Development Approach

RNAO’s BPG development team used the GRADE approach to develop this guideline, which is in line with international reporting standards [10]. GRADE is a transparent and structured process to evaluate the certainty of a body of evidence from systematic reviews in order to develop sound, evidence-based recommendations in guidelines [10]. The systematic reviews were conducted in accordance with the PRISMA (Preferred Reporting Items for Systematic Reviews and Meta-Analyses) guidelines [11] (Checklist 1). The following section will describe how the purpose and scope of the guideline were determined, the 5 systematic reviews that were conducted, and the resulting 4 recommendations that were drafted following completion of the systematic reviews.

### Scoping the Best Practice Guideline

To determine the purpose and scope of this BPG, the guideline development team conducted an environmental scan on existing clinical guidelines on this topic and appraised those guidelines. Two guideline development methodologists (GDMs) determined inclusion or exclusion criteria and searched an established list of websites for guidelines and other relevant content (eg, quality standards) published between January 2016 and March 2021 (Multimedia Appendix 1). Expert panel members were also asked to suggest additional guidelines for review. Guidelines were reviewed for content, applicability to health provider scope of practice, accessibility, and quality. Each GDM individually evaluated guideline quality using the Appraisal of Guidelines for Research and Evaluation II instrument [12]. Through this process, it was determined that no guidelines had been developed addressing evidence-based recommendations in this unique and growing area, especially as it relates to nurses and clinical practice.

The team also completed a preliminary literature review to examine available evidence on digital health for nurses, including how digital health technologies are being integrated into the nursing process; how digital health technologies are facilitators and/or barriers for nurses when maintaining and advancing professional practice; and what outcomes are used to measure the impact of using digital health technologies in nursing practice. Two databases were searched for literature (CINAHL and MEDLINE) between January 2016 and May 2021. Screening for eligibility was conducted independently by 2 GDMs with conflicts resolved through consensus. Data extraction was completed for the included studies on a customized Microsoft Excel sheet developed by the GDMs. Elements of data extraction (such as study setting, intervention, and outcomes) were determined by the GDMs. An analysis of themes across the studies was synthesized, and the themes, interventions, and outcomes were later presented to the expert panel.

GDMs also conducted 22 key informant interviews and 2 discussion groups with diverse experts in the field. Key informants included people with lived experience, direct care health and social service providers, and researchers selected based on their knowledge and expertise related to the BPG topic. Snowball sampling was also used to recruit key informants. See Textbox 1 for a description of the questions asked during the interviews. For the discussion groups, 3 sessions were convened with a total of 18 nursing students, clinical informatics nurses, and frontline nurses to understand the needs of nurses within digital health environments. GDMs used inductive qualitative content analysis to analyze data collected from key informant interviews and discussion groups, and this information was also presented to the expert panel.

Textbox 1. **Key informant interview questions.**

- How can digital health technologies impact the quality of care a person receives?
- How can digital health technologies promote or hinder the therapeutic nurse-client relationship?
- In what ways can a digital health environment enhance or hinder patient care delivery for underserved populations?
- What skills or competencies do nurses require in order to maintain professional practice in a digital health environment?
- What skills and competencies do nurse leaders require in order to support the interface between nursing clinical practice and digital health technology?
- What challenges do nurses face when working in a digital health environment?
- What challenges or struggles do you face in your current practice related to the use or implementation of digital health technologies?
- What challenges do nurses face when trying to engage in the design, development, and evaluation of digital health environments?
- What policies or practices can help nurses maintain professional practice in a digital health environment?
- What outcomes should we explore in the literature to measure the impact of using digital health technologies in clinical nursing practice?
- What should the scope of this guideline be?
- What should this best practice guideline (BPG) address in order to be most useful in practice for nurses and people receiving care?
- Are there any last thoughts on what is important for us to consider when starting the development of this BPG?

### Identifying Priority Recommendation Questions and Outcomes

The BPG development team assembled a panel of 17 experts, including 2 cochairs, from nursing practice, research, education, and policy, as well as other members of the interprofessional team, and people with lived experience representing a range of sectors and practice areas. The BPG was supported by 2 cochairs with relevant clinical and research experience, one of whom was a doctorate-prepared registered nurse, and the other cochair led the pan-Canadian Electronic Health Record Clinical Engagement Strategy for 6 years at Canada Health Infoway. The expert panel also included representatives from different geographical areas, including rural, suburban, and urban. From July to December 2021, 4 panel meetings were held to determine the BPG’s purpose, scope, and research questions that informed the systematic reviews. During the first orientation meeting, the expert panel was introduced to RNAO’s BPG program, the systematic review process, and the GRADE approach. Additional electronic materials were also sent to the panel to familiarize them with the BPG development process and the GRADE approach. Declarations of conflicts of interest that might be construed as constituting a perceived and/or actual conflict were made by all members of the expert panel prior to their participation in guideline development work, and on an ongoing basis.

During the initial phase of the guideline development process, the expert panel prioritized 4 research questions and corresponding outcomes deemed most important to this topic. An amendment to the PROSPERO registration was made following these initial meetings, once the panel determined through email correspondence that a fifth research question should be added. Textbox 2 displays the final recommendation questions and outcomes that informed focused research questions for the systematic reviews.

Textbox 2. **Recommendation questions and outcomes in the clinical practice in a digital health environment best practice guideline.**

*Recommendation question 1:* Should practical (eg, hands-on) professional development education focused on the use of digital health technologies within an organization be recommended or not for all nurses?

*Outcomes*: nurse competence (with using technology), nurse acceptance of technology, nurse-sensitive outcomes (eg, falls, pressure injuries, and pain), nurse involvement in the technology life cycle, nurse confidence (with using technology), and nurse-person therapeutic relationship.

*Recommendation question 2:* Should education about relational care and interpersonal communication skills be recommended or not for nurses practicing in virtual care settings and in-person digital health environments?

*Outcomes*: person or caregiver or family experience or satisfaction, nurse competence (with using technology), nurse confidence (with using technology), nurse-person therapeutic relationship, and person or caregiver or family involvement and engagement in care.

*Recommendation question 3:* Should the implementation of interdisciplinary peer champion models in health service organizations be recommended or not to facilitate education for health providers on the use of digital health technologies?

*Outcomes*: health provider competence (with using technology), health provider adoption of technology, health provider confidence (with using technology), health provider sensitive outcomes (eg, pressure injuries and pain), and sustainability of education (ie, knowledge and skills retention).

*Recommendation question 4:* Should the use of predictive analytics software or systems (eg, command centers and risk assessment software tools) for nurses providing care in all practice settings be recommended or not to inform clinical decision-making and improve clinical outcomes?

*Outcomes*: proactive or anticipatory care, critical incidents, failure to rescue, consistent application of evidence-based practice, and nurse-sensitive outcomes (eg, falls, pressure injuries, and pain).

*Recommendation question 5:* Should a distributive model (vs no distributive model or any other type of change management model) be recommended to integrate digital health competencies into the professional practice roles and responsibilities of nurses at all levels within an organization?

*Outcomes*: nurse competence (with using technology), nurse engagement (with using, developing, acquiring, and participating in education about the technology), nurse confidence (with using technology), person or caregiver or family experience or satisfaction, and nurses being able to define what their role is.

### Systematic Retrieval of the Evidence

The systematic reviews for the guideline were registered with PROSPERO in 2022 (CRD42022321580). Upon consultation with the expert panel, 4 amendments were made to the original PROSPERO registered protocol. These included: (1) adding an additional database to search (IEEE Xplore) in April 2022, (2) adding an additional systematic review question (December 2022), (3) conducting indirect evidence searches (January 2023), and (4) publishing the final version of the guideline online (May 2024). All other systematic review methods followed the protocol outlined in the original PROSPERO registration.

Five separate systematic review search strategies were developed and run by an external health sciences librarian from the University Health Network after consulting with 2 GDMs (CB and LH). The systematic searches included peer-reviewed studies of any study design (eg, quantitative, qualitative, mixed methods, and systematic reviews) published in English from January 2017 to July 2022. The following databases were searched: MEDLINE, MEDLINE Epub Ahead of Print and In-Process, Embase, Emcare Nursing, Cochrane Central Register of Controlled Trials, Cochrane Database of Systematic Reviews, APA PsychInfo, CINAHL, and IEEE Xplore. Expert panel members were also asked to review their personal libraries for key studies not found through the above search strategies. For more details and the full search strategy used for each systematic review, please refer to Multimedia Appendix 2.

After conducting the initial searches, it was decided to look for further indirect evidence to support each question. Direct evidence comes from research that directly compares the interventions of interest when applied to the populations of interest and measures outcomes important to patients [13]. Evidence can be indirect if the population differs, the intervention differs, or outcomes differ from those of original interest [13]. The health science librarian conducted additional indirect evidence searches from January 2023 to March 2023 for systematic reviews published in English. The BPG team recognizes that direct evidence allows for more confidence in the results; however, in the absence of direct evidence, GRADE notes that indirect evidence can be used and downgraded accordingly [10,13]. The broader populations and interventions searched were considered sufficiently direct by the expert panel and in line with the original methodology. To ensure the most up-to-date evidence was included in the BPG, an update search was also conducted in English between January 2023 to January 2024 for recommendation questions 1 to 4. However, an update search for question 5 was not completed since a recommendation was not drafted for this area. For the full search strategies, see Multimedia Appendix 2.

### Eligibility Criteria

All search results from the librarian were uploaded into DistillerSR software (DistillerSR Inc). All steps of the systematic review process were completed by 2 GDMs (CB and LH for the initial search and CB and LB for the update search). Two GDMs independently completed title and abstract screening using standardized screening guides developed by the GDMs. Screening guides were reviewed by senior members of the RNAO team prior to use. Studies included at this stage had the full text reviewed independently by both GDMs. Final inclusion was deemed appropriate if studies answered the research question, included prioritized outcomes, were published in English, and were accessible for retrieval. See Textbox 3 for inclusion and exclusion criteria, and Multimedia Appendix 2 for further details. Disagreements were settled by consensus. For the initial systematic search, any study design was eligible to be included. For the updated indirect systematic searches, study designs were limited to systematic reviews and meta-analyses.

Textbox 3. **Inclusion and exclusion criteria.**

Inclusion criteria:

- A primary focus on the interventions of interest and the prioritized outcomes per research question
- A focus on digital health technologies
- Applicable to nurses or health providers providing care in all practice settings (including registered nurses, registered practical nurses, nursing students, and nurse practitioners)
- Applicable to all health or social service organizations, or academic institutions
- Published after January 2017
- Published in English
- Accessible for retrieval
- Conducted in any geographic region
- Peer-reviewed literature
- Any study design (eg, quantitative, qualitative, mixed methods, and systematic reviews), but when conducting the indirect searches, only systematic reviews and meta-analyses were included.

Exclusion criteria:

- Topic NOT related to the interventions or prioritized outcomes per research question
- Dissertations, commentaries, narratives, discussion papers, case studies, expert reports, consensus documents, and studies with no specific methodology
- Studies not published in English
- Unpublished literature (eg, gray literature)
- Studies published prior to 2017

### Data Extraction and Quality Appraisal

Data extraction was completed on the included studies for each research question. The included studies were divided between GDMs and each reviewer independently extracted details from their assigned studies using standardized Excel sheets that were developed by the RNAO team (Multimedia Appendix 3). Each Excel sheet had a designated outcome for which study details were recorded. Details such as the setting, intervention and control description, the outcome and how it was measured, and study results were recorded by 1 GDM. Any harms (such as adverse effects), information on values, preferences, and health equity were also recorded. The second GDM independently reviewed the extracted data for accuracy. Quality appraisal of each article was completed independently by each GDM. The Cochrane Risk of Bias 2.0 tool [14] was used to appraise randomized controlled trials (RCTs), the risk of bias in nonrandomized studies—of interventions (ROBINS-I) tool [15] was used to appraise nonrandomized studies, and the risk of bias in systematic reviews (ROBIS) tool [16] was used to appraise systematic reviews. If a systematic review received a low risk of bias score using the ROBIS tool, and the review’s authors completed a risk of bias assessment within the paper, those assessments were also considered when conducting the GRADE consensus. After quality appraisal was completed by both GDMs, GRADE consensus was completed to assess the certainty of evidence for each outcome for each research question. GRADE uses five categories to rate the certainty of evidence as high, moderate, low, and very low by examining (1) risk of bias, (2) inconsistency, (3) imprecision, (4) indirectness, and (5) publication bias [10]. After the 5 categories had been graded, a certainty of evidence was determined for each of the 4 drafted recommendations corresponding to the research questions.

### Drafting Recommendations in the BPG

As per the GRADE methodology, the GDMs created an evidence profile (EP) and evidence to decision (EtD) framework for each recommendation [4,10]. The EP outlined details regarding the certainty of evidence across outcomes and the GRADE domains (MultimediaAppendices 4^7^). The EtD frameworks provided a narrative summary of the evidence for draft recommendations, described the certainty of evidence, and provided details around values and preferences regarding the intervention, as well as health equity considerations found in the systematic reviews. Expert panel members were provided with the EPs and EtD frameworks to review prior to 3 (virtual) half-day meetings to determine the direction (ie, a recommendation for or against an intervention) and the strength (ie, strong or conditional) of the BPG’s recommendations. A conditional recommendation is one for which the desirable effects probably outweigh the undesirable effects, and there is a need to consider more carefully than usual the individual’s circumstances, values, and preferences [10]. If there was insufficient direct or indirect evidence to develop a recommendation, the expert panel also had the option not to proceed with a recommendation. The expert panel determined that current evidence was insufficient to assess the certainty of effects of a distributive model (recommendation question 5) compared to other types of change management models to integrate digital health competencies into the professional practice roles and responsibilities of nurses within an organization; thus, no recommendation was made.

The recommendations and draft BPG also underwent several rounds of internal and external review prior to publication [17]. External reviewers for RNAO BPGs are identified through a public call issued on the RNAO website [17]. For this BPG, the written external review process was completed between September 14, 2023, and October 23, 2023. External reviewers with diverse perspectives, such as nurses and health providers, administrators, researchers, educators, nursing students, and people with lived experience, provided direct feedback.

## Results

### Summary of Results

**Figure 1.**
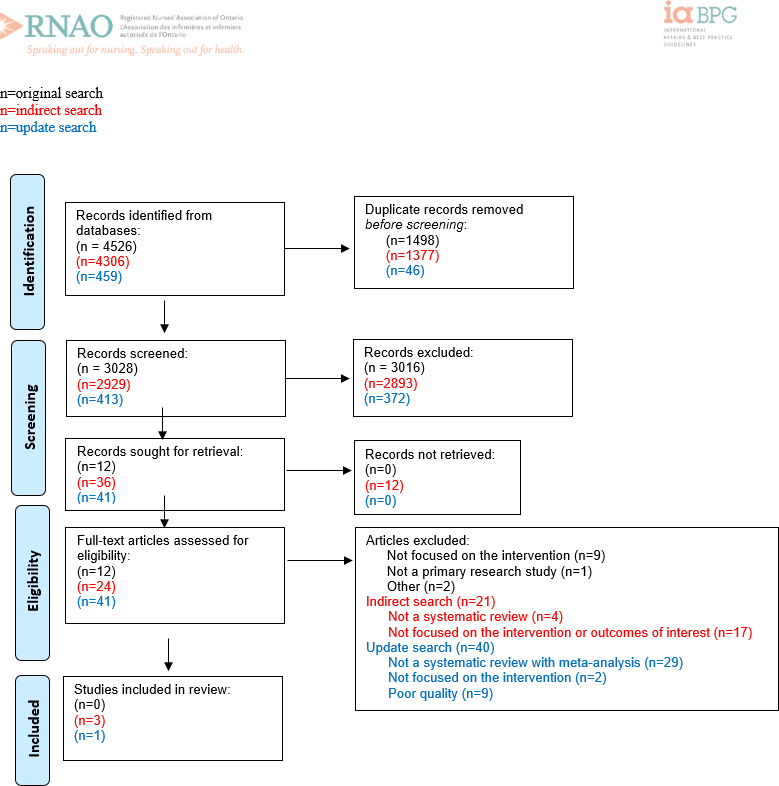
PRISMA (Preferred Reporting Items for Systematic Reviews and Meta-Analyses) flow diagram for recommendation question 1: “Should practical (eg, hands-on) professional development education be focused on the use of digital health technologies within an organization be recommended or not for all nurses?” Adapted from Page MJ et al [11].

For PRISMA flow diagrams, see Figures1^5^. Two reviewers screened over 22,500 articles for the 5 original research questions. After screening, the 2 GDMs reviewed 253 full-text articles for relevance to the research questions and outcomes, and 18 articles met the requirements to inform the recommendations. It was determined through consultation with the expert panel that question 5 did not have enough evidence to support the recommendation, so a recommendation was not developed. Thus, 4 recommendations were drafted (one per each of the corresponding systematic reviews), and the strength of the recommendations was determined through discussion and consensus by the expert panel, based on the available evidence (Table 1).

**Figure 2.**
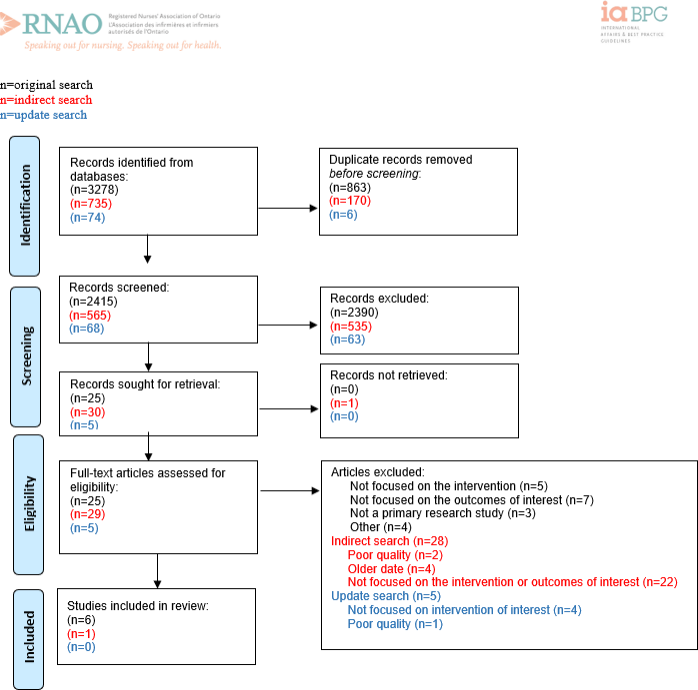
PRISMA (Preferred Reporting Items for Systematic Reviews and Meta-Analyses) flow diagram for recommendation question 2: “Should education about relational care and interpersonal communication skills be recommended or not for nurses practicing in virtual care settings and in-person digital health environments?” Adapted from Page MJ et al [11].

**Figure 3.**
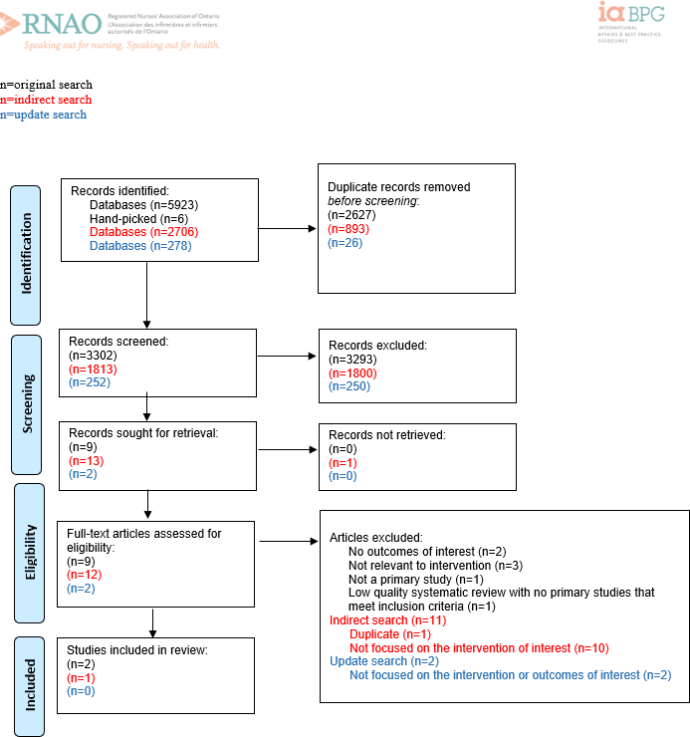
PRISMA (Preferred Reporting Items for Systematic Reviews and Meta-Analyses) flow diagram for recommendation question 3: “Should the implementation of interdisciplinary peer champion models in health service organizations be recommended or not to facilitate education for health providers on the use of digital health technologies?” Adapted from Page MJ et al [11].

**Figure 4.**
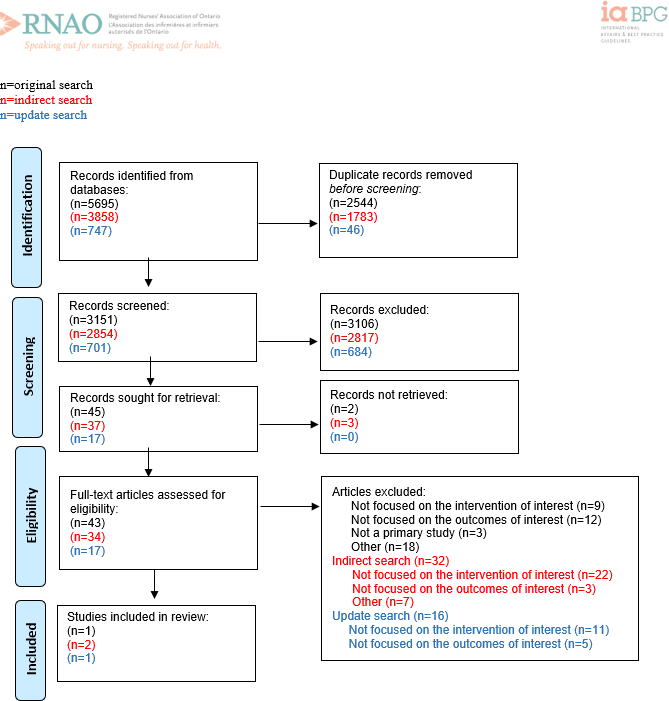
PRISMA (Preferred Reporting Items for Systematic Reviews and Meta-Analyses) flow diagram for recommendation question 4: “Should the use of predictive analytics software or systems (eg, command centers and risk assessment software tools) for nurses providing care in all practice settings be recommended or not to inform clinical decision-making and improve clinical outcomes?” Adapted from Page MJ et al [11].

**Figure 5.**
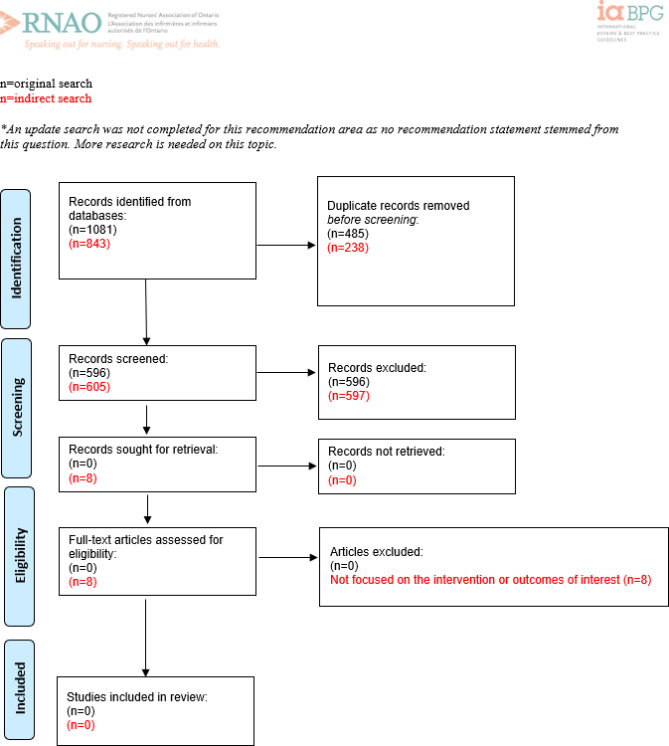
PRISMA (Preferred Reporting Items for Systematic Reviews and Meta-Analyses) flow diagram for recommendation question 5: “Should a distributive model (vs no distributive model or any other type of change management model) be recommended to integrate digital health competencies into the professional practice roles and responsibilities of nurses at all levels within an organization?” Adapted from Page MJ et al [11].

**Table 1.** Summary of recommendations in the best practice guideline.

Recommendation	Strength of recommendation
*Recommendation 1.0:* the expert panel suggests that health service and academic organizations provide ongoing education to nurses and health providers that includes hands-on training for the use of digital health technologies.	Conditional
*Recommendation 2.0:* the expert panel suggests that health service and academic organizations provide ongoing education to nurses and health providers that focuses on interpersonal communication skills when using digital health technologies.	Conditional
*Recommendation 3.0:* the expert panel suggests that health service organizations implement interdisciplinary peer champion models to facilitate education for nurses and health providers on the use of digital health technologies.	Conditional
*Recommendation 4.0:* the expert panel suggests that health service organizations implement CDSS^a^ or early warning systems that use artificial intelligence–driven predictive analytics to support nurses’ and health providers’ clinical decision-making.	Conditional

a CDSS: clinical decision support system.

### Recommendation 1.0: The Expert Panel Suggests That Health-Service and Academic Organizations Provide Ongoing Education to Nurses and Health Providers That Includes Hands-on Training for the Use of Digital Health Technologies

Practical or hands-on education refers to deliberate practice, hands-on training, or simulation training (ie, more than just viewing e-learning modules) [4]. The intervention of interest examined whether practical or hands-on education for professional development was more effective than standard education (ie, no-hands-on education component) when training nurses and health providers on the use of digital health technologies [4]. Four meta-analyses informed this recommendation [18-21]. The 4 meta-analyses were assessed for risk of bias using the ROBIS tool, and each one had a low risk of bias [18-21]. Studies included in the meta-analyses were assessed by the authors of the meta-analyses, and they used the Cochrane risk-of-bias 2.0 tool for RCTs, the ROBINS-I tool for nonrandomized studies, and the National Institute for Health and Care Excellence quality appraisal checklist [18-21]. Nine studies within the meta-analyses had a critical risk of bias, 18 studies had high risk of bias, 4 studies had unclear risk of bias, and 1 study had low risk of bias [18-21]. There were concerns noted around allocation concealment, blinding, incomplete outcome data, missing outcome data, selection of the reported results, confounding, allocation concealment, and selection of participants [18-21].

Examples of practical or hands-on professional development education discussed in the studies included nurses practicing using electronic health records while being supervised in a computer lab, and hands-on training for using virtual care platforms [18-21]. For more details on the study designs, the risk of bias assessments, how the interventions were delivered, and outcome measures, refer to the GRADE EP found in Multimedia Appendix 4.

The results of the systematic review suggest that hands-on education for nurses and health providers may improve nurses’ competence and confidence, and the nurse-person therapeutic relationship (while the technology is used with the person receiving care). The expert panel determined that the overall evidence was of very low certainty due to the risk of bias in the primary studies, indirectness in the outcomes, inconsistency in the results, and imprecision due to small sample sizes [4]. Based on this certainty of evidence, the panel determined the strength of the recommendation to be conditional.

### Recommendation 2.0: The Expert Panel Suggests That Health-Service and Academic Organizations Provide Ongoing Education to Nurses and Health Providers That Focuses on Interpersonal Communication Skills When Using Digital Health Technologies

Interpersonal communication describes the communication between a nurse or health provider and a person receiving care. It includes both verbal and nonverbal communication, as well as leading and listening skills that enable a person to interact positively with others in an effective manner [4,22]. The types of education varied across the studies and included didactic and simulation-based education (eg, simulated patients) to improve medical students’ interpersonal communication during consultations; training on incorporating computers or electronic health records into nurse-patient encounters; and education on telehealth communication strategies (eg, phone and video consults) [23-29]. Most studies examined focused on medical students [23-27,29], and 1 study focused on nursing students [28].

Seven studies informed this recommendation, including 1 systematic review, 5 additional nonrandomized studies, and 1 mixed methods study [23-29]. The review was assessed using the ROBIS tool and had a low risk of bias [23]. Studies included in the review were assessed by the review authors in accordance with the Cochrane Handbook for Systematic Reviews of Interventions; none were deemed as having a high risk of bias overall [23]. Nonrandomized studies and the mixed-methods study were assessed using the ROBINS-I tool, and there was a critical risk of bias related to confounding variables, deviations from the intended interventions, missing data, measurement of outcomes, and selection of the reported results [24-29].

The 7 studies illustrated that there may be benefits when health service and academic organizations provide nurses and other health providers with education about the importance of interpersonal communication when using digital health technologies [4,23-29]. Benefits may include improved person, caregiver, or family experience or satisfaction with care, and increased competence and confidence among nurses; however, the overall certainty of the evidence using the GRADE methodology was very low, due to risk of bias in the seven studies, few participants, and inconsistency in results [4]. Based on these factors, the expert panel determined the strength of the recommendation to be conditional. For more details on the study designs, risk of bias assessments, how the interventions were delivered, and outcome measures, refer to the GRADE EP in Multimedia Appendix 5.

### Recommendation 3.0: The Expert Panel Suggests That Health Service Organizations Implement Interdisciplinary Peer Champion Models to Facilitate Education for Nurses and Health Providers on the Use of Digital Health Technologies

Interdisciplinary peer champions refer to super-users or champions that are nurses or other members of the interdisciplinary health care team with expertise and additional training in digital health [4]. These individuals function as a resource for other staff, helping to answer questions and teach staff about new technology during implementation. Peer champions can also help identify gaps in the technology or its implementation in practice. This recommendation examined the effects of organizations implementing peer champion models to facilitate education for staff about digital health technologies.

One systematic review of 6 RCTs and 2 nonrandomized single-arm studies informed this recommendation [30-32]. The review was assessed using the ROBIS tool and had a low risk of bias [30]. Studies included in the review were assessed by the review authors using the Cochrane risk-of-bias tool for RCTs; 5 studies had a high risk of bias and 1 study had an unclear risk of bias [30]. The nonrandomized studies were assessed using the ROBINS-I tool, and there was a critical risk of bias related to confounding variables, missing data, measurement of the outcomes, and selection of the reported results [31,32].

The use of peer champions in health service organizations may increase health providers’ adoption of technology and health provider competence [4]. The overall certainty of evidence was low due to a serious risk of bias in the individual studies and a low number of participants [4]. Based on the available evidence, the expert panel determined the recommendation to be conditional. For more details on the study designs, risk of bias assessments, how the interventions were delivered, and outcome measures, refer to the GRADE EP in Multimedia Appendix 6.

### Recommendation 4.0: The Expert Panel Suggests That Health Service Organizations Implement Clinical Decision Support Systems or Early Warning Systems That Use AI-Driven Predictive Analytics to Support Nurses’ and Health Providers’ Clinical Decision-Making

CDSS or early warning systems refer to software found in risk assessment software tools, early warning systems, command centers, and other software systems that use AI machine learning algorithms to interpret data independently [4]. The recommendation question examined whether adding these systems benefits clinical decision-making for nurses and other health providers.

One systematic review of RCTs, 1 nonrandomized single-arm study, and 2 systematic reviews of nonrandomized studies informed this recommendation [33-36]. Included reviews were assessed using the ROBIS tool and had a low risk of bias [33,35,36]. Studies included in 1 review were assessed by the review authors using the Critical Appraisal Skills Programme checklist for RCTs; 2 studies had a low risk of bias and 1 study had a high risk of bias [33]. Concerns were noted around the lack of details describing the methods, and the lack of blinding [33]. Studies included in another review were assessed by the review authors using the Prediction model Risk Of Bias Assessment Tool; all 10 studies had high or unclear risk of bias [36]. The nonrandomized study was assessed using the ROBINS-I tool and had a critical risk of bias due to lack of control for confounding variables, deviations from the intended intervention, and selection of the reported results [34]. Studies in the final review were assessed by the review authors using the ROBINS-I tool; all 5 included studies had a critical risk of bias [35]. Concerns were noted around confounding, selection of participants, missing data, measurement of outcomes, and selection in reported results [35].

There may be benefits when implementing CDSS or early warning systems that use AI-driven predictive analytics to inform nurses’ clinical decision-making, such as improved proactive or anticipatory care, decreased failure to rescue, consistent application of evidence-based practice, and improved nurse-sensitive outcomes [4]. The overall certainty of evidence was low due to risk of bias and few participants [4]. As evidence is still emerging on this topic and the results were mixed, the expert panel determined the strength of the recommendation to be conditional. For more detail on the study designs, risk of bias assessments, how the interventions were delivered, and outcome measures, refer to the GRADE EP in Multimedia Appendix 7.

## Discussion

### Digital Health Considerations

In 2019, the World Health Organization released a global strategy on digital health acknowledging the vital role digital health plays in planning and providing health services [2]. As digital health technologies become increasingly integrated into health care, nurses need leadership and guidance to safely and effectively use technology in practice. RNAO’s BPG provides evidence-based recommendations to foster nurses’ ability to maintain, advance, and strengthen professional practice in the context of a digital health environment [4]. The guideline’s recommendations focus on (1) hands-on education related to the use of digital health technologies, (2) education about interpersonal communication skills when using digital health technologies, (3) using interdisciplinary peer-champion models to provide education about digital health technologies, and (4) implementing CDSS that uses AI to support but not replace clinical decision-making. While not discussed in this article, additional good practice statements are also provided in the guideline [4].

While digital health has the potential to enhance the quality of care and address key health system challenges, the importance of considering the digital determinants of health, including digital literacy and the digital divide, to ensure equitable delivery of care must be considered. Digital literacy refers to a person’s ability to effectively interact with digital technology, using skills required to find, understand, appraise, and apply health information specifically from electronic sources [37]. The digital divide refers to the gap between those who have access to digital technologies, including the internet, accessible health websites and portals, versus those who do not [38]. The World Health Organization’s global strategy on digital health notes that digital technologies are to be adaptable to different countries and contexts to help address key health system challenges, while incorporating equity, diversity, and inclusion principles [2]. Unfortunately, the use of certain digital health technologies such as CDSS that use AI may be difficult to implement in less affluent health care systems due to the digital divide [39]. The effectiveness of implementing CDSS that use AI to detect changes in a patient’s condition is also dependent on having staff who respond appropriately to these digital tools as well as nursing leadership to continuously oversee the refinement of CDSS and algorithms as needed. As outlined by Richardson et al [40] in their framework for digital health equity, there are several domains of equity including biological, behavioral, physical/built environment, sociocultural environment, and the health care system. The framework can help support the work of digital health technology developers to think about and incorporate principles of digital health equity from the very beginning of the technology development process [4,40]. The framework is also important for end-users, researchers, and health systems leaders, as digital health transformation requires health leaders at all levels to understand how the digital determinants impact health equity [4,40].

In addition to considering the digital determinants of health, when discussing the use of digital health technology with a person receiving care and/or their family, nurses must consider: their preferences and goals; capability and motivation for using technology; how the technology fits into their current care routines; and any costs associated with using the technology [4,41]. Digital health technologies have the potential to enhance a person’s experience of the care they receive [5]; however, nurses must consider a person’s values and preferences for using technology and ensure that using the technology does not negatively impact or compromise the nurse-patient therapeutic relationship [2,5].

### Implementation and Evaluation Considerations

Evidence-based guidelines are effective when there are tools and strategies in place to facilitate their implementation into practice [12]. RNAO uses an integrated approach to ensure that guidelines are both trustworthy and applicable in real-world settings [17]. This BPG includes several tools to support its implementation, including implementation tips, supporting resources, appendices related to the recommendations, and good practice statements. The BPG also directs readers to RNAO’s Leading Change Toolkit, which can be used to guide change initiatives, including the implementation of BPGs [42]. RNAO has a network of best practice champions who are the change agents that aid in the implementation of the guidelines, and Best Practice Spotlight Organizations^®^ (BPSO^®^) internationally from over 13 different countries that partner with RNAO to systematically implement and evaluate RNAO’s BPGs [17].

Finally, a monitoring and evaluation table outlines structure, process, and outcome indicators that health service organizations can use to monitor the impact of BPG implementation. Ongoing evaluation is crucial to support the uptake and impact of BPGs on person, organizational, and health systems outcomes [17]. RNAO houses 2 data systems to support BPSOs to monitor and evaluate BPGs: MyBPSO and Nursing Quality Indicators for Reporting and Evaluation^® ^[17]. These 2 data systems are used by BPSOs to report evaluation and monitoring data. As of November 2025, implementation of this BPG has begun in BPSOs, and 1 large Canadian community hospital BPSO has demonstrated 99% (555/562) of nursing staff were compliant with orientation to technologies. Evaluation has also indicated that 84% (474/562) of nurses at this hospital reported comfort with hospital-based technologies to deliver care, and 30% (20,188/72,342) of patients enrolled in a digital patient portal over an 18-month period. Evaluation and monitoring of outcomes is ongoing, and it is anticipated that in the coming years, more BPSOs will implement this valuable BPG.

### Future Research Considerations

The expert panel noted that although rigorous RCTs are needed, more exploration including qualitative research is also needed in the area of digital health as it pertains to nursing and clinical practice. For example, studies that examine the efficacy, accuracy, and generalizability of AI-driven predictive analytics, and qualitative studies exploring how nurses and health providers adapt their communication skills in digital health environments. National and international research institutes focused specifically on advancing digital health technologies and integrating digital health practices into clinical care for nurses and health providers would also be beneficial.

### Limitations

A few limitations were noted by the expert panel and GDMs during the development of the BPG. First, research in digital health that is specific to nurses and clinical practice is an emerging area. As research is yet to be well established, most evidence for the prioritized research questions was of low or very low certainty; thus, all the recommendations contained in the BPG were deemed conditional. There were few well-designed RCTs, and many of the nonrandomized studies had a high risk of bias, small sample sizes, and inconsistent results. In addition, due to the paucity of research evidence focused on nurses and digital health, the expert panel considered indirect evidence. According to the GRADE methodology, directness is assessed based on the relevance to the target population, intervention, and outcomes of interest [10]. Although GRADE methods allow for the use of indirect evidence, the reliance on indirect evidence due to insufficient direct evidence is a limitation in this BPG, recognizing that indirect evidence may introduce potential biases or uncertainties. The absence of research and use of indirect evidence is noted in the BPG as research gaps, stressing areas for further exploration.

Despite these limitations, expert panel members and additional external reviewers noted the need for guidance on this topic and the importance of publishing this guideline. Conditional recommendations are not to be seen as less important or less trustworthy; they simply imply that there is a need to consider more carefully than usual the individual person or family’s circumstances, preferences, and values [10]. When implementing conditional recommendations, health providers need to allocate more time to shared decision-making and comprehensively explain the potential benefits and harms to people and their families [10]. It is becoming increasingly common for clinical guidelines to only include conditional recommendations, as guideline panels and developers recognize the importance of thinking holistically [43,44]. As evidenced by the COVID-19 pandemic, guideline developers also must balance the need for guidance with rapidly evolving research topics [43]. In this BPG specifically, conditional recommendations allow for guidance on an emerging topic (clinical practice in a digital health environment) while recognizing the need for nurses and health providers to consider the implications within their own health care context. Additionally, it has been argued that the implementation of all recommendations, including strong recommendations, depends on social and relational processes governing decision-making for individuals [43]. With this argument in mind, end users of all guidelines should think about contextual implications and the values and preferences of patients when implementing both strong and conditional recommendations.

A final limitation is that the authors only included studies published in English from 2017 onwards. They did not search for gray literature or search reference lists of included studies for further evidence due to timelines and feasibility. Therefore, it is possible that some additional studies were missed.

### Conclusions

Digital health within the context of the clinical environment is an emerging topic. This BPG provides 4 evidence-based recommendations, along with good practice statements, implementation and evaluation, and monitoring resources. At the time of BPG development, no guidelines had been developed addressing evidence-based recommendations in this unique and growing area, especially as it relates to nurses and health providers. It is anticipated that this BPG can support nurses, other health providers, and health and academic organizations to make informed decisions about education and care related to digital health that can ultimately improve provider, patient, and system outcomes.

## Supplementary material

10.2196/74942 Multimedia Appendix 1 Guideline Search Strategy.

10.2196/74942 Multimedia Appendix 2 Systematic Review Search Strategies.

10.2196/74942 Multimedia Appendix 3 Sample Data Extraction Tables.

10.2196/74942 Multimedia Appendix 4 Recommendation 1 Evidence Profile.

10.2196/74942 Multimedia Appendix 5 Recommendation 2 Evidence Profile.

10.2196/74942 Multimedia Appendix 6 Recommendation 3 Evidence Profile.

10.2196/74942 Multimedia Appendix 7 Recommendation 4 Evidence Profile.

10.2196/74942 Checklist 1 PRISMA (Preferred Reporting Items for Systematic Reviews and Meta-Analyses) checklist.

## Abbreviations

AI: artificial intelligence
BPG: best practice guideline
BPSO: Best Practice Spotlight Organization
CDSS: clinical decision support system
EP: evidence profile
EtD: evidence to decision
GDM: guideline development methodologist
GRADE: Grading of Recommendations, Assessment, Development, and Evaluation
PRISMA: Preferred Reporting Items for Systematic Reviews and Meta-Analyses
PROSPERO: International Prospective Register of Systematic Reviews
RCT: randomized controlled trial
RNAO: Registered Nurses’ Association of Ontario
ROBINS-I: risk of bias in nonrandomized studies—of interventions
ROBIS: risk of bias in systematic reviews
