# Supplementary material for: Developing a Best Practice Guideline for Clinical Practice in a Digital Health Environment: Systematic Reviews Based on the Grading of Recommendations, Assessment, Development, and Evaluation Approach
Source: JMIR Nurs. 2026 Jan 23;9:e74942. doi: 10.2196/74942 (PMC12829893; doi:10.2196/74942)
Supplement: Multimedia Appendix 1 [file nursing-v9-e74942-s001.pdf]

Multimedia Appendix

Search Strategy for Guidelines and Standards

1. Structured Website Search

Two guideline development methodologists searched an established list of websites for guidelines and standards. Guidelines and standards were selected based on the following criteria:

- Guideline or standard in English
- Available and accessible for retrieval
- Dated no earlier than January 2016
- Relevant to nursing practice
  - Nurses caring for adult and pediatric populations
  - Registered nurses, nurse practitioners, and registered practical nurses
- Applicable to the Canadian context (e.g., studies in countries with similar culture and economic status/developed nations such as OECD countries https://www.oecd.org/about/document/ratification-oecd-convention.htm)
- Focused on the topic area:
  - Nurses working in digital health environments
  - Nurses implementing digital health technologies in clinical settings
  - Nurses involved in the design, development and/or evaluation of digital health technologies
  - Nurse leaders supporting the interface between nursing practice and digital health technology
- If the document was a guideline, it was:
  - Developed based on a systematic search of the literature
  - Contained a description of how the recommendations were developed
  - Included the word guideline or recommendation in the title or description

Websites searched:

- Trip medical database
- The Physiotherapy Evidence Database (PEDro)
- Occupational Therapy Systematic Evaluation of Evidence (OTseeker)
- National Institute for Health and Care Excellence (NICE)
- CPG Infobase: Clinical Practice Guideline Database
- Scottish Intercollegiate Guidelines Network (SIGN)
- National Health and Medical Research Council (NHMRC): Clinical Practice Guidelines
- eGuidelines Plus
- Guidelines International Network (GIN)
- Ontario Health
- Health Standards Organizatio (HSO)
- Google and Google Scholar
- Agency for Healthcare Research and Quality (AHRQ)
- Digital Health Canada

- Canadian Nursing Informatics Association (CNIA)
- Canada Health Infoway
- International Medical Informatics Association (IMIA)
- ECRI Institute
- World Health Organization (WHO)

2. Hand Search

RNAO expert panel members were asked to review personal libraries to identify and submit potentially relevant guidelines. Guidelines submitted for consideration by RNAO expert panel members are integrated into the retrieved list of guidelines if they have not been identified by the online guideline search. For this BPG, there were no relevant guidelines submitted by the expert panel.
