# Supplementary material for: Developing a Best Practice Guideline for Clinical Practice in a Digital Health Environment: Systematic Reviews Based on the Grading of Recommendations, Assessment, Development, and Evaluation Approach
Source: JMIR Nurs. 2026 Jan 23;9:e74942. doi: 10.2196/74942 (PMC12829893; doi:10.2196/74942)
Supplement: Multimedia Appendix 2 [file nursing-v9-e74942-s002.pdf]

Multimedia Appendix

Systematic Review Search Strategy

Originally, seven systematic reviews of recent literature relevant to the scope of the Best Practice Guideline (BPG) were conducted by a health sciences librarian. Five systematic reviews also had an indirect search completed. Both the original and the indirect search strategies for these five reviews are outlined in this document. Ultimately, four systematic reviews informed the recommendations in the BPG (one systematic review did not result in a recommendation; please refer to Appendix C of the full BPG for more detailed information).

In addition, expert panel members were asked to review their personal libraries to identify key articles not found through the above search strategies. Articles identified by panel members were included in the search results if two guideline development methodologists independently determined the articles had not been identified by the literature search and met the inclusion criteria.

Databases Searched: MEDLINE, MEDLINE Epub Ahead of Print and In-Process, Embase, Emcare Nursing, Cochrane Central Register of Controlled Trials, Cochrane Database of Systematic Reviews, APA PsychInfo, Cumulative Index to Nursing and Allied Health (CINAHL), and IEEE Explore.

Inclusion Criteria:

The following criteria were used to guide the literature search:

- A primary focus on the following interventions for health and social service organizations, academic institutions, health and social service providers and/or students, and the prioritized outcomes per topic:
  - Recommendation Question #1:
    - **Intervention –** practical (e.g., hands-on) professional development education focused on the use of digital health technologies
    - **Outcomes –** Nurse competence (with using technology), nurse acceptance of technology, nurse sensitive outcomes (i.e., falls, pressure injuries, pain), nurse involvement in the technology lifecycle, nurse confidence (with using technology), and nurse-person therapeutic relationship.
  - Recommendation Question # 2:
    - **Intervention** – education about relational care and interpersonal communication skills for nurses practicing in virtual care settings and in-person digital health environments
    - **Outcomes –** Person/caregiver/family experience or satisfaction, nurse competence (with using technology), nurse confidence (with using technology), nurse-person therapeutic relationship, and person/caregiver/family involvement and engagement in care.
  - Recommendation Question #3:
    - **Intervention –** interdisciplinary peer champion models to facilitate education for health providers on the use of digital health technologies
    - **Outcomes –** Health provider competence (with using technology), health provider adoption of technology, health provider confidence (with using technology), health

provider sensitive outcomes (i.e., falls, pressure injuries, pain), and sustainability of education (i.e., knowledge and skills retention).

- Recommendation Question #4:
  - **Intervention –** leveraging predictive analytics (e.g., command centers and risk assessment software tools) for nurses providing care in all practice settings
  - **Outcomes –** Proactive/anticipatory care, critical incidents, failure to rescue, consistent application of evidence-based practice, and nurse sensitive outcomes (i.e., falls, pressure injuries, pain).

- Recommendation Question #5:
  - **Intervention** – a distributive model to integrate digital health competencies into the professional practice roles and responsibilities of nurses at all levels within an organization
  - **Outcomes** – Nurse competence (with using technology), nurse engagement (with using, developing, acquiring, and participating in education about the technology), nurse confidence (with using technology]), person/caregiver/family experience or satisfaction, and nurses being able to define what their role is.

- A focus on digital health technologies
- Nurses providing care in all practice settings (including registered nurses, registered practical nurses, and nurse practitioners)
- Applicable to all health service organizations and health service providers
- Published after January 2017
- Published in English
- Accessible for retrieval
- Conducted in any geographic region
- Peer-reviewed literature
- Any study design* (e.g., quantitative, qualitative, mixed methods, systematic reviews)

*Note: when conducting the indirect searches, only systematic reviews and meta-analyses were included.

<u>Exclusion Criteria:</u>

The following criteria were used to exclude literature from the search:

- Topic **NOT** related to the aforementioned interventions or prioritized outcomes
- Dissertations, commentaries, narratives, discussion papers, case studies, expert reports, consenus documents, studies with no specific methodology
- Studies not published in English
- Upublished literature (e.g., grey literature)
- Studies published prior to 2017

<u>Search Terms:</u>

<u>Recommendation Question #1:</u> Should practical (e.g., hands-on) professional development education focused on the use of digital health technologies within an organization be recommended or not for all nurses?

Original Search Strategy:

Dates searched: January 2017 – February 4, 2022

Databases searched:

- MEDLINE
- MEDLINE Epub Ahead of Print and In-Process
- Embase
- Emcare Nursing
- Cochrane Central Register of Controlled Trials
- Cochrane Database of Systematic Reviews
- APA PsychInfo
- Cumulative Index to Nursing and Allied Health (CINAHL)
- IEEE Explore *(search run July 14, 2022)*

Database: MEDLINE
Search strategy:

#	Searches	Results
1	nurses/ or nurse administrators/ or nurse practitioners/ or family nurse practitioners/ or pediatric nurse practitioners/ or nurse specialists/ or nurse anesthetists/ or nurse clinicians/ or nurse midwives/ or nurses, pediatric/ or nurses, neonatal/ or nurses, community health/ or nurses, public health/	93019
2	nursing staff/ or nursing staff, hospital/	68777
3	nursing care/ or cardiovascular nursing/ or critical care nursing/ or developmental disability nursing/ or emergency nursing/ or geriatric nursing/ or holistic nursing/ or home nursing/ or "hospice and palliative care nursing"/ or maternal-child nursing/ or medical-surgical nursing/ or nephrology nursing/ or neuroscience nursing/ or nursing, practical/ or obstetric nursing/ or occupational health nursing/ or oncology nursing/ or orthopedic nursing/ or pediatric nursing/ or perioperative nursing/ or primary nursing/ or psychiatric nursing/ or rehabilitation nursing/ or trauma nursing/	127691
4	primary care nursing/	547
5	specialties, nursing/ or advanced practice nursing/ or cardiovascular nursing/ or community health nursing/ or home health nursing/ or parish nursing/ or critical care nursing/ or developmental disability nursing/ or emergency nursing/ or family nursing/ or forensic nursing/ or geriatric nursing/ or holistic nursing/ or "hospice and palliative care nursing"/ or maternal-child nursing/ or neonatal nursing/ or medical-surgical nursing/ or midwifery/ or military nursing/ or nephrology nursing/ or neuroscience	162077

	nursing/ or obstetric nursing/ or occupational health nursing/ or oncology nursing/ or orthopedic nursing/ or pediatric nursing/ or perioperative nursing/ or operating room nursing/ or postanesthesia nursing/ or psychiatric nursing/ or public health nursing/ or "radiologic and imaging nursing"/ or rehabilitation nursing/ or rural nursing/ or school nursing/ or transcultural nursing/ or trauma nursing/	
6	Nursing, Practical/	3442
7	Nurse's Role/	42224
8	nursing/ or nursing, private duty/ or nursing, supervisory/ or office nursing/ or telenursing/ or travel nursing/	60675
9	nursing process/ or nursing assessment/ or nursing diagnosis/	38375
10	nursing services/ or home care services/ or home health nursing/ or nursing service, hospital/	51191
11	Nursing, Team/	2590
12	Nursing Faculty Practice/	614
13	(nurse or nurses or nursing).tw,kf.	440312
14	or/1-13 [Nurses]	653578
15	Computer User Training/	2041
16	computer user training*.tw,kf.	6
17	Computer Assisted Instruction/	12320
18	(computer assisted instruction* or computerized programmed instruction* or computerized self instruction program*).tw,kf.	653
19	interactive tutorial/ or webcast/	1358
20	(interactive tutorial or interactive tutorials or webcast* or "web cast*").tw,kf.	310
21	InService Training/	20689
22	(inservice or "in service" or "on the job training" or "training on the job").tw,kf.	9017
23	Simulation Training/ or High Fidelity Simulation Training/ or Patient Simulation/	10958
24	(interactive learning or simulation or simulat*).tw,kf.	424763
25	"hands on".tw,kf.	6473
26	(practical adj3 (application or component* or element* or professional development or session*)).tw,kf.	13334
27	education, continuing/ or education, nursing, continuing/	32509

28	(continuing education or continuing nursing education or "post basic nursing education" or "post registration nursing education").tw,kf.	12863
29	or/15-28 [Practical or hands-on professional development education]	520445
30	14 and 29	38144
31	telemedicine/	32231
32	(telemedicine or telecardiology or teledermatology or telediagnosis or telediagnoses or telemonitoring or telenephrology or teleneurology or telepsychology or teleradiology or teleradiotherap* or telesurger* or teletherap* or videoconsult*).tw,kf.	19936
33	("tele medicine" or "tele cardiology" or "tele dermatology" or "tele diagnosis" or "tele diagnoses" or "tele monitoring" or "tele nephrology" or "tele neurology" or "tele psychology" or "tele radiology" or "tele radiotherap*" or "tele surger*" or "tele therap*" or "video consult*").tw,kf.	819
34	("e-health" or ehealth or "tele health").tw,kf.	6865
35	(telenurs* or "tele nurs*").tw,kf.	243
36	remote consultation/	5457
37	("cyber consult*" or cyberconsult* or econsult* or "e−consult*" or "email based consult*" or "internet consult*" or "internet based consult*" or "online consult*" or "tele consult*" or "telephone based consult*" or "telephone consult*" or "virtual consult*" or "web consult*" or "webbased consult*" or "web based consult*").tw,kf.	1576
38	telerehabilitation/	719
39	("e−rehabilitation" or remote rehabilitation tele rehabilitation or virtual rehabilitation).tw,kf.	134
40	medical records systems, computerized/	19131
41	(computerised medical record system* or computerized medical record system*).tw,kf.	94
42	electronic health records/	24408
43	(electronic health record* or computerised patient record* or computerized patient record* or electronic medical record* or electronic patient record* or ehr or emr).tw,kf.	41831
44	health smart cards/	59
45	(smart card or smart cards).tw,kf.	293
46	medical order entry systems/	2398
47	medical order entry system*.tw,kf.	83
48	hospital information systems/	11034

49	(hospital information system* or patient health record information system*).tw,kf.	2636
50	ambulatory care information systems/	1171
51	ambulatory care information system*.tw,kf.	15
52	electronic prescribing/	1130
53	("e–prescribing" or "e–prescription*" or electronic prescription*).tw,kf.	537
54	("c.p.o.e. system" or computer* order entry or computer* physician order entry or computer* provider order entry or computer* order entry or computer* prescriber order entry or computer* prescribing order entry or computer* prescription order entry or "cpoe").tw,kf.	1881
55	operating room information systems/	527
56	(operating room information system* or operating room information management system*).tw,kf.	28
57	Point-of-Care Systems/	15045
58	(bedside computing or bedside technolog* or point of care system* or point of care technolog* or point of care information system*).tw,kf.	708
59	bedside information system*.tw,kf.	10
60	(management information system* or management information).tw,kf.	1875
61	clinical pharmacy information systems/	1198
62	clinical pharmacy information system*.tw,kf.	15
63	database management systems/	7718
64	(database management system* or database management tool or database management tools or database manager system* or database managing system* or data base manager system* or data base management software or data base management system* or data base management tool or data base management tools).tw,kf.	587
65	decision support systems, management/	966
66	decision support system*.tw,kf.	6020
67	Mobile Applications/	9485
68	(mobile app or mobile apps or mobile application* or mobile health app or mobile health apps or mobile health application* or portable software app or portable software apps or portable software application* or tablet app or tablet apps or tablet application*).tw,kf.	5372

69	cell phone/	9479
70	(cell phone or cell phones or cellphone or cellphones or cellular phone or cellular phones or cellular telephone or cellular telephones or mobile phone or mobile phones or mobile telephone or mobile telephones).tw,kf.	10502
71	smartphone/	7318
72	(smartphone or smartphones or smart phones or smart phones).tw,kf.	12362
73	text messaging/	3946
74	(text messaging or texting).tw,kf.	2749
75	videoconferencing/	2183
76	(video conference* or video conferencing or videoconferenc*).tw,kf.	3278
77	webcast/	1079
78	webcasts as topic/	403
79	(webcast* or web cast*).tw,kf.	237
80	Wireless Technology/	4165
81	(wireless communication* or wireless technol*).tw,kf.	1343
82	"Cell Phone Use"/	326
83	("cell phone usage" or "cell phone utilisation" or "cell phone utilization" or "cellphone usage" or "cellphone use" or "cellphone utilisation " or "cellphone utilization" or "cell phone use" or "mobile phone usage" or "mobile phone use" or "mobile phone utilisation" or "mobile phone utilization").tw,kf.	1254
84	computers, handheld/	3934
85	(hand held computer* or handheld computer* or palm PC or palmtop or personal data assistant* or personal digital assistant or pocket computer* or pocket sized computer*).tw,kf.	1356
86	internet/	78307
87	internet.tw,kf.	49659
88	internet-based intervention/	849
89	(internet based intervention* or online based intervention* or online intervention* or web intervention* or web based intervention*).tw,kf.	2417
90	smart technolog*.tw,kf.	253
91	wearable electronic devices/	5471
92	(wearable computer or wearable computers or wearable electronic device*).tw,kf.	259

93	fitness trackers/	969
94	(activity tracker* or fitness tracker* or smart watch or smart watches or pedometer*).tw,kf.	3302
95	smart glasses/	117
96	("ar glasses" or "ar head mounted device" or "ar head mounted display" or "ar head worn display" or "ar headset" or "ar hud" or "arhmd" or "hmd ar" or "optical see through head mounted display" or "ost hmd" or "see through hmd" or "google glasses" or "smartglasses" or "smart glasses").tw,kf.	174
97	(augmented reality glasses or augmented reality head mounted device or augmented reality head mounted display or augmented reality head up display or augmented reality head worn display or head mounted display augmented reality).tw,kf.	37
98	artificial intelligence/	29580
99	(artificial intelligence or machine intelligence).tw,kf.	12258
100	expert systems/	3464
101	(expert system* or knowledge based system*).tw,kf.	3226
102	fuzzy logic/	4939
103	(fuzzy logic or fuzzy model or fuzzy models).tw,kf.	1730
104	machine learning/	21941
105	(machine learning or learning machine*).tw,kf.	36959
106	deep learning/	9379
107	(deep learning or hierarchical learning).tw,kf.	15601
108	unsupervised machine learning/	633
109	unsupervised machine learning.tw,kf.	474
110	natural language processing/	5278
111	natural language processing.tw,kf.	3817
112	neural networks, computer/	36523
113	("ann approach*" or "ann model" or "ann method*" or "ann training" or artificial neural network or computer neural network or computer neural networks).tw,kf.	6861
114	robotics/	23640
115	(robotics or nanorobotics or robot or robots).tw,kf.	23245
116	biomedical technology/	7060
117	(biomedical technology or bio medical technology).tw,kf.	372

118	informatics/	1121
119	informatics.tw,kf.	13971
120	medical informatics/	12686
121	(clinical informatics or clinical information science or clinical information technology or health informatics or health information science or health information technology or medical computer science or medical information science or medical informatics or medical information technology).tw,kf.	8441
122	medical informatics computing/	761
123	nursing informatics/	1597
124	nursing informatics.tw,kf.	837
125	health information exchange/	1037
126	health information exchange.tw,kf.	978
127	medical informatics applications/	2549
128	medical informatics applications.tw,kf.	108
129	decision making, computer-assisted/	2867
130	(decision support system* or decision support techniques).tw,kf.	6359
131	diagnosis, computer-assisted/	23725
132	(automatic diagnosis or computer assisted diagnosis or computer diagnosis or automatic diagnoses or computer assisted diagnoses or computer diagnoses).tw,kf.	1232
133	image interpretation, computer-assisted/	47479
134	computer assisted image interpretation.tw,kf.	21
135	radiographic image interpretation, computer-assisted/	15552
136	computer assisted radiographic image interpretation*.tw,kf.	16
137	therapy, computer-assisted/	6950
138	computer assisted therap*.tw,kf.	81
139	drug therapy, computer-assisted/	1690
140	computer assisted drug therap*.tw,kf.	16
141	decision support systems, clinical/	8929
142	clinical decision support system*.tw,kf.	2215
143	information systems/	19234
144	(information system or information systems).tw,kf.	32388

145	online systems/	8513
146	(online system or online systems).tw,kf.	542
147	user-computer interface/	38879
148	(computer interface* or computer user interface*).tw,kf.	5127
149	Social Media/	12541
150	(social media or social medium or Facebook or Flickr or Instagram or LinkedIn or MySpace or Pinterest or Reddit or "Sina Weibo" or Snapchat or online social network* or social networking platform* or social networking site* or social networking website* or social platform* or TikTok or Tumblr or Twitter or "web 2.0" or "web 2.0s" or WeChat or WhatsApp or YouTube).tw,kf.	20055
151	Virtual Reality/	3988
152	(virtual reality or virtual realities).tw,kf.	10142
153	Augmented Reality/	675
154	(augmented realities or augmented reality or mixed realities or mixed reality).tw,kf.	2241
155	holography/	2074
156	(hologram* or holograph*).tw,kf.	2776
157	Printing, Three-Dimensional/	8927
158	("3d printing*" or "3 d printing*" or "3 dimensional printing*" or "three dimensional printing*").tw,kf.	7017
159	(chatbot or chatbots or "chat bot" or "chat bots").tw,kf.	218
160	"virtual care".tw,kf.	420
161	(closed loop medicines system* or closed loop medication system* or closed loop medicines process* or closed loop medication process*).tw,kf.	2
162	(bedside station or bedside stations or bed side station or bed side stations or bedside terminal or bedside terminals or bed side terminal or bed side terminals).tw,kf.	55
163	predictive analytic*.tw,kf.	512
164	or/31-163	612220
165	30 and 164	3461
166	limit 165 to english language	3306
167	limit 166 to yr="2017 -Current"	727

Database: MEDLINE Epub Ahead of Print and In-Process
Search strategy:

#	Searches	Results
1	(nurse or nurses or nursing).tw,kf.	44536
2	computer user training*.tw,kf.	5
3	(computer assisted instruction* or computerized programmed instruction* or computerized self instruction program*).tw,kf.	52
4	(interactive tutorial or interactive tutorials or webcast* or "web cast*").tw,kf.	64
5	(inservice or "in service" or "on the job training" or "training on the job").tw,kf.	1668
6	(interactive learning or simulation or simulat*).tw,kf.	187347
7	"hands on".tw,kf.	1611
8	(practical adj3 (application or component* or element* or professional development or session*)).tw,kf.	5244
9	(continuing education or continuing nursing education or "post basic nursing education" or "post registration nursing education").tw,kf.	1073
10	or/2-9 [Practical or hands-on professional development education]	196173
11	1 and 10 [Nurses AND Practical or hands-on professional development education]	1429
12	(telemedicine or telecardiology or teledermatology or telediagnosis or telediagnoses or telemonitoring or telenephrology or teleneurology or telepsychology or teleradiology or teleradiotherap* or telesurger* or teletherap* or videoconsult*).tw,kf.	5022
13	("tele medicine" or "tele cardiology" or "tele dermatology" or "tele diagnosis" or "tele diagnoses" or "tele monitoring" or "tele nephrology" or "tele neurology" or "tele psychology" or "tele radiology" or "tele radiotherap*" or "tele surger*" or "tele therap*" or "video consult*").tw,kf.	302
14	("e-health" or ehealth or "tele health").tw,kf.	2203
15	(telenurs* or "tele nurs*").tw,kf.	58
16	("cyber consult*" or cyberconsult* or econsult* or "e–consult*" or "email based consult*" or "internet consult*" or "internet based consult*" or "online consult*" or "tele consult*" or "telephone based consult*" or "telephone consult*" or "virtual consult*" or "web consult*" or "webbased consult*" or "web based consult*").tw,kf.	452
17	("e–rehabilitation" or remote rehabilitation tele rehabilitation or virtual rehabilitation).tw,kf.	41

18	(computerised medical record system* or computerized medical record system*).tw,kf.	15
19	(electronic health record* or computerised patient record* or computerized patient record* or electronic medical record* or electronic patient record* or ehr or emr).tw,kf.	9747
20	(smart card or smart cards).tw,kf.	55
21	medical order entry system*.tw,kf.	28
22	(hospital information system* or patient health record information system*).tw,kf.	382
23	ambulatory care information system*.tw,kf.	6
24	("e−prescribing" or "e−prescription*" or electronic prescription*).tw,kf.	89
25	("c.p.o.e. system" or computer* order entry or computer* physician order entry or computer* provider order entry or computer* order entry or computer* prescriber order entry or computer* prescribing order entry or computer* prescription order entry or "cpoe").tw,kf.	216
26	(operating room information system* or operating room information management system*).tw,kf.	3
27	(bedside computing or bedside technolog* or point of care system* or point of care technolog* or point of care information system*).tw,kf.	163
28	(management information system* or management information).tw,kf.	285
29	clinical pharmacy information system*.tw,kf.	10
30	(database management system* or database management tool or database management tools or database manager system* or database managing system* or data base manager system* or data base management software or data base management system* or data base management tool or data base management tools).tw,kf.	92
31	decision support system*.tw,kf.	1204
32	(mobile app or mobile apps or mobile application* or mobile health app or mobile health apps or mobile health application* or portable software app or portable software apps or portable software application* or tablet app or tablet apps or tablet application*).tw,kf.	2832
33	(cell phone or cell phones or cellphone or cellphones or cellular phone or cellular phones or cellular telephone or cellular telephones or mobile phone or mobile phones or mobile telephone or mobile telephones).tw,kf.	3831
34	(smartphone or smartphones or smart phones or smart phones).tw,kf.	5580
35	(text messaging or texting).tw,kf.	811
36	(video conference* or video conferencing or videoconferenc*).tw,kf.	944

37	(webcast* or web cast*).tw,kf.	48
38	(wireless communication* or wireless technol*).tw,kf.	1346
39	("cell phone usage" or "cell phone utilisation" or "cell phone utilization" or "cellphone usage" or "cellphone use" or "cellphone utilisation " or "cellphone utilization" or "cell phone use" or "mobile phone usage" or "mobile phone use" or "mobile phone utilisation" or "mobile phone utilization").tw,kf.	294
40	(hand held computer* or handheld computer* or palm PC or palmtop or personal data assistant* or personal digital assistant or pocket computer* or pocket sized computer*).tw,kf.	120
41	internet.tw,kf.	13888
42	(internet based intervention* or online based intervention* or online intervention* or web intervention* or web based intervention*).tw,kf.	877
43	smart technolog*.tw,kf.	132
44	(wearable computer or wearable computers or wearable electronic device*).tw,kf.	331
45	(activity tracker* or fitness tracker* or smart watch or smart watches or pedometer*).tw,kf.	793
46	("ar glasses" or "ar head mounted device" or "ar head mounted display" or "ar head worn display" or "ar headset" or "ar hud" or "arhmd" or "hmd ar" or "optical see through head mounted display" or "ost hmd" or "see through hmd" or "google glasses" or "smartglasses" or "smart glasses").tw,kf.	112
47	(augmented reality glasses or augmented reality head mounted device or augmented reality head mounted display or augmented reality head up display or augmented reality head worn display or head mounted display augmented reality).tw,kf.	16
48	(artificial intelligence or machine intelligence).tw,kf.	7689
49	(expert system* or knowledge based system*).tw,kf.	444
50	(fuzzy logic or fuzzy model or fuzzy models).tw,kf.	974
51	(machine learning or learning machine*).tw,kf.	20976
52	(deep learning or hierarchical learning).tw,kf.	11178
53	unsupervised machine learning.tw,kf.	294
54	natural language processing.tw,kf.	1479
55	("ann approach*" or "ann model" or "ann method*" or "ann training" or artificial neural network or computer neural network or computer neural networks).tw,kf.	2524

56	(robotics or nanorobotics or robot or robots).tw,kf.	11366
57	(biomedical technology or bio medical technology).tw,kf.	99
58	informatics.tw,kf.	3242
59	(clinical informatics or clinical information science or clinical information technology or health informatics or health information science or health information technology or medical computer science or medical information science or medical informatics or medical information technology).tw,kf.	1847
60	nursing informatics.tw,kf.	62
61	health information exchange.tw,kf.	195
62	medical informatics applications.tw,kf.	39
63	(decision support system* or decision support techniques).tw,kf.	1329
64	(automatic diagnosis or computer assisted diagnosis or computer diagnosis or automatic diagnoses or computer assisted diagnoses or computer diagnoses).tw,kf.	243
65	computer assisted image interpretation.tw,kf.	3
66	computer assisted radiographic image interpretation*.tw,kf.	8
67	computer assisted therap*.tw,kf.	24
68	computer assisted drug therap*.tw,kf.	1
69	clinical decision support system*.tw,kf.	482
70	(information system or information systems).tw,kf.	5147
71	(online system or online systems).tw,kf.	150
72	(computer interface* or computer user interface*).tw,kf.	1562
73	(social media or social medium or Facebook or Flickr or Instagram or LinkedIn or MySpace or Pinterest or Reddit or "Sina Weibo" or Snapchat or online social network* or social networking platform* or social networking site* or social networking website* or social platform* or TikTok or Tumblr or Twitter or "web 2.0" or "web 2.0s" or WeChat or WhatsApp or YouTube).tw,kf.	8873
74	(virtual reality or virtual realities).tw,kf.	3272
75	(augmented realities or augmented reality or mixed realities or mixed reality).tw,kf.	1265
76	(hologram* or holograph*).tw,kf.	8947
77	("3d printing*" or "3 d printing*" or "3 dimensional printing*" or "three dimensional printing*").tw,kf.	5550
78	(chatbot or chatbots or "chat bot" or "chat bots").tw,kf.	178

79	"virtual care".tw,kf.	222
80	predictive analytic*.tw,kf.	278
81	or/12-80	120622
82	11 and 81	154
83	limit 82 to english language	150
84	limit 83 to yr="2017 -Current"	117

Database: Embase
Search strategy:

#	Searches	Results
1	nurse/ or expert nurse/ or licensed practical nurse/ or nurse consultant/ or practical nurse/ or registered nurse/ or staff nurse/	149051
2	nursing/ or practical nursing/ or telenursing/ or travel nursing/	236044
3	advanced practice nurse/	968
4	nurse administrator/ or charge nurse/ or head nurse/ or nurse manager/	15251
5	nurse practitioner/ or acute care nurse practitioner/ or adult nurse practitioner/ or emergency nurse practitioner/ or family nurse practitioner/ or gerontologic nurse practitioner/ or infection control practitioner/ or neonatal nurse practitioner/ or pediatric nurse practitioner/	27964
6	nurse specialist/ or clinical nurse specialist/ or neonatal nurse/ or nurse anesthetist/ or nurse midwife/ or oncology nurse/ or pediatric nurse/	14785
7	nursing staff/	75941
8	nursing care/ or visiting nursing service/	37743
9	nursing discipline/ or acquired immune deficiency syndrome nursing/ or addictions nursing/ or ambulatory care nursing/ or anesthesia nursing/ or burn nursing/ or camp nursing/ or cardiovascular nursing/ or dermatology nursing/ or emergency nursing/ or enterostomal therapy nursing/ or family nursing/ or flight nursing/ or forensic nursing/ or gastroenterology nursing/ or genetics nursing/ or gynecologic nursing/ or hospice nursing/ or intravenous nursing/ or learning disability nursing/ or military nursing/ or nephrology nursing/ or neuroscience nursing/ or nurse midwifery/ or obstetrical nursing/ or occupational health nursing/ or ophthalmic nursing/ or orthopedic nursing/	45732

	or palliative nursing/ or parish nursing/ or perinatal nursing/ or perioperative nursing/ or postanesthesia nursing/ or prison nursing/ or radiology nursing/ or rehabilitation nursing/ or respiratory nursing/ or rural health nursing/ or school health nursing/ or urologic nursing/	
10	community health nursing/ or community psychiatric nursing/	26907
11	community health nursing/ or community psychiatric nursing/	26907
12	geriatric nursing/ or psychogeriatric nursing/	13058
13	intensive care nursing/ or newborn intensive care nursing/ or pediatric intensive care nursing/	3054
14	newborn nursing/	3893
15	oncology nursing/ or pediatric oncology nursing/	8420
16	pediatric nursing/	13189
17	psychiatric nursing/	16896
18	surgical nursing/ or plastic surgical nursing/	279
19	advanced practice nursing/	2096
20	practical nursing/	157
21	nursing role/	654
22	nursing process/	7075
23	nursing assessment/	27389
24	nursing diagnosis/	4730
25	team nursing/	419
26	(nurse or nurses or nursing).tw,kw.	577020
27	or/1-26	804079
28	human computer interaction/	6491
29	computer user training*.tw,kw.	21
30	(computer assisted instruction* or computerized programmed instruction* or computerized self instruction program*).tw,kw.	958
31	webcast/	413
32	(interactive tutorial or interactive tutorials or webcast* or "web cast*").tw,kf.	616
33	"in service training"/	16682
34	(inservice or "in service" or "on the job training" or "training on the job").tw,kw.	13758

35	simulation/ or computer simulation/ or digital twin/ or discrete event simulation/ or high fidelity simulation/ or high-fidelity patient simulation/ or patient simulation/	338414
36	(interactive learning or simulation or simulat*).tw,kw.	661345
37	"hands on".tw,kw.	13314
38	(practical adj3 (application or component* or element* or professional development or session*)).tw,kw.	21773
39	continuing education/	32784
40	(continuing education or continuing nursing education or "post basic nursing education" or "post registration nursing education").tw,kw.	17567
41	or/28-40 [Practical or hands-on professional development education]	847456
42	27 and 41 [Nurses AND Practical or hands-on professional development education]	30513
43	telemedicine/ or telecardiology/ or teledermatology/ or telediagnosis/ or telemonitoring/ or telenephrology/ or teleneurology/ or telepsychology/ or teleradiology/ or teleradiotherapy/ or telesurgery/ or teletherapy/ or video consultation/	44194
44	(telemedicine or telecardiology or teledermatology or telediagnosis or telediagnoses or telemonitoring or telenephrology or teleneurology or telepsychology or teleradiology or teleradiotherap* or telesurger* or teletherap* or videoconsult*).tw,kw.	32689
45	("tele medicine" or "tele cardiology" or "tele dermatology" or "tele diagnosis" or "tele diagnoses" or "tele monitoring" or "tele nephrology" or "tele neurology" or "tele psychology" or "tele radiology" or "tele radiotherap*" or "tele surger*" or "tele therap*" or "video consult*").tw,kw.	1858
46	telehealth/	11872
47	("e-health" or ehealth or "tele health").tw,kw.	10265
48	telenursing/	316
49	(telenurs* or "tele nurs*").tw,kw.	324
50	teleconsultation/ or electronic consultation/	12994
51	("cyber consult*" or cyberconsult* or econsult* or "e–consult*" or "email based consult*" or "internet consult*" or "internet based consult*" or "online consult*" or "tele consult*" or "telephone based consult*" or "telephone consult*" or "virtual consult*" or "web consult*" or "webbased consult*" or "web based consult*").tw,kw.	3482
52	telerehabilitation/	1604

53	("e−rehabilitation" or remote rehabilitation or tele rehabilitation or virtual rehabilitation).tw,kw.	655
54	electronic medical record system/	1903
55	(computerised medical record system* or computerized medical record system*).tw,kw.	169
56	electronic health record/ or electronic medical record/ or electronic patient record/	95487
57	(electronic health record* or computerised patient record* or computerized patient record* or electronic medical record* or electronic patient record* or ehr or emr).tw,kw.	98134
58	smart card/	262
59	(smart card or smart cards).tw,kw.	447
60	physician order entry system/	314
61	medical order entry system*.tw,kw.	151
62	hospital information system/	20682
63	(hospital information system* or patient health record information system*).tw,kw.	4493
64	ambulatory care information system*.tw,kw.	39
65	electronic prescribing/	3630
66	("e−prescribing" or "e−prescription*" or electronic prescription*).tw,kw.	1333
67	computerized provider order entry/	2113
68	("c.p.o.e. system" or computer* order entry or computer* physician order entry or computer* provider order entry or computer* order entry or computer* prescriber order entry or computer* prescribing order entry or computer* prescription order entry or "cpoe").tw,kw.	3065
69	operating room information system/	35
70	(operating room information system* or operating room information management system*).tw,kw.	56
71	"point of care system"/	3078
72	(bedside computing or bedside technolog* or point of care system* or point of care technolog* or point of care information system*).tw,kw.	1208
73	bedside information system/	14
74	bedside information system*.tw,kw.	11
75	information system/	40577

76	(management information system* or management information).tw,kw.	2849
77	medical information system/	22247
78	clinical pharmacy information system*.tw,kw.	32
79	database management system/	561
80	(database management system* or database management tool or database management tools or database manager system* or database managing system* or data base manager system* or data base management software or data base management system* or data base management tool or data base management tools).tw,kw.	907
81	decision support system/	24730
82	decision support system*.tw,kw.	8801
83	mobile application/ or mobile health application/	18744
84	(mobile app or mobile apps or mobile application* or mobile health app or mobile health apps or mobile health application* or portable software app or portable software apps or portable software application* or tablet app or tablet apps or tablet application*).tw,kw.	9992
85	mobile phone/	19559
86	(cell phone or cell phones or cellphone or cellphones or cellular phone or cellular phones or cellular telephone or cellular telephones or mobile phone or mobile phones or mobile telephone or mobile telephones).tw,kw.	16926
87	smartphone/	19168
88	(smartphone or smartphones or smart phones or smart phones).tw,kw.	22896
89	text messaging/	6384
90	(text messaging or texting).tw,kw.	4402
91	videoconferencing/	6552
92	(video conference* or video conferencing or videoconferenc*).tw,kw.	5898
93	webcast/	413
94	(webcast* or web cast*).tw,kw.	479
95	wireless communication/	6370
96	(wireless communication* or wireless technol*).tw,kw.	2847
97	"cell phone use"/	1302

98	("cell phone usage" or "cell phone utilisation" or "cell phone utilization" or "cellphone usage" or "cellphone use" or "cellphone utilisation " or "cellphone utilization" or "cell phone use" or "mobile phone usage" or "mobile phone use" or "mobile phone utilisation" or "mobile phone utilization").tw,kw.	1860
99	personal digital assistant/	1676
100	(hand held computer* or handheld computer* or palm PC or palmtop or personal data assistant* or personal digital assistant or pocket computer* or pocket sized computer*).tw,kw.	1776
101	internet/	116160
102	internet.tw,kw.	83093
103	web-based intervention/	1469
104	(internet based intervention* or online based intervention* or online intervention* or web intervention* or web based intervention*).tw,kw.	3782
105	smart technolog*.tw,kw.	455
106	wearable computer/	852
107	(wearable computer or wearable computers or wearable electronic device*).tw,kw.	498
108	smart watch/ or activity tracker/	1871
109	(activity tracker* or fitness tracker* or smart watch or smart watches or pedometer*).tw,kw.	5577
110	smart glasses/	177
111	("ar glasses" or "ar head mounted device" or "ar head mounted display" or "ar head worn display" or "ar headset" or "ar hud" or "arhmd" or "hmd ar" or "optical see through head mounted display" or "ost hmd" or "see through hmd" or "google glasses" or "smartglasses" or "smart glasses").tw,kw.	341
112	artificial intelligence/	38465
113	(artificial intelligence or machine intelligence).tw,kw.	23872
114	computer heuristics/	348
115	computer heuristic*.tw,kw.	1
116	expert system/	5585
117	(expert system* or knowledge based system*).tw,kw.	5111
118	fuzzy logic/	4473
119	(fuzzy logic or fuzzy model or fuzzy models).tw,kw.	3419

120	machine learning/	52683
121	(machine learning or learning machine*).tw,kw.	68178
122	deep learning/	21576
123	(deep learning or hierarchical learning).tw,kw.	30683
124	unsupervised machine learning/	1447
125	unsupervised machine learning.tw,kw.	975
126	natural language processing/	7319
127	natural language processing.tw,kw.	6336
128	artificial neural network/	41935
129	("ann approach*" or "ann model" or "ann method*" or "ann training" or artificial neural network or computer neural network or computer neural networks).tw,kw.	11480
130	robotics/ or nanorobotics/	43416
131	(robotics or nanorobotics or robot or robots).tw,kw.	45603
132	medical technology/	36390
133	(biomedical technology or bio medical technology).tw,kw.	608
134	information science/	12858
135	informatics.tw,kw.	18509
136	medical informatics/	21914
137	(clinical informatics or clinical information science or clinical information technology or health informatics or health information science or health information technology or medical computer science or medical information science or medical informatics or medical information technology).tw,kw.	11573
138	medical informatics/	21914
139	nursing informatics/	1651
140	nursing informatics.tw,kw.	981
141	health information exchange.tw,kw.	1254
142	(decision support system* or decision support techniques).tw,kw.	9501
143	computer assisted diagnosis/	42599
144	(automatic diagnosis or computer assisted diagnosis or computer diagnosis or automatic diagnoses or computer assisted diagnoses or computer diagnoses).tw,kw.	2045
145	computer assisted image interpretation.tw,kw.	48

146	computer assisted radiographic image interpretation*.tw,kw.	28
147	computer assisted therapy/	4802
148	computer assisted therap*.tw,kw.	155
149	computer assisted drug therapy/	928
150	computer assisted drug therap*.tw,kw.	35
151	clinical decision support system/	4388
152	clinical decision support system*.tw,kw.	3371
153	information system/	40577
154	(information system or information systems).tw,kw.	47805
155	online system/	28847
156	(online system or online systems).tw,kw.	1030
157	computer interface/	34096
158	(computer interface* or computer user interface*).tw,kw.	7457
159	social media/	32906
160	(social media or social medium or Facebook or Flickr or Instagram or LinkedIn or MySpace or Pinterest or Reddit or "Sina Weibo" or Snapchat or online social network* or social networking platform* or social networking site* or social networking website* or social platform* or TikTok or Tumblr or Twitter or "web 2.0" or "web 2.0s" or WeChat or WhatsApp or YouTube).tw,kw.	37119
161	virtual reality/	21394
162	(virtual reality or virtual realities).tw,kw.	17182
163	augmented reality/	1170
164	(augmented realities or augmented reality or mixed realities or mixed reality).tw,kw.	4117
165	holography/	4201
166	(hologram* or holograph*).tw,kw.	6858
167	three dimensional printing/	16374
168	("3d printing*" or "3 d printing*" or "3 dimensional printing*" or "three dimensional printing*").tw,kw.	13195
169	(chatbot or chatbots or "chat bot" or "chat bots").tw,kw.	408
170	"virtual care".tw,kw.	733
171	(closed loop medicines system* or closed loop medication system* or closed loop medicines process* or closed loop medication process*).tw,kw.	6

172	(bedside station or bedside stations or bed side station or bed side stations or bedside terminal or bedside terminals or bed side terminal or bed side terminals).tw,kw.	57
173	predictive analytic?.tw,kw.	982
174	or/43-173	948975
175	42 and 174	3372
176	limit 175 to english language	3266
177	limit 176 to yr="2017 -Current"	988
178	limit 177 to (conference abstract or conference paper or "conference review")	274
179	177 not 178	714

Database: Emcare Nursing
Search strategy:

#	Searches	Results
1	nurse/ or expert nurse/ or licensed practical nurse/ or nurse consultant/ or practical nurse/ or registered nurse/ or staff nurse/	142569
2	nursing/ or practical nursing/ or telenursing/ or travel nursing/	99050
3	advanced practice nurse/	2258
4	nurse administrator/ or charge nurse/ or head nurse/ or nurse manager/	7986
5	nurse practitioner/ or acute care nurse practitioner/ or adult nurse practitioner/ or emergency nurse practitioner/ or family nurse practitioner/ or gerontologic nurse practitioner/ or infection control practitioner/ or neonatal nurse practitioner/ or pediatric nurse practitioner/	15271
6	nurse specialist/ or clinical nurse specialist/ or neonatal nurse/ or nurse anesthetist/ or nurse midwife/ or oncology nurse/ or pediatric nurse/	6108
7	nursing staff/	26650
8	nursing care/ or visiting nursing service/	17917
9	nursing discipline/ or acquired immune deficiency syndrome nursing/ or addictions nursing/ or ambulatory care nursing/ or anesthesia nursing/ or burn nursing/ or camp nursing/ or cardiovascular nursing/ or dermatology nursing/ or emergency nursing/ or enterostomal therapy nursing/ or family nursing/ or flight nursing/ or forensic nursing/ or gastroenterology nursing/ or genetics nursing/ or gynecologic nursing/ or hospice	14592

	nursing/ or intravenous nursing/ or learning disability nursing/ or military nursing/ or nephrology nursing/ or neuroscience nursing/ or nurse midwifery/ or obstetrical nursing/ or occupational health nursing/ or ophthalmic nursing/ or orthopedic nursing/ or palliative nursing/ or parish nursing/ or perinatal nursing/ or perioperative nursing/ or postanesthesia nursing/ or prison nursing/ or radiology nursing/ or rehabilitation nursing/ or respiratory nursing/ or rural health nursing/ or school health nursing/ or urologic nursing/	
10	community health nursing/ or community psychiatric nursing/	5219
11	community health nursing/ or community psychiatric nursing/	5219
12	geriatric nursing/ or psychogeriatric nursing/	2350
13	intensive care nursing/ or newborn intensive care nursing/ or pediatric intensive care nursing/	2006
14	newborn nursing/	2233
15	oncology nursing/ or pediatric oncology nursing/	2531
16	pediatric nursing/	2304
17	psychiatric nursing/	4274
18	surgical nursing/ or plastic surgical nursing/	445
19	advanced practice nursing/	1564
20	practical nursing/	186
21	nursing role/	1871
22	nursing process/	1721
23	nursing assessment/	4711
24	nursing diagnosis/	1697
25	team nursing/	181
26	(nurse or nurses or nursing).tw,kw.	287544
27	or/1-26	345030
28	human computer interaction/	4090
29	computer user training*.tw,kw.	13
30	(computer assisted instruction* or computerized programmed instruction* or computerized self instruction program*).tw,kw.	438
31	webcast/	84
32	(interactive tutorial or interactive tutorials or webcast* or "web cast*").tw,kf.	159

33	"in service training"/	1782
34	(inservice or "in service" or "on the job training" or "training on the job").tw,kw.	6719
35	simulation/ or computer simulation/ or digital twin/ or discrete event simulation/ or high fidelity simulation/ or high-fidelity patient simulation/ or patient simulation/	81300
36	(interactive learning or simulation or simulat*).tw,kw.	143539
37	"hands on".tw,kw.	4128
38	(practical adj3 (application or component* or element* or professional development or session*)).tw,kw.	5338
39	continuing education/	17788
40	(continuing education or continuing nursing education or "post basic nursing education" or "post registration nursing education").tw,kw.	7734
41	or/28-40 [Practical or hands-on professional development education]	192499
42	27 and 41 [Nurses AND Practical or hands-on professional development education]	13874
43	telemedicine/ or telecardiology/ or teledermatology/ or telediagnosis/ or telemonitoring/ or telenephrology/ or teleneurology/ or telepsychology/ or teleradiology/ or teleradiotherapy/ or telesurgery/ or teletherapy/ or video consultation/	16345
44	(telemedicine or telecardiology or teledermatology or telediagnosis or telediagnoses or telemonitoring or telenephrology or teleneurology or telepsychology or teleradiology or teleradiotherap* or telesurger* or teletherap* or videoconsult*).tw,kw.	14409
45	("tele medicine" or "tele cardiology" or "tele dermatology" or "tele diagnosis" or "tele diagnoses" or "tele monitoring" or "tele nephrology" or "tele neurology" or "tele psychology" or "tele radiology" or "tele radiotherap*" or "tele surger*" or "tele therap*" or "video consult*").tw,kw.	655
46	telehealth/	8829
47	("e-health" or ehealth or "tele health").tw,kw.	6885
48	telenursing/	295
49	(telenurs* or "tele nurs*").tw,kw.	333
50	teleconsultation/ or electronic consultation/	5183
51	("cyber consult*" or cyberconsult* or econsult* or "e−consult*" or "email based consult*" or "internet consult*" or "internet based consult*" or "online consult*" or	1163

	"tele consult*" or "telephone based consult*" or "telephone consult*" or "virtual consult*" or "web consult*" or "webbased consult*" or "web based consult*").tw,kw.	
52	telerehabilitation/	710
53	("e–rehabilitation" or remote rehabilitation or tele rehabilitation or virtual rehabilitation).tw,kw.	318
54	electronic medical record system/	514
55	(computerised medical record system* or computerized medical record system*).tw,kw.	107
56	electronic health record/ or electronic medical record/ or electronic patient record/	31711
57	(electronic health record* or computerised patient record* or computerized patient record* or electronic medical record* or electronic patient record* or ehr or emr).tw,kw.	29232
58	smart card/	261
59	(smart card or smart cards).tw,kw.	281
60	physician order entry system/	67
61	medical order entry system*.tw,kw.	146
62	hospital information system/	6854
63	(hospital information system* or patient health record information system*).tw,kw.	1879
64	ambulatory care information system*.tw,kw.	22
65	electronic prescribing/	1312
66	("e–prescribing" or "e–prescription*" or electronic prescription*).tw,kw.	445
67	computerized provider order entry/	1223
68	("c.p.o.e. system" or computer* order entry or computer* physician order entry or computer* provider order entry or computer* order entry or computer* prescriber order entry or computer* prescribing order entry or computer* prescription order entry or "cpoe").tw,kw.	1518
69	operating room information system/	16
70	(operating room information system* or operating room information management system*).tw,kw.	21
71	"point of care system"/	447
72	(bedside computing or bedside technolog* or point of care system* or point of care technolog* or point of care information system*).tw,kw.	476

73	bedside information system*.tw,kw.	6
74	information system/	18425
75	(management information system* or management information).tw,kw.	1162
76	medical information system/	13412
77	clinical pharmacy information system*.tw,kw.	18
78	database management system/	106
79	(database management system* or database management tool or database management tools or database manager system* or database managing system* or data base manager system* or data base management software or data base management system* or data base management tool or data base management tools).tw,kw.	277
80	decision support system/	10702
81	decision support system*.tw,kw.	4240
82	mobile application/ or mobile health application/	6745
83	(mobile app or mobile apps or mobile application* or mobile health app or mobile health apps or mobile health application* or portable software app or portable software apps or portable software application* or tablet app or tablet apps or tablet application*).tw,kw.	4956
84	mobile phone/	7627
85	(cell phone or cell phones or cellphone or cellphones or cellular phone or cellular phones or cellular telephone or cellular telephones or mobile phone or mobile phones or mobile telephone or mobile telephones).tw,kw.	7814
86	smartphone/	4473
87	(smartphone or smartphones or smart phones or smart phones).tw,kw.	9202
88	text messaging/	3115
89	(text messaging or texting).tw,kw.	2547
90	videoconferencing/	2866
91	(video conference* or video conferencing or videoconferenc*).tw,kw.	2869
92	webcast/	84
93	(webcast* or web cast*).tw,kw.	107
94	wireless communication/	1759
95	(wireless communication* or wireless technol*).tw,kw.	879

96	"cell phone use"/	655
97	("cell phone usage" or "cell phone utilisation" or "cell phone utilization" or "cellphone usage" or "cellphone use" or "cellphone utilisation " or "cellphone utilization" or "cell phone use" or "mobile phone usage" or "mobile phone use" or "mobile phone utilisation" or "mobile phone utilization").tw,kw.	929
98	personal digital assistant/	980
99	(hand held computer* or handheld computer* or palm PC or palmtop or personal data assistant* or personal digital assistant or pocket computer* or pocket sized computer*).tw,kw.	1030
100	internet/	54098
101	internet.tw,kw.	41739
102	web-based intervention/	726
103	(internet based intervention* or online based intervention* or online intervention* or web intervention* or web based intervention*).tw,kw.	2510
104	smart technolog*.tw,kw.	208
105	wearable computer/	41
106	(wearable computer or wearable computers or wearable electronic device*).tw,kw.	182
107	smart watch/ or activity tracker/	295
108	(activity tracker* or fitness tracker* or smart watch or smart watches or pedometer*).tw,kw.	2755
109	smart glasses/	25
110	("ar glasses" or "ar head mounted device" or "ar head mounted display" or "ar head worn display" or "ar headset" or "ar hud" or "arhmd" or "hmd ar" or "optical see through head mounted display" or "ost hmd" or "see through hmd" or "google glasses" or "smartglasses" or "smart glasses").tw,kw.	102
111	artificial intelligence/	7140
112	(artificial intelligence or machine intelligence).tw,kw.	6785
113	computer heuristics/	97
114	computer heuristic*.tw,kw.	1
115	expert system/	1924
116	(expert system* or knowledge based system*).tw,kw.	1310
117	fuzzy logic/	724

118	(fuzzy logic or fuzzy model or fuzzy models).tw,kw.	928
119	machine learning/	12866
120	(machine learning or learning machine*).tw,kw.	19053
121	deep learning/	5041
122	(deep learning or hierarchical learning).tw,kw.	8294
123	unsupervised machine learning/	284
124	unsupervised machine learning.tw,kw.	232
125	natural language processing/	2447
126	natural language processing.tw,kw.	2909
127	artificial neural network/	7528
128	("ann approach*" or "ann model" or "ann method*" or "ann training" or artificial neural network or computer neural network or computer neural networks).tw,kw.	2650
129	robotics/ or nanorobotics/	10764
130	(robotics or nanorobotics or robot or robots).tw,kw.	10000
131	medical technology/	12662
132	(biomedical technology or bio medical technology).tw,kw.	255
133	information science/	6716
134	informatics.tw,kw.	9410
135	medical informatics/	11578
136	(clinical informatics or clinical information science or clinical information technology or health informatics or health information science or health information technology or medical computer science or medical information science or medical informatics or medical information technology).tw,kw.	8428
137	medical informatics/	11578
138	nursing informatics/	1168
139	nursing informatics.tw,kw.	1050
140	health information exchange.tw,kw.	807
141	(decision support system* or decision support techniques).tw,kw.	4575
142	computer assisted diagnosis/	4363
143	(automatic diagnosis or computer assisted diagnosis or computer diagnosis or automatic diagnoses or computer assisted diagnoses or computer diagnoses).tw,kw.	583

144	computer assisted image interpretation.tw,kw.	25
145	computer assisted radiographic image interpretation*.tw,kw.	14
146	computer assisted therapy/	1253
147	computer assisted therap*.tw,kw.	98
148	computer assisted drug therapy/	166
149	computer assisted drug therap*.tw,kw.	15
150	clinical decision support system/	1782
151	clinical decision support system*.tw,kw.	1884
152	information system/	18425
153	(information system or information systems).tw,kw.	19445
154	online system/	11091
155	(online system or online systems).tw,kw.	397
156	computer interface/	7220
157	(computer interface* or computer user interface*).tw,kw.	2305
158	social media/	16019
159	(social media or social medium or Facebook or Flickr or Instagram or LinkedIn or MySpace or Pinterest or Reddit or "Sina Weibo" or Snapchat or online social network* or social networking platform* or social networking site* or social networking website* or social platform* or TikTok or Tumblr or Twitter or "web 2.0" or "web 2.0s" or WeChat or WhatsApp or YouTube).tw,kw.	20814
160	virtual reality/	10619
161	(virtual reality or virtual realities).tw,kw.	8325
162	augmented reality/	365
163	(augmented realities or augmented reality or mixed realities or mixed reality).tw,kw.	1562
164	holography/	587
165	(hologram* or holograph*).tw,kw.	1017
166	three dimensional printing/	3317
167	("3d printing*" or "3 d printing*" or "3 dimensional printing*" or "three dimensional printing*").tw,kw.	3088
168	(chatbot or chatbots or "chat bot" or "chat bots").tw,kw.	237
169	"virtual care".tw,kw.	330

170	(closed loop medicines system* or closed loop medication system* or closed loop medicines process* or closed loop medication process*).tw,kw.	2
171	(bedside station or bedside stations or bed side station or bed side stations or bedside terminal or bedside terminals or bed side terminal or bed side terminals).tw,kw.	7
172	predictive analytic?.tw,kw.	380
173	or/43-172	322003
174	42 and 173	1602
175	limit 174 to english language	1553
176	limit 175 to yr="2017 -Current"	463
177	limit 176 to (conference abstract or conference paper or "conference review")	10
178	176 not 177	453

Database: Cochrane Central Register of Controlled Trials
Search strategy:

#	Searches	Results
1	nurses/ or nurse administrators/ or nurse practitioners/ or family nurse practitioners/ or pediatric nurse practitioners/ or nurse specialists/ or nurse anesthetists/ or nurse clinicians/ or nurse midwives/ or nurses, pediatric/ or nurses, neonatal/ or nurses, community health/ or nurses, public health/	1313
2	nursing staff/ or nursing staff, hospital/	675
3	nursing care/ or cardiovascular nursing/ or critical care nursing/ or developmental disability nursing/ or emergency nursing/ or geriatric nursing/ or holistic nursing/ or home nursing/ or "hospice and palliative care nursing"/ or maternal-child nursing/ or medical-surgical nursing/ or nephrology nursing/ or neuroscience nursing/ or nursing, practical/ or obstetric nursing/ or occupational health nursing/ or oncology nursing/ or orthopedic nursing/ or pediatric nursing/ or perioperative nursing/ or primary nursing/ or psychiatric nursing/ or rehabilitation nursing/ or trauma nursing/	1617
4	primary care nursing/	35
5	specialties, nursing/ or advanced practice nursing/ or cardiovascular nursing/ or community health nursing/ or home health nursing/ or parish nursing/ or critical care nursing/ or developmental disability nursing/ or emergency nursing/ or family nursing/	2199

	or forensic nursing/ or geriatric nursing/ or holistic nursing/ or "hospice and palliative care nursing"/ or maternal-child nursing/ or neonatal nursing/ or medical-surgical nursing/ or midwifery/ or military nursing/ or nephrology nursing/ or neuroscience nursing/ or obstetric nursing/ or occupational health nursing/ or oncology nursing/ or orthopedic nursing/ or pediatric nursing/ or perioperative nursing/ or operating room nursing/ or postanesthesia nursing/ or psychiatric nursing/ or public health nursing/ or "radiologic and imaging nursing"/ or rehabilitation nursing/ or rural nursing/ or school nursing/ or transcultural nursing/ or trauma nursing/	
6	Nursing, Practical/	10
7	Nurse's Role/	368
8	nursing/ or nursing, private duty/ or nursing, supervisory/ or office nursing/ or telenursing/ or travel nursing/	121
9	nursing process/ or nursing assessment/ or nursing diagnosis/	568
10	nursing services/ or home care services/ or home health nursing/ or nursing service, hospital/	1944
11	Nursing, Team/	22
12	Nursing Faculty Practice/	4
13	(nurse or nurses or nursing).tw,kw.	44123
14	or/1-13 [Nurses]	46916
15	Computer User Training/	53
16	computer user training*.tw,kw.	6
17	Computer Assisted Instruction/	1264
18	(computer assisted instruction* or computerized programmed instruction* or computerized self instruction program*).tw,kw.	203
19	interactive tutorial/ or webcast/	0
20	(interactive tutorial or interactive tutorials or webcast* or "web cast*").tw,kw.	26
21	InService Training/	753
22	(inservice or "in service" or "on the job training" or "training on the job").tw,kw.	23098
23	Simulation Training/ or High Fidelity Simulation Training/ or Patient Simulation/	1132
24	(interactive learning or simulation or simulat*).tw,kw.	21648
25	"hands on".tw,kw.	6581

26	(practical adj3 (application or component* or element* or professional development or session*)).tw,kw.	598
27	education, continuing/ or education, nursing, continuing/	409
28	(continuing education or continuing nursing education or "post basic nursing education" or "post registration nursing education").tw,kw.	453
29	or/15-28 [Practical or hands-on professional development education]	53207
30	14 and 29	5608
31	telemedicine/	2598
32	(telemedicine or telecardiology or teledermatology or telediagnosis or telediagnoses or telemonitoring or telenephrology or teleneurology or telepsychology or teleradiology or teleradiotherap* or telesurger* or teletherap* or videoconsult*).tw,kw.	3555
33	("tele medicine" or "tele cardiology" or "tele dermatology" or "tele diagnosis" or "tele diagnoses" or "tele monitoring" or "tele nephrology" or "tele neurology" or "tele psychology" or "tele radiology" or "tele radiotherap*" or "tele surger*" or "tele therap*" or "video consult*").tw,kw.	253
34	("e-health" or ehealth or "tele health").tw,kw.	1320
35	(telenurs* or "tele nurs*").tw,kw.	150
36	remote consultation/	389
37	("cyber consult*" or cyberconsult* or econsult* or "e−consult*" or "email based consult*" or "internet consult*" or "internet based consult*" or "online consult*" or "tele consult*" or "telephone based consult*" or "telephone consult*" or "virtual consult*" or "web consult*" or "webbased consult*" or "web based consult*").tw,kw.	531
38	telerehabilitation/	151
39	("e−rehabilitation" or remote rehabilitation tele rehabilitation or virtual rehabilitation).tw,kw.	63
40	medical records systems, computerized/	199
41	(computerised medical record system* or computerized medical record system*).tw,kw.	8
42	electronic health records/	431
43	(electronic health record* or computerised patient record* or computerized patient record* or electronic medical record* or electronic patient record* or ehr or emr).tw,kw.	5862

44	health smart cards/	1
45	(smart card or smart cards).tw,kw.	17
46	medical order entry systems/	71
47	medical order entry system*.tw,kw.	0
48	hospital information systems/	47
49	(hospital information system* or patient health record information system*).tw,kw.	126
50	ambulatory care information systems/	25
51	ambulatory care information system*.tw,kw.	1
52	electronic prescribing/	25
53	("e−prescribing" or "e−prescription*" or electronic prescription*).tw,kw.	53
54	("c.p.o.e. system" or computer* order entry or computer* physician order entry or computer* provider order entry or computer* order entry or computer* prescriber order entry or computer* prescribing order entry or computer* prescription order entry or "cpoe").tw,kw.	135
55	operating room information systems/	3
56	(operating room information system* or operating room information management system*).tw,kw.	2
57	Point-of-Care Systems/	462
58	(bedside computing or bedside technolog* or point of care system* or point of care technolog* or point of care information system*).tw,kw.	95
59	bedside information system*.tw,kw.	0
60	(management information system* or management information).tw,kw.	167
61	clinical pharmacy information systems/	21
62	clinical pharmacy information system*.tw,kw.	3
63	database management systems/	14
64	(database management system* or database management tool or database management tools or database manager system* or database managing system* or data base manager system* or data base management software or data base management system* or data base management tool or data base management tools).tw,kw.	16
65	decision support systems, management/	8
66	decision support system*.tw,kw.	1656

67	Mobile Applications/	970
68	(mobile app or mobile apps or mobile application* or mobile health app or mobile health apps or mobile health application* or portable software app or portable software apps or portable software application* or tablet app or tablet apps or tablet application*).tw,kw.	3027
69	cell phone/	761
70	(cell phone or cell phones or cellphone or cellphones or cellular phone or cellular phones or cellular telephone or cellular telephones or mobile phone or mobile phones or mobile telephone or mobile telephones).tw,kw.	3770
71	smartphone/	593
72	(smartphone or smartphones or smart phones or smart phones).tw,kw.	5274
73	text messaging/	1060
74	(text messaging or texting).tw,kw.	2287
75	videoconferencing/	216
76	(video conference* or video conferencing or videoconferenc*).tw,kw.	1307
77	webcast/	0
78	webcasts as topic/	27
79	(webcast* or web cast*).tw,kw.	18
80	Wireless Technology/	49
81	(wireless communication* or wireless technol*).tw,kw.	128
82	"Cell Phone Use"/	11
83	("cell phone usage" or "cell phone utilisation" or "cell phone utilization" or "cellphone usage" or "cellphone use" or "cellphone utilisation " or "cellphone utilization" or "cell phone use" or "mobile phone usage" or "mobile phone use" or "mobile phone utilisation" or "mobile phone utilization").tw,kw.	3199
84	computers, handheld/	304
85	(hand held computer* or handheld computer* or palm PC or palmtop or personal data assistant* or personal digital assistant or pocket computer* or pocket sized computer*).tw,kw.	263
86	internet/	4112
87	internet.tw,kw.	10876
88	internet-based intervention/	292

89	(internet based intervention* or online based intervention* or online intervention* or web intervention* or web based intervention*).tw,kw.	2716
90	smart technolog*.tw,kw.	18
91	wearable electronic devices/	115
92	(wearable computer or wearable computers or wearable electronic device*).tw,kw.	17
93	fitness trackers/	134
94	(activity tracker* or fitness tracker* or smart watch or smart watches or pedometer*).tw,kw.	2491
95	smart glasses/	7
96	("ar glasses" or "ar head mounted device" or "ar head mounted display" or "ar head worn display" or "ar headset" or "ar hud" or "arhmd" or "hmd ar" or "optical see through head mounted display" or "ost hmd" or "see through hmd" or "google glasses" or "smartglasses" or "smart glasses").tw,kw.	39
97	(augmented reality glasses or augmented reality head mounted device or augmented reality head mounted display or augmented reality head up display or augmented reality head worn display or head mounted display augmented reality).tw,kw.	6
98	artificial intelligence/	199
99	(artificial intelligence or machine intelligence).tw,kw.	806
100	expert systems/	60
101	(expert system* or knowledge based system*).tw,kw.	198
102	fuzzy logic/	40
103	(fuzzy logic or fuzzy model or fuzzy models).tw,kw.	52
104	machine learning/	134
105	(machine learning or learning machine*).tw,kw.	1843
106	deep learning/	33
107	(deep learning or hierarchical learning).tw,kw.	712
108	unsupervised machine learning/	0
109	unsupervised machine learning.tw,kw.	27
110	natural language processing/	12
111	natural language processing.tw,kw.	218
112	neural networks, computer/	124

113	("ann approach*" or "ann model" or "ann method*" or "ann training" or artificial neural network or computer neural network or computer neural networks).tw,kw.	329
114	robotics/	712
115	(robotics or nanorobotics or robot or robots).tw,kw.	3589
116	biomedical technology/	21
117	(biomedical technology or bio medical technology).tw,kw.	365
118	informatics/	2
119	informatics.tw,kw.	551
120	medical informatics/	85
121	(clinical informatics or clinical information science or clinical information technology or health informatics or health information science or health information technology or medical computer science or medical information science or medical informatics or medical information technology).tw,kw.	457
122	medical informatics computing/	0
123	nursing informatics/	10
124	nursing informatics.tw,kw.	18
125	health information exchange/	9
126	health information exchange.tw,kw.	41
127	medical informatics applications/	23
128	medical informatics applications.tw,kw.	3
129	decision making, computer-assisted/	134
130	(decision support system* or decision support techniques).tw,kw.	1686
131	diagnosis, computer-assisted/	735
132	(automatic diagnosis or computer assisted diagnosis or computer diagnosis or automatic diagnoses or computer assisted diagnoses or computer diagnoses).tw,kw.	165
133	image interpretation, computer-assisted/	992
134	computer assisted image interpretation.tw,kw.	0
135	radiographic image interpretation, computer-assisted/	399
136	computer assisted radiographic image interpretation*.tw,kw.	0
137	therapy, computer-assisted/	1370
138	computer assisted therap*.tw,kw.	265

139	drug therapy, computer-assisted/	153
140	computer assisted drug therap*.tw,kw.	23
141	decision support systems, clinical/	430
142	clinical decision support system*.tw,kw.	374
143	information systems/	66
144	(information system or information systems).tw,kw.	1736
145	online systems/	161
146	(online system or online systems).tw,kw.	643
147	user-computer interface/	1330
148	(computer interface* or computer user interface*).tw,kw.	587
149	Social Media/	226
150	(social media or social medium or Facebook or Flickr or Instagram or LinkedIn or MySpace or Pinterest or Reddit or "Sina Weibo" or Snapchat or online social network* or social networking platform* or social networking site* or social networking website* or social platform* or TikTok or Tumblr or Twitter or "web 2.0" or "web 2.0s" or WeChat or WhatsApp or YouTube).tw,kw.	3126
151	Virtual Reality/	401
152	(virtual reality or virtual realities).tw,kw.	4316
153	Augmented Reality/	19
154	(augmented realities or augmented reality or mixed realities or mixed reality).tw,kw.	310
155	holography/	5
156	(hologram* or holograph*).tw,kw.	73
157	Printing, Three-Dimensional/	93
158	("3d printing*" or "3 d printing*" or "3 dimensional printing*" or "three dimensional printing*").tw,kw.	375
159	(chatbot or chatbots or "chat bot" or "chat bots").tw,kw.	80
160	"virtual care".tw,kw.	38
161	(closed loop medicines system* or closed loop medication system* or closed loop medicines process* or closed loop medication process*).tw,kw.	0
162	(bedside station or bedside stations or bed side station or bed side stations or bedside terminal or bedside terminals or bed side terminal or bed side terminals).tw,kw.	0
163	predictive analytic*.tw,kw.	64

164	or/31-163	61487
165	30 and 164	884
166	limit 165 to english language	532
167	limit 166 to yr="2017 -Current"	224

Database: Cochrane Database of Systematic Reviews
Search strategy:

#	Searches	Results
1	(nurse or nurses or nursing).mp.	3002
2	(nurse or nurses or nursing).mp.	3002
3	(computer assisted instruction* or computerized programmed instruction* or computerized self instruction program*).mp.	44
4	(interactive tutorial or interactive tutorials or webcast* or "web cast*").mp.	18
5	(inservice or "in service" or "on the job training" or "training on the job").mp.	118
6	(interactive learning or simulation or simulat*).mp.	445
7	"hands on".mp.	59
8	(practical adj3 (application or component* or element* or professional development or session*)).mp.	31
9	(continuing education or continuing nursing education or "post basic nursing education" or "post registration nursing education").mp.	60
10	or/2-9 [Practical or hands-on professional development education]	3312
11	1 and 10 [Nurses AND Practical or hands-on professional development education]	3002
12	(telemedicine or telecardiology or teledermatology or telediagnosis or telediagnoses or telemonitoring or telenephrology or teleneurology or telepsychology or teleradiology or teleradiotherap* or telesurger* or teletherap* or videoconsult*).mp.	154
13	("tele medicine" or "tele cardiology" or "tele dermatology" or "tele diagnosis" or "tele diagnoses" or "tele monitoring" or "tele nephrology" or "tele neurology" or "tele psychology" or "tele radiology" or "tele radiotherap*" or "tele surger*" or "tele therap*" or "video consult*").mp.	35
14	("e-health" or ehealth or "tele health").mp.	109

15	(telenurs* or "tele nurs*").mp.	25
16	("cyber consult*" or cyberconsult* or econsult* or "e−consult*" or "email based consult*" or "internet consult*" or "internet based consult*" or "online consult*" or "tele consult*" or "telephone based consult*" or "telephone consult*" or "virtual consult*" or "web consult*" or "webbased consult*" or "web based consult*").mp.	65
17	("e−rehabilitation" or remote rehabilitation tele rehabilitation or virtual rehabilitation).mp.	3
18	(computerised medical record system* or computerized medical record system*).mp.	3
19	(electronic health record* or computerised patient record* or computerized patient record* or electronic medical record* or electronic patient record* or ehr or emr).mp.	98
20	(smart card or smart cards).mp.	2
21	medical order entry system*.mp.	6
22	(hospital information system* or patient health record information system*).mp.	10
23	ambulatory care information system*.mp.	5
24	("e−prescribing" or "e−prescription*" or electronic prescription*).mp.	4
25	("c.p.o.e. system" or computer* order entry or computer* physician order entry or computer* provider order entry or computer* order entry or computer* prescriber order entry or computer* prescribing order entry or computer* prescription order entry or "cpoe").mp.	14
26	(operating room information system* or operating room information management system*).mp.	1
27	(bedside computing or bedside technolog* or point of care system* or point of care technolog* or point of care information system*).mp.	23
28	(management information system* or management information).mp.	87
29	clinical pharmacy information system*.mp.	3
30	(database management system* or database management tool or database management tools or database manager system* or database managing system* or data base manager system* or data base management software or data base management system* or data base management tool or data base management tools).mp.	4
31	decision support system*.mp.	71
32	(mobile app or mobile apps or mobile application* or mobile health app or mobile health apps or mobile health application* or portable software app or portable software apps or portable software application* or tablet app or tablet apps or tablet application*).mp.	61

33	(cell phone or cell phones or cellphone or cellphones or cellular phone or cellular phones or cellular telephone or cellular telephones or mobile phone or mobile phones or mobile telephone or mobile telephones).mp.	201
34	(smartphone or smartphones or smart phones or smart phones).mp.	131
35	(text messaging or texting).mp.	97
36	(video conference* or video conferencing or videoconferenc*).mp.	75
37	(webcast* or web cast*).mp.	18
38	(wireless communication* or wireless technol*).mp.	22
39	("cell phone usage" or "cell phone utilisation" or "cell phone utilization" or "cellphone usage" or "cellphone use" or "cellphone utilisation " or "cellphone utilization" or "cell phone use" or "mobile phone usage" or "mobile phone use" or "mobile phone utilisation" or "mobile phone utilization").mp.	6
40	(hand held computer* or handheld computer* or palm PC or palmtop or personal data assistant* or personal digital assistant or pocket computer* or pocket sized computer*).mp.	41
41	internet.mp.	893
42	(internet based intervention* or online based intervention* or online intervention* or web intervention* or web based intervention*).mp.	88
43	smart technolog*.mp.	3
44	(wearable computer or wearable computers or wearable electronic device*).mp.	3
45	(activity tracker* or fitness tracker* or smart watch or smart watches or pedometer*).mp.	77
46	("ar glasses" or "ar head mounted device" or "ar head mounted display" or "ar head worn display" or "ar headset" or "ar hud" or "arhmd" or "hmd ar" or "optical see through head mounted display" or "ost hmd" or "see through hmd" or "google glasses" or "smartglasses" or "smart glasses").mp.	0
47	(augmented reality glasses or augmented reality head mounted device or augmented reality head mounted display or augmented reality head up display or augmented reality head worn display or head mounted display augmented reality).mp.	0
48	(artificial intelligence or machine intelligence).mp.	33
49	(expert system* or knowledge based system*).mp.	13
50	(fuzzy logic or fuzzy model or fuzzy models).mp.	7

51	(machine learning or learning machine*).mp.	95
52	(deep learning or hierarchical learning).mp.	4
53	unsupervised machine learning.mp.	1
54	natural language processing.mp.	3
55	("ann approach*" or "ann model" or "ann method*" or "ann training" or artificial neural network or computer neural network or computer neural networks).mp.	2
56	(robotics or nanorobotics or robot or robots).mp.	65
57	(biomedical technology or bio medical technology).mp.	5
58	informatics.mp.	77
59	(clinical informatics or clinical information science or clinical information technology or health informatics or health information science or health information technology or medical computer science or medical information science or medical informatics or medical information technology).mp.	68
60	nursing informatics.mp.	11
61	health information exchange.mp.	1
62	medical informatics applications.mp.	11
63	(decision support system* or decision support techniques).mp.	82
64	(automatic diagnosis or computer assisted diagnosis or computer diagnosis or automatic diagnoses or computer assisted diagnoses or computer diagnoses).mp.	17
65	computer assisted image interpretation.mp.	0
66	computer assisted radiographic image interpretation*.mp.	0
67	computer assisted therap*.mp.	19
68	computer assisted drug therap*.mp.	3
69	clinical decision support system*.mp.	19
70	(information system or information systems).mp.	127
71	(online system or online systems).mp.	10
72	(computer interface* or computer user interface*).mp.	28
73	(social media or social medium or Facebook or Flickr or Instagram or LinkedIn or MySpace or Pinterest or Reddit or "Sina Weibo" or Snapchat or online social network* or social networking platform* or social networking site* or social networking website* or social platform* or TikTok or Tumblr or Twitter or "web 2.0" or "web 2.0s" or WeChat or WhatsApp or YouTube).mp.	144

74	(virtual reality or virtual realities).mp.	85
75	(augmented realities or augmented reality or mixed realities or mixed reality).mp.	5
76	(hologram* or holograph*).mp.	1
77	("3d printing*" or "3 d printing*" or "3 dimensional printing*" or "three dimensional printing*").mp.	0
78	(chatbot or chatbots or "chat bot" or "chat bots").mp.	1
79	"virtual care".mp.	0
80	predictive analytic*.mp.	0
81	or/12-80	1631
82	11 and 81	820
83	limit 82 to last 6 years	330
84	limit 83 to full systematic reviews	281

Database: APA PsychInfo
Search strategy:

#	Searches	Results
1	nurses/ or psychiatric nurses/ or public health service nurses/	33900
2	nursing/	24760
3	(nurse or nurses or nursing).tw,id.	106871
4	or/1-3	107418
5	human computer interaction/	11722
6	computer user training*.tw,id.	3
7	computer assisted instruction/ or computer supported collaborative learning/ or intelligent tutoring systems/	18134
8	electronic learning/ or adaptive learning/ or asynchronous learning/ or digital game-based learning/ or mobile learning/	3507
9	(computer assisted instruction* or computerized programmed instruction* or computerized self instruction program*).tw,id.	1866
10	(interactive tutorial or interactive tutorials or webcast* or "web cast*").tw,id.	121
11	inservice training/ or on the job training/	1127

12	(inservice or "in service" or "on the job training" or "training on the job").tw,id.	12991
13	simulation/ or simulation games/	22496
14	(interactive learning or simulation* or simulat*).tw,id.	75959
15	"hands on".tw,id.	4226
16	(practical adj3 (application or component* or element* or professional development or session*)).tw,id.	4200
17	continuing education/	1804
18	professional development/	21388
19	(continuing education or continuing nursing education or "post basic nursing education" or "post registration nursing education").tw,id.	4073
20	or/5-19	157436
21	4 and 20	5207
22	telemedicine/ or online therapy/ or teleconsultation/ or telepsychiatry/ or telepsychology/ or telerehabilitation/	9798
23	teleconferencing/	956
24	(telemedicine or telecardiology or teledermatology or telediagnosis or telediagnoses or telemonitoring or telenephrology or teleneurology or telepsychology or teleradiology or teleradiotherap* or telesurger* or teletherap* or videoconsult*).tw,id.	3273
25	("tele medicine" or "tele cardiology" or "tele dermatology" or "tele diagnosis" or "tele diagnoses" or "tele monitoring" or "tele nephrology" or "tele neurology" or "tele psychology" or "tele radiology" or "tele radiotherap*" or "tele surger*" or "tele therap*" or "video consult*").tw,id.	160
26	("e-health" or ehealth or "tele health").tw,id.	2488
27	(telenurs* or "tele nurs*").tw,id.	94
28	online therapy/	3529
29	("cyber consult*" or cyberconsult* or econsult* or "e−consult*" or "email based consult*" or "internet consult*" or "internet based consult*" or "online consult*" or "tele consult*" or "telephone based consult*" or "telephone consult*" or "virtual consult*" or "web consult*" or "webbased consult*" or "web based consult*").tw,id.	393
30	("e−rehabilitation" or remote rehabilitation or tele rehabilitation or virtual rehabilitation).tw,id.	105
31	(computerised medical record system* or computerized medical record system*).tw,id.	2

32	electronic health records/	1017
33	(electronic health record* or computerised patient record* or computerized patient record* or electronic medical record* or electronic patient record* or ehr or emr).tw,id.	5353
34	(smart card or smart cards).tw,id.	54
35	medical order entry system*.tw,id.	2
36	(hospital information system* or patient health record information system*).tw,id.	101
37	ambulatory care information system*.tw,id.	0
38	("e−prescribing" or "e−prescription*" or electronic prescription*).tw,id.	60
39	("c.p.o.e. system" or computer* order entry or computer* physician order entry or computer* provider order entry or computer* order entry or computer* prescriber order entry or computer* prescribing order entry or computer* prescription order entry or "cpoe").tw,id.	90
40	(operating room information system* or operating room information management system*).tw,id.	0
41	(bedside computing or bedside technolog* or point of care system* or point of care technolog* or point of care information system*).tw,id.	33
42	bedside information system*.tw,id.	0
43	information systems/	5777
44	(management information system* or management information).tw,id.	808
45	clinical pharmacy information system*.tw,id.	2
46	(database management system* or database management tool or database management tools or database manager system* or database managing system* or data base manager system* or data base management software or data base management system* or data base management tool or data base management tools).tw,id.	94
47	decision support systems/	3524
48	decision support system*.tw,id.	2119
49	mobile applications/	1606
50	(mobile app or mobile apps or mobile application* or mobile health app or mobile health apps or mobile health application* or portable software app or portable software apps or portable software application* or tablet app or tablet apps or tablet application*).tw,id.	2441

51	mobile devices/ or tablet computers/ or mobile phones/	8359
52	(cell phone or cell phones or cellphone or cellphones or cellular phone or cellular phones or cellular telephone or cellular telephones or mobile phone or mobile phones or mobile telephone or mobile telephones).tw,id.	5896
53	smartphones/	2370
54	smartphones/ or "smartphone use"/	2682
55	(smartphone or smartphones or smart phones or smart phones).tw,id.	5928
56	(text messaging or texting).tw,id.	2295
57	videoconferencing/ or video-based interventions/	871
58	(video conference* or video conferencing or videoconferenc*).tw,id.	2390
59	(webcast* or web cast*).tw,id.	81
60	wireless technologies/	576
61	(wireless communication* or wireless technol*).tw,id.	332
62	("cell phone usage" or "cell phone utilisation" or "cell phone utilization" or "cellphone usage" or "cellphone use" or "cellphone utilisation " or "cellphone utilization" or "cell phone use" or "mobile phone usage" or "mobile phone use" or "mobile phone utilisation" or "mobile phone utilization").tw,id.	975
63	(hand held computer* or handheld computer* or palm PC or palmtop or personal data assistant* or personal digital assistant or pocket computer* or pocket sized computer*).tw,id.	774
64	internet/	30121
65	internet.tw,id.	45342
66	(internet based intervention* or online based intervention* or online intervention* or web intervention* or web based intervention*).tw,id.	2283
67	smart technolog*.tw,id.	138
68	wearable devices/	450
69	(wearable computer or wearable computers or wearable electronic device*).tw,id.	84
70	(activity tracker* or fitness tracker* or smart watch or smart watches or pedometer*).tw,id.	1147
71	("ar glasses" or "ar head mounted device" or "ar head mounted display" or "ar head worn display" or "ar headset" or "ar hud" or "arhmd" or "hmd ar" or "optical see	55

	through head mounted display" or "ost hmd" or "see through hmd" or "google glasses" or "smartglasses" or "smart glasses").tw,id.	
72	artificial intelligence/	9342
73	(artificial intelligence or machine intelligence).tw,id.	6423
74	computer heuristic*.tw,id.	3
75	expert systems/	5883
76	(expert system* or knowledge based system*).tw,id.	2360
77	fuzzy logic/	1702
78	(fuzzy logic or fuzzy model or fuzzy models).tw,id.	935
79	machine learning/	9933
80	(machine learning or learning machine*).tw,id.	9672
81	(deep learning or hierarchical learning).tw,id.	2391
82	unsupervised machine learning.tw,id.	101
83	natural language processing/	702
84	natural language processing.tw,id.	1447
85	artificial neural networks/ or deep neural networks/	4713
86	("ann approach*" or "ann model" or "ann method*" or "ann training" or artificial neural network or computer neural network or computer neural networks).tw,id.	1082
87	robotics/ or avatars/ or social robotics/	7428
88	(robotics or nanorobotics or robot or robots).tw,id.	8369
89	(biomedical technology or bio medical technology).tw,id.	68
90	informatics.tw,id.	1693
91	(clinical informatics or clinical information science or clinical information technology or health informatics or health information science or health information technology or medical computer science or medical information science or medical informatics or medical information technology).tw,id.	1231
92	medical informatics.tw,id.	229
93	nursing informatics.tw,id.	84
94	health information exchange.tw,id.	126
95	(decision support system* or decision support techniques).tw,id.	2166
96	computer assisted diagnosis/	1611

97	(automatic diagnosis or computer assisted diagnosis or computer diagnosis or automatic diagnoses or computer assisted diagnoses or computer diagnoses).tw,id.	156
98	computer assisted image interpretation.tw,id.	0
99	computer assisted radiographic image interpretation*.tw,id.	0
100	computer assisted therapy/	1171
101	computer assisted therap*.tw,id.	109
102	computer assisted drug therap*.tw,id.	0
103	clinical decision support system*.tw,id.	243
104	information systems/	5777
105	(information system or information systems).tw,id.	8674
106	(online system or online systems).tw,id.	221
107	(computer interface* or computer user interface*).tw,id.	2620
108	social media/ or online social networks/	18393
109	(social media or social medium or Facebook or Flickr or Instagram or LinkedIn or MySpace or Pinterest or Reddit or "Sina Weibo" or Snapchat or online social network* or social networking platform* or social networking site* or social networking website* or social platform* or TikTok or Tumblr or Twitter or "web 2.0" or "web 2.0s" or WeChat or WhatsApp or YouTube).tw,id.	25904
110	virtual reality/ or augmented reality/	10170
111	(virtual reality or virtual realities).tw,id.	7530
112	(augmented realities or augmented reality or mixed realities or mixed reality).tw,id.	1272
113	(hologram* or holograph*).tw,id.	436
114	("3d printing*" or "3 d printing*" or "3 dimensional printing*" or "three dimensional printing*").tw,id.	133
115	(chatbot or chatbots or "chat bot" or "chat bots").tw,id.	201
116	"virtual care".tw,id.	60
117	(closed loop medicines system* or closed loop medication system* or closed loop medicines process* or closed loop medication process*).tw,id.	0
118	(bedside station or bedside stations or bed side station or bed side stations or bedside terminal or bedside terminals or bed side terminal or bed side terminals).tw,id.	1
119	predictive analytic?.tw,id.	190
120	or/22-119	178607

121	21 and 120	407
122	limit 121 to english language	400
123	limit 122 to yr="2017 -Current"	129
124	limit 123 to "0110 peer-reviewed journal"	93

Database: Cumulative Index to Nursing and Allied Health (CINAHL)
Search strategy:

#	Query	Limiters/Expanders	Results
S1	(MH "Nursing Manpower+")	Expanders - Apply equivalent subjects Search modes - Boolean/Phrase	263,515
S2	(MH "Nursing Role")	Expanders - Apply equivalent subjects Search modes - Boolean/Phrase	62,288
S3	(MH "Telenursing")	Expanders - Apply equivalent subjects Search modes - Boolean/Phrase	2,241
S4	(MH "Nursing Process")	Expanders - Apply equivalent subjects Search modes - Boolean/Phrase	3,641
S5	(MH "Nursing Assessment")	Expanders - Apply equivalent subjects Search modes - Boolean/Phrase	18,387
S6	(MH "Nursing Diagnosis")	Expanders - Apply equivalent subjects Search modes - Boolean/Phrase	4,718
S7	(MH "Nursing Service")	Expanders - Apply equivalent subjects Search modes - Boolean/Phrase	1,495
S8	(MH "Team Nursing") OR (MH "Nursing Care Delivery Systems") OR (MH "Differentiated Nursing Practice") OR (MH "Functional Nursing") OR (MH "Modular Nursing") OR (MH "Primary Nursing") OR (MH "Total Patient Care Nursing") OR (MH "Nursing Care")	Expanders - Apply equivalent subjects Search modes - Boolean/Phrase	28,984
S9	TI (nurse or nurses or nursing) OR AB (nurse or nurses or nursing)	Expanders - Apply equivalent subjects Search modes - Boolean/Phrase	592,584
S10	S1 OR S2 OR S3 OR S4 OR S5 OR S6 OR S7 OR S8 OR S9	Expanders - Apply equivalent subjects Search modes - Boolean/Phrase	728,448

S11	(MH "Computer User Training")	Expanders - Apply equivalent subjects Search modes - Boolean/Phrase	793
S12	TI (computer user training*) OR AB (computer user training*)	Expanders - Apply equivalent subjects Search modes - Boolean/Phrase	0
S13	(MH "Computer Assisted Instruction")	Expanders - Apply equivalent subjects Search modes - Boolean/Phrase	8,129
S14	TI (computer assisted instruction* or computerized programmed instruction* or computerized self instruction program*) OR AB (computer assisted instruction* or computerized programmed instruction* or computerized self instruction program*)	Expanders - Apply equivalent subjects Search modes - Boolean/Phrase	362
S15	(MH "Webcasts")	Expanders - Apply equivalent subjects Search modes - Boolean/Phrase	689
S16	TI (interactive tutorial or interactive tutorials or webcast* or "web cast*") OR AB (interactive tutorial or interactive tutorials or webcast* or "web cast*")	Expanders - Apply equivalent subjects Search modes - Boolean/Phrase	295
S17	(MH "Staff Development")	Expanders - Apply equivalent subjects Search modes - Boolean/Phrase	29,162
S18	TI (inservice or "in service" or "on the job training" or "training on the job") OR AB (inservice or "in service" or "on the job training" or "training on the job")	Expanders - Apply equivalent subjects Search modes - Boolean/Phrase	168,834
S19	(MH "Patient Simulation") OR (MH "Simulations") OR (MH "Vignettes")	Expanders - Apply equivalent subjects Search modes - Boolean/Phrase	26,447
S20	TI (interactive learning or simulation* or simulat*) OR AB (interactive learning or simulation* or simulat*)	Expanders - Apply equivalent subjects Search modes - Boolean/Phrase	63,174
S21	TI ("hands on") OR AB ("hands on")	Expanders - Apply equivalent subjects Search modes - Boolean/Phrase	18,678
S22	TI (practical N3 (application or component* or element* or professional development or session*)) OR AB (practical N3 (application or component* or element* or professional development or session*))	Expanders - Apply equivalent subjects Search modes - Boolean/Phrase	3,919

S23	(MH "Education, Post-RN") OR (MH "Education, Continuing") OR (MH "Education, Nursing, Continuing")	Expanders - Apply equivalent subjects Search modes - Boolean/Phrase	29,956
S24	TI (continuing education or continuing nursing education or "post basic nursing education" or "post registration nursing education") OR AB (continuing education or continuing nursing education or "post basic nursing education" or "post registration nursing education")	Expanders - Apply equivalent subjects Search modes - Boolean/Phrase	14,704
S25	S11 OR S12 OR S13 OR S14 OR S15 OR S16 OR S17 OR S18 OR S19 OR S20 OR S21 OR S22 OR S23 OR S24	Expanders - Apply equivalent subjects Search modes - Boolean/Phrase	332,408
S26	S10 AND S25	Expanders - Apply equivalent subjects Search modes - Boolean/Phrase	69,157
S27	(MH "Telemedicine") OR (MH "Telepathology") OR (MH "Teleradiology") OR (MH "Telenutrition")	Expanders - Apply equivalent subjects Search modes - Boolean/Phrase	15,214
S28	TI (telemedicine or telecardiology or teledermatology or telediagnosis or telediagnoses or telemonitoring or telenephrology or teleneurology or telepsychology or teleradiology or teleradiotherap* or telesurger* or teletherap* or videoconsult*) OR AB (telemedicine or telecardiology or teledermatology or telediagnosis or telediagnoses or telemonitoring or telenephrology or teleneurology or telepsychology or teleradiology or teleradiotherap* or telesurger* or teletherap* or videoconsult*)	Expanders - Apply equivalent subjects Search modes - Boolean/Phrase	8,867
S29	TI ("tele medicine" or "tele cardiology" or "tele dermatology" or "tele diagnosis" or "tele diagnoses" or "tele monitoring" or "tele nephrology" or "tele neurology" or "tele psychology" or "tele radiology" or "tele radiotherap*" or "tele surger*" or "tele	Expanders - Apply equivalent subjects Search modes - Boolean/Phrase	408

	therap*" or "video consult*") OR AB ("tele medicine" or "tele cardiology" or "tele dermatology" or "tele diagnosis" or "tele diagnoses" or "tele monitoring" or "tele nephrology" or "tele neurology" or "tele psychology" or "tele radiology" or "tele radiotherap*" or "tele surger*" or "tele therap*" or "video consult*")		
S30	(MH "Telehealth")	Expanders - Apply equivalent subjects Search modes - Boolean/Phrase	11,533
S31	TI ("e-health" or ehealth or "tele health") OR AB ("e-health" or ehealth or "tele health")	Expanders - Apply equivalent subjects Search modes - Boolean/Phrase	4,257
S32	(MH "Telenursing")	Expanders - Apply equivalent subjects Search modes - Boolean/Phrase	2,241
S33	TI (telenurs* or "tele nurs*") OR AB (telenurs* or "tele nurs*")	Expanders - Apply equivalent subjects Search modes - Boolean/Phrase	258
S34	(MH "Remote Consultation")	Expanders - Apply equivalent subjects Search modes - Boolean/Phrase	2,706
S35	TI ("cyber consult*" or cyberconsult* or econsult* or "e−consult*" or "email based consult*" or "internet consult*" or "internet based consult*" or "online consult*" or "tele consult*" or "telephone based consult*" or "telephone consult*" or "virtual consult*" or "web consult*" or "webbased consult*" or "web based consult*") OR AB ("cyber consult*" or cyberconsult* or econsult* or "e−consult*" or "email based consult*" or "internet consult*" or "internet based consult*" or "online consult*" or "tele consult*" or "telephone based consult*" or "telephone consult*" or "virtual consult*" or "web consult*" or "webbased consult*" or "web based consult*")	Expanders - Apply equivalent subjects Search modes - Boolean/Phrase	1,195
S36	(MH "Telerehabilitation")	Expanders - Apply equivalent subjects Search modes - Boolean/Phrase	392

S37	TI ("e−rehabilitation" or remote rehabilitation or tele rehabilitation or virtual rehabilitation) OR AB ("e−rehabilitation" or remote rehabilitation or tele rehabilitation or virtual rehabilitation)	Expanders - Apply equivalent subjects<br>Search modes - Boolean/Phrase	235
S38	TI (computerised medical record system* or computerized medical record system*) OR AB (computerised medical record system* or computerized medical record system*)	Expanders - Apply equivalent subjects<br>Search modes - Boolean/Phrase	33
S39	(MH "Electronic Health Records")	Expanders - Apply equivalent subjects<br>Search modes - Boolean/Phrase	27,784
S40	TI (electronic health record* or computerised patient record* or computerized patient record* or electronic medical record* or electronic patient record* or ehr or emr) OR AB (electronic health record* or computerised patient record* or computerized patient record* or electronic medical record* or electronic patient record* or ehr or emr)	Expanders - Apply equivalent subjects<br>Search modes - Boolean/Phrase	30,339
S41	(MH "Smart Cards")	Expanders - Apply equivalent subjects<br>Search modes - Boolean/Phrase	170
S42	TI (smart card or smart cards) OR AB (smart card or smart cards)	Expanders - Apply equivalent subjects<br>Search modes - Boolean/Phrase	134
S43	TI (medical order entry system*) OR AB (medical order entry system*)	Expanders - Apply equivalent subjects<br>Search modes - Boolean/Phrase	2
S44	(MH "Hospital Information Systems")	Expanders - Apply equivalent subjects<br>Search modes - Boolean/Phrase	3,354
S45	TI (hospital information system* or patient health record information system*) OR AB (hospital information system* or patient health record information system*)	Expanders - Apply equivalent subjects<br>Search modes - Boolean/Phrase	910
S46	(MH "Ambulatory Care Information Systems")	Expanders - Apply equivalent subjects<br>Search modes - Boolean/Phrase	316

S47	TI (ambulatory care information system*) OR AB (ambulatory care information system*)	Expanders - Apply equivalent subjects Search modes - Boolean/Phrase	4
S48	TI ("e−prescribing" or "e−prescription*" or electronic prescription*) OR AB ("e−prescribing" or "e−prescription*" or electronic prescription*)	Expanders - Apply equivalent subjects Search modes - Boolean/Phrase	780
S49	(MH "Electronic Order Entry")	Expanders - Apply equivalent subjects Search modes - Boolean/Phrase	3,439
S50	TI ("c.p.o.e. system" or "computer* order entry" or "computer* physician order entry" or "computer* provider order entry" or "computer* order entry" or "computer* prescriber order entry" or "computer* prescribing order entry" or "computer* prescription order entry" or "cpoe") OR AB ("c.p.o.e. system" or "computer* order entry" or "computer* physician order entry" or "computer* provider order entry" or "computer* order entry" or "computer* prescriber order entry" or "computer* prescribing order entry" or "computer* prescription order entry" or "cpoe")	Expanders - Apply equivalent subjects Search modes - Boolean/Phrase	1,361
S51	(MH "Operating Room Information Systems")	Expanders - Apply equivalent subjects Search modes - Boolean/Phrase	415
S52	TI (operating room information system* or operating room information management system*) OR AB (operating room information system* or operating room information management system*)	Expanders - Apply equivalent subjects Search modes - Boolean/Phrase	8
S53	(MH "Clinical Information Systems")	Expanders - Apply equivalent subjects Search modes - Boolean/Phrase	7,266
S54	TI (bedside computing or bedside technolog* or point of care system* or point of care technolog* or point of care information system*) OR AB (bedside computing or bedside technolog* or point	Expanders - Apply equivalent subjects Search modes - Boolean/Phrase	221

	of care system* or point of care technolog* or point of care information system*)		
S55	TI (clinical information system*) OR AB (clinical information system*)	Expanders - Apply equivalent subjects Search modes - Boolean/Phrase	809
S56	(MH "Management Information Systems")	Expanders - Apply equivalent subjects Search modes - Boolean/Phrase	1,808
S57	TI (management information system* or management information) OR AB (management information system* or management information)	Expanders - Apply equivalent subjects Search modes - Boolean/Phrase	907
S58	(MH "Clinical Pharmacy Information Systems")	Expanders - Apply equivalent subjects Search modes - Boolean/Phrase	1,177
S59	TI (clinical pharmacy information system*) OR AB (clinical pharmacy information system*)	Expanders - Apply equivalent subjects Search modes - Boolean/Phrase	1
S60	TI (database management system* or database management tool or database management tools or database manager system* or database managing system* or data base manager system* or data base management software or data base management system* or data base management tool or data base management tools) OR AB (database management system* or database management tool or database management tools or database manager system* or database managing system* or data base manager system* or data base management software or data base management system* or data base management tool or data base management tools)	Expanders - Apply equivalent subjects Search modes - Boolean/Phrase	90
S61	(MH "Decision Support Systems, Management")	Expanders - Apply equivalent subjects Search modes - Boolean/Phrase	491
S62	TI (decision support system*) OR AB (decision support system*)	Expanders - Apply equivalent subjects Search modes - Boolean/Phrase	2,578

S63	(MH "Mobile Applications")	Expanders - Apply equivalent subjects Search modes - Boolean/Phrase	10,191
S64	TI (mobile app or mobile apps or mobile application* or mobile health app or mobile health apps or mobile health application* or portable software app or portable software apps or portable software application* or tablet app or tablet apps or tablet application*) OR AB (mobile app or mobile apps or mobile application* or mobile health app or mobile health apps or mobile health application* or portable software app or portable software apps or portable software application* or tablet app or tablet apps or tablet application*)	Expanders - Apply equivalent subjects Search modes - Boolean/Phrase	4,166
S65	(MH "Cellular Phone")	Expanders - Apply equivalent subjects Search modes - Boolean/Phrase	2,104
S66	TI (cell phone or cell phones or cellphone or cellphones or cellular phone or cellular phones or cellular telephone or cellular telephones or mobile phone or mobile phones or mobile telephone or mobile telephones) OR AB (cell phone or cell phones or cellphone or cellphones or cellular phone or cellular phones or cellular telephone or cellular telephones or mobile phone or mobile phones or mobile telephone or mobile telephones)	Expanders - Apply equivalent subjects Search modes - Boolean/Phrase	6,265
S67	(MH "Smartphone")	Expanders - Apply equivalent subjects Search modes - Boolean/Phrase	3,503
S68	TI (smartphone or smartphones or smart phones or smart phones) OR AB (smartphone or smartphones or smart phones or smart phones)	Expanders - Apply equivalent subjects Search modes - Boolean/Phrase	8,206
S69	(MH "Text Messaging")	Expanders - Apply equivalent subjects Search modes - Boolean/Phrase	3,668

S70	TI (text messaging or texting) OR AB (text messaging or texting)	Expanders - Apply equivalent subjects Search modes - Boolean/Phrase	2,348
S71	(MH "Videoconferencing")	Expanders - Apply equivalent subjects Search modes - Boolean/Phrase	2,450
S72	TI (video conference* or video conferencing or videoconferenc*) OR AB (video conference* or video conferencing or videoconferenc*)	Expanders - Apply equivalent subjects Search modes - Boolean/Phrase	2,242
S73	(MH "Webcasts")	Expanders - Apply equivalent subjects Search modes - Boolean/Phrase	689
S74	TI (webcast* or web cast*) OR AB (webcast* or web cast*)	Expanders - Apply equivalent subjects Search modes - Boolean/Phrase	258
S75	TI (wireless communication* or wireless technol*) OR AB (wireless communication* or wireless technol*)	Expanders - Apply equivalent subjects Search modes - Boolean/Phrase	489
S76	(MH "Computers, Hand-Held")	Expanders - Apply equivalent subjects Search modes - Boolean/Phrase	4,629
S77	TI (hand held computer* or handheld computer* or palm PC or palmtop or personal data assistant* or personal digital assistant or pocket computer* or pocket sized computer*) OR AB (hand held computer* or handheld computer* or palm PC or palmtop or personal data assistant* or personal digital assistant or pocket computer* or pocket sized computer*)	Expanders - Apply equivalent subjects Search modes - Boolean/Phrase	1,049
S78	(MH "Internet")	Expanders - Apply equivalent subjects Search modes - Boolean/Phrase	52,935
S79	TI internet OR AB internet	Expanders - Apply equivalent subjects Search modes - Boolean/Phrase	33,903
S80	(MH "Internet-Based Intervention")	Expanders - Apply equivalent subjects Search modes - Boolean/Phrase	329
S81	TI (internet based intervention* or online based intervention* or online intervention* or web intervention* or web based intervention*) OR AB (internet based	Expanders - Apply equivalent subjects Search modes - Boolean/Phrase	1,738

	intervention* or online based intervention* or online intervention* or web intervention* or web based intervention*)		
S82	TI (smart technolog*) OR AB (smart technolog*)	Expanders - Apply equivalent subjects<br>Search modes - Boolean/Phrase	153
S83	TI (wearable computer or wearable computers or wearable electronic device*) OR AB (wearable computer or wearable computers or wearable electronic device*)	Expanders - Apply equivalent subjects<br>Search modes - Boolean/Phrase	37
S84	(MH "Fitness Trackers")	Expanders - Apply equivalent subjects<br>Search modes - Boolean/Phrase	276
S85	TI (activity tracker* or fitness tracker* or smart watch or smart watches or pedometer*) OR AB (activity tracker* or fitness tracker* or smart watch or smart watches or pedometer*)	Expanders - Apply equivalent subjects<br>Search modes - Boolean/Phrase	2,349
S86	(MH "Smart Glasses")	Expanders - Apply equivalent subjects<br>Search modes - Boolean/Phrase	18
S87	TI ("ar glasses" or "ar head mounted device" or "ar head mounted display" or "ar head worn display" or "ar headset" or "ar hud" or "arhmd" or "hmd ar" or "optical see through head mounted display" or "ost hmd" or "see through hmd" or "google glasses" or "smartglasses" or "smart glasses") OR AB ("ar glasses" or "ar head mounted device" or "ar head mounted display" or "ar head worn display" or "ar headset" or "ar hud" or "arhmd" or "hmd ar" or "optical see through head mounted display" or "ost hmd" or "see through hmd" or "google glasses" or "smartglasses" or "smart glasses")	Expanders - Apply equivalent subjects<br>Search modes - Boolean/Phrase	78
S88	(MH "Artificial Intelligence")	Expanders - Apply equivalent subjects<br>Search modes - Boolean/Phrase	6,426
S89	TI (artificial intelligence or machine intelligence) OR AB (artificial intelligence or machine intelligence)	Expanders - Apply equivalent subjects<br>Search modes - Boolean/Phrase	5,187

S90	TI (computer heuristic*) OR AB (computer heuristic*)	Expanders - Apply equivalent subjects Search modes - Boolean/Phrase	0
S91	(MH "Expert Systems")	Expanders - Apply equivalent subjects Search modes - Boolean/Phrase	534
S92	TI (expert system* or knowledge based system*) OR AB (expert system* or knowledge based system*)	Expanders - Apply equivalent subjects Search modes - Boolean/Phrase	556
S93	TI (fuzzy logic or fuzzy model or fuzzy models) OR AB (fuzzy logic or fuzzy model or fuzzy models)	Expanders - Apply equivalent subjects Search modes - Boolean/Phrase	302
S94	(MH "Machine Learning")	Expanders - Apply equivalent subjects Search modes - Boolean/Phrase	2,758
S95	TI (machine learning or learning machine*) OR AB (machine learning or learning machine*)	Expanders - Apply equivalent subjects Search modes - Boolean/Phrase	8,961
S96	(MH "Deep Learning")	Expanders - Apply equivalent subjects Search modes - Boolean/Phrase	853
S97	TI (deep learning or hierarchical learning) OR AB (deep learning or hierarchical learning)	Expanders - Apply equivalent subjects Search modes - Boolean/Phrase	3,239
S98	TI (unsupervised machine learning) OR AB (unsupervised machine learning)	Expanders - Apply equivalent subjects Search modes - Boolean/Phrase	128
S99	(MH "Natural Language Processing")	Expanders - Apply equivalent subjects Search modes - Boolean/Phrase	2,420
S100	TI (natural language processing) OR AB (natural language processing)	Expanders - Apply equivalent subjects Search modes - Boolean/Phrase	1,641
S101	(MH "Neural Networks (Computer)")	Expanders - Apply equivalent subjects Search modes - Boolean/Phrase	2,912
S102	TI ("ann approach*" or "ann model" or "ann method*" or "ann training" or artificial neural network or computer neural network or computer neural networks) OR AB ("ann approach*" or "ann model" or "ann method*" or "ann training" or artificial	Expanders - Apply equivalent subjects Search modes - Boolean/Phrase	1,352

	neural network or computer neural network or computer neural networks)		
S103	(MH "Robotics")	Expanders - Apply equivalent subjects Search modes - Boolean/Phrase	8,496
S104	TI (robotics or nanorobotics or robot or robots) OR AB (robotics or nanorobotics or robot or robots)	Expanders - Apply equivalent subjects Search modes - Boolean/Phrase	14,037
S105	TI (biomedical technology or bio medical technology) OR AB (biomedical technology or bio medical technology)	Expanders - Apply equivalent subjects Search modes - Boolean/Phrase	123
S106	(MH "Informatics")	Expanders - Apply equivalent subjects Search modes - Boolean/Phrase	1,348
S107	TI informatics OR AB informatics	Expanders - Apply equivalent subjects Search modes - Boolean/Phrase	10,950
S108	(MH "Medical Informatics") OR (MH "Nursing Informatics") OR (MH "Health Informatics")	Expanders - Apply equivalent subjects Search modes - Boolean/Phrase	12,584
S109	TI (clinical informatics or clinical information science or clinical information technology or health informatics or health information science or health information technology or medical computer science or medical information science or medical informatics or medical information technology) OR AB (clinical informatics or clinical information science or clinical information technology or health informatics or health information science or health information technology or medical computer science or medical information science or medical informatics or medical information technology)	Expanders - Apply equivalent subjects Search modes - Boolean/Phrase	8,034
S110	(MH "Electronic Data Interchange")	Expanders - Apply equivalent subjects Search modes - Boolean/Phrase	3,842
S111	TI (health information exchange) OR AB (health information exchange)	Expanders - Apply equivalent subjects Search modes - Boolean/Phrase	1,022

S112	(MH "Decision Making, Computer Assisted")	Expanders - Apply equivalent subjects Search modes - Boolean/Phrase	1,366
S113	TI (decision support system* or decision support techniques) OR AB (decision support system* or decision support techniques)	Expanders - Apply equivalent subjects Search modes - Boolean/Phrase	2,582
S114	(MH "Diagnosis, Computer Assisted")	Expanders - Apply equivalent subjects Search modes - Boolean/Phrase	4,713
S115	TI (automatic diagnosis or computer assisted diagnosis or computer diagnosis or automatic diagnoses or computer assisted diagnoses or computer diagnoses) OR AB (automatic diagnosis or computer assisted diagnosis or computer diagnosis or automatic diagnoses or computer assisted diagnoses or computer diagnoses)	Expanders - Apply equivalent subjects Search modes - Boolean/Phrase	165
S116	(MH "Image Interpretation, Computer Assisted")	Expanders - Apply equivalent subjects Search modes - Boolean/Phrase	9,404
S117	TI (computer assisted image interpretation) OR AB (computer assisted image interpretation)	Expanders - Apply equivalent subjects Search modes - Boolean/Phrase	1
S118	(MH "Radiographic Image Interpretation, Computer-Assisted")	Expanders - Apply equivalent subjects Search modes - Boolean/Phrase	4,492
S119	TI (computer assisted radiographic image interpretation*) OR AB (computer assisted radiographic image interpretation*)	Expanders - Apply equivalent subjects Search modes - Boolean/Phrase	0
S120	(MH "Therapy, Computer Assisted") OR (MH "Drug Therapy, Computer Assisted")	Expanders - Apply equivalent subjects Search modes - Boolean/Phrase	5,946
S121	TI (computer assisted therap*) OR AB (computer assisted therap*)	Expanders - Apply equivalent subjects Search modes - Boolean/Phrase	36
S122	TI (computer assisted drug therap*) OR AB (computer assisted drug therap*)	Expanders - Apply equivalent subjects Search modes - Boolean/Phrase	4
S123	(MH "Decision Support Systems, Clinical")	Expanders - Apply equivalent subjects Search modes - Boolean/Phrase	5,962

S124	TI (clinical decision support system*) OR AB (clinical decision support system*)	Expanders - Apply equivalent subjects Search modes - Boolean/Phrase	1,220
S125	(MH "Information Systems")	Expanders - Apply equivalent subjects Search modes - Boolean/Phrase	4,870
S126	TI (information system or information systems) OR AB (information system or information systems)	Expanders - Apply equivalent subjects Search modes - Boolean/Phrase	13,829
S127	(MH "Online Systems")	Expanders - Apply equivalent subjects Search modes - Boolean/Phrase	1,864
S128	TI (online system or online systems) OR AB (online system or online systems)	Expanders - Apply equivalent subjects Search modes - Boolean/Phrase	245
S129	(MH "User-Computer Interface")	Expanders - Apply equivalent subjects Search modes - Boolean/Phrase	11,017
S130	TI (computer interface* or computer user interface*) OR AB (computer interface* or computer user interface*)	Expanders - Apply equivalent subjects Search modes - Boolean/Phrase	752
S131	(MH "Social Media") OR (MH "Facebook") OR (MH "Twitter")	Expanders - Apply equivalent subjects Search modes - Boolean/Phrase	19,227
S132	(MH "Online Social Networking")	Expanders - Apply equivalent subjects Search modes - Boolean/Phrase	488
S133	TI (social media or social medium or Facebook or Flickr or Instagram or LinkedIn or MySpace or Pinterest or Reddit or "Sina Weibo" or Snapchat or online social network* or social networking platform* or social networking site* or social networking website* or social platform* or TikTok or Tumblr or Twitter or "web 2.0" or "web 2.0s" or WeChat or WhatsApp or YouTube)	Expanders - Apply equivalent subjects Search modes - Boolean/Phrase	12,598
S134	AB (social media or social medium or Facebook or Flickr or Instagram or LinkedIn or MySpace or Pinterest or Reddit or "Sina Weibo" or Snapchat or online social network* or social networking platform* or social networking site* or social networking website* or social platform* or TikTok or	Expanders - Apply equivalent subjects Search modes - Boolean/Phrase	17,263

	Tumblr or Twitter or "web 2.0" or "web 2.0s" or WeChat or WhatsApp or YouTube)		
S135	(MH "Virtual Reality")	Expanders - Apply equivalent subjects Search modes - Boolean/Phrase	6,211
S136	TI (virtual reality or virtual realities) OR AB (virtual reality or virtual realities)	Expanders - Apply equivalent subjects Search modes - Boolean/Phrase	5,025
S137	(MH "Augmented Reality")	Expanders - Apply equivalent subjects Search modes - Boolean/Phrase	287
S138	TI (augmented realities or augmented reality or mixed realities or mixed reality) OR AB (augmented realities or augmented reality or mixed realities or mixed reality)	Expanders - Apply equivalent subjects Search modes - Boolean/Phrase	970
S139	(MH "Holography")	Expanders - Apply equivalent subjects Search modes - Boolean/Phrase	100
S140	TI (hologram* or holograph*) OR AB (hologram* or holograph*)	Expanders - Apply equivalent subjects Search modes - Boolean/Phrase	216
S141	(MH "Printing, Three-Dimensional")	Expanders - Apply equivalent subjects Search modes - Boolean/Phrase	2,182
S142	TI ("3d printing*" or "3 d printing*" or "3 dimensional printing*" or "three dimensional printing*") OR AB ("3d printing*" or "3 d printing*" or "3 dimensional printing*" or "three dimensional printing*")	Expanders - Apply equivalent subjects Search modes - Boolean/Phrase	1,273
S143	TI (chatbot or chatbots or "chat bot" or "chat bots") OR AB (chatbot or chatbots or "chat bot" or "chat bots")	Expanders - Apply equivalent subjects Search modes - Boolean/Phrase	243
S144	TI "virtual care" OR AB "virtual care"	Expanders - Apply equivalent subjects Search modes - Boolean/Phrase	343
S145	TI (closed loop medicines system* or closed loop medication system* or closed loop medicines process* or closed loop medication process*) OR AB (closed loop medicines system* or closed loop medication system* or closed loop	Expanders - Apply equivalent subjects Search modes - Boolean/Phrase	1

	medicines process* or closed loop medication process*)		
S146	TI (bedside station or bedside stations or bed side station or bed side stations or bedside terminal or bedside terminals or bed side terminal or bed side terminals) OR AB (bedside station or bedside stations or bed side station or bed side stations or bedside terminal or bedside terminals or bed side terminal or bed side terminals)	Expanders - Apply equivalent subjects Search modes - Boolean/Phrase	29
S147	TI (predictive analytic OR predictive analytics) OR AB (predictive analytic OR predictive analytics)	Expanders - Apply equivalent subjects Search modes - Boolean/Phrase	369
S148	S27 OR S28 OR S29 OR S30 OR S31 OR S32 OR S33 OR S34 OR S35 OR S36 OR S37 OR S38 OR S39 OR S40 OR S41 OR S42 OR S43 OR S44 OR S45 OR S46 OR S47 OR S48 OR S49 OR S50 OR S51 OR S52 OR S53 OR S54 OR S55 OR S56 OR S57 OR S58 OR S59 OR S60 OR S61 OR S62 OR S63 OR S64 OR S65 OR S66 OR S67 OR S68 OR S69 OR S70 OR S71 OR S72 OR S73 OR S74 OR S75 OR S76 OR S77 OR S78 OR S79 OR S80 OR S81 OR S82 OR S83 OR S84 OR S85 OR S86 OR S87 OR S88 OR S89 OR S90 OR S91 OR S92 OR S93 OR S94 OR S95 OR S96 OR S97 OR S98 OR S99 OR S100 OR S101 OR S102 OR S103 OR S104 OR S105 OR S106 OR S107 OR S108 OR S109 OR S110 OR S111 OR S112 OR S113 OR S114 OR S115 OR S116 OR S117 OR S118 OR S119 OR S120 OR S121 OR S122 OR S123 OR S124 OR S125 OR S126 OR S127 OR S128 OR S129 OR S130 OR S131 OR S132 OR S133 OR S134 OR S135 OR S136 OR S137 OR S138 OR S139 OR S140 OR S141 OR S142 OR S143 OR S144 OR S145 OR S146 OR S147	Expanders - Apply equivalent subjects Search modes - Boolean/Phrase	322,837
S149	S26 AND S148	Expanders - Apply equivalent subjects Search modes - Boolean/Phrase	4,904

S150	S26 AND S148	Limiters - English Language<br>Expanders - Apply equivalent subjects<br>Search modes - Boolean/Phrase	4,657
S151	S26 AND S148	Limiters - Published Date: 20170101-20221231; English Language<br>Expanders - Apply equivalent subjects<br>Search modes - Boolean/Phrase	1,637
S152	S26 AND S148	Limiters - Published Date: 20170101-20221231; English Language; Peer Reviewed<br>Expanders - Apply equivalent subjects<br>Search modes - Boolean/Phrase	1,526

Database: IEEE Explore
Search strategy:

Group 1 Terms Strategy

("All Metadata":telemedicine OR "All Metadata":telecardiology OR "All Metadata":teledermatology OR "All Metadata":telediagnosis OR "All Metadata":telediagnoses OR "All Metadata":telemonitoring OR "All Metadata":telenephrology OR "All Metadata":teleneurology OR "All Metadata":telepsychology OR "All Metadata":teleradiology OR "All Metadata":teleradiotherapy OR "All Metadata":telesurgery OR "All Metadata":teletherapy teletherapies OR "All Metadata":videoconsult OR "All Metadata":videoconsults OR "All Metadata":"tele medicine" OR "All Metadata":"tele cardiology" OR "All Metadata":"tele dermatology" OR "All Metadata":"tele diagnosis" OR "All Metadata":"tele diagnoses" OR "All Metadata":"tele monitoring" OR "All Metadata":"tele nephrology" OR "All Metadata":"tele neurology" OR "All Metadata":"tele psychology" OR "All Metadata":"tele radiology" OR "All Metadata":"tele radiotherapy" OR "All Metadata":"tele surgery" OR "All Metadata":"tele therapy" OR "All Metadata":telehealth OR "All Metadata":"tele health" OR "All Metadata":"e-health" OR "All Metadata":ehealth OR "All Metadata":telenurse OR "All Metadata":"tele nurse" OR "All Metadata":telenursing OR "All Metadata":"tele nursing" OR "All Metadata":"e−rehabilitation" OR "All Metadata":remote rehabilitation OR "All Metadata":"tele rehabilitation" OR "All Metadata":"virtual rehabilitation") OR ("All Metadata":"cyber consult" OR "All Metadata":"cyber consulting" OR "All Metadata":"cyber consultation" OR "All Metadata":cyberconsult OR "All Metadata":econsult OR "All Metadata":cyberconsultation OR "All Metadata":econsultation OR "All Metadata":"e consult" OR "All Metadata":"e consulting" OR "All Metadata":"e consutlation" OR "All Metadata":"email based consult" OR "All Metadata":"email based consulting" OR "All Metadata":"email based consultation" OR "All Metadata":"online consult" OR "All Metadata":"online consultation" OR "All Metadata":"tele consult"

OR "All Metadata":"tele consultation" OR "All Metadata":"telephone based consult" OR "All Metadata":"telephone based consultation" OR "All Metadata":"telephone consult" OR "All Metadata":"telephone consultation" OR "All Metadata":"video consult" OR "All Metadata":"video consultation" OR "All Metadata":"virtual consult" OR "All Metadata":"virtual consultation" OR "All Metadata":"cyber consults" OR "All Metadata":"cyber consultations" OR "All Metadata":cyberconsults OR "All Metadata":econsults OR "All Metadata":cyberconsultations OR "All Metadata":econsultations OR "All Metadata":"e consults" OR "All Metadata":"e consutlations" OR "All Metadata":"email based consults" OR "All Metadata":"email based consultations" OR "All Metadata":"online consults" OR "All Metadata":"online consultations" OR "All Metadata":"tele consults" OR "All Metadata":"tele consultations" OR "All Metadata":"telephone based consults" OR "All Metadata":"telephone based consultations" OR "All Metadata":"telephone consults" OR "All Metadata":"telephone consultations" OR "All Metadata":"video consults" OR "All Metadata":"video consultations" OR "All Metadata":"virtual consults" OR "All Metadata":"virtual consultations" OR "All Metadata":"video conference" OR "All Metadata":"video conferences" OR "All Metadata":video conferencing OR "All Metadata":videoconference OR "All Metadata":videoconferences OR "All Metadata":webcast OR "All Metadata":webcasts OR "All Metadata":webcasting OR "All Metadata":"virtual care") AND ("All Metadata":computer* ONEAR/2 instruction OR "All Metadata":computer user training OR "All Metadata": interactive tutorial OR "All Metadata":webcast OR "All Metadata":web cast OR "All Metadata":webcasting OR "All Metadata":web casting OR "All Metadata":interactive learning OR "All Metadata":simulation) OR ("All Metadata":inservice OR "All Metadata":"in service" OR "All Metadata":"on the job training" OR "All Metadata":"training on the job" OR "All Metadata":"hands on") OR ("All Metadata":practical ONEAR/3 application OR "All Metadata":practical ONEAR/3 component OR "All Metadata":practical ONEAR/3 element OR "All Metadata":practical ONEAR/3 professional development OR "All Metadata":practical ONEAR/3 session) OR ("All Metadata":continuing education OR "All Metadata":continuing nursing education OR "All Metadata":post basic nursing education OR "All Metadata":post registration nursing education)

Search Within Results

nurse OR nurses OR nursing

Filters Applied:

Journals

2017 - 2022

Group 2 Terms Strategy

("All Metadata":"medical record" OR "All Metadata":"health record" OR "All Metadata":"patient record" OR "All Metadata":ehr OR "All Metadata":emr OR "All Metadata":"medical records" OR "All Metadata":"health records" OR "All Metadata":"patient records" OR "All Metadata":ehrs OR "All

Metadata":emrs OR "All Metadata":"health information" OR "All Metadata":"smart card" OR "All Metadata":"smart cards" OR "All Metadata":"order entry" OR "All Metadata":"information system" OR "All Metadata":"information systems" OR "All Metadata":"information management system" OR "All Metadata":"information management systems" OR "All Metadata":"database management" OR "All Metadata":"decision support" OR "All Metadata":"point of care" OR "All Metadata":bedside OR "All Metadata":"bedside" OR "All Metadata":"electronic prescribing" OR "All Metadata":"e prescribing" OR "All Metadata":"e prescription" OR "All Metadata":"e prescriptions" OR "All Metadata":"electronic prescription" OR "All Metadata":"electronic prescriptions" OR "All Metadata":"closed loop medicines system" OR "All Metadata":"closed loop medicines systems" OR "All Metadata":"closed loop medication system" OR "All Metadata":"closed loop medication systems" OR "All Metadata":"closed loop medicines process" OR "All Metadata":"closed loop medication process") AND ("All Metadata":computer* ONEAR/2 instruction OR "All Metadata":computer user training OR "All Metadata": interactive tutorial OR "All Metadata":webcast OR "All Metadata":web cast OR "All Metadata":webcasting OR "All Metadata":web casting OR "All Metadata":interactive learning OR "All Metadata":simulation) OR ("All Metadata":inservice OR "All Metadata":"in service" OR "All Metadata":"on the job training" OR "All Metadata":"training on the job" OR "All Metadata":"hands on") OR ("All Metadata":practical ONEAR/3 application OR "All Metadata":practical ONEAR/3 component OR "All Metadata":practical ONEAR/3 element OR "All Metadata":practical ONEAR/3 professional development OR "All Metadata":practical ONEAR/3 session) OR ("All Metadata":continuing education OR "All Metadata":continuing nursing education OR "All Metadata":post basic nursing education OR "All Metadata":post registration nursing education)

Search Within Results

nurse OR nurses OR nursing

Filters Applied:

Journals

2017 - 2022

Group 3 Terms Strategy

("All Metadata":"mobile app" OR "All Metadata":"mobile apps" OR "All Metadata":"mobile application" OR "All Metadata":"health app" OR "All Metadata":"health apps" OR "All Metadata":"health application" OR "All Metadata":"health applications" OR "All Metadata":"portable software app" OR "All Metadata":"portable software apps" OR "All Metadata":"portable software application" OR "All Metadata":"portable software applications" OR "All Metadata":"tablet app" OR "All Metadata":"tablet apps" OR "All Metadata":"tablet application" OR "All Metadata":"tablet applications" OR "All Metadata":"cell phone" OR "All Metadata":"cell phones" OR "All Metadata":"cellphone" OR "All Metadata":"cellphones" OR "All Metadata":"cellular phone" OR "All Metadata":"cellular phones" OR "All

Metadata":"cellular telephone" OR "All Metadata":"cellular telephones" OR "All Metadata":"mobile phone" OR "All Metadata":"mobile phones" OR "All Metadata":"mobile telephone" OR "All Metadata":"mobile telephones" OR "All Metadata":smartphone OR "All Metadata":smartphones OR "All Metadata":"smart phones" OR "All Metadata":"smart phones" OR "All Metadata":"text messaging" OR "All Metadata":texting) OR ("All Metadata":"social media" OR "All Metadata":"social medium" OR "All Metadata":Facebook OR "All Metadata":Flickr OR "All Metadata":Instagram OR "All Metadata":LinkedIn OR "All Metadata":MySpace OR "All Metadata":Pinterest OR "All Metadata":Reddit OR "All Metadata":"Sina Weibo" OR "All Metadata":Snapchat OR "All Metadata":"online social network" OR "All Metadata":"social networking platform" OR "All Metadata":"social networking site" OR "All Metadata":"social networking website" OR "All Metadata":"social platform" OR "All Metadata":TikTok OR "All Metadata":Tumblr OR "All Metadata":Twitter OR "All Metadata":"web 2.0" OR "All Metadata":"web 2.0s" OR "All Metadata":WeChat OR "All Metadata":WhatsApp OR "All Metadata":YouTube OR "All Metadata":chatbot OR "All Metadata":chatbots OR "All Metadata":"chat bot" OR "All Metadata":"chat bots" OR "All Metadata":"smart technology" OR "All Metadata":"smart technologies" OR "All Metadata":wearable OR "All Metadata":wearables OR "All Metadata":"activity tracker" OR "All Metadata":"activity trackers" OR "All Metadata":"fitness tracker" OR "All Metadata":"fitness trackers" OR "All Metadata":smartwatch OR "All Metadata":"smart watch" OR "All Metadata":"smart watches" OR "All Metadata":pedometer OR "All Metadata":pedometers OR "All Metadata":smartglasses OR "All Metadata":"smart glasses" OR "All Metadata":"virtual reality" OR "All Metadata":"virtual realities" OR "All Metadata":"augmented realities" OR "All Metadata":"augmented reality" OR "All Metadata":"mixed realities" OR "All Metadata":"mixed reality" OR "All Metadata":hologram OR "All Metadata":holograms OR "All Metadata":holograph OR "All Metadata":holographs OR "All Metadata":"3d printing" OR "All Metadata":"3 d printing*" OR "All Metadata":"3 dimensional printing" OR "All Metadata":"three dimensional printing") OR ("All Metadata":wireless OR "All Metadata":internet OR "All Metadata":web OR "All Metadata":webbased OR "All Metadata":"web based") AND ("All Metadata":computer* ONEAR/2 instruction OR "All Metadata":computer user training OR "All Metadata": interactive tutorial OR "All Metadata":webcast OR "All Metadata":web cast OR "All Metadata":webcasting OR "All Metadata":web casting OR "All Metadata":interactive learning OR "All Metadata":simulation) OR ("All Metadata":inservice OR "All Metadata":"in service" OR "All Metadata":"on the job training" OR "All Metadata":"training on the job" OR "All Metadata":"hands on") OR ("All Metadata":practical ONEAR/3 application OR "All Metadata":practical ONEAR/3 component OR "All Metadata":practical ONEAR/3 element OR "All Metadata":practical ONEAR/3 professional development OR "All Metadata":practical ONEAR/3 session) OR ("All Metadata":continuing education OR "All Metadata":continuing nursing education OR "All Metadata":post basic nursing education OR "All Metadata":post registration nursing education)

Search Within Results

nurse OR nurses OR nursing

Filters Applied:

Journals

2017 - 2022

Group 4 Terms Strategy

("All Metadata":"artificial intelligence" OR "All Metadata":"machine intelligence" OR "All Metadata":"expert system" OR "All Metadata":"expert systems" OR "All Metadata":"knowledge based system" OR "All Metadata":"knowledge based systems" OR "All Metadata":"fuzzy logic" OR "All Metadata":"fuzzy model" OR "All Metadata":"fuzzy models" OR "All Metadata":"machine learning" OR "All Metadata":"learning machine" OR "All Metadata":"learning machines" OR "All Metadata":"deep learning" OR "All Metadata":"hierarchical learning" OR "All Metadata":"ann approach" OR "All Metadata":"ann model" OR "All Metadata":"ann method" OR "All Metadata":"ann training" OR "All Metadata":"neural network" OR "All Metadata":"neural networks" OR "All Metadata":robotics OR "All Metadata":nanorobotics OR "All Metadata":robot OR "All Metadata":robots OR "All Metadata":"biomedical technology" OR "All Metadata":"bio medical technology" OR "All Metadata":"biomedical technologies" OR "All Metadata":"bio medical technologies") OR ("All Metadata":informatics OR "All Metadata":"clinical information science" OR "All Metadata":"clinical information technology" OR "All Metadata":"clinical information technologies" OR "All Metadata":"health information science" OR "All Metadata":"health information technology" OR "All Metadata":"health information technologies" OR "All Metadata":"medical computer science" OR "All Metadata":"medical information science" OR "All Metadata":"medical information technology" OR "All Metadata":"medical information technology" OR "All Metadata":"automatic diagnosis" OR "All Metadata":"computer assisted diagnosis" OR "All Metadata":"computer diagnosis" OR "All Metadata":"automatic diagnoses" OR "All Metadata":"computer assisted diagnoses" OR "All Metadata":"computer diagnoses" OR "All Metadata":"information system" OR "All Metadata":"information systems" OR "All Metadata":"online system" OR "All Metadata":"online systems" OR "All Metadata":"computer interface" OR "All Metadata":"computer interefaces" OR "All Metadata":"computer user interface" OR "All Metadata":"computer user interfaces") OR ("All Metadata":"predictive analytics") AND ("All Metadata":computer* ONEAR/2 instruction OR "All Metadata":computer user training OR "All Metadata": interactive tutorial OR "All Metadata":webcast OR "All Metadata":web cast OR "All Metadata":webcasting OR "All Metadata":web casting OR "All Metadata":interactive learning OR "All Metadata":simulation) OR ("All Metadata":inservice OR "All Metadata":"in service" OR "All Metadata":"on the job training" OR "All Metadata":"training on the job" OR "All Metadata":"hands on") OR ("All Metadata":practical ONEAR/3 application OR "All Metadata":practical ONEAR/3 component OR "All Metadata":practical ONEAR/3 element OR "All Metadata":practical ONEAR/3 professional development OR "All Metadata":practical ONEAR/3 session) OR ("All Metadata":continuing education OR "All Metadata":continuing nursing education OR "All Metadata":post basic nursing education OR "All Metadata":post registration nursing education)

Search Within Results

nurse OR nurses OR nursing

Filters Applied:

Journals

2017 - 2022

Indirect Search for Recommendation Question #1: Should practical (e.g., hands-on) professional development education focused on the use of digital health technologies within an organization be recommended or not for all nurses?

Dates searched: January 1, 2017 – January 27, 2023

Databases searched: MEDLINE, Embase, and CINAHL

**Note: The original search strategy was broadened to examine practical or hands-on professional development education in general (not specific to digital health technologies), and all health providers (not just nurses). The search was only applied to MEDLINE, Embase, and CINAHL for feasibility purposes, and limited to systematic reviews in order to obtain the highest quality evidence.*

Database: Medline
Search strategy:

#	Searches	Results
1	exp "Attitude of Health Personnel"/	168882
2	exp Health Personnel/	601015
3	exp Personnel, Hospital/	94935
4	exp Health Occupations/	1836824
5	exp Patient Care Team/	72541
6	Social Work/	16205
7	Social Workers/	1060
8	Nurse's Role/	42671
9	Licensed Practical Nurses/	97
10	exp Nurses/	97093
11	exp Nursing Staff/	69534
12	Nursing Staff, Hospital/	47729
13	exp Nursing/	262497
14	Nursing, Practical/	3444
15	Health Educators/	491
16	exp Administrative Personnel/	42155

17	(health* adj2 (provider? or staff? or personnel? or employe* or profession* or occupation? or practitioner? or worker?)).tw,kf.	315245
18	(hospital* adj2 (provider? or staff? or personnel? or employe* or profession* or occupation? or practitioner? or worker?)).tw,kf.	21151
19	nurs*.tw,kf.	523533
20	(patient care? adj2 team?).tw,kf.	1023
21	(health* adj2 educator?).tw,kf.	3786
22	(social adj2 worker?).tw,kf.	12571
23	(support* adj2 (worker? or staff?)).tw,kf.	8093
24	clinician?.tw,kf.	287978
25	doctor?.tw,kf.	142491
26	physician?.tw,kf.	450553
27	practitioner?.tw,kf.	176036
28	surgeon?.tw,kf.	242999
29	Caregivers/	48514
30	(caregiver? or care-giver? or carer or carers).tw,kf.	105611
31	or/1-30	3628402
32	Computer User Training/	2042
33	computer user training*.tw,kf.	12
34	Computer Assisted Instruction/	12495
35	(computer assisted instruction* or computerised programmed instruction* or computerised self instruction program* or computerized programmed instruction* or computerized self instruction program*).tw,kf.	722
36	interactive tutorial/ or webcast/	1391
37	(interactive tutorial or interactive tutorials or webcast* or "web cast*").tw,kf.	392
38	InService Training/	20734
39	(inservice or "in service" or "on the job training" or "training on the job").tw,kf.	11621
40	Simulation Training/ or High Fidelity Simulation Training/ or Patient Simulation/	11424
41	(interactive learning or simulation or simulat*).tw,kf.	671745
42	"hands on".tw,kf.	8953
43	(practical adj3 (application or component* or element* or professional development or session*)).tw,kf.	21828
44	exp education, continuing/ or education, nursing, continuing/	62573
45	(continuing education or continuing medical education or continuing pharma* education or continuing nursing education or "post basic nursing education" or "post registration nursing education").tw,kf.	20542
46	or/32-45 [Practical or hands-on professional development education]	809398
47	31 and 46	129622
48	31 and 47	129622
49	limit 47 to english language	117072
50	limit 49 to yr="2017 -Current"	31836

51	review/	3113503
52	(medline or medlars or pubmed or grateful med or CINAHL or scisearch or psychinfo or psycinfo or psychlit or psyclit or handsearch* or hand search* or manual* search* or electronic database* or bibliographic database* or embase or lilacs or scopus or "web of science").mp.	368475
53	51 and 52	195172
54	meta-analysis.mp.	266344
55	meta-analysis as topic/	22091
56	meta-analysis/	174676
57	systematic review*.tw,kf.	276564
58	cochrane database*.jn.	16118
59	or/53-58	504298
60	50 and 59	1561

Database: Embase
Search strategy:

#	Searches	Results
1	exp health personnel attitude/	203349
2	exp health care personnel/	1979662
3	exp hospital personnel/	152475
4	exp multidisciplinary team/	23939
5	social work/	30297
6	social worker/	16361
7	licensed practical nurse/	377
8	exp nurse/	219255
9	nursing staff/	77532
10	exp nursing/	416453
11	practical nursing/	166
12	exp health educator/	3426
13	exp administrative personnel/	35058
14	nurse attitude/	43186
15	(health* adj2 (provider? or staff? or personnel? or employe* or profession* or occupation? or practitioner? or worker?)).tw,kw.	403574
16	(hospital* adj2 (provider? or staff? or personnel? or employe* or profession* or occupation? or practitioner? or worker?)).tw,kw.	29216
17	nurs*.tw,kw.	628964
18	(patient care? adj2 team?).tw,kw.	870
19	(health* adj2 educator?).tw,kw.	4354
20	(social adj2 worker?).tw,kw.	21269
21	(support* adj2 (worker? or staff?)).tw,kw.	11412

22	clinician?.tw,kw.	414400
23	doctor?.tw,kw.	212522
24	physician?.tw,kw.	660440
25	practitioner?.tw,kw.	227115
26	surgeon?.tw,kw.	384676
27	caregiver/	107260
28	(caregiver? or care-giver? or carer or carers).tw,kw.	148739
29	or/1-28	3784692
30	human computer interaction/	6641
31	computer user training*.tw,kw.	21
32	(computer assisted instruction* or computerised programmed instruction* or computerised self instruction program* or computerized programmed instruction* or computerized self instruction program*).tw,kw.	977
33	webcast/	436
34	(interactive tutorial or interactive tutorials or webcast* or "web cast*").tw,kf.	653
35	"in service training"/	16787
36	(inservice or "in service" or "on the job training" or "training on the job").tw,kw.	14816
37	simulation/ or computer simulation/ or digital twin/ or discrete event simulation/ or high fidelity simulation/ or high-fidelity patient simulation/ or patient simulation/	375219
38	(interactive learning or simulation or simulat*).tw,kw.	723710
39	"hands on".tw,kw.	14785
40	(practical adj3 (application or component* or element* or professional development or session*)).tw,kw.	24724
41	continuing education/	33463
42	(continuing education or continuing medical education or continuing pharma* education or continuing nursing education or "post basic nursing education" or "post registration nursing education").tw,kw.	27011
43	or/30-42 [Practical or hands-on professional development education]	924760
44	29 and 43	121528
45	limit 44 to english language	114762
46	limit 45 to yr="2017 -Current"	42424
47	limit 46 to (conference abstract or conference paper or "conference review")	13897
48	46 not 47	28527
49	"review"/	2885312
50	(medline or medlars or pubmed or grateful med or CINAHL or scisearch or psychinfo or psycinfo or psychlit or psyclit or handsearch* or hand search* or manual* search* or electronic database* or bibliographic database* or embase or lilacs or scopus or web of science).mp.	468382
51	49 and 50	186635
52	meta-analysis.mp.	395370
53	"meta analysis (topic)"/	50823

54	meta analysis/	275503
55	systematic review*.tw,kw.	339660
56	cochrane database*.jn.	22827
57	or/51-56	649595
58	48 and 57	1103

Database: Cumulative Index to Nursing and Allied Health (CINAHL)
Search strategy:

#	Query	Limiters/Expanders	Results
S1	(MH "Attitude of Health Personnel+")	Expanders - Apply equivalent subjects Search modes - Boolean/Phrase	122,420
S2	(MH "Health Personnel+")	Expanders - Apply equivalent subjects Search modes - Boolean/Phrase	638,997
S3	(MH "Personnel, Health Facility+")	Expanders - Apply equivalent subjects Search modes - Boolean/Phrase	44,740
S4	(MH "Health Occupations+")	Expanders - Apply equivalent subjects Search modes - Boolean/Phrase	826,191
S5	(MH "Multidisciplinary Care Team+")	Expanders - Apply equivalent subjects Search modes - Boolean/Phrase	51,779
S6	(MH "Social Work+") or (MH "Social Workers")	Expanders - Apply equivalent subjects Search modes - Boolean/Phrase	24,550
S7	(MH "Nursing Role")	Expanders - Apply equivalent subjects Search modes - Boolean/Phrase	65,578
S8	(MH "Practical Nurses") OR (MH "Practical Nursing")	Expanders - Apply equivalent subjects Search modes - Boolean/Phrase	7,721
S9	(MH "Nurses+")	Expanders - Apply equivalent subjects Search modes - Boolean/Phrase	242,183
S10	(MH "Nursing Staff, Hospital")	Expanders - Apply equivalent subjects Search modes - Boolean/Phrase	25,051
S11	(MH "Health Educators+")	Expanders - Apply equivalent subjects Search modes - Boolean/Phrase	4,214
S12	(MH "Administrative Personnel+")	Expanders - Apply equivalent subjects Search modes - Boolean/Phrase	40,701
S13	TI (health* N2 provider#) OR AB (health* N2 provider#)	Expanders - Apply equivalent subjects Search modes - Boolean/Phrase	57,617
S14	TI (hospital* N2 provider#) OR AB (hospital* N2 provider#)	Expanders - Apply equivalent subjects Search modes - Boolean/Phrase	1,828

S15	TI (health* N2 staff#) OR AB (health* N2 staff#)	Expanders - Apply equivalent subjects Search modes - Boolean/Phrase	8,953
S16	TI (hospital* N2 staff#) OR AB (hospital* N2 staff#)	Expanders - Apply equivalent subjects Search modes - Boolean/Phrase	6,060
S17	TI (health* N2 personnel#) OR AB (health* N2 personnel#)	Expanders - Apply equivalent subjects Search modes - Boolean/Phrase	5,436
S18	TI (hospital* N2 personnel#) OR AB (hospital* N2 personnel#)	Expanders - Apply equivalent subjects Search modes - Boolean/Phrase	1,154
S19	TI (health* N2 employe*) OR AB (health* N2 employe*)	Expanders - Apply equivalent subjects Search modes - Boolean/Phrase	7,120
S20	TI (hospital* N2 employe*) OR AB (hospital* N2 employe*)	Expanders - Apply equivalent subjects Search modes - Boolean/Phrase	2,071
S21	TI (health* N2 profession*) OR AB (health* N2 profession*)	Expanders - Apply equivalent subjects Search modes - Boolean/Phrase	100,794
S22	TI (hospital* N2 profession*) OR AB (hospital* N2 profession*)	Expanders - Apply equivalent subjects Search modes - Boolean/Phrase	1,988
S23	TI (health* N2 occupation#) OR AB (health* N2 occupation#)	Expanders - Apply equivalent subjects Search modes - Boolean/Phrase	1,015
S24	TI (hospital* N2 occupation#) OR AB (hospital* N2 occupation#)	Expanders - Apply equivalent subjects Search modes - Boolean/Phrase	58
S25	TI (health* N2 practitioner#) OR AB (health* N2 practitioner#)	Expanders - Apply equivalent subjects Search modes - Boolean/Phrase	12,181
S26	TI (hospital* N2 practitioner#) OR AB (hospital* N2 practitioner#)	Expanders - Apply equivalent subjects Search modes - Boolean/Phrase	927
S27	TI (health* N2 worker#) OR AB (health* N2 worker#)	Expanders - Apply equivalent subjects Search modes - Boolean/Phrase	34,064
S28	TI (hospital* N2 worker#) OR AB (hospital* N2 worker#)	Expanders - Apply equivalent subjects Search modes - Boolean/Phrase	1,596
S29	TI nurs* OR AB nurs*	Expanders - Apply equivalent subjects Search modes - Boolean/Phrase	641,168
S30	TI (patient care# N2 team#) OR AB (patient care# N2 team#)	Expanders - Apply equivalent subjects Search modes - Boolean/Phrase	589
S31	TI (health* N2 educator#) OR AB (health* N2 educator#)	Expanders - Apply equivalent subjects Search modes - Boolean/Phrase	3,763
S32	TI (social N2 worker#) OR AB (social N2 worker#)	Expanders - Apply equivalent subjects Search modes - Boolean/Phrase	14,825
S33	TI (support* N2 (worker# or staff#)) OR AB (support* N2 (worker# or staff#))	Expanders - Apply equivalent subjects Search modes - Boolean/Phrase	9,638

S34	TI clinician# OR AB clinician#	Expanders - Apply equivalent subjects Search modes - Boolean/Phrase	123,182
S35	TI doctor# OR AB doctor#	Expanders - Apply equivalent subjects Search modes - Boolean/Phrase	65,364
S36	TI physician# OR AB physician#	Expanders - Apply equivalent subjects Search modes - Boolean/Phrase	169,372
S37	TI practitioner# OR AB practitioner#	Expanders - Apply equivalent subjects Search modes - Boolean/Phrase	107,872
S38	TI surgeon# OR AB surgeon#	Expanders - Apply equivalent subjects Search modes - Boolean/Phrase	63,392
S39	(MH "Caregivers")	Expanders - Apply equivalent subjects Search modes - Boolean/Phrase	42,899
S40	TI (caregiver# or care-giver# or carer or carers) OR AB (caregiver# or care-giver# or carer or carers)	Expanders - Apply equivalent subjects Search modes - Boolean/Phrase	79,608
S41	S1 OR S2 OR S3 OR S4 OR S5 OR S6 OR S7 OR S8 OR S9 OR S10 OR S11 OR S12 OR S13 OR S14 OR S15 OR S16 OR S17 OR S18 OR S19 OR S20 OR S21 OR S22 OR S23 OR S24 OR S25 OR S26 OR S27 OR S28 OR S29 OR S30 OR S31 OR S32 OR S33 OR S34 OR S35 OR S36 OR S37 OR S38 OR S39 OR S40	Expanders - Apply equivalent subjects Search modes - Boolean/Phrase	2,195,709
S42	(MH "Computer User Training")	Expanders - Apply equivalent subjects Search modes - Boolean/Phrase	798
S43	TI (computer user training*) OR AB (computer user training*)	Expanders - Apply equivalent subjects Search modes - Boolean/Phrase	0
S44	TI (computer user training*) OR AB (computer user training*)	Expanders - Apply equivalent subjects Search modes - SmartText Searching	394
S45	(MH "Computer Assisted Instruction")	Expanders - Apply equivalent subjects Search modes - Boolean/Phrase	8,293
S46	TI (computer assisted instruction* or computerized programmed instruction* or computerized self instruction program*) OR AB (computer assisted instruction* or computerized programmed instruction* or computerized self instruction program*)	Expanders - Apply equivalent subjects Search modes - Boolean/Phrase	372
S47	(MH "Webcasts")	Expanders - Apply equivalent subjects Search modes - Boolean/Phrase	901
S48	TI (interactive tutorial or interactive tutorials or webcast* or "web cast*") OR AB	Expanders - Apply equivalent subjects Search modes - Boolean/Phrase	314

	(interactive tutorial or interactive tutorials or webcast* or "web cast*")		
S49	(MH "Staff Development")	Expanders - Apply equivalent subjects Search modes - Boolean/Phrase	29,792
S50	TI (inservice or "in service" or "on the job training" or "training on the job") OR AB (inservice or "in service" or "on the job training" or "training on the job")	Expanders - Apply equivalent subjects Search modes - Boolean/Phrase	184,398
S51	(MH "Patient Simulation") OR (MH "Simulations") OR (MH "Vignettes")	Expanders - Apply equivalent subjects Search modes - Boolean/Phrase	28,534
S52	TI (interactive learning or simulation* or simulat*) OR AB (interactive learning or simulation* or simulat*)	Expanders - Apply equivalent subjects Search modes - Boolean/Phrase	68,542
S53	TI ("hands on") OR AB ("hands on")	Expanders - Apply equivalent subjects Search modes - Boolean/Phrase	20,277
S54	TI (practical N3 (application or component* or element* or professional development or session*)) OR AB (practical N3 (application or component* or element* or professional development or session*))	Expanders - Apply equivalent subjects Search modes - Boolean/Phrase	4,297
S55	(MH "Education, Continuing") OR (MH "Education, Medical, Continuing") OR (MH "Education, Nursing, Continuing") OR (MH "Education, Continuing (Credit)")	Expanders - Apply equivalent subjects Search modes - Boolean/Phrase	141,067
S56	TI (continuing education or continuing nursing education or "post basic nursing education" or "post registration nursing education") OR AB (continuing education or continuing nursing education or "post basic nursing education" or "post registration nursing education")	Expanders - Apply equivalent subjects Search modes - Boolean/Phrase	15,586
S57	S42 OR S43 OR S44 OR S45 OR S46 OR S47 OR S48 OR S49 OR S50 OR S51 OR S52 OR S53 OR S54 OR S55 OR S56	Expanders - Apply equivalent subjects Search modes - Boolean/Phrase	458,827
S58	S41 AND S57	Expanders - Apply equivalent subjects Search modes - Boolean/Phrase	216,475
S59	(MH "Literature Review")	Expanders - Apply equivalent subjects Search modes - Boolean/Phrase	9,005
S60	TI (medline OR medlars OR pubmed OR grateful med OR CINAHL OR scisearch OR psychinfo OR psycinfo OR psychlit OR psyclit OR handsearch* OR "hand search*" OR "manual* search*" OR "electronic database*"	Expanders - Apply equivalent subjects Search modes - Boolean/Phrase	139,075

	OR "bibliographic database*" OR embase OR lilacs OR scopus OR "web of science") OR AB (medline OR medlars OR pubmed OR grateful med OR CINAHL OR scisearch OR psychinfo OR psycinfo OR psychlit OR psyclit OR handsearch* OR "hand search*" OR "manual* search*" OR "electronic database*" OR "bibliographic database*" OR embase OR lilacs OR scopus OR "web of science")		
S61	S59 AND S60	Expanders - Apply equivalent subjects<br>Search modes - Boolean/Phrase	1,148
S62	TI ("meta-analysis") OR AB ("meta-analysis")	Expanders - Apply equivalent subjects<br>Search modes - Boolean/Phrase	90,032
S63	(MH "Meta Analysis")	Expanders - Apply equivalent subjects<br>Search modes - Boolean/Phrase	68,361
S64	TI (systematic review*) OR AB (systematic review*)	Expanders - Apply equivalent subjects<br>Search modes - Boolean/Phrase	136,786
S65	JN cochrane database*	Expanders - Apply equivalent subjects<br>Search modes - Boolean/Phrase	6,023
S66	S61 OR S62 OR S63 OR S64 OR S65	Expanders - Apply equivalent subjects<br>Search modes - Boolean/Phrase	185,587
S67	S58 AND S66	Expanders - Apply equivalent subjects<br>Search modes - Boolean/Phrase	3,343
S68	S58 AND S66	Limiters - English Language<br>Expanders - Apply equivalent subjects<br>Search modes - Boolean/Phrase	3,283
S69	S58 AND S66	Limiters - Published Date: 20170101-20231231; English Language<br>Expanders - Apply equivalent subjects<br>Search modes - Boolean/Phrase	1,642

<u>Recommendation Question #2:</u> Should education about relational care and interpersonal communication skills be recommended or not for nurses practicing in virtual care settings and in-person digital health environments?

Dates searched: January 1, 2017 – February 21, 2023

Databases searched:

- MEDLINE
- MEDLINE Epub Ahead of Print and In-Process
- Embase
- Emcare Nursing

- Cochrane Central Register of Controlled Trials
- Cochrane Database of Systematic Reviews
- APA PsychInfo
- Cumulative Index to Nursing and Allied Health (CINAHL)
- IEEE Explore *(search run July 19, 2022)*

Database: Medline
Search strategy:

#	Searches	Results
1	Computer User Training/	2041
2	computer user training*.tw,kf.	6
3	Computer Assisted Instruction/	12330
4	(computer assisted instruction* or computerised programmed instruction* or computerised self instruction program* or computerized programmed instruction* or computerized self instruction program*).tw,kf.	655
5	interactive tutorial/ or webcast/	1359
6	(interactive tutorial or interactive tutorials or webcast* or "web cast*").tw,kf.	311
7	InService Training/	20692
8	(inservice or "in service" or "on the job training" or "training on the job").tw,kf.	9041
9	Simulation Training/ or High Fidelity Simulation Training/ or Patient Simulation/	10981
10	(interactive learning or simulation or simulat*).tw,kf.	426196
11	education, continuing/ or education, nursing, continuing/	32517
12	(continuing education or continuing nursing education or "post basic nursing education" or "post registration nursing education").tw,kf.	12875
13	exp Health Personnel/ed [Education]	63591
14	or/1-13 [Professional development education]	550534
15	telemedicine/	32401
16	(telemedicine or telecardiology or teledermatology or telediagnosis or telediagnoses or telehealth or telemonitoring or telenephrology or teleneurology or telepsychology or teleradiology or teleradiotherap* or telesurger* or teletherap* or videoconsult*).tw,kf.	24743
17	("tele medicine" or "tele cardiology" or "tele dermatology" or "tele diagnosis" or "tele diagnoses" or "tele monitoring" or "tele nephrology" or "tele neurology" or "tele psychology" or "tele radiology" or "tele radiotherap*" or "tele surger*" or "tele therap*" or "video consult*").tw,kf.	834
18	("e-health" or ehealth or "tele health").tw,kf.	6903
19	(telenurs* or "tele nurs*").tw,kf.	246
20	remote consultation/	5469
21	("cyber consult*" or cyberconsult* or econsult* or "e–consult*" or "email based consult*" or "internet consult*" or "internet based consult*" or "online consult*" or "tele consult*" or "telephone based consult*" or "telephone consult*" or "virtual consult*" or "web consult*" or "webbased consult*" or "web based consult*").tw,kf.	1591

22	telerehabilitation/	722
23	("e−rehabilitation" or remote rehabilitation or tele rehabilitation or virtual rehabilitation).tw,kf.	350
24	medical records systems, computerized/	19133
25	(computerised medical record system* or computerized medical record system*).tw,kf.	94
26	electronic health records/	24476
27	(electronic health record* or computerised patient record* or computerized patient record* or electronic medical record* or electronic patient record* or ehr or emr).tw,kf.	42070
28	health smart cards/	59
29	(smart card or smart cards).tw,kf.	293
30	medical order entry systems/	2400
31	medical order entry system*.tw,kf.	84
32	hospital information systems/	11035
33	(hospital information system* or patient health record information system*).tw,kf.	2651
34	ambulatory care information systems/	1171
35	ambulatory care information system*.tw,kf.	15
36	electronic prescribing/	1134
37	("e−prescribing" or "e−prescription*" or electronic prescription*).tw,kf.	540
38	("c.p.o.e. system" or computer* order entry or computer* physician order entry or computer* provider order entry or computer* order entry or computer* prescriber order entry or computer* prescribing order entry or computer* prescription order entry or "cpoe").tw,kf.	1887
39	operating room information systems/	527
40	(operating room information system* or operating room information management system*).tw,kf.	28
41	Point-of-Care Systems/	15079
42	(bedside computing or bedside technolog* or point of care system* or point of care technolog* or point of care information system*).tw,kf.	714
43	bedside information system*.tw,kf.	10
44	(management information system* or management information).tw,kf.	1882
45	clinical pharmacy information systems/	1199
46	clinical pharmacy information system*.tw,kf.	15
47	database management systems/	7718
48	(database management system* or database management tool or database management tools or database manager system* or database managing system* or data base manager system* or data base management software or data base management system* or data base management tool or data base management tools).tw,kf.	587
49	decision support systems, management/	966
50	decision support system*.tw,kf.	6040
51	Mobile Applications/	9533

52	(mobile app or mobile apps or mobile application* or mobile health app or mobile health apps or mobile health application* or portable software app or portable software apps or portable software application* or tablet app or tablet apps or tablet application*).tw,kf.	5404
53	cell phone/	9492
54	(cell phone or cell phones or cellphone or cellphones or cellular phone or cellular phones or cellular telephone or cellular telephones or mobile phone or mobile phones or mobile telephone or mobile telephones).tw,kf.	11726
55	smartphone/	7369
56	(smartphone or smartphones or smart phone or smart phones).tw,kf.	12905
57	text messaging/	3960
58	(text messaging or texting).tw,kf.	2759
59	videoconferencing/	2190
60	(video conference* or video conferencing or videoconferenc*).tw,kf.	3298
61	webcast/	1080
62	webcasts as topic/	404
63	(webcast* or web cast*).tw,kf.	238
64	Wireless Technology/	4189
65	(wireless communication* or wireless technol*).tw,kf.	1356
66	"Cell Phone Use"/	327
67	("cell phone usage" or "cell phone utilisation" or "cell phone utilization" or "cellphone usage" or "cellphone use" or "cellphone utilisation " or "cellphone utilization" or "cell phone use" or "mobile phone usage" or "mobile phone use" or "mobile phone utilisation" or "mobile phone utilization").tw,kf.	1256
68	computers, handheld/	3937
69	(hand held computer* or handheld computer* or palm PC or palmtop or personal data assistant* or personal digital assistant or pocket computer* or pocket sized computer*).tw,kf.	1356
70	internet/	78395
71	internet.tw,kf.	49850
72	internet-based intervention/	860
73	(internet based intervention* or online based intervention* or online intervention* or web intervention* or web based intervention*).tw,kf.	2428
74	smart technolog*.tw,kf.	253
75	wearable electronic devices/	5551
76	(wearable computer or wearable computers or wearable electronic device*).tw,kf.	261
77	fitness trackers/	977
78	(activity tracker* or fitness tracker* or smart watch or smart watches or pedometer*).tw,kf.	3317
79	smart glasses/	117
80	("ar glasses" or "ar head mounted device" or "ar head mounted display" or "ar head worn display" or "ar headset" or "ar hud" or "arhmd" or "hmd ar" or "optical see	176

	through head mounted display" or "ost hmd" or "see through hmd" or "google glasses" or "smartglasses" or "smart glasses").tw,kf.	
81	(augmented reality glasses or augmented reality head mounted device or augmented reality head mounted display or augmented reality head up display or augmented reality head worn display or head mounted display augmented reality).tw,kf.	37
82	artificial intelligence/	29749
83	(artificial intelligence or machine intelligence).tw,kf.	12485
84	computer heuristics/	51
85	computer heuristic*.tw,kf.	0
86	expert systems/	3466
87	(expert system* or knowledge based system*).tw,kf.	3230
88	fuzzy logic/	4953
89	(fuzzy logic or fuzzy model or fuzzy models).tw,kf.	1739
90	machine learning/	22249
91	(machine learning or learning machine*).tw,kf.	37461
92	deep learning/	9559
93	(deep learning or hierarchical learning).tw,kf.	15920
94	unsupervised machine learning/	638
95	unsupervised machine learning.tw,kf.	485
96	natural language processing/	5297
97	natural language processing.tw,kf.	3845
98	neural networks, computer/	36801
99	("ann approach*" or "ann model" or "ann method*" or "ann training" or artificial neural network or computer neural network or computer neural networks).tw,kf.	6903
100	robotics/	23738
101	(robotics or nanorobotics or robot or robots).tw,kf.	23412
102	biomedical technology/	7070
103	(biomedical technology or bio medical technology).tw,kf.	372
104	informatics/	1122
105	informatics.tw,kf.	14012
106	medical informatics/	12709
107	(clinical informatics or clinical information science or clinical information technology or health informatics or health information science or health information technology or medical computer science or medical information science or medical informatics or medical information technology).tw,kf.	8462
108	medical informatics computing/	761
109	nursing informatics/	1597
110	nursing informatics.tw,kf.	837
111	health information exchange/	1042
112	health information exchange.tw,kf.	982
113	medical informatics applications/	2549

114	medical informatics applications.tw,kf.	109
115	decision making, computer-assisted/	2868
116	(decision support system* or decision support techniques).tw,kf.	6379
117	diagnosis, computer-assisted/	23745
118	(automatic diagnosis or computer assisted diagnosis or computer diagnosis or automatic diagnoses or computer assisted diagnoses or computer diagnoses).tw,kf.	1237
119	image interpretation, computer-assisted/	47510
120	computer assisted image interpretation.tw,kf.	21
121	radiographic image interpretation, computer-assisted/	15567
122	computer assisted radiographic image interpretation*.tw,kf.	16
123	therapy, computer-assisted/	6953
124	computer assisted therap*.tw,kf.	82
125	drug therapy, computer-assisted/	1690
126	computer assisted drug therap*.tw,kf.	16
127	decision support systems, clinical/	8949
128	clinical decision support system*.tw,kf.	2228
129	information systems/	19240
130	(information system or information systems).tw,kf.	32504
131	online systems/	8516
132	(online system or online systems).tw,kf.	544
133	user-computer interface/	38898
134	(computer interface* or computer user interface*).tw,kf.	5133
135	Social Media/	12640
136	(social media or social medium or Facebook or Flickr or Instagram or LinkedIn or MySpace or Pinterest or Reddit or "Sina Weibo" or Snapchat or online social network* or social networking platform* or social networking site* or social networking website* or social platform* or TikTok or Tumblr or Twitter or "web 2.0" or "web 2.0s" or WeChat or WhatsApp or YouTube).tw,kf.	20256
137	Virtual Reality/	4036
138	(virtual reality or virtual realities).tw,kf.	10205
139	Augmented Reality/	686
140	(augmented realities or augmented reality or mixed realities or mixed reality).tw,kf.	2263
141	holography/	2081
142	(hologram* or holograph*).tw,kf.	2786
143	Printing, Three-Dimensional/	8994
144	("3d printing*" or "3 d printing*" or "3 dimensional printing*" or "three dimensional printing*").tw,kf.	7073
145	(chatbot or chatbots or "chat bot" or "chat bots").tw,kf.	221
146	"virtual care".tw,kf.	430
147	(closed loop medicines system* or closed loop medication system* or closed loop medicines process* or closed loop medication process*).tw,kf.	2

148	(bedside station or bedside stations or bed side station or bed side stations or bedside terminal or bedside terminals or bed side terminal or bed side terminals).tw,kf.	55
149	predictive analytic*.tw,kf.	517
150	or/15-149 [digital environment]	616131
151	14 and 150 [Professional development education AND digital environment]	49151
152	interpersonal relations/ or professional-patient relations/ or nurse-patient relations/ or physician-patient relations/	210222
153	(nurse* patient relation* or patient nurs* relation* or professional* patient relation* or patient professional* relation* or doctor patient relation* or patient doctor relation* or physician patient relation* or patient physician relation*).tw,kf.	24802
154	(interpersonal relation* or "inter personal relation*" or social relation*).tw,kf.	15806
155	(interpersonal care or "inter personal care").tw,kf.	144
156	Empathy/	21344
157	(caring or compassion* or empathy or empathetic).tw,kf.	62818
158	rapport.tw,kf.	4003
159	Humanism/	3689
160	humanistic care.tw,kf.	134
161	Patient-Centered Care/	21818
162	(patient centered care or patient centered nursing or patient centred care or patient centred nursing or patient focused care).tw,kf.	7971
163	(interpersonal communication* or "inter personal communication*").tw,kf.	1580
164	Professionalism/	1610
165	professionalism.tw,kf.	7966
166	etiquette.tw,kf.	496
167	(webside manner? or web side manner? or virtual manner?).tw,kf.	20
168	(bedside manner? or bed side manner?).tw,kf.	173
169	Communication/	92851
170	(communication skill? or communication technique?).tw,kf.	11544
171	(relation* adj (center* or centr*) adj (care or caring)).tw,kf.	181
172	or/152-171	390243
173	151 and 172	1729
174	limit 173 to english language	1680
175	limit 174 to yr="2017 -Current"	573

Database: MEDLINE Epub Ahead of Print and In-Process
Search strategy:

#	Searches	Results
1	computer user training*.tw,kf.	5

2	(computer assisted instruction* or computerised programmed instruction* or computerised self instruction program* or computerized programmed instruction* or computerized self instruction program*).tw,kf.	52
3	(interactive tutorial or interactive tutorials or webcast* or "web cast*").tw,kf.	63
4	(inservice or "in service" or "on the job training" or "training on the job").tw,kf.	1686
5	(interactive learning or simulation or simulat*).tw,kf.	188424
6	(continuing education or continuing nursing education or "post basic nursing education" or "post registration nursing education").tw,kf.	1085
7	or/1-6	191163
8	(telemedicine or telecardiology or teledermatology or telediagnosis or telediagnoses or telemonitoring or telenephrology or teleneurology or telepsychology or teleradiology or teleradiotherap* or telesurger* or teletherap* or videoconsult*).tw,kf.	5120
9	("tele medicine" or "tele cardiology" or "tele dermatology" or "tele diagnosis" or "tele diagnoses" or "tele monitoring" or "tele nephrology" or "tele neurology" or "tele psychology" or "tele radiology" or "tele radiotherap*" or "tele surger*" or "tele therap*" or "video consult*").tw,kf.	303
10	("e-health" or ehealth or "tele health").tw,kf.	2241
11	(telenurs* or "tele nurs*").tw,kf.	57
12	("cyber consult*" or cyberconsult* or econsult* or "e−consult*" or "email based consult*" or "internet consult*" or "internet based consult*" or "online consult*" or "tele consult*" or "telephone based consult*" or "telephone consult*" or "virtual consult*" or "web consult*" or "webbased consult*" or "web based consult*").tw,kf.	458
13	("e−rehabilitation" or remote rehabilitation tele rehabilitation or virtual rehabilitation).tw,kf.	41
14	(computerised medical record system* or computerized medical record system*).tw,kf.	15
15	(electronic health record* or computerised patient record* or computerized patient record* or electronic medical record* or electronic patient record* or ehr or emr).tw,kf.	9908
16	(smart card or smart cards).tw,kf.	57
17	medical order entry system*.tw,kf.	27
18	(hospital information system* or patient health record information system*).tw,kf.	380
19	ambulatory care information system*.tw,kf.	6
20	("e−prescribing" or "e−prescription*" or electronic prescription*).tw,kf.	89
21	("c.p.o.e. system" or computer* order entry or computer* physician order entry or computer* provider order entry or computer* order entry or computer* prescriber order entry or computer* prescribing order entry or computer* prescription order entry or "cpoe").tw,kf.	213
22	(operating room information system* or operating room information management system*).tw,kf.	3
23	(bedside computing or bedside technolog* or point of care system* or point of care technolog* or point of care information system*).tw,kf.	162
24	bedside information system*.tw,kf.	0
25	(management information system* or management information).tw,kf.	289
26	clinical pharmacy information system*.tw,kf.	10

27	(database management system* or database management tool or database management tools or database manager system* or database managing system* or data base manager system* or data base management software or data base management system* or data base management tool or data base management tools).tw,kf.	92
28	decision support system*.tw,kf.	1229
29	(mobile app or mobile apps or mobile application* or mobile health app or mobile health apps or mobile health application* or portable software app or portable software apps or portable software application* or tablet app or tablet apps or tablet application*).tw,kf.	2884
30	(cell phone or cell phones or cellphone or cellphones or cellular phone or cellular phones or cellular telephone or cellular telephones or mobile phone or mobile phones or mobile telephone or mobile telephones).tw,kf.	4204
31	(smartphone or smartphones or smart phone or smart phones).tw,kf.	5883
32	(text messaging or texting).tw,kf.	817
33	(video conference* or video conferencing or videoconferenc*).tw,kf.	966
34	(webcast* or web cast*).tw,kf.	47
35	(wireless communication* or wireless technol*).tw,kf.	1354
36	("cell phone usage" or "cell phone utilisation" or "cell phone utilization" or "cellphone usage" or "cellphone use" or "cellphone utilisation " or "cellphone utilization" or "cell phone use" or "mobile phone usage" or "mobile phone use" or "mobile phone utilisation" or "mobile phone utilization").tw,kf.	298
37	(hand held computer* or handheld computer* or palm PC or palmtop or personal data assistant* or personal digital assistant or pocket computer* or pocket sized computer*).tw,kf.	121
38	internet.tw,kf.	14061
39	(internet based intervention* or online based intervention* or online intervention* or web intervention* or web based intervention*).tw,kf.	903
40	smart technolog*.tw,kf.	138
41	(wearable computer or wearable computers or wearable electronic device*).tw,kf.	335
42	(activity tracker* or fitness tracker* or smart watch or smart watches or pedometer*).tw,kf.	801
43	("ar glasses" or "ar head mounted device" or "ar head mounted display" or "ar head worn display" or "ar headset" or "ar hud" or "arhmd" or "hmd ar" or "optical see through head mounted display" or "ost hmd" or "see through hmd" or "google glasses" or "smartglasses" or "smart glasses").tw,kf.	112
44	(augmented reality glasses or augmented reality head mounted device or augmented reality head mounted display or augmented reality head up display or augmented reality head worn display or head mounted display augmented reality).tw,kf.	16
45	(artificial intelligence or machine intelligence).tw,kf.	7915
46	computer heuristic*.tw,kf.	0
47	(expert system* or knowledge based system*).tw,kf.	446
48	(fuzzy logic or fuzzy model or fuzzy models).tw,kf.	977

49	(machine learning or learning machine*).tw,kf.	21448
50	(deep learning or hierarchical learning).tw,kf.	11505
51	unsupervised machine learning.tw,kf.	296
52	natural language processing.tw,kf.	1523
53	("ann approach*" or "ann model" or "ann method*" or "ann training" or artificial neural network or computer neural network or computer neural networks).tw,kf.	2544
54	(robotics or nanorobotics or robot or robots).tw,kf.	11439
55	(biomedical technology or bio medical technology).tw,kf.	100
56	informatics.tw,kf.	3293
57	(clinical informatics or clinical information science or clinical information technology or health informatics or health information science or health information technology or medical computer science or medical information science or medical informatics or medical information technology).tw,kf.	1882
58	nursing informatics.tw,kf.	63
59	health information exchange.tw,kf.	197
60	medical informatics applications.tw,kf.	39
61	(decision support system* or decision support techniques).tw,kf.	1358
62	(automatic diagnosis or computer assisted diagnosis or computer diagnosis or automatic diagnoses or computer assisted diagnoses or computer diagnoses).tw,kf.	245
63	computer assisted image interpretation.tw,kf.	3
64	computer assisted radiographic image interpretation*.tw,kf.	8
65	computer assisted therap*.tw,kf.	23
66	computer assisted drug therap*.tw,kf.	1
67	clinical decision support system*.tw,kf.	494
68	(information system or information systems).tw,kf.	5192
69	(online system or online systems).tw,kf.	154
70	(computer interface* or computer user interface*).tw,kf.	1586
71	(social media or social medium or Facebook or Flickr or Instagram or LinkedIn or MySpace or Pinterest or Reddit or "Sina Weibo" or Snapchat or online social network* or social networking platform* or social networking site* or social networking website* or social platform* or TikTok or Tumblr or Twitter or "web 2.0" or "web 2.0s" or WeChat or WhatsApp or YouTube).tw,kf.	9055
72	(virtual reality or virtual realities).tw,kf.	3335
73	(augmented realities or augmented reality or mixed realities or mixed reality).tw,kf.	1292
74	(hologram* or holograph*).tw,kf.	8961
75	("3d printing*" or "3 d printing*" or "3 dimensional printing*" or "three dimensional printing*").tw,kf.	5647
76	(chatbot or chatbots or "chat bot" or "chat bots").tw,kf.	181
77	"virtual care".tw,kf.	235
78	(closed loop medicines system* or closed loop medication system* or closed loop medicines process* or closed loop medication process*).tw,kf.	0

79	(bedside station or bedside stations or bed side station or bed side stations or bedside terminal or bedside terminals or bed side terminal or bed side terminals).tw,kf.	0
80	predictive analytic*.tw,kf.	285
81	or/8-80	122823
82	7 and 81	9843
83	(nurse* patient relation* or patient nurs* relation* or professional* patient relation* or patient professional* relation* or doctor patient relation* or patient doctor relation* or physician patient relation* or patient physician relation* or patient healthcare professional relation* or patient health care professional relation*).tw,kf.	1293
84	(interpersonal relation* or "inter personal relation*" or social relation*).tw,kf.	2913
85	(interpersonal care or "inter personal care").tw,kf.	12
86	(interpersonal interaction? or "inter personal interaction?").tw,kf.	247
87	(caring or compassion* or empathy or empathetic or sympathy or sympathetic).tw,kf.	16203
88	rapport.tw,kf.	1471
89	humanistic care.tw,kf.	28
90	(patient centered care or patient centered nursing or patient centred care or patient centred nursing or patient focused care).tw,kf.	1776
91	(interpersonal communication* or "inter personal communication*").tw,kf.	313
92	professionalism*.tw,kf.	1121
93	etiquette.tw,kf.	114
94	(webside manner? or web side manner? or virtual manner?).tw,kf.	6
95	(bedside manner? or bed side manner?).tw,kf.	24
96	(communication skill? or communication technique?).tw,kf.	2025
97	(relation* adj (center* or centr*) adj (care or caring)).tw,kf.	39
98	or/83-97	26295
99	82 and 98	80
100	limit 99 to english language	80
101	limit 100 to yr="2017 -Current"	68

Database: Embase
Search strategy:

#	Searches	Results
1	human computer interaction/	6512
2	computer user training*.tw,kw.	21
3	(computer assisted instruction* or computerised programmed instruction* or computerised self instruction program* or computerized programmed instruction* or computerized self instruction program*).tw,kw.	963
4	webcast/	414
5	(interactive tutorial or interactive tutorials or webcast* or "web cast*").tw,kf.	617

6	"in service training"/	16694
7	(inservice or "in service" or "on the job training" or "training on the job").tw,kw.	13822
8	simulation/ or computer simulation/ or digital twin/ or discrete event simulation/ or high fidelity simulation/ or high-fidelity patient simulation/ or patient simulation/	340215
9	(interactive learning or simulation or simulat*).tw,kw.	664153
10	continuing education/	32835
11	(continuing education or continuing nursing education or "post basic nursing education" or "post registration nursing education").tw,kw.	17606
12	or/1-11	819701
13	telemedicine/ or telecardiology/ or teledermatology/ or telediagnosis/ or telemonitoring/ or telenephrology/ or teleneurology/ or telepsychology/ or teleradiology/ or teleradiotherapy/ or telesurgery/ or teletherapy/ or video consultation/	44650
14	(telemedicine or telecardiology or teledermatology or telediagnosis or telediagnoses or telehealth or telemonitoring or telenephrology or teleneurology or telepsychology or teleradiology or teleradiotherap* or telesurger* or teletherap* or videoconsult*).tw,kw.	41200
15	("tele medicine" or "tele cardiology" or "tele dermatology" or "tele diagnosis" or "tele diagnoses" or "tele monitoring" or "tele nephrology" or "tele neurology" or "tele psychology" or "tele radiology" or "tele radiotherap*" or "tele surger*" or "tele therap*" or "video consult*").tw,kw.	1893
16	telehealth/	12152
17	("e-health" or ehealth or "tele health").tw,kw.	10358
18	telenursing/	319
19	(telenurs* or "tele nurs*").tw,kw.	328
20	teleconsultation/ or electronic consultation/	13110
21	("cyber consult*" or cyberconsult* or econsult* or "e−consult*" or "email based consult*" or "internet consult*" or "internet based consult*" or "online consult*" or "tele consult*" or "telephone based consult*" or "telephone consult*" or "virtual consult*" or "web consult*" or "webbased consult*" or "web based consult*").tw,kw.	3549
22	telerehabilitation/	1639
23	("e−rehabilitation" or remote rehabilitation or tele rehabilitation or virtual rehabilitation).tw,kw.	667
24	electronic medical record system/	1932
25	(computerised medical record system* or computerized medical record system*).tw,kw.	169
26	electronic health record/ or electronic medical record/ or electronic patient record/	96258
27	(electronic health record* or computerised patient record* or computerized patient record* or electronic medical record* or electronic patient record* or ehr or emr).tw,kw.	98978
28	smart card/	263
29	(smart card or smart cards).tw,kw.	447
30	physician order entry system/	314

31	medical order entry system*.tw,kw.	151
32	hospital information system/	20704
33	(hospital information system* or patient health record information system*).tw,kw.	4513
34	ambulatory care information system*.tw,kw.	39
35	electronic prescribing/	3642
36	("e–prescribing" or "e–prescription*" or electronic prescription*).tw,kw.	1336
37	computerized provider order entry/	2117
38	("c.p.o.e. system" or computer* order entry or computer* physician order entry or computer* provider order entry or computer* order entry or computer* prescriber order entry or computer* prescribing order entry or computer* prescription order entry or "cpoe").tw,kw.	3074
39	operating room information system/	36
40	(operating room information system* or operating room information management system*).tw,kw.	56
41	"point of care system"/	3100
42	(bedside computing or bedside technolog* or point of care system* or point of care technolog* or point of care information system*).tw,kw.	1214
43	bedside information system/	14
44	bedside information system*.tw,kw.	11
45	information system/	40658
46	(management information system* or management information).tw,kw.	2857
47	medical information system/	22304
48	clinical pharmacy information system*.tw,kw.	32
49	database management system/	567
50	(database management system* or database management tool or database management tools or database manager system* or database managing system* or data base manager system* or data base management software or data base management system* or data base management tool or data base management tools).tw,kw.	910
51	decision support system/	24865
52	decision support system*.tw,kw.	8861
53	mobile application/ or mobile health application/	18923
54	(mobile app or mobile apps or mobile application* or mobile health app or mobile health apps or mobile health application* or portable software app or portable software apps or portable software application* or tablet app or tablet apps or tablet application*).tw,kw.	10095
55	mobile phone/	19662
56	(cell phone or cell phones or cellphone or cellphones or cellular phone or cellular phones or cellular telephone or cellular telephones or mobile phone or mobile phones or mobile telephone or mobile telephones).tw,kw.	19055
57	smartphone/	19388
58	(smartphone or smartphones or smart phone or smart phones).tw,kw.	24488

59	text messaging/	6419
60	(text messaging or texting).tw,kw.	4425
61	videoconferencing/	6641
62	(video conference* or video conferencing or videoconferenc*).tw,kw.	5971
63	webcast/	414
64	(webcast* or web cast*).tw,kw.	480
65	wireless communication/	6402
66	(wireless communication* or wireless technol*).tw,kw.	2862
67	"cell phone use"/	1316
68	("cell phone usage" or "cell phone utilisation" or "cell phone utilization" or "cellphone usage" or "cellphone use" or "cellphone utilisation " or "cellphone utilization" or "cell phone use" or "mobile phone usage" or "mobile phone use" or "mobile phone utilisation" or "mobile phone utilization").tw,kw.	1864
69	personal digital assistant/	1680
70	(hand held computer* or handheld computer* or palm PC or palmtop or personal data assistant* or personal digital assistant or pocket computer* or pocket sized computer*).tw,kw.	1777
71	internet/	116402
72	internet.tw,kw.	83530
73	web-based intervention/	1512
74	(internet based intervention* or online based intervention* or online intervention* or web intervention* or web based intervention*).tw,kw.	3817
75	smart technolog*.tw,kw.	465
76	wearable computer/	866
77	(wearable computer or wearable computers or wearable electronic device*).tw,kw.	504
78	smart watch/ or activity tracker/	1902
79	(activity tracker* or fitness tracker* or smart watch or smart watches or pedometer*).tw,kw.	5612
80	smart glasses/	180
81	("ar glasses" or "ar head mounted device" or "ar head mounted display" or "ar head worn display" or "ar headset" or "ar hud" or "arhmd" or "hmd ar" or "optical see through head mounted display" or "ost hmd" or "see through hmd" or "google glasses" or "smartglasses" or "smart glasses").tw,kw.	344
82	artificial intelligence/	38976
83	(artificial intelligence or machine intelligence).tw,kw.	24385
84	computer heuristics/	349
85	computer heuristic*.tw,kw.	1
86	expert system/	5595
87	(expert system* or knowledge based system*).tw,kw.	5120
88	fuzzy logic/	4489
89	(fuzzy logic or fuzzy model or fuzzy models).tw,kw.	3434

90	machine learning/	53991
91	(machine learning or learning machine*).tw,kw.	69273
92	deep learning/	22139
93	(deep learning or hierarchical learning).tw,kw.	31386
94	unsupervised machine learning/	1471
95	unsupervised machine learning.tw,kw.	991
96	natural language processing/	7374
97	natural language processing.tw,kw.	6397
98	artificial neural network/	42150
99	("ann approach*" or "ann model" or "ann method*" or "ann training" or artificial neural network or computer neural network or computer neural networks).tw,kw.	11541
100	robotics/ or nanorobotics/	43522
101	(robotics or nanorobotics or robot or robots).tw,kw.	45882
102	medical technology/	36441
103	(biomedical technology or bio medical technology).tw,kw.	609
104	information science/	12920
105	informatics.tw,kw.	18584
106	medical informatics/	21952
107	(clinical informatics or clinical information science or clinical information technology or health informatics or health information science or health information technology or medical computer science or medical information science or medical informatics or medical information technology).tw,kw.	11610
108	nursing informatics/	1651
109	nursing informatics.tw,kw.	981
110	health information exchange.tw,kw.	1257
111	(decision support system* or decision support techniques).tw,kw.	9563
112	computer assisted diagnosis/	42663
113	(automatic diagnosis or computer assisted diagnosis or computer diagnosis or automatic diagnoses or computer assisted diagnoses or computer diagnoses).tw,kw.	2054
114	computer assisted image interpretation.tw,kw.	48
115	computer assisted radiographic image interpretation*.tw,kw.	28
116	computer assisted therapy/	4803
117	computer assisted therap*.tw,kw.	155
118	computer assisted drug therapy/	928
119	computer assisted drug therap*.tw,kw.	35
120	clinical decision support system/	4428
121	clinical decision support system*.tw,kw.	3400
122	information system/	40658
123	(information system or information systems).tw,kw.	48029
124	online system/	28950
125	(online system or online systems).tw,kw.	1039

126	computer interface/	34167
127	(computer interface* or computer user interface*).tw,kw.	7501
128	social media/	33413
129	(social media or social medium or Facebook or Flickr or Instagram or LinkedIn or MySpace or Pinterest or Reddit or "Sina Weibo" or Snapchat or online social network* or social networking platform* or social networking site* or social networking website* or social platform* or TikTok or Tumblr or Twitter or "web 2.0" or "web 2.0s" or WeChat or WhatsApp or YouTube).tw,kw.	37674
130	virtual reality/	21553
131	(virtual reality or virtual realities).tw,kw.	17307
132	augmented reality/	1196
133	(augmented realities or augmented reality or mixed realities or mixed reality).tw,kw.	4155
134	holography/	4216
135	(hologram* or holograph*).tw,kw.	6881
136	three dimensional printing/	16568
137	("3d printing*" or "3 d printing*" or "3 dimensional printing*" or "three dimensional printing*").tw,kw.	13336
138	(chatbot or chatbots or "chat bot" or "chat bots").tw,kw.	416
139	"virtual care".tw,kw.	761
140	(closed loop medicines system* or closed loop medication system* or closed loop medicines process* or closed loop medication process*).tw,kw.	6
141	(bedside station or bedside stations or bed side station or bed side stations or bedside terminal or bedside terminals or bed side terminal or bed side terminals).tw,kw.	57
142	predictive analytic?.tw,kw.	995
143	or/13-142	956810
144	12 and 143	73142
145	human relation/	98469
146	professional-patient relationship/ or doctor patient relationship/ or nurse patient relationship/	52641
147	(nurse* patient relation* or patient nurs* relation* or professional* patient relation* or patient professional* relation* or doctor patient relation* or patient doctor relation* or physician patient relation* or patient physician relation* or patient healthcare professional relation* or patient health care professional relation*).tw,kw.	15516
148	(interpersonal relation* or "inter personal relation*" or social relation*).tw,kw.	23480
149	(interpersonal care or "inter personal care").tw,kw.	180
150	(interpersonal interaction? or "inter personal interaction?").tw,kw.	1576
151	empathy/	29882
152	(caring or compassion* or empathy or empathetic or sympathy or sympathetic).tw,kw.	216145
153	rapport.tw,kw.	4772
154	humanism/	3791
155	humanistic care.tw,kw.	207

156	(patient centered care or patient centered nursing or patient centred care or patient centred nursing or patient focused care).tw,kw.	12627
157	interpersonal communication/	181535
158	(interpersonal communication* or "inter personal communication*").tw,kw.	2372
159	professionalism/	10212
160	professionalism.tw,kw.	10934
161	etiquette.tw,kw.	757
162	(webside manner? or web side manner? or virtual manner?).tw,kw.	32
163	(bedside manner? or bed side manner?).tw,kw.	272
164	communication skill/	15894
165	(communication skill? or communication technique?).tw,kw.	17717
166	(relation* adj (center* or centr*) adj (care or caring)).tw,kw.	208
167	or/145-166	592034
168	144 and 167	2135
169	limit 168 to english language	2091
170	limit 169 to yr="2017 -Current"	686
171	limit 170 to (conference abstract or conference paper or "conference review")	96
172	170 not 171	590

Database: Emcare Nursing
Search strategy:

#	Searches	Results
1	human computer interaction/	4094
2	computer user training*.tw,kw.	13
3	(computer assisted instruction* or computerised programmed instruction* or computerised self instruction program* or computerized programmed instruction* or computerized self instruction program*).tw,kw.	440
4	webcast/	84
5	(interactive tutorial or interactive tutorials or webcast* or "web cast*").tw,kf.	160
6	"in service training"/	1789
7	(inservice or "in service" or "on the job training" or "training on the job").tw,kw.	6748
8	simulation/ or computer simulation/ or digital twin/ or discrete event simulation/ or high fidelity simulation/ or high-fidelity patient simulation/ or patient simulation/	81503
9	(interactive learning or simulation or simulat*).tw,kw.	144282
10	continuing education/	17807
11	(continuing education or continuing nursing education or "post basic nursing education" or "post registration nursing education").tw,kw.	7774
12	or/1-11	185048

13	telemedicine/ or telecardiology/ or teledermatology/ or telediagnosis/ or telemonitoring/ or telenephrology/ or teleneurology/ or telepsychology/ or teleradiology/ or teleradiotherapy/ or telesurgery/ or teletherapy/ or video consultation/	16384
14	(telemedicine or telecardiology or teledermatology or telediagnosis or telediagnoses or telehealth or telemonitoring or telenephrology or teleneurology or telepsychology or teleradiology or teleradiotherap* or telesurger* or teletherap* or videoconsult*).tw,kw.	18687
15	("tele medicine" or "tele cardiology" or "tele dermatology" or "tele diagnosis" or "tele diagnoses" or "tele monitoring" or "tele nephrology" or "tele neurology" or "tele psychology" or "tele radiology" or "tele radiotherap*" or "tele surger*" or "tele therap*" or "video consult*").tw,kw.	661
16	telehealth/	8890
17	("e-health" or ehealth or "tele health").tw,kw.	6944
18	telenursing/	295
19	(telenurs* or "tele nurs*").tw,kw.	333
20	teleconsultation/ or electronic consultation/	5189
21	("cyber consult*" or cyberconsult* or econsult* or "e−consult*" or "email based consult*" or "internet consult*" or "internet based consult*" or "online consult*" or "tele consult*" or "telephone based consult*" or "telephone consult*" or "virtual consult*" or "web consult*" or "webbased consult*" or "web based consult*").tw,kw.	1179
22	telerehabilitation/	721
23	("e−rehabilitation" or remote rehabilitation or tele rehabilitation or virtual rehabilitation).tw,kw.	324
24	electronic medical record system/	518
25	(computerised medical record system* or computerized medical record system*).tw,kw.	107
26	electronic health record/ or electronic medical record/ or electronic patient record/	31758
27	(electronic health record* or computerised patient record* or computerized patient record* or electronic medical record* or electronic patient record* or ehr or emr).tw,kw.	29447
28	smart card/	261
29	(smart card or smart cards).tw,kw.	281
30	physician order entry system/	67
31	medical order entry system*.tw,kw.	146
32	hospital information system/	6857
33	(hospital information system* or patient health record information system*).tw,kw.	1888
34	ambulatory care information system*.tw,kw.	22
35	electronic prescribing/	1313
36	("e−prescribing" or "e−prescription*" or electronic prescription*).tw,kw.	446
37	computerized provider order entry/	1223

38	("c.p.o.e. system" or computer* order entry or computer* physician order entry or computer* provider order entry or computer* order entry or computer* prescriber order entry or computer* prescribing order entry or computer* prescription order entry or "cpoe").tw,kw.	1520
39	operating room information system/	16
40	(operating room information system* or operating room information management system*).tw,kw.	21
41	"point of care system"/	447
42	(bedside computing or bedside technolog* or point of care system* or point of care technolog* or point of care information system*).tw,kw.	478
43	bedside information system/	0
44	bedside information system*.tw,kw.	6
45	information system/	18445
46	(management information system* or management information).tw,kw.	1168
47	medical information system/	13424
48	clinical pharmacy information system*.tw,kw.	18
49	database management system/	106
50	(database management system* or database management tool or database management tools or database manager system* or database managing system* or data base manager system* or data base management software or data base management system* or data base management tool or data base management tools).tw,kw.	278
51	decision support system/	10718
52	decision support system*.tw,kw.	4261
53	mobile application/ or mobile health application/	6758
54	(mobile app or mobile apps or mobile application* or mobile health app or mobile health apps or mobile health application* or portable software app or portable software apps or portable software application* or tablet app or tablet apps or tablet application*).tw,kw.	5002
55	mobile phone/	7640
56	(cell phone or cell phones or cellphone or cellphones or cellular phone or cellular phones or cellular telephone or cellular telephones or mobile phone or mobile phones or mobile telephone or mobile telephones).tw,kw.	8677
57	smartphone/	4503
58	(smartphone or smartphones or smart phone or smart phones).tw,kw.	9652
59	text messaging/	3120
60	(text messaging or texting).tw,kw.	2564
61	videoconferencing/	2880
62	(video conference* or video conferencing or videoconferenc*).tw,kw.	2903
63	webcast/	84
64	(webcast* or web cast*).tw,kw.	107
65	wireless communication/	1761

66	(wireless communication* or wireless technol*).tw,kw.	884
67	"cell phone use"/	660
68	("cell phone usage" or "cell phone utilisation" or "cell phone utilization" or "cellphone usage" or "cellphone use" or "cellphone utilisation " or "cellphone utilization" or "cell phone use" or "mobile phone usage" or "mobile phone use" or "mobile phone utilisation" or "mobile phone utilization").tw,kw.	932
69	personal digital assistant/	981
70	(hand held computer* or handheld computer* or palm PC or palmtop or personal data assistant* or personal digital assistant or pocket computer* or pocket sized computer*).tw,kw.	1031
71	internet/	54147
72	internet.tw,kw.	41934
73	web-based intervention/	735
74	(internet based intervention* or online based intervention* or online intervention* or web intervention* or web based intervention*).tw,kw.	2531
75	smart technolog*.tw,kw.	210
76	wearable computer/	41
77	(wearable computer or wearable computers or wearable electronic device*).tw,kw.	184
78	smart watch/ or activity tracker/	298
79	(activity tracker* or fitness tracker* or smart watch or smart watches or pedometer*).tw,kw.	2769
80	smart glasses/	25
81	("ar glasses" or "ar head mounted device" or "ar head mounted display" or "ar head worn display" or "ar headset" or "ar hud" or "arhmd" or "hmd ar" or "optical see through head mounted display" or "ost hmd" or "see through hmd" or "google glasses" or "smartglasses" or "smart glasses").tw,kw.	104
82	artificial intelligence/	7189
83	(artificial intelligence or machine intelligence).tw,kw.	6961
84	computer heuristics/	97
85	computer heuristic*.tw,kw.	1
86	expert system/	1925
87	(expert system* or knowledge based system*).tw,kw.	1313
88	fuzzy logic/	725
89	(fuzzy logic or fuzzy model or fuzzy models).tw,kw.	932
90	machine learning/	12938
91	(machine learning or learning machine*).tw,kw.	19372
92	deep learning/	5107
93	(deep learning or hierarchical learning).tw,kw.	8550
94	unsupervised machine learning/	285
95	unsupervised machine learning.tw,kw.	236
96	natural language processing/	2456

97	natural language processing.tw,kw.	2947
98	artificial neural network/	7545
99	("ann approach*" or "ann model" or "ann method*" or "ann training" or artificial neural network or computer neural network or computer neural networks).tw,kw.	2669
100	robotics/ or nanorobotics/	10772
101	(robotics or nanorobotics or robot or robots).tw,kw.	10067
102	medical technology/	12665
103	(biomedical technology or bio medical technology).tw,kw.	257
104	information science/	6723
105	informatics.tw,kw.	9443
106	medical informatics/	11589
107	(clinical informatics or clinical information science or clinical information technology or health informatics or health information science or health information technology or medical computer science or medical information science or medical informatics or medical information technology).tw,kw.	8468
108	nursing informatics/	1172
109	nursing informatics.tw,kw.	1054
110	health information exchange.tw,kw.	814
111	(decision support system* or decision support techniques).tw,kw.	4597
112	computer assisted diagnosis/	4363
113	(automatic diagnosis or computer assisted diagnosis or computer diagnosis or automatic diagnoses or computer assisted diagnoses or computer diagnoses).tw,kw.	590
114	computer assisted image interpretation.tw,kw.	25
115	computer assisted radiographic image interpretation*.tw,kw.	14
116	computer assisted therapy/	1253
117	computer assisted therap*.tw,kw.	98
118	computer assisted drug therapy/	166
119	computer assisted drug therap*.tw,kw.	15
120	clinical decision support system/	1786
121	clinical decision support system*.tw,kw.	1895
122	information system/	18445
123	(information system or information systems).tw,kw.	19545
124	online system/	11093
125	(online system or online systems).tw,kw.	397
126	computer interface/	7224
127	(computer interface* or computer user interface*).tw,kw.	2320
128	social media/	16142
129	(social media or social medium or Facebook or Flickr or Instagram or LinkedIn or MySpace or Pinterest or Reddit or "Sina Weibo" or Snapchat or online social network* or social networking platform* or social networking site* or social networking website*	21060

	or social platform* or TikTok or Tumblr or Twitter or "web 2.0" or "web 2.0s" or WeChat or WhatsApp or YouTube).tw,kw.	
130	virtual reality/	10646
131	(virtual reality or virtual realities).tw,kw.	8391
132	augmented reality/	367
133	(augmented realities or augmented reality or mixed realities or mixed reality).tw,kw.	1586
134	holography/	587
135	(hologram* or holograph*).tw,kw.	1023
136	three dimensional printing/	3340
137	("3d printing*" or "3 d printing*" or "3 dimensional printing*" or "three dimensional printing*").tw,kw.	3128
138	(chatbot or chatbots or "chat bot" or "chat bots").tw,kw.	241
139	"virtual care".tw,kw.	340
140	(closed loop medicines system* or closed loop medication system* or closed loop medicines process* or closed loop medication process*).tw,kw.	2
141	(bedside station or bedside stations or bed side station or bed side stations or bedside terminal or bedside terminals or bed side terminal or bed side terminals).tw,kw.	7
142	predictive analytic?.tw,kw.	383
143	or/13-142	324547
144	12 and 143	19580
145	human relation/	21835
146	professional-patient relationship/ or doctor patient relationship/ or nurse patient relationship/	11400
147	(nurse* patient relation* or patient nurs* relation* or professional* patient relation* or patient professional* relation* or doctor patient relation* or patient doctor relation* or physician patient relation* or patient physician relation* or patient healthcare professional relation* or patient health care professional relation*).tw,kw.	6640
148	(interpersonal relation* or "inter personal relation*" or social relation*).tw,kw.	13463
149	(interpersonal care or "inter personal care").tw,kw.	117
150	(interpersonal interaction? or "inter personal interaction?").tw,kw.	974
151	empathy/	14030
152	(caring or compassion* or empathy or empathetic or sympathy or sympathetic).tw,kw.	66901
153	rapport.tw,kw.	2313
154	humanism/	1048
155	humanistic care.tw,kw.	100
156	(patient centered care or patient centered nursing or patient centred care or patient centred nursing or patient focused care).tw,kw.	7471
157	interpersonal communication/	85767
158	(interpersonal communication* or "inter personal communication*").tw,kw.	1479
159	professionalism/	7718
160	professionalism.tw,kw.	5969

161	etiquette.tw,kw.	332
162	(webside manner? or web side manner? or virtual manner?).tw,kw.	15
163	(bedside manner? or bed side manner?).tw,kw.	86
164	communication skill/	11901
165	(communication skill? or communication technique?).tw,kw.	9239
166	(relation* adj (center* or centr*) adj (care or caring)).tw,kw.	158
167	or/145-166	214991
168	144 and 167	1158
169	limit 168 to english language	1137
170	limit 169 to yr="2017 -Current"	234
171	limit 170 to (conference abstract or conference paper or "conference review")	4
172	170 not 171	230

Database: Cochrane Central Register of Controlled Trials
Search strategy:

#	Searches	Results
1	Computer User Training/	53
2	computer user training*.tw,kw.	6
3	Computer Assisted Instruction/	1264
4	(computer assisted instruction* or computerised programmed instruction* or computerised self instruction program* or computerized programmed instruction* or computerized self instruction program*).tw,kw.	204
5	interactive tutorial/ or webcast/	0
6	(interactive tutorial or interactive tutorials or webcast* or "web cast*").tw,kw.	26
7	InService Training/	753
8	(inservice or "in service" or "on the job training" or "training on the job").tw,kw.	23160
9	Simulation Training/ or High Fidelity Simulation Training/ or Patient Simulation/	1140
10	(interactive learning or simulation or simulat*).tw,kw.	21729
11	education, continuing/ or education, nursing, continuing/	409
12	(continuing education or continuing nursing education or "post basic nursing education" or "post registration nursing education").tw,kw.	453
13	exp Health Personnel/ and ed.fs.	2144
14	or/1-13	48384
15	telemedicine/	2617
16	(telemedicine or telecardiology or teledermatology or telediagnosis or telediagnoses or telehealth or telemonitoring or telenephrology or teleneurology or telepsychology or teleradiology or teleradiotherap* or telesurger* or teletherap* or videoconsult*).tw,kw.	5736

17	("tele medicine" or "tele cardiology" or "tele dermatology" or "tele diagnosis" or "tele diagnoses" or "tele monitoring" or "tele nephrology" or "tele neurology" or "tele psychology" or "tele radiology" or "tele radiotherap*" or "tele surger*" or "tele therap*" or "video consult*").tw,kw.	258
18	("e-health" or ehealth or "tele health").tw,kw.	1329
19	(telenurs* or "tele nurs*").tw,kw.	150
20	remote consultation/	389
21	("cyber consult*" or cyberconsult* or econsult* or "e−consult*" or "email based consult*" or "internet consult*" or "internet based consult*" or "online consult*" or "tele consult*" or "telephone based consult*" or "telephone consult*" or "virtual consult*" or "web consult*" or "webbased consult*" or "web based consult*").tw,kw.	533
22	telerehabilitation/	154
23	("e−rehabilitation" or remote rehabilitation or tele rehabilitation or virtual rehabilitation).tw,kw.	253
24	medical records systems, computerized/	199
25	(computerised medical record system* or computerized medical record system*).tw,kw.	8
26	electronic health records/	431
27	(electronic health record* or computerised patient record* or computerized patient record* or electronic medical record* or electronic patient record* or ehr or emr).tw,kw.	5888
28	health smart cards/	1
29	(smart card or smart cards).tw,kw.	17
30	medical order entry systems/	71
31	medical order entry system*.tw,kw.	0
32	hospital information systems/	47
33	(hospital information system* or patient health record information system*).tw,kw.	126
34	ambulatory care information systems/	25
35	ambulatory care information system*.tw,kw.	1
36	electronic prescribing/	25
37	("e−prescribing" or "e−prescription*" or electronic prescription*).tw,kw.	53
38	("c.p.o.e. system" or computer* order entry or computer* physician order entry or computer* provider order entry or computer* order entry or computer* prescriber order entry or computer* prescribing order entry or computer* prescription order entry or "cpoe").tw,kw.	135
39	operating room information systems/	3
40	(operating room information system* or operating room information management system*).tw,kw.	2
41	Point-of-Care Systems/	464
42	(bedside computing or bedside technolog* or point of care system* or point of care technolog* or point of care information system*).tw,kw.	95
43	bedside information system*.tw,kw.	0

44	(management information system* or management information).tw,kw.	168
45	clinical pharmacy information systems/	21
46	clinical pharmacy information system*.tw,kw.	3
47	database management systems/	14
48	(database management system* or database management tool or database management tools or database manager system* or database managing system* or data base manager system* or data base management software or data base management system* or data base management tool or data base management tools).tw,kw.	16
49	decision support systems, management/	8
50	decision support system*.tw,kw.	1664
51	Mobile Applications/	984
52	(mobile app or mobile apps or mobile application* or mobile health app or mobile health apps or mobile health application* or portable software app or portable software apps or portable software application* or tablet app or tablet apps or tablet application*).tw,kw.	3057
53	cell phone/	764
54	(cell phone or cell phones or cellphone or cellphones or cellular phone or cellular phones or cellular telephone or cellular telephones or mobile phone or mobile phones or mobile telephone or mobile telephones).tw,kw.	4173
55	smartphone/	599
56	(smartphone or smartphones or smart phones or smart phones).tw,kw.	5306
57	text messaging/	1063
58	(text messaging or texting).tw,kw.	2294
59	videoconferencing/	218
60	(video conference* or video conferencing or videoconferenc*).tw,kw.	1322
61	webcast/	0
62	webcasts as topic/	27
63	(webcast* or web cast*).tw,kw.	18
64	Wireless Technology/	49
65	(wireless communication* or wireless technol*).tw,kw.	128
66	"Cell Phone Use"/	11
67	("cell phone usage" or "cell phone utilisation" or "cell phone utilization" or "cellphone usage" or "cellphone use" or "cellphone utilisation " or "cellphone utilization" or "cell phone use" or "mobile phone usage" or "mobile phone use" or "mobile phone utilisation" or "mobile phone utilization").tw,kw.	3206
68	computers, handheld/	305
69	(hand held computer* or handheld computer* or palm PC or palmtop or personal data assistant* or personal digital assistant or pocket computer* or pocket sized computer*).tw,kw.	263
70	internet/	4132
71	internet.tw,kw.	10935

72	internet-based intervention/	300
73	(internet based intervention* or online based intervention* or online intervention* or web intervention* or web based intervention*).tw,kw.	2742
74	smart technolog*.tw,kw.	21
75	wearable electronic devices/	121
76	(wearable computer or wearable computers or wearable electronic device*).tw,kw.	17
77	fitness trackers/	136
78	(activity tracker* or fitness tracker* or smart watch or smart watches or pedometer*).tw,kw.	2504
79	smart glasses/	7
80	("ar glasses" or "ar head mounted device" or "ar head mounted display" or "ar head worn display" or "ar headset" or "ar hud" or "arhmd" or "hmd ar" or "optical see through head mounted display" or "ost hmd" or "see through hmd" or "google glasses" or "smartglasses" or "smart glasses").tw,kw.	39
81	(augmented reality glasses or augmented reality head mounted device or augmented reality head mounted display or augmented reality head up display or augmented reality head worn display or head mounted display augmented reality).tw,kw.	6
82	artificial intelligence/	207
83	(artificial intelligence or machine intelligence).tw,kw.	820
84	expert systems/	60
85	(expert system* or knowledge based system*).tw,kw.	198
86	fuzzy logic/	40
87	(fuzzy logic or fuzzy model or fuzzy models).tw,kw.	52
88	machine learning/	140
89	(machine learning or learning machine*).tw,kw.	1867
90	deep learning/	36
91	(deep learning or hierarchical learning).tw,kw.	718
92	unsupervised machine learning/	0
93	unsupervised machine learning.tw,kw.	27
94	natural language processing/	12
95	natural language processing.tw,kw.	219
96	neural networks, computer/	126
97	("ann approach*" or "ann model" or "ann method*" or "ann training" or artificial neural network or computer neural network or computer neural networks).tw,kw.	329
98	robotics/	716
99	(robotics or nanorobotics or robot or robots).tw,kw.	3610
100	biomedical technology/	21
101	(biomedical technology or bio medical technology).tw,kw.	365
102	informatics/	2
103	informatics.tw,kw.	551
104	medical informatics/	85

105	(clinical informatics or clinical information science or clinical information technology or health informatics or health information science or health information technology or medical computer science or medical information science or medical informatics or medical information technology).tw,kw.	457
106	medical informatics computing/	0
107	nursing informatics/	10
108	nursing informatics.tw,kw.	18
109	health information exchange/	9
110	health information exchange.tw,kw.	42
111	medical informatics applications/	23
112	medical informatics applications.tw,kw.	3
113	decision making, computer-assisted/	134
114	(decision support system* or decision support techniques).tw,kw.	1694
115	diagnosis, computer-assisted/	735
116	(automatic diagnosis or computer assisted diagnosis or computer diagnosis or automatic diagnoses or computer assisted diagnoses or computer diagnoses).tw,kw.	165
117	image interpretation, computer-assisted/	992
118	computer assisted image interpretation.tw,kw.	0
119	radiographic image interpretation, computer-assisted/	402
120	computer assisted radiographic image interpretation*.tw,kw.	0
121	therapy, computer-assisted/	1373
122	computer assisted therap*.tw,kw.	265
123	drug therapy, computer-assisted/	153
124	computer assisted drug therap*.tw,kw.	23
125	decision support systems, clinical/	430
126	clinical decision support system*.tw,kw.	375
127	information systems/	66
128	(information system or information systems).tw,kw.	1746
129	online systems/	161
130	(online system or online systems).tw,kw.	645
131	user-computer interface/	1332
132	(computer interface* or computer user interface*).tw,kw.	591
133	Social Media/	234
134	(social media or social medium or Facebook or Flickr or Instagram or LinkedIn or MySpace or Pinterest or Reddit or "Sina Weibo" or Snapchat or online social network* or social networking platform* or social networking site* or social networking website* or social platform* or TikTok or Tumblr or Twitter or "web 2.0" or "web 2.0s" or WeChat or WhatsApp or YouTube).tw,kw.	3152
135	Virtual Reality/	413
136	(virtual reality or virtual realities).tw,kw.	4343
137	Augmented Reality/	19

138	(augmented realities or augmented reality or mixed realities or mixed reality).tw,kw.	313
139	holography/	5
140	(hologram* or holograph*).tw,kw.	74
141	Printing, Three-Dimensional/	96
142	("3d printing*" or "3 d printing*" or "3 dimensional printing*" or "three dimensional printing*").tw,kw.	378
143	(chatbot or chatbots or "chat bot" or "chat bots").tw,kw.	82
144	"virtual care".tw,kw.	40
145	(closed loop medicines system* or closed loop medication system* or closed loop medicines process* or closed loop medication process*).tw,kw.	0
146	(bedside station or bedside stations or bed side station or bed side stations or bedside terminal or bedside terminals or bed side terminal or bed side terminals).tw,kw.	0
147	predictive analytic*.tw,kw.	64
148	or/15-147	62769
149	14 and 148	7089
150	interpersonal relations/ or professional-patient relations/ or nurse-patient relations/ or physician- patient relations/	4892
151	(nurse* patient relation* or patient nurs* relation* or professional* patient relation* or patient professional* relation* or doctor patient relation* or patient doctor relation* or physician patient relation* or patient physician relation*).tw,kw.	5267
152	(interpersonal relation* or "inter personal relation*" or social relation*).tw,kw.	1553
153	(interpersonal care or "inter personal care").tw,kw.	8
154	Empathy/	566
155	(caring or compassion* or empathy or empathetic).tw,kw.	5776
156	rapport.tw,kw.	489
157	Humanism/	11
158	humanistic care.tw,kw.	15
159	Patient-Centered Care/	677
160	(patient centered care or patient centered nursing or patient centred care or patient centred nursing or patient focused care).tw,kw.	628
161	(interpersonal communication* or "inter personal communication*").tw,kw.	2215
162	Professionalism/	11
163	professionalism.tw,kw.	197
164	etiquette.tw,kw.	26
165	(webside manner? or web side manner? or virtual manner?).tw,kw.	0
166	(bedside manner? or bed side manner?).tw,kw.	8
167	Communication/	2503
168	(communication skill? or communication technique?).tw,kw.	2151
169	(relation* adj (center* or centr*) adj (care or caring)).tw,kw.	5
170	or/150-169	23014
171	149 and 170	474

172	limit 171 to english language	346
173	limit 172 to yr="2017 -Current"	145

Database: Cochrane Database of Systematic Reviews
Search strategy:

#	Searches	Results
1	(computer assisted instruction* or computerised programmed instruction* or computerised self instruction program* or computerized programmed instruction* or computerized self instruction program*).mp.	44
2	(interactive tutorial or interactive tutorials or webcast* or "web cast*").mp.	18
3	(inservice or "in service" or "on the job training" or "training on the job").mp.	118
4	(interactive learning or simulation or simulat*).mp.	445
5	(continuing education or continuing nursing education or "post basic nursing education" or "post registration nursing education").mp.	60
6	or/1-5	604
7	(telemedicine or telecardiology or teledermatology or telediagnosis or telediagnoses or telehealth or telemonitoring or telenephrology or teleneurology or telepsychology or teleradiology or teleradiotherap* or telesurger* or teletherap* or videoconsult*).mp.	173
8	("tele medicine" or "tele cardiology" or "tele dermatology" or "tele diagnosis" or "tele diagnoses" or "tele monitoring" or "tele nephrology" or "tele neurology" or "tele psychology" or "tele radiology" or "tele radiotherap*" or "tele surger*" or "tele therap*" or "video consult*").mp.	35
9	("e-health" or ehealth or "tele health").mp.	109
10	(telenurs* or "tele nurs*").mp.	25
11	("cyber consult*" or cyberconsult* or econsult* or "e−consult*" or "email based consult*" or "internet consult*" or "internet based consult*" or "online consult*" or "tele consult*" or "telephone based consult*" or "telephone consult*" or "virtual consult*" or "web consult*" or "webbased consult*" or "web based consult*").mp.	65
12	("e−rehabilitation" or remote rehabilitation or tele rehabilitation or virtual rehabilitation).mp.	11
13	(computerised medical record system* or computerized medical record system*).mp.	3
14	(electronic health record* or computerised patient record* or computerized patient record* or electronic medical record* or electronic patient record* or ehr or emr).mp.	98
15	(smart card or smart cards).mp.	2
16	medical order entry system*.mp.	6
17	(hospital information system* or patient health record information system*).mp.	10
18	ambulatory care information system*.mp.	5
19	("e−prescribing" or "e−prescription*" or electronic prescription*).mp.	4

20	("c.p.o.e. system" or computer* order entry or computer* physician order entry or computer* provider order entry or computer* order entry or computer* prescriber order entry or computer* prescribing order entry or computer* prescription order entry or "cpoe").mp.	14
21	(operating room information system* or operating room information management system*).mp.	1
22	(bedside computing or bedside technolog* or point of care system* or point of care technolog* or point of care information system*).mp.	23
23	(management information system* or management information).mp.	87
24	clinical pharmacy information system*.mp.	3
25	(database management system* or database management tool or database management tools or database manager system* or database managing system* or data base manager system* or data base management software or data base management system* or data base management tool or data base management tools).mp.	4
26	decision support system*.mp.	71
27	(mobile app or mobile apps or mobile application* or mobile health app or mobile health apps or mobile health application* or portable software app or portable software apps or portable software application* or tablet app or tablet apps or tablet application*).mp.	61
28	(cell phone or cell phones or cellphone or cellphones or cellular phone or cellular phones or cellular telephone or cellular telephones or mobile phone or mobile phones or mobile telephone or mobile telephones).mp.	207
29	(smartphone or smartphones or smart phone or smart phones).mp.	141
30	(text messaging or texting).mp.	97
31	(video conference* or video conferencing or videoconferenc*).mp.	75
32	(webcast* or web cast*).mp.	18
33	(wireless communication* or wireless technol*).mp.	22
34	("cell phone usage" or "cell phone utilisation" or "cell phone utilization" or "cellphone usage" or "cellphone use" or "cellphone utilisation " or "cellphone utilization" or "cell phone use" or "mobile phone usage" or "mobile phone use" or "mobile phone utilisation" or "mobile phone utilization").mp.	6
35	(hand held computer* or handheld computer* or palm PC or palmtop or personal data assistant* or personal digital assistant or pocket computer* or pocket sized computer*).mp.	41
36	internet.mp.	893
37	(internet based intervention* or online based intervention* or online intervention* or web intervention* or web based intervention*).mp.	88
38	smart technolog*.mp.	3
39	(wearable computer or wearable computers or wearable electronic device*).mp.	3
40	(activity tracker* or fitness tracker* or smart watch or smart watches or pedometer*).mp.	77
41	("ar glasses" or "ar head mounted device" or "ar head mounted display" or "ar head worn display" or "ar headset" or "ar hud" or "arhmd" or "hmd ar" or "optical see through	0

	head mounted display" or "ost hmd" or "see through hmd" or "google glasses" or "smartglasses" or "smart glasses").mp.	
42	(augmented reality glasses or augmented reality head mounted device or augmented reality head mounted display or augmented reality head up display or augmented reality head worn display or head mounted display augmented reality).mp.	0
43	(artificial intelligence or machine intelligence).mp.	33
44	(expert system* or knowledge based system*).mp.	13
45	(fuzzy logic or fuzzy model or fuzzy models).mp.	7
46	(machine learning or learning machine*).mp.	95
47	(deep learning or hierarchical learning).mp.	4
48	unsupervised machine learning.mp.	1
49	natural language processing.mp.	3
50	("ann approach*" or "ann model" or "ann method*" or "ann training" or artificial neural network or computer neural network or computer neural networks).mp.	2
51	(robotics or nanorobotics or robot or robots).mp.	65
52	(biomedical technology or bio medical technology).mp.	5
53	informatics.mp.	77
54	(clinical informatics or clinical information science or clinical information technology or health informatics or health information science or health information technology or medical computer science or medical information science or medical informatics or medical information technology).mp.	68
55	nursing informatics.mp.	11
56	health information exchange.mp.	1
57	medical informatics applications.mp.	11
58	(decision support system* or decision support techniques).mp.	82
59	(automatic diagnosis or computer assisted diagnosis or computer diagnosis or automatic diagnoses or computer assisted diagnoses or computer diagnoses).mp.	17
60	computer assisted image interpretation.mp.	0
61	computer assisted radiographic image interpretation*.mp.	0
62	computer assisted therap*.mp.	19
63	computer assisted drug therap*.mp.	3
64	clinical decision support system*.mp.	19
65	(information system or information systems).mp.	127
66	(online system or online systems).mp.	10
67	(computer interface* or computer user interface*).mp.	28
68	(social media or social medium or Facebook or Flickr or Instagram or LinkedIn or MySpace or Pinterest or Reddit or "Sina Weibo" or Snapchat or online social network* or social networking platform* or social networking site* or social networking website* or social platform* or TikTok or Tumblr or Twitter or "web 2.0" or "web 2.0s" or WeChat or WhatsApp or YouTube).mp.	145
69	(virtual reality or virtual realities).mp.	85
70	(augmented realities or augmented reality or mixed realities or mixed reality).mp.	5

71	(hologram* or holograph*).mp.	1
72	("3d printing*" or "3 d printing*" or "3 dimensional printing*" or "three dimensional printing*").mp.	0
73	(chatbot or chatbots or "chat bot" or "chat bots").mp.	1
74	"virtual care".mp.	0
75	predictive analytic*.mp.	0
76	or/7-75	1646
77	6 and 76	244
78	(nurse* patient relation* or patient nurs* relation* or professional* patient relation* or patient professional* relation* or doctor patient relation* or patient doctor relation* or physician patient relation* or patient physician relation*).mp.	78
79	(interpersonal relation* or "inter personal relation*" or social relation*).mp.	227
80	(interpersonal care or "inter personal care").mp.	3
81	(caring or compassion* or empathy or empathetic).mp.	529
82	rapport.mp.	43
83	humanistic care.mp.	0
84	(patient centered care or patient centered nursing or patient centred care or patient centred nursing or patient focused care).mp.	82
85	(interpersonal communication* or "inter personal communication*").mp.	43
86	professionalism*.mp.	18
87	etiquette.mp.	6
88	(webside manner? or web side manner? or virtual manner?).mp.	0
89	(bedside manner? or bed side manner?).mp.	0
90	(communication skill? or communication technique?).mp.	149
91	(relation* adj (center* or centr*) adj (care or caring)).mp.	1
92	or/78-91	920
93	77 and 92	96
94	limit 93 to last 6 years	37
95	limit 94 to full systematic reviews	32

Database: APA PsychInfo
Search strategy:

#	Searches	Results
1	human computer interaction/	11768
2	computer user training*.tw,id.	3
3	computer assisted instruction/ or computer supported collaborative learning/ or intelligent tutoring systems/	18166

4	electronic learning/ or adaptive learning/ or asynchronous learning/ or digital game-based learning/ or mobile learning/	3547
5	(computer assisted instruction* or computerised programmed instruction* or computerised self instruction program* or computerized programmed instruction* or computerized self instruction program*).tw,id.	1868
6	(interactive tutorial or interactive tutorials or webcast* or "web cast*").tw,id.	121
7	inservice training/ or on the job training/	1127
8	(inservice or "in service" or "on the job training" or "training on the job").tw,id.	13023
9	simulation/ or simulation games/	22599
10	(interactive learning or simulation* or simulat*).tw,id.	76120
11	continuing education/	1806
12	professional development/	21459
13	(continuing education or continuing nursing education or "post basic nursing education" or "post registration nursing education").tw,id.	4077
14	or/1-13	150387
15	telemedicine/ or online therapy/ or teleconsultation/ or telepsychiatry/ or telepsychology/ or telerehabilitation/	9841
16	teleconferencing/	957
17	(telemedicine or telecardiology or teledermatology or telediagnosis or telediagnoses or telehealth or telemonitoring or telenephrology or teleneurology or telepsychology or teleradiology or teleradiotherap* or telesurger* or teletherap* or videoconsult*).tw,id.	5266
18	("tele medicine" or "tele cardiology" or "tele dermatology" or "tele diagnosis" or "tele diagnoses" or "tele monitoring" or "tele nephrology" or "tele neurology" or "tele psychology" or "tele radiology" or "tele radiotherap*" or "tele surger*" or "tele therap*" or "video consult*").tw,id.	161
19	("e-health" or ehealth or "tele health").tw,id.	2498
20	(telenurs* or "tele nurs*").tw,id.	94
21	online therapy/	3540
22	("cyber consult*" or cyberconsult* or econsult* or "e−consult*" or "email based consult*" or "internet consult*" or "internet based consult*" or "online consult*" or "tele consult*" or "telephone based consult*" or "telephone consult*" or "virtual consult*" or "web consult*" or "webbased consult*" or "web based consult*").tw,id.	394
23	("e−rehabilitation" or remote rehabilitation or tele rehabilitation or virtual rehabilitation).tw,id.	105
24	(computerised medical record system* or computerized medical record system*).tw,id.	2
25	electronic health records/	1022
26	(electronic health record* or computerised patient record* or computerized patient record* or electronic medical record* or electronic patient record* or ehr or emr).tw,id.	5381
27	(smart card or smart cards).tw,id.	54
28	medical order entry system*.tw,id.	2
29	(hospital information system* or patient health record information system*).tw,id.	101
30	ambulatory care information system*.tw,id.	0

31	("e−prescribing" or "e−prescription*" or electronic prescription*).tw,id.	61
32	("c.p.o.e. system" or computer* order entry or computer* physician order entry or computer* provider order entry or computer* order entry or computer* prescriber order entry or computer* prescribing order entry or computer* prescription order entry or "cpoe").tw,id.	90
33	(operating room information system* or operating room information management system*).tw,id.	0
34	(bedside computing or bedside technolog* or point of care system* or point of care technolog* or point of care information system*).tw,id.	33
35	bedside information system*.tw,id.	0
36	information systems/	5783
37	(management information system* or management information).tw,id.	809
38	clinical pharmacy information system*.tw,id.	2
39	(database management system* or database management tool or database management tools or database manager system* or database managing system* or data base manager system* or data base management software or data base management system* or data base management tool or data base management tools).tw,id.	94
40	decision support systems/	3533
41	decision support system*.tw,id.	2127
42	mobile applications/	1617
43	(mobile app or mobile apps or mobile application* or mobile health app or mobile health apps or mobile health application* or portable software app or portable software apps or portable software application* or tablet app or tablet apps or tablet application*).tw,id.	2453
44	mobile devices/ or tablet computers/ or mobile phones/	8384
45	(cell phone or cell phones or cellphone or cellphones or cellular phone or cellular phones or cellular telephone or cellular telephones or mobile phone or mobile phones or mobile telephone or mobile telephones).tw,id.	6659
46	smartphones/	2387
47	smartphones/ or "smartphone use"/	2707
48	(smartphone or smartphones or smart phone or smart phones).tw,id.	6173
49	(text messaging or texting).tw,id.	2303
50	videoconferencing/ or video-based interventions/	881
51	(video conference* or video conferencing or videoconferenc*).tw,id.	2407
52	(webcast* or web cast*).tw,id.	81
53	wireless technologies/	577
54	(wireless communication* or wireless technol*).tw,id.	336
55	("cell phone usage" or "cell phone utilisation" or "cell phone utilization" or "cellphone usage" or "cellphone use" or "cellphone utilisation " or "cellphone utilization" or "cell phone use" or "mobile phone usage" or "mobile phone use" or "mobile phone utilisation" or "mobile phone utilization").tw,id.	978

56	(hand held computer* or handheld computer* or palm PC or palmtop or personal data assistant* or personal digital assistant or pocket computer* or pocket sized computer*).tw,id.	774
57	internet/	30154
58	internet.tw,id.	45456
59	(internet based intervention* or online based intervention* or online intervention* or web intervention* or web based intervention*).tw,id.	2285
60	smart technolog*.tw,id.	138
61	wearable devices/	451
62	(wearable computer or wearable computers or wearable electronic device*).tw,id.	84
63	(activity tracker* or fitness tracker* or smart watch or smart watches or pedometer*).tw,id.	1148
64	("ar glasses" or "ar head mounted device" or "ar head mounted display" or "ar head worn display" or "ar headset" or "ar hud" or "arhmd" or "hmd ar" or "optical see through head mounted display" or "ost hmd" or "see through hmd" or "google glasses" or "smartglasses" or "smart glasses").tw,id.	55
65	artificial intelligence/	9399
66	(artificial intelligence or machine intelligence).tw,id.	6490
67	computer heuristic*.tw,id.	3
68	expert systems/	5891
69	(expert system* or knowledge based system*).tw,id.	2360
70	fuzzy logic/	1707
71	(fuzzy logic or fuzzy model or fuzzy models).tw,id.	936
72	machine learning/	10005
73	(machine learning or learning machine*).tw,id.	9762
74	(deep learning or hierarchical learning).tw,id.	2438
75	unsupervised machine learning.tw,id.	104
76	natural language processing/	714
77	natural language processing.tw,id.	1461
78	artificial neural networks/ or deep neural networks/	4760
79	("ann approach*" or "ann model" or "ann method*" or "ann training" or artificial neural network or computer neural network or computer neural networks).tw,id.	1084
80	robotics/ or avatars/ or social robotics/	7459
81	(robotics or nanorobotics or robot or robots).tw,id.	8413
82	(biomedical technology or bio medical technology).tw,id.	68
83	informatics.tw,id.	1697
84	(clinical informatics or clinical information science or clinical information technology or health informatics or health information science or health information technology or medical computer science or medical information science or medical informatics or medical information technology).tw,id.	1232
85	medical informatics.tw,id.	230
86	nursing informatics.tw,id.	84

87	health information exchange.tw,id.	126
88	(decision support system* or decision support techniques).tw,id.	2174
89	computer assisted diagnosis/	1617
90	(automatic diagnosis or computer assisted diagnosis or computer diagnosis or automatic diagnoses or computer assisted diagnoses or computer diagnoses).tw,id.	157
91	computer assisted image interpretation.tw,id.	0
92	computer assisted radiographic image interpretation*.tw,id.	0
93	computer assisted therapy/	1173
94	computer assisted therap*.tw,id.	109
95	computer assisted drug therap*.tw,id.	0
96	clinical decision support system*.tw,id.	244
97	information systems/	5783
98	(information system or information systems).tw,id.	8685
99	(online system or online systems).tw,id.	221
100	(computer interface* or computer user interface*).tw,id.	2630
101	social media/ or online social networks/	18496
102	(social media or social medium or Facebook or Flickr or Instagram or LinkedIn or MySpace or Pinterest or Reddit or "Sina Weibo" or Snapchat or online social network* or social networking platform* or social networking site* or social networking website* or social platform* or TikTok or Tumblr or Twitter or "web 2.0" or "web 2.0s" or WeChat or WhatsApp or YouTube).tw,id.	26056
103	virtual reality/ or augmented reality/	10215
104	(virtual reality or virtual realities).tw,id.	7569
105	(augmented realities or augmented reality or mixed realities or mixed reality).tw,id.	1290
106	(hologram* or holograph*).tw,id.	436
107	("3d printing*" or "3 d printing*" or "3 dimensional printing*" or "three dimensional printing*").tw,id.	136
108	(chatbot or chatbots or "chat bot" or "chat bots").tw,id.	205
109	"virtual care".tw,id.	62
110	(closed loop medicines system* or closed loop medication system* or closed loop medicines process* or closed loop medication process*).tw,id.	0
111	(bedside station or bedside stations or bed side station or bed side stations or bedside terminal or bedside terminals or bed side terminal or bed side terminals).tw,id.	1
112	predictive analytic?.tw,id.	191
113	or/15-112	180114
114	14 and 113	23753
115	interpersonal interaction/	32891
116	(nurse* patient relation* or patient nurs* relation* or professional* patient relation* or patient professional* relation* or doctor patient relation* or patient doctor relation* or physician patient relation* or patient physician relation* or patient healthcare professional relation* or patient health care professional relation*).tw,id.	5003
117	(interpersonal relation* or "inter personal relation*" or social relation*).tw,id.	39009

118	(interpersonal care or "inter personal care").tw,id.	65
119	(interpersonal interaction? or "inter personal interaction?").tw,id.	3848
120	sympathy/	3373
121	(caring or compassion* or empathy or empathetic or sympathy or sympathetic).tw,id.	81538
122	rapport.tw,id.	5720
123	humanism/	2241
124	humanistic care.tw,id.	41
125	Patient centered care/	385
126	(patient centered care or patient centered nursing or patient centred care or patient centred nursing or patient focused care).tw,id.	2631
127	interpersonal communication/	15065
128	(interpersonal communication* or "inter personal communication*").tw,id.	3969
129	professionalism/	3996
130	professionalism.tw,id.	5252
131	etiquette.tw,id.	537
132	(webside manner? or web side manner? or virtual manner?).tw,id.	6
133	(bedside manner? or bed side manner?).tw,id.	69
134	communication skills/	7625
135	(communication skill? or communication technique?).tw,id.	10952
136	(relation* adj (center* or centr*) adj (care or caring)).tw,id.	134
137	or/115-136	196736
138	114 and 137	598
139	limit 138 to english language	586
140	limit 139 to yr="2017 -Current"	179
141	limit 140 to "0110 peer-reviewed journal"	134

Database: Cumulative Index to Nursing and Allied Health (CINAHL)
Search strategy:

#	Query	Limiters/Expanders	Results
S1	(MH "Computer User Training")	Expanders - Apply equivalent subjects<br>Search modes - Boolean/Phrase	794
S2	TI (computer user training*) OR AB (computer user training*)	Expanders - Apply equivalent subjects<br>Search modes - Boolean/Phrase	0
S3	(MH "Computer Assisted Instruction")	Expanders - Apply equivalent subjects<br>Search modes - Boolean/Phrase	8,132

S4	TI (computer assisted instruction* or computerised programmed instruction* or computerised self instruction program* or computerized programmed instruction* or computerized self instruction program*) OR AB (computer assisted instruction* or computerised programmed instruction* or computerised self instruction program* or computerized programmed instruction* or computerized self instruction program*)	Expanders - Apply equivalent subjects<br>Search modes - Boolean/Phrase	362
S5	(MH "Webcasts")	Expanders - Apply equivalent subjects<br>Search modes - Boolean/Phrase	702
S6	TI (interactive tutorial or interactive tutorials or webcast* or "web cast*") OR AB (interactive tutorial or interactive tutorials or webcast* or "web cast*")	Expanders - Apply equivalent subjects<br>Search modes - Boolean/Phrase	295
S7	(MH "Staff Development")	Expanders - Apply equivalent subjects<br>Search modes - Boolean/Phrase	29,170
S8	TI (inservice or "in service" or "on the job training" or "training on the job") OR AB (inservice or "in service" or "on the job training" or "training on the job")	Expanders - Apply equivalent subjects<br>Search modes - Boolean/Phrase	169,471
S9	(MH "Patient Simulation") OR (MH "Simulations") OR (MH "Vignettes")	Expanders - Apply equivalent subjects<br>Search modes - Boolean/Phrase	26,548
S10	TI (interactive learning or simulation* or simulat*) OR AB (interactive learning or simulation* or simulat*)	Expanders - Apply equivalent subjects<br>Search modes - Boolean/Phrase	63,431
S11	(MH "Education, Post-RN") OR (MH "Education, Continuing") OR (MH "Education, Nursing, Continuing") OR (MH "Health Personnel/ED")	Expanders - Apply equivalent subjects<br>Search modes - Boolean/Phrase	37,529
S12	TI (continuing education or continuing nursing education or "post basic nursing education" or "post registration nursing education") OR AB (continuing education or continuing nursing education or "post basic nursing education" or "post registration nursing education")	Expanders - Apply equivalent subjects<br>Search modes - Boolean/Phrase	14,724

S13	S1 OR S2 OR S3 OR S4 OR S5 OR S6 OR S7 OR S8 OR S9 OR S10 OR S11 OR S12	Expanders - Apply equivalent subjects<br>Search modes - Boolean/Phrase	319,218
S14	(MH "Telemedicine") OR (MH "Telepathology") OR (MH "Teleradiology") OR (MH "Telenutrition")	Expanders - Apply equivalent subjects<br>Search modes - Boolean/Phrase	15,293
S15	TI (telemedicine or telecardiology or teledermatology or telediagnosis or telediagnoses or telehealth or telemonitoring or telenephrology or teleneurology or telepsychology or teleradiology or teleradiotherap* or telesurger* or teletherap* or videoconsult*) OR AB (telemedicine or telecardiology or teledermatology or telediagnosis or telediagnoses or telehealth or telemonitoring or telenephrology or teleneurology or telepsychology or teleradiology or teleradiotherap* or telesurger* or teletherap* or videoconsult*)	Expanders - Apply equivalent subjects<br>Search modes - Boolean/Phrase	14,334
S16	TI ("tele medicine" or "tele cardiology" or "tele dermatology" or "tele diagnosis" or "tele diagnoses" or "tele monitoring" or "tele nephrology" or "tele neurology" or "tele psychology" or "tele radiology" or "tele radiotherap*" or "tele surger*" or "tele therap*" or "video consult*") OR AB ("tele medicine" or "tele cardiology" or "tele dermatology" or "tele diagnosis" or "tele diagnoses" or "tele monitoring" or "tele nephrology" or "tele neurology" or "tele psychology" or "tele radiology" or "tele radiotherap*" or "tele surger*" or "tele therap*" or "video consult*")	Expanders - Apply equivalent subjects<br>Search modes - Boolean/Phrase	414
S17	(MH "Telehealth")	Expanders - Apply equivalent subjects<br>Search modes - Boolean/Phrase	11,615
S18	TI ("e-health" or ehealth or "tele health") OR AB ("e-health" or ehealth or "tele health")	Expanders - Apply equivalent subjects<br>Search modes - Boolean/Phrase	4,278
S19	(MH "Telenursing")	Expanders - Apply equivalent subjects<br>Search modes - Boolean/Phrase	2,244

S20	TI (telenurs* or "tele nurs*") OR AB (telenurs* or "tele nurs*")	Expanders - Apply equivalent subjects<br>Search modes - Boolean/Phrase	260
S21	(MH "Remote Consultation")	Expanders - Apply equivalent subjects<br>Search modes - Boolean/Phrase	2,725
S22	TI ("cyber consult*" or cyberconsult* or econsult* or "e−consult*" or "email based consult*" or "internet consult*" or "internet based consult*" or "online consult*" or "tele consult*" or "telephone based consult*" or "telephone consult*" or "virtual consult*" or "web consult*" or "webbased consult*" or "web based consult*") OR AB ("cyber consult*" or cyberconsult* or econsult* or "e−consult*" or "email based consult*" or "internet consult*" or "internet based consult*" or "online consult*" or "tele consult*" or "telephone based consult*" or "telephone consult*" or "virtual consult*" or "web consult*" or "webbased consult*" or "web based consult*")	Expanders - Apply equivalent subjects<br>Search modes - Boolean/Phrase	1,211
S23	(MH "Telerehabilitation")	Expanders - Apply equivalent subjects<br>Search modes - Boolean/Phrase	399
S24	TI ("e−rehabilitation" or remote rehabilitation or tele rehabilitation or virtual rehabilitation) OR AB ("e−rehabilitation" or remote rehabilitation or tele rehabilitation or virtual rehabilitation)	Expanders - Apply equivalent subjects<br>Search modes - Boolean/Phrase	237
S25	TI (computerised medical record system* or computerized medical record system*) OR AB (computerised medical record system* or computerized medical record system*)	Expanders - Apply equivalent subjects<br>Search modes - Boolean/Phrase	33
S26	(MH "Electronic Health Records")	Expanders - Apply equivalent subjects<br>Search modes - Boolean/Phrase	27,894
S27	TI (electronic health record* or computerised patient record* or computerized patient record* or electronic medical record* or electronic patient record* or ehr or emr) OR AB (electronic	Expanders - Apply equivalent subjects<br>Search modes - Boolean/Phrase	30,532

	health record* or computerised patient record* or computerized patient record* or electronic medical record* or electronic patient record* or ehr or emr)		
S28	(MH "Smart Cards")	Expanders - Apply equivalent subjects<br>Search modes - Boolean/Phrase	170
S29	TI (smart card or smart cards) OR AB (smart card or smart cards)	Expanders - Apply equivalent subjects<br>Search modes - Boolean/Phrase	134
S30	TI (medical order entry system*) OR AB (medical order entry system*)	Expanders - Apply equivalent subjects<br>Search modes - Boolean/Phrase	2
S31	(MH "Hospital Information Systems")	Expanders - Apply equivalent subjects<br>Search modes - Boolean/Phrase	3,363
S32	TI (hospital information system* or patient health record information system*) OR AB (hospital information system* or patient health record information system*)	Expanders - Apply equivalent subjects<br>Search modes - Boolean/Phrase	915
S33	(MH "Ambulatory Care Information Systems")	Expanders - Apply equivalent subjects<br>Search modes - Boolean/Phrase	316
S34	TI (ambulatory care information system*) OR AB (ambulatory care information system*)	Expanders - Apply equivalent subjects<br>Search modes - Boolean/Phrase	4
S35	TI ("e−prescribing" or "e−prescription*" or electronic prescription*) OR AB ("e−prescribing" or "e−prescription*" or electronic prescription*)	Expanders - Apply equivalent subjects<br>Search modes - Boolean/Phrase	782
S36	(MH "Electronic Order Entry")	Expanders - Apply equivalent subjects<br>Search modes - Boolean/Phrase	3,443
S37	TI ("c.p.o.e. system" or computer* order entry or computer* physician order entry or computer* provider order entry or computer* order entry or computer* prescriber order entry or computer* prescribing order entry or computer* prescription order entry or "cpoe") OR AB ("c.p.o.e. system" or computer* order entry or computer* physician order entry or	Expanders - Apply equivalent subjects<br>Search modes - Boolean/Phrase	1,364

	computer* provider order entry or computer* order entry or computer* prescriber order entry or computer* prescribing order entry or computer* prescription order entry or "cpoe")		
S38	(MH "Operating Room Information Systems")	Expanders - Apply equivalent subjects<br>Search modes - Boolean/Phrase	415
S39	TI (operating room information system* or operating room information management system*) OR AB (operating room information system* or operating room information management system*)	Expanders - Apply equivalent subjects<br>Search modes - Boolean/Phrase	8
S40	(MH "Clinical Information Systems")	Expanders - Apply equivalent subjects<br>Search modes - Boolean/Phrase	7,280
S41	TI (bedside computing or bedside technolog* or point of care system* or point of care technolog* or point of care information system*) OR AB (bedside computing or bedside technolog* or point of care system* or point of care technolog* or point of care information system*)	Expanders - Apply equivalent subjects<br>Search modes - Boolean/Phrase	222
S42	TI (clinical information system*) OR AB (clinical information system*)	Expanders - Apply equivalent subjects<br>Search modes - Boolean/Phrase	810
S43	(MH "Management Information Systems")	Expanders - Apply equivalent subjects<br>Search modes - Boolean/Phrase	1,811
S44	TI (management information system* or management information) OR AB (management information system* or management information)	Expanders - Apply equivalent subjects<br>Search modes - Boolean/Phrase	912
S45	(MH "Clinical Pharmacy Information Systems")	Expanders - Apply equivalent subjects<br>Search modes - Boolean/Phrase	1,177
S46	TI (clinical pharmacy information system*) OR AB (clinical pharmacy information system*)	Expanders - Apply equivalent subjects<br>Search modes - Boolean/Phrase	1
S47	TI (database management system* or database management tool or database management tools or database manager	Expanders - Apply equivalent subjects<br>Search modes - Boolean/Phrase	90

	system* or database managing system* or data base manager system* or data base management software or data base management system* or data base management tool or data base management tools) OR AB (database management system* or database management tool or database management tools or database manager system* or database managing system* or data base manager system* or data base management software or data base management system* or data base management tool or data base management tools)		
S48	(MH "Decision Support Systems, Management")	Expanders - Apply equivalent subjects<br>Search modes - Boolean/Phrase	491
S49	TI (decision support system*) OR AB (decision support system*)	Expanders - Apply equivalent subjects<br>Search modes - Boolean/Phrase	2,588
S50	(MH "Mobile Applications")	Expanders - Apply equivalent subjects<br>Search modes - Boolean/Phrase	10,266
S51	TI (mobile app or mobile apps or mobile application* or mobile health app or mobile health apps or mobile health application* or portable software app or portable software apps or portable software application* or tablet app or tablet apps or tablet application*) OR AB (mobile app or mobile apps or mobile application* or mobile health app or mobile health apps or mobile health application* or portable software app or portable software apps or portable software application* or tablet app or tablet apps or tablet application*)	Expanders - Apply equivalent subjects<br>Search modes - Boolean/Phrase	4,197
S52	(MH "Cellular Phone")	Expanders - Apply equivalent subjects<br>Search modes - Boolean/Phrase	2,111
S53	TI (cell phone or cell phones or cellphone or cellphones or cellular phone or cellular phones or cellular telephone or cellular telephones or mobile phone or mobile	Expanders - Apply equivalent subjects<br>Search modes - Boolean/Phrase	6,295

	phones or mobile telephone or mobile telephones) OR AB (cell phone or cell phones or cellphone or cellphones or cellular phone or cellular phones or cellular telephone or cellular telephones or mobile phone or mobile phones or mobile telephone or mobile telephones)		
S54	(MH "Smartphone")	Expanders - Apply equivalent subjects<br>Search modes - Boolean/Phrase	3,525
S55	TI (smartphone or smartphones or smart phone or smart phones) OR AB (smartphone or smartphones or smart phone or smart phones)	Expanders - Apply equivalent subjects<br>Search modes - Boolean/Phrase	8,260
S56	(MH "Text Messaging")	Expanders - Apply equivalent subjects<br>Search modes - Boolean/Phrase	3,700
S57	TI (text messaging or texting) OR AB (text messaging or texting)	Expanders - Apply equivalent subjects<br>Search modes - Boolean/Phrase	2,360
S58	(MH "Videoconferencing")	Expanders - Apply equivalent subjects<br>Search modes - Boolean/Phrase	2,468
S59	TI (video conference* or video conferencing or videoconferenc*) OR AB (video conference* or video conferencing or videoconferenc*)	Expanders - Apply equivalent subjects<br>Search modes - Boolean/Phrase	2,263
S60	(MH "Webcasts")	Expanders - Apply equivalent subjects<br>Search modes - Boolean/Phrase	702
S61	TI (webcast* or web cast*) OR AB (webcast* or web cast*)	Expanders - Apply equivalent subjects<br>Search modes - Boolean/Phrase	258
S62	TI (wireless communication* or wireless technol*) OR AB (wireless communication* or wireless technol*)	Expanders - Apply equivalent subjects<br>Search modes - Boolean/Phrase	490
S63	(MH "Computers, Hand-Held")	Expanders - Apply equivalent subjects<br>Search modes - Boolean/Phrase	4,632

S64	TI (hand held computer* or handheld computer* or palm PC or palmtop or personal data assistant* or personal digital assistant or pocket computer* or pocket sized computer*) OR AB (hand held computer* or handheld computer* or palm PC or palmtop or personal data assistant* or personal digital assistant or pocket computer* or pocket sized computer*)	Expanders - Apply equivalent subjects<br>Search modes - Boolean/Phrase	1,049
S65	(MH "Internet")	Expanders - Apply equivalent subjects<br>Search modes - Boolean/Phrase	53,031
S66	TI internet OR AB internet	Expanders - Apply equivalent subjects<br>Search modes - Boolean/Phrase	33,998
S67	(MH "Internet-Based Intervention")	Expanders - Apply equivalent subjects<br>Search modes - Boolean/Phrase	340
S68	TI (internet based intervention* or online based intervention* or online intervention* or web intervention* or web based intervention*) OR AB (internet based intervention* or online based intervention* or online intervention* or web intervention* or web based intervention*)	Expanders - Apply equivalent subjects<br>Search modes - Boolean/Phrase	1,747
S69	TI (smart technolog*) OR AB (smart technolog*)	Expanders - Apply equivalent subjects<br>Search modes - Boolean/Phrase	153
S70	TI (wearable computer or wearable computers or wearable electronic device*) OR AB (wearable computer or wearable computers or wearable electronic device*)	Expanders - Apply equivalent subjects<br>Search modes - Boolean/Phrase	37
S71	(MH "Fitness Trackers")	Expanders - Apply equivalent subjects<br>Search modes - Boolean/Phrase	278
S72	TI (activity tracker* or fitness tracker* or smart watch or smart watches or pedometer*) OR AB (activity tracker* or fitness tracker* or smart watch or smart watches or pedometer*)	Expanders - Apply equivalent subjects<br>Search modes - Boolean/Phrase	2,358

S73	(MH "Smart Glasses")	Expanders - Apply equivalent subjects<br>Search modes - Boolean/Phrase	19
S74	TI ("ar glasses" or "ar head mounted device" or "ar head mounted display" or "ar head worn display" or "ar headset" or "ar hud" or "arhmd" or "hmd ar" or "optical see through head mounted display" or "ost hmd" or "see through hmd" or "google glasses" or "smartglasses" or "smart glasses") OR AB ("ar glasses" or "ar head mounted device" or "ar head mounted display" or "ar head worn display" or "ar headset" or "ar hud" or "arhmd" or "hmd ar" or "optical see through head mounted display" or "ost hmd" or "see through hmd" or "google glasses" or "smartglasses" or "smart glasses")	Expanders - Apply equivalent subjects<br>Search modes - Boolean/Phrase	79
S75	(MH "Artificial Intelligence")	Expanders - Apply equivalent subjects<br>Search modes - Boolean/Phrase	6,510
S76	TI (artificial intelligence or machine intelligence) OR AB (artificial intelligence or machine intelligence)	Expanders - Apply equivalent subjects<br>Search modes - Boolean/Phrase	5,264
S77	TI (computer heuristic*) OR AB (computer heuristic*)	Expanders - Apply equivalent subjects<br>Search modes - Boolean/Phrase	0
S78	(MH "Expert Systems")	Expanders - Apply equivalent subjects<br>Search modes - Boolean/Phrase	534
S79	TI (expert system* or knowledge based system*) OR AB (expert system* or knowledge based system*)	Expanders - Apply equivalent subjects<br>Search modes - Boolean/Phrase	556
S80	TI (fuzzy logic or fuzzy model or fuzzy models) OR AB (fuzzy logic or fuzzy model or fuzzy models)	Expanders - Apply equivalent subjects<br>Search modes - Boolean/Phrase	302
S81	(MH "Machine Learning")	Expanders - Apply equivalent subjects<br>Search modes - Boolean/Phrase	2,826
S82	TI (machine learning or learning machine*) OR AB (machine learning or learning machine*)	Expanders - Apply equivalent subjects<br>Search modes - Boolean/Phrase	9,069

S83	(MH "Deep Learning")	Expanders - Apply equivalent subjects<br>Search modes - Boolean/Phrase	870
S84	TI (deep learning or hierarchical learning) OR AB (deep learning or hierarchical learning)	Expanders - Apply equivalent subjects<br>Search modes - Boolean/Phrase	3,280
S85	TI (unsupervised machine learning) OR AB (unsupervised machine learning)	Expanders - Apply equivalent subjects<br>Search modes - Boolean/Phrase	128
S86	(MH "Natural Language Processing")	Expanders - Apply equivalent subjects<br>Search modes - Boolean/Phrase	2,428
S87	TI (natural language processing) OR AB (natural language processing)	Expanders - Apply equivalent subjects<br>Search modes - Boolean/Phrase	1,653
S88	(MH "Neural Networks (Computer)")	Expanders - Apply equivalent subjects<br>Search modes - Boolean/Phrase	2,928
S89	TI ("ann approach*" or "ann model" or "ann method*" or "ann training" or artificial neural network or computer neural network or computer neural networks) OR AB ("ann approach*" or "ann model" or "ann method*" or "ann training" or artificial neural network or computer neural network or computer neural networks)	Expanders - Apply equivalent subjects<br>Search modes - Boolean/Phrase	1,357
S90	(MH "Robotics")	Expanders - Apply equivalent subjects<br>Search modes - Boolean/Phrase	8,524
S91	TI (robotics or nanorobotics or robot or robots) OR AB (robotics or nanorobotics or robot or robots)	Expanders - Apply equivalent subjects<br>Search modes - Boolean/Phrase	14,108
S92	TI (biomedical technology or bio medical technology) OR AB (biomedical technology or bio medical technology)	Expanders - Apply equivalent subjects<br>Search modes - Boolean/Phrase	124
S93	(MH "Informatics")	Expanders - Apply equivalent subjects<br>Search modes - Boolean/Phrase	1,354
S94	TI informatics OR AB informatics	Expanders - Apply equivalent subjects<br>Search modes - Boolean/Phrase	11,072

S95	(MH "Medical Informatics") OR (MH "Nursing Informatics") OR (MH "Health Informatics")	Expanders - Apply equivalent subjects<br>Search modes - Boolean/Phrase	12,609
S96	TI (clinical informatics or clinical information science or clinical information technology or health informatics or health information science or health information technology or medical computer science or medical information science or medical informatics or medical information technology) OR AB (clinical informatics or clinical information science or clinical information technology or health informatics or health information science or health information technology or medical computer science or medical information science or medical informatics or medical information technology)	Expanders - Apply equivalent subjects<br>Search modes - Boolean/Phrase	8,048
S97	(MH "Electronic Data Interchange")	Expanders - Apply equivalent subjects<br>Search modes - Boolean/Phrase	3,851
S98	TI (health information exchange) OR AB (health information exchange)	Expanders - Apply equivalent subjects<br>Search modes - Boolean/Phrase	1,023
S99	(MH "Decision Making, Computer Assisted")	Expanders - Apply equivalent subjects<br>Search modes - Boolean/Phrase	1,367
S100	TI (decision support system* or decision support techniques) OR AB (decision support system* or decision support techniques)	Expanders - Apply equivalent subjects<br>Search modes - Boolean/Phrase	2,592
S101	(MH "Diagnosis, Computer Assisted")	Expanders - Apply equivalent subjects<br>Search modes - Boolean/Phrase	4,721
S102	TI (automatic diagnosis or computer assisted diagnosis or computer diagnosis or automatic diagnoses or computer assisted diagnoses or computer diagnoses) OR AB (automatic diagnosis or computer assisted diagnosis or computer diagnosis or automatic diagnoses or computer assisted diagnoses or computer diagnoses)	Expanders - Apply equivalent subjects<br>Search modes - Boolean/Phrase	165

S103	(MH "Image Interpretation, Computer Assisted")	Expanders - Apply equivalent subjects<br>Search modes - Boolean/Phrase	9,408
S104	TI (computer assisted image interpretation) OR AB (computer assisted image interpretation)	Expanders - Apply equivalent subjects<br>Search modes - Boolean/Phrase	1
S105	(MH "Radiographic Image Interpretation, Computer-Assisted")	Expanders - Apply equivalent subjects<br>Search modes - Boolean/Phrase	4,496
S106	TI (computer assisted radiographic image interpretation*) OR AB (computer assisted radiographic image interpretation*)	Expanders - Apply equivalent subjects<br>Search modes - Boolean/Phrase	0
S107	(MH "Therapy, Computer Assisted") OR (MH "Drug Therapy, Computer Assisted")	Expanders - Apply equivalent subjects<br>Search modes - Boolean/Phrase	5,949
S108	TI (computer assisted therap*) OR AB (computer assisted therap*)	Expanders - Apply equivalent subjects<br>Search modes - Boolean/Phrase	36
S109	TI (computer assisted drug therap*) OR AB (computer assisted drug therap*)	Expanders - Apply equivalent subjects<br>Search modes - Boolean/Phrase	4
S110	(MH "Decision Support Systems, Clinical")	Expanders - Apply equivalent subjects<br>Search modes - Boolean/Phrase	5,994
S111	TI (clinical decision support system*) OR AB (clinical decision support system*)	Expanders - Apply equivalent subjects<br>Search modes - Boolean/Phrase	1,226
S112	(MH "Information Systems")	Expanders - Apply equivalent subjects<br>Search modes - Boolean/Phrase	4,876
S113	TI (information system or information systems) OR AB (information system or information systems)	Expanders - Apply equivalent subjects<br>Search modes - Boolean/Phrase	13,874
S114	(MH "Online Systems")	Expanders - Apply equivalent subjects<br>Search modes - Boolean/Phrase	1,864
S115	TI (online system or online systems) OR AB (online system or online systems)	Expanders - Apply equivalent subjects<br>Search modes - Boolean/Phrase	248

S116	(MH "User-Computer Interface")	Expanders - Apply equivalent subjects<br>Search modes - Boolean/Phrase	11,032
S117	TI (computer interface* or computer user interface*) OR AB (computer interface* or computer user interface*)	Expanders - Apply equivalent subjects<br>Search modes - Boolean/Phrase	753
S118	(MH "Social Media") OR (MH "Facebook") OR (MH "Twitter")	Expanders - Apply equivalent subjects<br>Search modes - Boolean/Phrase	19,346
S119	(MH "Online Social Networking")	Expanders - Apply equivalent subjects<br>Search modes - Boolean/Phrase	493
S120	TI (social media or social medium or Facebook or Flickr or Instagram or LinkedIn or MySpace or Pinterest or Reddit or "Sina Weibo" or Snapchat or online social network* or social networking platform* or social networking site* or social networking website* or social platform* or TikTok or Tumblr or Twitter or "web 2.0" or "web 2.0s" or WeChat or WhatsApp or YouTube)	Expanders - Apply equivalent subjects<br>Search modes - Boolean/Phrase	12,658
S121	AB (social media or social medium or Facebook or Flickr or Instagram or LinkedIn or MySpace or Pinterest or Reddit or "Sina Weibo" or Snapchat or online social network* or social networking platform* or social networking site* or social networking website* or social platform* or TikTok or Tumblr or Twitter or "web 2.0" or "web 2.0s" or WeChat or WhatsApp or YouTube)	Expanders - Apply equivalent subjects<br>Search modes - Boolean/Phrase	17,394
S122	(MH "Virtual Reality")	Expanders - Apply equivalent subjects<br>Search modes - Boolean/Phrase	6,267
S123	TI (virtual reality or virtual realities) OR AB (virtual reality or virtual realities)	Expanders - Apply equivalent subjects<br>Search modes - Boolean/Phrase	5,058
S124	(MH "Augmented Reality")	Expanders - Apply equivalent subjects<br>Search modes - Boolean/Phrase	293
S125	TI (augmented realities or augmented reality or mixed realities or mixed reality)	Expanders - Apply equivalent subjects<br>Search modes - Boolean/Phrase	982

	OR AB (augmented realities or augmented reality or mixed realities or mixed reality)		
S126	(MH "Holography")	Expanders - Apply equivalent subjects<br>Search modes - Boolean/Phrase	100
S127	TI (hologram* or holograph*) OR AB (hologram* or holograph*)	Expanders - Apply equivalent subjects<br>Search modes - Boolean/Phrase	216
S128	(MH "Printing, Three-Dimensional")	Expanders - Apply equivalent subjects<br>Search modes - Boolean/Phrase	2,192
S129	TI ("3d printing*" or "3 d printing*" or "3 dimensional printing*" or "three dimensional printing*") OR AB ("3d printing*" or "3 d printing*" or "3 dimensional printing*" or "three dimensional printing*")	Expanders - Apply equivalent subjects<br>Search modes - Boolean/Phrase	1,280
S130	TI (chatbot or chatbots or "chat bot" or "chat bots") OR AB (chatbot or chatbots or "chat bot" or "chat bots")	Expanders - Apply equivalent subjects<br>Search modes - Boolean/Phrase	247
S131	TI "virtual care" OR AB "virtual care"	Expanders - Apply equivalent subjects<br>Search modes - Boolean/Phrase	352
S132	TI (closed loop medicines system* or closed loop medication system* or closed loop medicines process* or closed loop medication process*) OR AB (closed loop medicines system* or closed loop medication system* or closed loop medicines process* or closed loop medication process*)	Expanders - Apply equivalent subjects<br>Search modes - Boolean/Phrase	1
S133	TI (bedside station or bedside stations or bed side station or bed side stations or bedside terminal or bedside terminals or bed side terminal or bed side terminals or bedside information system*) OR AB (bedside station or bedside stations or bed side station or bed side stations or bedside terminal or bedside terminals or bed side terminal or bed side terminals or bedside information system*)	Expanders - Apply equivalent subjects<br>Search modes - Boolean/Phrase	31

S134	TI (predictive analytic OR predictive analytics) OR AB (predictive analytic OR predictive analytics)	Expanders - Apply equivalent subjects<br>Search modes - Boolean/Phrase	371
S135	S14 OR S15 OR S16 OR S17 OR S18 OR S19 OR S20 OR S21 OR S22 OR S23 OR S24 OR S25 OR S26 OR S27 OR S28 OR S29 OR S30 OR S31 OR S32 OR S33 OR S34 OR S35 OR S36 OR S37 OR S38 OR S39 OR S40 OR S41 OR S42 OR S43 OR S44 OR S45 OR S46 OR S47 OR S48 OR S49 OR S50 OR S51 OR S52 OR S53 OR S54 OR S55 OR S56 OR S57 OR S58 OR S59 OR S60 OR S61 OR S62 OR S63 OR S64 OR S65 OR S66 OR S67 OR S68 OR S69 OR S70 OR S71 OR S72 OR S73 OR S74 OR S75 OR S76 OR S77 OR S78 OR S79 OR S80 OR S81 OR S82 OR S83 OR S84 OR S85 OR S86 OR S87 OR S88 OR S89 OR S90 OR S91 OR S92 OR S93 OR S94 OR S95 OR S96 OR S97 OR S98 OR S99 OR S100 OR S101 OR S102 OR S103 OR S104 OR S105 OR S106 OR S107 OR S108 OR S109 OR S110 OR S111 OR S112 OR S113 OR S114 OR S115 OR S116 OR S117 OR S118 OR S119 OR S120 OR S121 OR S122 OR S123 OR S124 OR S125 OR S126 OR S127 OR S128 OR S129 OR S130 OR S131 OR S132 OR S133 OR S134	Expanders - Apply equivalent subjects<br>Search modes - Boolean/Phrase	325,466
S136	(MH "Professional-Patient Relations") OR (MH "Nurse-Patient Relations") OR (MH "Physician-Patient Relations") OR (MH "Interpersonal Relations")	Expanders - Apply equivalent subjects<br>Search modes - Boolean/Phrase	153,688
S137	TI (nurse* patient relation* or patient nurs* relation* or professional* patient relation* or patient professional* relation* or doctor patient relation* or patient doctor relation* or physician patient relation* or patient physician relation* or patient healthcare professional relation* or patient health care professional relation*)	Expanders - Apply equivalent subjects<br>Search modes - Boolean/Phrase	1,022
S138	AB (nurse* patient relation* or patient nurs* relation* or professional* patient relation* or patient professional* relation* or doctor patient relation* or patient doctor relation* or physician patient relation* or patient physician relation* or patient	Expanders - Apply equivalent subjects<br>Search modes - Boolean/Phrase	3,864

	healthcare professional relation* or patient health care professional relation*)		
S139	TI (interpersonal relation* or "inter personal relation*" or social relation*) OR AB (interpersonal relation* or "inter personal relation*" or social relation*)	Expanders - Apply equivalent subjects<br>Search modes - Boolean/Phrase	9,662
S140	TI (interpersonal care or "inter personal care") OR AB (interpersonal care or "inter personal care")	Expanders - Apply equivalent subjects<br>Search modes - Boolean/Phrase	128
S141	TI (interpersonal interaction# or "inter personal interaction#") OR AB (interpersonal interaction# or "inter personal interaction#")	Expanders - Apply equivalent subjects<br>Search modes - Boolean/Phrase	635
S142	(MH "Empathy")	Expanders - Apply equivalent subjects<br>Search modes - Boolean/Phrase	12,004
S143	(MH "Compassion")	Expanders - Apply equivalent subjects<br>Search modes - Boolean/Phrase	2,621
S144	TI (caring or compassion* or empathy or empathetic or sympathy or sympathetic) OR AB (caring or compassion* or empathy or empathetic or sympathy or sympathetic)	Expanders - Apply equivalent subjects<br>Search modes - Boolean/Phrase	72,328
S145	(MH "Humanism")	Expanders - Apply equivalent subjects<br>Search modes - Boolean/Phrase	2,082
S146	TI "humanistic care" OR AB "humanistic care"	Expanders - Apply equivalent subjects<br>Search modes - Boolean/Phrase	162
S147	(MH "Patient Centered Care")	Expanders - Apply equivalent subjects<br>Search modes - Boolean/Phrase	33,445
S148	TI (patient centered care or patient centered nursing or patient centred care or patient centred nursing or patient focused care) OR AB (patient centered care or patient centered nursing or patient centred care or patient centred nursing or patient focused care)	Expanders - Apply equivalent subjects<br>Search modes - Boolean/Phrase	5,475

S149	TI (interpersonal communication* or "inter personal communication*") OR AB (interpersonal communication* or "inter personal communication*")	Expanders - Apply equivalent subjects<br>Search modes - Boolean/Phrase	1,098
S150	TI rapport OR AB rapport	Expanders - Apply equivalent subjects<br>Search modes - Boolean/Phrase	4,333
S151	(MH "Professionalism")	Expanders - Apply equivalent subjects<br>Search modes - Boolean/Phrase	7,638
S152	TI professionalism OR AB professionalism	Expanders - Apply equivalent subjects<br>Search modes - Boolean/Phrase	5,618
S153	TI etiquette OR AB etiquette	Expanders - Apply equivalent subjects<br>Search modes - Boolean/Phrase	594
S154	TI (webside manner# or web side manner# or virtual manner#) OR AB (webside manner# or web side manner# or virtual manner#)	Expanders - Apply equivalent subjects<br>Search modes - Boolean/Phrase	13
S155	TI (bedside manner# or bed side manner#) OR AB (bedside manner# or bed side manner#)	Expanders - Apply equivalent subjects<br>Search modes - Boolean/Phrase	167
S156	(MH "Communication") OR (MH "Communication Skills")	Expanders - Apply equivalent subjects<br>Search modes - Boolean/Phrase	96,306
S157	TI (communication skill# or communication technique#) OR AB (communication skill# or communication technique#)	Expanders - Apply equivalent subjects<br>Search modes - Boolean/Phrase	8,632
S158	TI (relation* N1 (center* or centr*) N1 (care or caring)) OR AB (relation* N1 (center* or centr*) N1 (care or caring))	Expanders - Apply equivalent subjects<br>Search modes - Boolean/Phrase	189
S159	S136 OR S137 OR S138 OR S139 OR S140 OR S141 OR S142 OR S143 OR S144 OR S145 OR S146 OR S147 OR S148 OR S149 OR S150 OR S151 OR S152 OR S153 OR S154 OR S155 OR S156 OR S157 OR S158	Expanders - Apply equivalent subjects<br>Search modes - Boolean/Phrase	342,293
S160	S13 AND S135 AND S159	Expanders - Apply equivalent subjects<br>Search modes - Boolean/Phrase	2,700

S161	S13 AND S135 AND S159	Limiters - English Language<br>Expanders - Apply equivalent subjects<br>Search modes - Boolean/Phrase	2,661
S162	S13 AND S135 AND S159	Limiters - Published Date: 20170101-20221231; English Language<br>Expanders - Apply equivalent subjects<br>Search modes - Boolean/Phrase	1,367
S163	S13 AND S135 AND S159	Limiters - Published Date: 20170101-20221231; English Language; Peer Reviewed<br>Expanders - Apply equivalent subjects<br>Search modes - Boolean/Phrase	1,237

Database: IEEE Explore
Search strategy:

Group 1 Terms Strategy

((("All Metadata":"nurse patient relation" OR "All Metadata":"patient nurse relation" OR "All Metadata":"professional patient relation" OR "All Metadata":"patient professional relation" OR "All Metadata":"nurse patient relationship" OR "All Metadata":"patient nurse relationship" OR "All Metadata":"professional patient relationship" OR "All Metadata":"patient professional relationship") OR ("All Metadata":"doctor patient relation" OR "All Metadata":"patient doctor relation" OR "All Metadata":"physician patient relation" OR "All Metadata":"patient physician relation" OR "All Metadata":"patient healthcare professional relation" OR "All Metadata":"patient health care professional relation" OR "All Metadata":"doctor patient relationship" OR "All Metadata":"patient doctor relationship" OR "All Metadata":"physician patient relationship" OR "All Metadata":"patient physician relationship" OR "All Metadata":"patient healthcare professional relationship" OR "All Metadata":"patient health care professional relationship") OR ("All Metadata":"interpersonal relation" OR "All Metadata":"interpersonal relationship" OR "All Metadata":"inter personal relation" OR "All Metadata":"inter personal relationship" OR "All Metadata":"social relation" OR "All Metadata":"social relationship") OR ("All Metadata":"interpersonal care" OR "All Metadata":"inter personal care" OR "All Metadata":"interpersonal interaction" OR "All Metadata":"inter personal interaction") OR ("All Metadata":caring OR "All Metadata":compassion OR "All Metadata":compassionate OR "All Metadata":empathy OR "All Metadata":empathetic OR "All Metadata":sympathy OR "All Metadata":sympathetic OR "All Metadata":rapport) OR ("All Metadata":"humanistic care" OR "All Metadata":"patient centered care" OR "All Metadata":"patient centered nursing" OR "All Metadata":"patient centred care" OR "All Metadata":"patient centred nursing" OR "All Metadata":"patient focused care") OR ("All Metadata":"interpersonal communication" OR "All Metadata":"inter personal communication" OR "All Metadata": professionalism OR "All

Metadata":etiquette OR "All Metadata":"webside manner" OR "All Metadata":"web side manner" OR "All Metadata":"virtual manner" OR "All Metadata":"bedside manner" OR "All Metadata":"bed side manner" OR "All Metadata":"communication skill" OR "All Metadata":"communication technique") OR ("All Metadata":relation ONEAR/2 care OR "All Metadata":relation ONEAR/2 caring OR "All Metadata":relation ONEAR/2 care OR "All Metadata":relation ONEAR/2 caring) AND ("All Metadata":computer* ONEAR/2 instruction OR "All Metadata":computer user training OR "All Metadata": interactive tutorial OR "All Metadata":webcast OR "All Metadata":web cast OR "All Metadata":webcasting OR "All Metadata":web casting OR "All Metadata":interactive learning OR "All Metadata":simulation) OR ("All Metadata":inservice OR "All Metadata":"in service" OR "All Metadata":"on the job training" OR "All Metadata":"training on the job" OR "All Metadata":"hands on") OR ("All Metadata":practical ONEAR/3 application OR "All Metadata":practical ONEAR/3 component OR "All Metadata":practical ONEAR/3 element OR "All Metadata":practical ONEAR/3 professional development OR "All Metadata":practical ONEAR/3 session) OR ("All Metadata":continuing education OR "All Metadata":continuing nursing education OR "All Metadata":post basic nursing education OR "All Metadata":post registration nursing education)) AND ("All Metadata":telemedicine OR "All Metadata":telecardiology OR "All Metadata":teledermatology OR "All Metadata":telediagnosis OR "All Metadata":telediagnoses OR "All Metadata":telemonitoring OR "All Metadata":telenephrology OR "All Metadata":teleneurology OR "All Metadata":telepsychology OR "All Metadata":teleradiology OR "All Metadata":teleradiotherapy OR "All Metadata":telesurgery OR "All Metadata":teletherapy teletherapies OR "All Metadata":videoconsult OR "All Metadata":videoconsults OR "All Metadata":"tele medicine" OR "All Metadata":"tele cardiology" OR "All Metadata":"tele dermatology" OR "All Metadata":"tele diagnosis" OR "All Metadata":"tele diagnoses" OR "All Metadata":"tele monitoring" OR "All Metadata":"tele nephrology" OR "All Metadata":"tele neurology" OR "All Metadata":"tele psychology" OR "All Metadata":"tele radiology" OR "All Metadata":"tele radiotherapy" OR "All Metadata":"tele surgery" OR "All Metadata":"tele therapy" OR "All Metadata":telehealth OR "All Metadata":"tele health" OR "All Metadata":"e-health" OR "All Metadata":ehealth OR "All Metadata":telenurse OR "All Metadata":"tele nurse" OR "All Metadata":telenursing OR "All Metadata":"tele nursing" OR "All Metadata":"e−rehabilitation" OR "All Metadata":remote rehabilitation OR "All Metadata":"tele rehabilitation" OR "All Metadata":"virtual rehabilitation") OR ("All Metadata":"cyber consult"))

Search Within Results

nurse OR nurses OR nursing

Filters Applied:

Journals

2017 - 2022

Group 2 Terms Strategy
((("All Metadata":"nurse patient relation" OR "All Metadata":"patient nurse relation" OR "All Metadata":"professional patient relation" OR "All Metadata":"patient professional relation" OR "All Metadata":"nurse patient relationship" OR "All Metadata":"patient nurse relationship" OR "All Metadata":"professional patient relationship" OR "All Metadata":"patient professional relationship") OR

("All Metadata":"doctor patient relation" OR "All Metadata":"patient doctor relation" OR "All Metadata":"physician patient relation" OR "All Metadata":"patient physician relation" OR "All Metadata":"patient healthcare professional relation" OR "All Metadata":"patient health care professional relation" OR "All Metadata":"doctor patient relationship" OR "All Metadata":"patient doctor relationship" OR "All Metadata":"physician patient relationship" OR "All Metadata":"patient physician relationship" OR "All Metadata":"patient healthcare professional relationship" OR "All Metadata":"patient health care professional relationship") OR ("All Metadata":"interpersonal relation" OR "All Metadata":"interpersonal relationship" OR "All Metadata":"inter personal relation" OR "All Metadata":"inter personal relationship" OR "All Metadata":"social relation" OR "All Metadata":"social relationship") OR ("All Metadata":"interpersonal care" OR "All Metadata":"inter personal care" OR "All Metadata":"interpersonal interaction" OR "All Metadata":"inter personal interaction") OR ("All Metadata":caring OR "All Metadata":compassion OR "All Metadata":compassionate OR "All Metadata":empathy OR "All Metadata":empathetic OR "All Metadata":sympathy OR "All Metadata":sympathetic OR "All Metadata":rapport) OR ("All Metadata":"humanistic care" OR "All Metadata":"patient centered care" OR "All Metadata":"patient centered nursing" OR "All Metadata":"patient centred care" OR "All Metadata":"patient centred nursing" OR "All Metadata":"patient focused care") OR ("All Metadata":"interpersonal communication" OR "All Metadata":"inter personal communication" OR "All Metadata": professionalism OR "All Metadata":etiquette OR "All Metadata":"webside manner" OR "All Metadata":"web side manner" OR "All Metadata":"virtual manner" OR "All Metadata":"bedside manner" OR "All Metadata":"bed side manner" OR "All Metadata":"communication skill" OR "All Metadata":"communication technique") OR ("All Metadata":relation ONEAR/2 care OR "All Metadata":relation ONEAR/2 caring OR "All Metadata":relation ONEAR/2 care OR "All Metadata":relation ONEAR/2 caring) AND ("All Metadata":computer* ONEAR/2 instruction OR "All Metadata":computer user training OR "All Metadata": interactive tutorial OR "All Metadata":webcast OR "All Metadata":web cast OR "All Metadata":webcasting OR "All Metadata":web casting OR "All Metadata":interactive learning OR "All Metadata":simulation) OR ("All Metadata":inservice OR "All Metadata":"in service" OR "All Metadata":"on the job training" OR "All Metadata":"training on the job" OR "All Metadata":"hands on") OR ("All Metadata":practical ONEAR/3 application OR "All Metadata":practical ONEAR/3 component OR "All Metadata":practical ONEAR/3 element OR "All Metadata":practical ONEAR/3 professional development OR "All Metadata":practical ONEAR/3 session) OR ("All Metadata":continuing education OR "All Metadata":continuing nursing education OR "All Metadata":post basic nursing education OR "All Metadata":post registration nursing education)) AND ("All Metadata":"cyber consulting" OR "All Metadata":"cyber consultation" OR "All Metadata":cyberconsult OR "All Metadata":econsult OR "All Metadata":cyberconsultation OR "All Metadata":econsultation OR "All Metadata":"e consult" OR "All Metadata":"e consulting" OR "All Metadata":"e consutlation" OR "All Metadata":"email based consult" OR "All Metadata":"email based consulting" OR "All Metadata":"email based consultation" OR "All Metadata":"online consult" OR "All Metadata":"online consultation" OR "All Metadata":"tele consult" OR "All Metadata":"tele consultation" OR "All Metadata":"telephone based consult" OR "All Metadata":"telephone based consultation" OR "All Metadata":"telephone consult" OR "All Metadata":"telephone consultation" OR "All Metadata":"video consult" OR "All Metadata":"video consultation" OR "All Metadata":"virtual consult" OR "All Metadata":"virtual consultation" OR "All Metadata":"cyber consults" OR "All Metadata":"cyber consultations" OR "All Metadata":cyberconsults OR "All Metadata":econsults OR "All Metadata":cyberconsultations OR "All Metadata":econsultations OR "All Metadata":"e consults" OR "All Metadata":"e consutlations" OR "All Metadata":"email based consults" OR "All Metadata":"email based consultations" OR "All Metadata":"online consults" OR "All Metadata":"online consultations" OR "All Metadata":"tele consults" OR "All Metadata":"tele

consultations" OR "All Metadata":"telephone based consults" OR "All Metadata":"telephone based consultations" OR "All Metadata":"telephone consults" OR "All Metadata":"telephone consultations" OR "All Metadata":"video consults" OR "All Metadata":"video consultations" OR "All Metadata":"virtual consults" OR "All Metadata":"virtual consultations" OR "All Metadata":"video conference" OR "All Metadata":"video conferences" OR "All Metadata":video conferencing OR "All Metadata":videoconference OR "All Metadata":videoconferences OR "All Metadata":webcast OR "All Metadata":webcasts OR "All Metadata":webcasting OR "All Metadata":"virtual care"))

Search Within Results

nurse OR nurses OR nursing

Filters Applied:

Journals

2017 – 2022

Group 3 Terms Strategy

((("All Metadata":"nurse patient relation" OR "All Metadata":"patient nurse relation" OR "All Metadata":"professional patient relation" OR "All Metadata":"patient professional relation" OR "All Metadata":"nurse patient relationship" OR "All Metadata":"patient nurse relationship" OR "All Metadata":"professional patient relationship" OR "All Metadata":"patient professional relationship") OR ("All Metadata":"doctor patient relation" OR "All Metadata":"patient doctor relation" OR "All Metadata":"physician patient relation" OR "All Metadata":"patient physician relation" OR "All Metadata":"patient healthcare professional relation" OR "All Metadata":"patient health care professional relation" OR "All Metadata":"doctor patient relationship" OR "All Metadata":"patient doctor relationship" OR "All Metadata":"physician patient relationship" OR "All Metadata":"patient physician relationship" OR "All Metadata":"patient healthcare professional relationship" OR "All Metadata":"patient health care professional relationship") OR ("All Metadata":"interpersonal relation" OR "All Metadata":"interpersonal relationship" OR "All Metadata":"inter personal relation" OR "All Metadata":"inter personal relationship" OR "All Metadata":"social relation" OR "All Metadata":"social relationship") OR ("All Metadata":"interpersonal care" OR "All Metadata":"inter personal care" OR "All Metadata":"interpersonal interaction" OR "All Metadata":"inter personal interaction") OR ("All Metadata":caring OR "All Metadata":compassion OR "All Metadata":compassionate OR "All Metadata":empathy OR "All Metadata":empathetic OR "All Metadata":sympathy OR "All Metadata":sympathetic OR "All Metadata":rapport) OR ("All Metadata":"humanistic care" OR "All Metadata":"patient centered care" OR "All Metadata":"patient centered nursing" OR "All Metadata":"patient centred care" OR "All Metadata":"patient centred nursing" OR "All Metadata":"patient focused care") OR ("All Metadata":"interpersonal communication" OR "All Metadata":"inter personal communication" OR "All Metadata": professionalism OR "All Metadata":etiquette OR "All Metadata":"webside manner" OR "All Metadata":"web side manner" OR "All Metadata":"virtual manner" OR "All Metadata":"bedside manner" OR "All Metadata":"bed side manner" OR "All Metadata":"communication skill" OR "All Metadata":"communication technique") OR

("All Metadata":relation ONEAR/2 care OR "All Metadata":relation ONEAR/2 caring OR "All Metadata":relation ONEAR/2 care OR "All Metadata":relation ONEAR/2 caring) AND ("All Metadata":computer* ONEAR/2 instruction OR "All Metadata":"computer user training" OR "All Metadata":"interactive tutorial" OR "All Metadata":webcast OR "All Metadata":web cast OR "All Metadata":webcasting OR "All Metadata":"web casting" OR "All Metadata":"interactive learning" OR "All Metadata":simulation) OR ("All Metadata":inservice OR "All Metadata":"in service" OR "All Metadata":"on the job training" OR "All Metadata":"training on the job" OR "All Metadata":"hands on") OR ("All Metadata":practical ONEAR/3 application OR "All Metadata":practical ONEAR/3 component OR "All Metadata":practical ONEAR/3 element OR "All Metadata":practical ONEAR/3 professional development OR "All Metadata":practical ONEAR/3 session) OR ("All Metadata":"continuing education" OR "All Metadata":"continuing nursing education" OR "All Metadata":"post basic nursing education" OR "All Metadata":"post registration nursing education")) AND ("All Metadata":"medical record" OR "All Metadata":"health record" OR "All Metadata":"patient record" OR "All Metadata":ehr OR "All Metadata":emr OR "All Metadata":"medical records" OR "All Metadata":"health records" OR "All Metadata":"patient records" OR "All Metadata":ehrs OR "All Metadata":emrs OR "All Metadata":"health information" OR "All Metadata":"smart card" OR "All Metadata":"smart cards" OR "All Metadata":"order entry" OR "All Metadata":"information system" OR "All Metadata":"information systems" OR "All Metadata":"information management system" OR "All Metadata":"information management systems" OR "All Metadata":"database management" OR "All Metadata":"decision support" OR "All Metadata":"point of care" OR "All Metadata":bedside OR "All Metadata":"bedside" OR "All Metadata":"electronic prescribing" OR "All Metadata":"e prescribing" OR "All Metadata":"e prescription" OR "All Metadata":"e prescriptions" OR "All Metadata":"electronic prescription" OR "All Metadata":"electronic prescriptions" OR "All Metadata":"closed loop medicines system" OR "All Metadata":"closed loop medicines systems" OR "All Metadata":"closed loop medication system" OR "All Metadata":"closed loop medication systems" OR "All Metadata":"closed loop medicines process" OR "All Metadata":"closed loop medication process"))

Search Within Results

nurse OR nurses OR nursing

Filters Applied:

Journals

2017 – 2022

Group 4 Terms Strategy

((("All Metadata":"nurse patient relation" OR "All Metadata":"patient nurse relation" OR "All Metadata":"professional patient relation" OR "All Metadata":"patient professional relation" OR "All Metadata":"nurse patient relationship" OR "All Metadata":"patient nurse relationship" OR "All Metadata":"professional patient relationship" OR "All Metadata":"patient professional relationship") OR ("All Metadata":"doctor patient relation" OR "All Metadata":"patient doctor relation" OR "All Metadata":"physician patient relation" OR "All Metadata":"patient physician relation" OR "All Metadata":"patient healthcare professional relation" OR "All Metadata":"patient health care

professional relation" OR "All Metadata":"doctor patient relationship" OR "All Metadata":"patient doctor relationship" OR "All Metadata":"physician patient relationship" OR "All Metadata":"patient physician relationship" OR "All Metadata":"patient healthcare professional relationship" OR "All Metadata":"patient health care professional relationship") OR ("All Metadata":"interpersonal relation" OR "All Metadata":"interpersonal relationship" OR "All Metadata":"inter personal relation" OR "All Metadata":"inter personal relationship" OR "All Metadata":"social relation" OR "All Metadata":"social relationship") OR ("All Metadata":"interpersonal care" OR "All Metadata":"inter personal care" OR "All Metadata":"interpersonal interaction" OR "All Metadata":"inter personal interaction") OR ("All Metadata":caring OR "All Metadata":compassion OR "All Metadata":compassionate OR "All Metadata":empathy OR "All Metadata":empathetic OR "All Metadata":sympathy OR "All Metadata":sympathetic OR "All Metadata":rapport) OR ("All Metadata":"humanistic care" OR "All Metadata":"patient centered care" OR "All Metadata":"patient centered nursing" OR "All Metadata":"patient centred care" OR "All Metadata":"patient centred nursing" OR "All Metadata":"patient focused care") OR ("All Metadata":"interpersonal communication" OR "All Metadata":"inter personal communication" OR "All Metadata": professionalism OR "All Metadata":etiquette OR "All Metadata":"webside manner" OR "All Metadata":"web side manner" OR "All Metadata":"virtual manner" OR "All Metadata":"bedside manner" OR "All Metadata":"bed side manner" OR "All Metadata":"communication skill" OR "All Metadata":"communication technique") OR ("All Metadata":relation ONEAR/2 care OR "All Metadata":relation ONEAR/2 caring OR "All Metadata":relation ONEAR/2 care OR "All Metadata":relation ONEAR/2 caring) AND ("All Metadata":computer* ONEAR/2 instruction OR "All Metadata":"computer user training" OR "All Metadata":"interactive tutorial" OR "All Metadata":webcast OR "All Metadata":web cast OR "All Metadata":webcasting OR "All Metadata":"web casting" OR "All Metadata":"interactive learning" OR "All Metadata":simulation) OR ("All Metadata":inservice OR "All Metadata":"in service" OR "All Metadata":"on the job training" OR "All Metadata":"training on the job" OR "All Metadata":"hands on") OR ("All Metadata":practical ONEAR/3 application OR "All Metadata":practical ONEAR/3 component OR "All Metadata":practical ONEAR/3 element OR "All Metadata":practical ONEAR/3 professional development OR "All Metadata":practical ONEAR/3 session) OR ("All Metadata":"continuing education" OR "All Metadata":"continuing nursing education" OR "All Metadata":"post basic nursing education" OR "All Metadata":"post registration nursing education")) AND ("All Metadata":"mobile app" OR "All Metadata":"mobile apps" OR "All Metadata":"mobile application" OR "All Metadata":"health app" OR "All Metadata":"health apps" OR "All Metadata":"health application" OR "All Metadata":"health applications" OR "All Metadata":"portable software app" OR "All Metadata":"portable software apps" OR "All Metadata":"portable software application" OR "All Metadata":"portable software applications" OR "All Metadata":"tablet app" OR "All Metadata":"tablet apps" OR "All Metadata":"tablet application" OR "All Metadata":"tablet applications" OR "All Metadata":"cell phone" OR "All Metadata":"cell phones" OR "All Metadata":"cellphone" OR "All Metadata":"cellphones" OR "All Metadata":"cellular phone" OR "All Metadata":"cellular phones" OR "All Metadata":"cellular telephone" OR "All Metadata":"cellular telephones" OR "All Metadata":"mobile phone" OR "All Metadata":"mobile phones" OR "All Metadata":"mobile telephone" OR "All Metadata":"mobile telephones" OR "All Metadata":smartphone OR "All Metadata":smartphones OR "All Metadata":"smart phones" OR "All Metadata":"smart phones" OR "All Metadata":"text messaging" OR "All Metadata":texting))

Search Within Results

nurse OR nurses OR nursing

Filters Applied:

Journals

2017 – 2022

Group 5 Terms Strategy

((("All Metadata":"nurse patient relation" OR "All Metadata":"patient nurse relation" OR "All Metadata":"professional patient relation" OR "All Metadata":"patient professional relation" OR "All Metadata":"nurse patient relationship" OR "All Metadata":"patient nurse relationship" OR "All Metadata":"professional patient relationship" OR "All Metadata":"patient professional relationship") OR ("All Metadata":"doctor patient relation" OR "All Metadata":"patient doctor relation" OR "All Metadata":"physician patient relation" OR "All Metadata":"patient physician relation" OR "All Metadata":"patient healthcare professional relation" OR "All Metadata":"patient health care professional relation" OR "All Metadata":"doctor patient relationship" OR "All Metadata":"patient doctor relationship" OR "All Metadata":"physician patient relationship" OR "All Metadata":"patient physician relationship" OR "All Metadata":"patient healthcare professional relationship" OR "All Metadata":"patient health care professional relationship") OR ("All Metadata":"interpersonal relation" OR "All Metadata":"interpersonal relationship" OR "All Metadata":"inter personal relation" OR "All Metadata":"inter personal relationship" OR "All Metadata":"social relation" OR "All Metadata":"social relationship") OR ("All Metadata":"interpersonal care" OR "All Metadata":"inter personal care" OR "All Metadata":"interpersonal interaction" OR "All Metadata":"inter personal interaction") OR ("All Metadata":caring OR "All Metadata":compassion OR "All Metadata":compassionate OR "All Metadata":empathy OR "All Metadata":empathetic OR "All Metadata":sympathy OR "All Metadata":sympathetic OR "All Metadata":rapport) OR ("All Metadata":"humanistic care" OR "All Metadata":"patient centered care" OR "All Metadata":"patient centered nursing" OR "All Metadata":"patient centred care" OR "All Metadata":"patient centred nursing" OR "All Metadata":"patient focused care") OR ("All Metadata":"interpersonal communication" OR "All Metadata":"inter personal communication" OR "All Metadata": professionalism OR "All Metadata":etiquette OR "All Metadata":"webside manner" OR "All Metadata":"web side manner" OR "All Metadata":"virtual manner" OR "All Metadata":"bedside manner" OR "All Metadata":"bed side manner" OR "All Metadata":"communication skill" OR "All Metadata":"communication technique") OR ("All Metadata":relation ONEAR/2 care OR "All Metadata":relation ONEAR/2 caring OR "All Metadata":relation ONEAR/2 care OR "All Metadata":relation ONEAR/2 caring) AND ("All Metadata":computer* ONEAR/2 instruction OR "All Metadata":"computer user training" OR "All Metadata":"interactive tutorial" OR "All Metadata":webcast OR "All Metadata":web cast OR "All Metadata":webcasting OR "All Metadata":"web casting" OR "All Metadata":"interactive learning" OR "All Metadata":simulation) OR ("All Metadata":inservice OR "All Metadata":"in service" OR "All Metadata":"on the job training" OR "All Metadata":"training on the job" OR "All Metadata":"hands on") OR ("All Metadata":practical ONEAR/3 application OR "All Metadata":practical ONEAR/3 component OR "All Metadata":practical ONEAR/3 element OR "All Metadata":practical ONEAR/3 professional development OR "All Metadata":practical ONEAR/3 session) OR ("All Metadata":"continuing education" OR "All Metadata":"continuing nursing education" OR "All Metadata":"post basic nursing education" OR "All Metadata":"post registration nursing education")) AND ("All Metadata":"social media" OR "All Metadata":"social medium" OR "All Metadata":Facebook OR "All Metadata":Flickr OR "All

Metadata":Instagram OR "All Metadata":LinkedIn OR "All Metadata":MySpace OR "All Metadata":Pinterest OR "All Metadata":Reddit OR "All Metadata":"Sina Weibo" OR "All Metadata":Snapchat OR "All Metadata":"online social network" OR "All Metadata":"social networking platform" OR "All Metadata":"social networking site" OR "All Metadata":"social networking website" OR "All Metadata":"social platform" OR "All Metadata":TikTok OR "All Metadata":Tumblr OR "All Metadata":Twitter OR "All Metadata":"web 2.0" OR "All Metadata":"web 2.0s" OR "All Metadata":WeChat OR "All Metadata":WhatsApp OR "All Metadata":YouTube OR "All Metadata":chatbot OR "All Metadata":chatbots OR "All Metadata":"chat bot" OR "All Metadata":"chat bots" OR "All Metadata":"smart technology" OR "All Metadata":"smart technologies" OR "All Metadata":wearable OR "All Metadata":wearables OR "All Metadata":"activity tracker" OR "All Metadata":"activity trackers" OR "All Metadata":"fitness tracker" OR "All Metadata":"fitness trackers" OR "All Metadata":smartwatch OR "All Metadata":"smart watch" OR "All Metadata":"smart watches" OR "All Metadata":pedometer OR "All Metadata":pedometers OR "All Metadata":smartglasses OR "All Metadata":"smart glasses" OR "All Metadata":"virtual reality" OR "All Metadata":"virtual realities" OR "All Metadata":"augmented realities" OR "All Metadata":"augmented reality" OR "All Metadata":"mixed realities" OR "All Metadata":"mixed reality" OR "All Metadata":hologram OR "All Metadata":holograms OR "All Metadata":holograph OR "All Metadata":holographs OR "All Metadata":"3d printing" OR "All Metadata":"3 d printing*" OR "All Metadata":"3 dimensional printing" OR "All Metadata":"three dimensional printing") OR ("All Metadata":wireless OR "All Metadata":internet OR "All Metadata":web OR "All Metadata":webbased OR "All Metadata":"web based"))

Search Within Results

nurse OR nurses OR nursing

Filters Applied:

Journals

2017 - 2022

Group 6 Terms Strategy

((("All Metadata":"nurse patient relation" OR "All Metadata":"patient nurse relation" OR "All Metadata":"professional patient relation" OR "All Metadata":"patient professional relation" OR "All Metadata":"nurse patient relationship" OR "All Metadata":"patient nurse relationship" OR "All Metadata":"professional patient relationship" OR "All Metadata":"patient professional relationship") OR ("All Metadata":"doctor patient relation" OR "All Metadata":"patient doctor relation" OR "All Metadata":"physician patient relation" OR "All Metadata":"patient physician relation" OR "All Metadata":"patient healthcare professional relation" OR "All Metadata":"patient health care professional relation" OR "All Metadata":"doctor patient relationship" OR "All Metadata":"patient doctor relationship" OR "All Metadata":"physician patient relationship" OR "All Metadata":"patient physician relationship" OR "All Metadata":"patient healthcare professional relationship" OR "All Metadata":"patient health care professional relationship") OR ("All Metadata":"interpersonal relation" OR "All Metadata":"interpersonal relationship" OR "All Metadata":"inter personal relation" OR "All

Metadata":"inter personal relationship" OR "All Metadata":"social relation" OR "All Metadata":"social relationship") OR ("All Metadata":"interpersonal care" OR "All Metadata":"inter personal care" OR "All Metadata":"interpersonal interaction" OR "All Metadata":"inter personal interaction") OR ("All Metadata":caring OR "All Metadata":compassion OR "All Metadata":compassionate OR "All Metadata":empathy OR "All Metadata":empathetic OR "All Metadata":sympathy OR "All Metadata":sympathetic OR "All Metadata":rapport) OR ("All Metadata":"humanistic care" OR "All Metadata":"patient centered care" OR "All Metadata":"patient centered nursing" OR "All Metadata":"patient centred care" OR "All Metadata":"patient centred nursing" OR "All Metadata":"patient focused care") OR ("All Metadata":"interpersonal communication" OR "All Metadata":"inter personal communication" OR "All Metadata": professionalism OR "All Metadata":etiquette OR "All Metadata":"webside manner" OR "All Metadata":"web side manner" OR "All Metadata":"virtual manner" OR "All Metadata":"bedside manner" OR "All Metadata":"bed side manner" OR "All Metadata":"communication skill" OR "All Metadata":"communication technique") OR ("All Metadata":relation ONEAR/2 care OR "All Metadata":relation ONEAR/2 caring OR "All Metadata":relation ONEAR/2 care OR "All Metadata":relation ONEAR/2 caring) AND ("All Metadata":computer* ONEAR/2 instruction OR "All Metadata":"computer user training" OR "All Metadata":"interactive tutorial" OR "All Metadata":webcast OR "All Metadata":web cast OR "All Metadata":webcasting OR "All Metadata":"web casting" OR "All Metadata":"interactive learning" OR "All Metadata":simulation) OR ("All Metadata":inservice OR "All Metadata":"in service" OR "All Metadata":"on the job training" OR "All Metadata":"training on the job" OR "All Metadata":"hands on") OR ("All Metadata":practical ONEAR/3 application OR "All Metadata":practical ONEAR/3 component OR "All Metadata":practical ONEAR/3 element OR "All Metadata":practical ONEAR/3 professional development OR "All Metadata":practical ONEAR/3 session) OR ("All Metadata":"continuing education" OR "All Metadata":"continuing nursing education" OR "All Metadata":"post basic nursing education" OR "All Metadata":"post registration nursing education")) AND ("All Metadata":"artificial intelligence" OR "All Metadata":"machine intelligence" OR "All Metadata":"expert system" OR "All Metadata":"expert systems" OR "All Metadata":"knowledge based system" OR "All Metadata":"knowledge based systems" OR "All Metadata":"fuzzy logic" OR "All Metadata":"fuzzy model" OR "All Metadata":"fuzzy models" OR "All Metadata":"machine learning" OR "All Metadata":"learning machine" OR "All Metadata":"learning machines" OR "All Metadata":"deep learning" OR "All Metadata":"hierarchical learning" OR "All Metadata":"ann approach" OR "All Metadata":"ann model" OR "All Metadata":"ann method" OR "All Metadata":"ann training" OR "All Metadata":"neural network" OR "All Metadata":"neural networks" OR "All Metadata":robotics OR "All Metadata":nanorobotics OR "All Metadata":robot OR "All Metadata":robots OR "All Metadata":"biomedical technology" OR "All Metadata":"bio medical technology" OR "All Metadata":"biomedical technologies" OR "All Metadata":"bio medical technologies") OR ("All Metadata":informatics OR "All Metadata":"clinical information science" OR "All Metadata":"clinical information technology" OR "All Metadata":"clinical information technologies" OR "All Metadata":"health information science" OR "All Metadata":"health information technology" OR "All Metadata":"health information technologies" OR "All Metadata":"medical computer science" OR "All Metadata":"medical information science" OR "All Metadata":"medical information technology" OR "All Metadata":"medical information technology" OR "All Metadata":"automatic diagnosis" OR "All Metadata":"computer assisted diagnosis" OR "All Metadata":"computer diagnosis" OR "All Metadata":"automatic diagnoses" OR "All Metadata":"computer assisted diagnoses" OR "All Metadata":"computer diagnoses" OR "All Metadata":"information system" OR "All Metadata":"information systems" OR "All Metadata":"online system" OR "All Metadata":"online systems" OR "All Metadata":"computer interface" OR "All Metadata":"computer interefaces" OR "All

Metadata":"computer user interface" OR "All Metadata":"computer user interfaces") OR ("All Metadata":"predictive analytics"))

Search Within Results

nurse OR nurses OR nursing

Filters Applied:

Journals

2017 - 2022

Indirect Search for Recommendation Question #2: Should education about relational care and interpersonal communication skills be recommended for nurses practicing in virtual care settings and in-person digital health environments?

Dates searched: January 1, 2017 – February 17, 2023

Databases searched: MEDLINE, Embase and CINAHL

**Note: The original search strategy was broadened to examine relational care and interpersonal communication skills in general (not specific to digital health technologies), and all health providers (not just nurses). The search was only applied to MEDLINE, Embase, and CINAHL for feasibility purposes, and limited to systematic reviews in order to obtain the highest quality evidence.*

Database: Medline
Search strategy:

#	Searches	Results
1	exp "Attitude of Health Personnel"/	168985
2	exp Health Personnel/	602334
3	exp Personnel, Hospital/	94980
4	exp Health Occupations/	1839086
5	exp Patient Care Team/	72570
6	Social Work/	16199
7	Social Workers/	1056
8	Nurse's Role/	42689
9	Licensed Practical Nurses/	99
10	exp Nurses/	97258

11	exp Nursing Staff/	69574
12	Nursing Staff, Hospital/	47756
13	exp Nursing/	262616
14	Nursing, Practical/	3444
15	Health Educators/	492
16	exp Administrative Personnel/	42164
17	(health* adj2 (provider? or staff? or personnel? or employe* or profession* or occupation? or practitioner? or worker?)).tw,kf.	315788
18	(hospital* adj2 (provider? or staff? or personnel? or employe* or profession* or occupation? or practitioner? or worker?)).tw,kf.	21133
19	nurs*.tw,kf.	524010
20	(patient care? adj2 team?).tw,kf.	1019
21	(health* adj2 educator?).tw,kf.	3803
22	(social adj2 worker?).tw,kf.	12548
23	(support* adj2 (worker? or staff?)).tw,kf.	8087
24	clinician?.tw,kf.	288672
25	doctor?.tw,kf.	142656
26	physician?.tw,kf.	451030
27	practitioner?.tw,kf.	176317
28	surgeon?.tw,kf.	243497
29	Caregivers/	48670
30	(caregiver? or care-giver? or carer or carers).tw,kf.	105711
31	or/1-30	3633089
32	Computer User Training/	2042
33	computer user training*.tw,kf.	12
34	Computer Assisted Instruction/	12501
35	(computer assisted instruction* or computerised programmed instruction* or computerised self instruction program* or computerized programmed instruction* or computerized self instruction program*).tw,kf.	721
36	interactive tutorial/ or webcast/	1391
37	(interactive tutorial or interactive tutorials or webcast* or "web cast*").tw,kf.	393
38	InService Training/	20739
39	(inservice or "in service" or "on the job training" or "training on the job").tw,kf.	11623
40	Simulation Training/ or High Fidelity Simulation Training/ or Patient Simulation/	11447
41	(interactive learning or simulation or simulat*).tw,kf.	672953
42	exp education, continuing/ or education, nursing, continuing/	62588
43	(continuing education or continuing medical education or continuing pharma* education or continuing nursing education or "post basic nursing education" or "post registration nursing education").tw,kf.	20549
44	exp Health Personnel/ed [Education]	64554

45	education, professional/ or clinical clerkship/ or exp education, medical/ or exp education, nursing/ or exp education, pharmacy/ or education, public health professional/	284438
46	or/32-45 [Professional development education]	1034161
47	interpersonal relations/ or professional-patient relations/ or nurse-patient relations/ or physician-patient relations/	212287
48	(nurse* patient relation* or patient nurs* relation* or professional* patient relation* or patient professional* relation* or doctor patient relation* or patient doctor relation* or physician patient relation* or patient physician relation* or patient healthcare professional relation* or patient health care professional relation*).tw,kf.	26787
49	(interpersonal relation* or "inter personal relation*" or social relation*).tw,kf.	20450
50	(interpersonal care or "inter personal care").tw,kf.	161
51	Empathy/	22480
52	(caring or compassion* or empathy or empathetic).tw,kf.	79447
53	rapport.tw,kf.	6285
54	Humanism/	3746
55	humanistic care.tw,kf.	213
56	Patient-Centered Care/	22634
57	(patient centered care or patient centered nursing or patient centred care or patient centred nursing or patient focused care).tw,kf.	10970
58	(interpersonal communication* or "inter personal communication*").tw,kf.	2094
59	Professionalism/	1758
60	professionalism.tw,kf.	9691
61	etiquette.tw,kf.	656
62	(webside manner? or web side manner? or virtual manner?).tw,kf.	33
63	(bedside manner? or bed side manner?).tw,kf.	215
64	Communication/	98330
65	(communication skill? or communication technique?).tw,kf.	14743
66	(relation* adj (center* or centr*) adj (care or caring)).tw,kf.	231
67	or/47-66	428798
68	31 and 46 and 67	30702
69	limit 68 to english language	28751
70	limit 69 to yr="2017 -Current"	7474
71	review/	3117010
72	(medline or medlars or pubmed or grateful med or CINAHL or scisearch or psychinfo or psycinfo or psychlit or psyclit or handsearch* or hand search* or manual* search* or electronic database* or bibliographic database* or embase or lilacs or scopus or "web of science").mp.	369357
73	71 and 72	195707
74	meta-analysis.mp.	267074
75	meta-analysis as topic/	22121

76	meta-analysis/	175909
77	meta-analysis.tw.	222959
78	cochrane database*.jn.	16190
79	or/73-78	404529
80	70 and 79	227

Database: Embase
Search strategy:

#	Searches	Results
1	exp health personnel attitude/	203650
2	exp health care personnel/	1988852
3	exp hospital personnel/	153201
4	exp multidisciplinary team/	24342
5	social work/	30396
6	social worker/	16423
7	licensed practical nurse/	380
8	exp nurse/	220075
9	nursing staff/	77616
10	exp nursing/	416847
11	practical nursing/	166
12	exp health educator/	3450
13	exp administrative personnel/	35104
14	nurse attitude/	43216
15	(health* adj2 (provider? or staff? or personnel? or employe* or profession* or occupation? or practitioner? or worker?)).tw,kw.	405876
16	(hospital* adj2 (provider? or staff? or personnel? or employe* or profession* or occupation? or practitioner? or worker?)).tw,kw.	29299
17	nurs*.tw,kw.	630798
18	(patient care? adj2 team?).tw,kw.	871
19	(health* adj2 educator?).tw,kw.	4376
20	(social adj2 worker?).tw,kw.	21330
21	(support* adj2 (worker? or staff?)).tw,kw.	11470
22	clinician?.tw,kw.	416726
23	doctor?.tw,kw.	213201
24	physician?.tw,kw.	662649
25	practitioner?.tw,kw.	228020
26	surgeon?.tw,kw.	386065
27	caregiver/	107989

28	(caregiver? or care-giver? or carer or carers).tw,kw.	149642
29	or/1-28	3799628
30	human computer interaction/	6661
31	computer user training*.tw,kw.	21
32	(computer assisted instruction* or computerised programmed instruction* or computerised self instruction program* or computerized programmed instruction* or computerized self instruction program*).tw,kw.	977
33	webcast/	437
34	(interactive tutorial or interactive tutorials or webcast* or "web cast*").tw,kf.	653
35	"in service training"/	16798
36	(inservice or "in service" or "on the job training" or "training on the job").tw,kw.	14868
37	simulation/ or computer simulation/ or digital twin/ or discrete event simulation/ or high fidelity simulation/ or high-fidelity patient simulation/ or patient simulation/	377592
38	(interactive learning or simulation or simulat*).tw,kw.	727707
39	continuing education/	33507
40	(continuing education or continuing medical education or continuing pharma* education or continuing nursing education or "post basic nursing education" or "post registration nursing education").tw,kw.	27078
41	exp medical education/ or exp paramedical education/	481823
42	or/30-41	1323538
43	human relation/	99822
44	professional-patient relationship/ or doctor patient relationship/ or nurse patient relationship/	54097
45	(nurse* patient relation* or patient nurs* relation* or professional* patient relation* or patient professional* relation* or doctor patient relation* or patient doctor relation* or physician patient relation* or patient physician relation* or patient healthcare professional relation* or patient health care professional relation*).tw,kw.	16311
46	(interpersonal relation* or "inter personal relation*" or social relation*).tw,kw.	25411
47	(interpersonal care or "inter personal care").tw,kw.	187
48	(interpersonal interaction? or "inter personal interaction?").tw,kw.	1730
49	empathy/	31838
50	(caring or compassion* or empathy or empathetic or sympathy or sympathetic).tw,kw.	229050
51	rapport.tw,kw.	5358
52	humanism/	3918
53	humanistic care.tw,kw.	262
54	(patient centered care or patient centered nursing or patient centred care or patient centred nursing or patient focused care).tw,kw.	14280
55	interpersonal communication/	186967
56	(interpersonal communication* or "inter personal communication*").tw,kw.	2585
57	professionalism/	10922
58	professionalism.tw,kw.	11677

59	etiquette.tw,kw.	816
60	(webside manner? or web side manner? or virtual manner?).tw,kw.	41
61	(bedside manner? or bed side manner?).tw,kw.	298
62	communication skill/	17555
63	(communication skill? or communication technique?).tw,kw.	19333
64	(relation* adj (center* or centr*) adj (care or caring)).tw,kw.	230
65	or/43-64	619065
66	29 and 42 and 65	37510
67	limit 66 to english language	35536
68	limit 67 to yr="2017 -Current"	11268
69	limit 68 to (books or chapter or conference abstract or conference paper or "conference review")	1761
70	68 not 69	9507
71	"review"/	2894928
72	(medline or medlars or pubmed or grateful med or CINAHL or scisearch or psychinfo or psycinfo or psychlit or psyclit or handsearch* or hand search* or manual* search* or electronic database* or bibliographic database* or embase or lilacs or scopus or web of science).mp.	472347
73	71 and 72	188599
74	meta-analysis.mp.	398297
75	"meta analysis (topic)"/	50966
76	meta analysis/	277891
77	systematic review*.tw.	338134
78	cochrane database*.jn.	22875
79	or/73-78	652534
80	70 and 79	326

Database: Cumulative Index to Nursing and Allied Health (CINAHL)
Search strategy:

#	Query	Limiters/Expanders	Results
S1	(MH "Attitude of Health Personnel+")	Expanders - Apply equivalent subjects<br>Search modes - Boolean/Phrase	122,929
S2	(MH "Health Personnel+")	Expanders - Apply equivalent subjects<br>Search modes - Boolean/Phrase	640,200
S3	(MH "Personnel, Health Facility+")	Expanders - Apply equivalent subjects<br>Search modes - Boolean/Phrase	44,877
S4	(MH "Health Occupations+")	Expanders - Apply equivalent subjects<br>Search modes - Boolean/Phrase	826,499

S5	(MH "Multidisciplinary Care Team+")	Expanders - Apply equivalent subjects<br>Search modes - Boolean/Phrase	51,930
S6	(MH "Social Work+")	Expanders - Apply equivalent subjects<br>Search modes - Boolean/Phrase	15,393
S7	(MH "Social Workers")	Expanders - Apply equivalent subjects<br>Search modes - Boolean/Phrase	11,230
S8	(MH "Nursing Role")	Expanders - Apply equivalent subjects<br>Search modes - Boolean/Phrase	65,723
S9	(MH "Practical Nurses") OR (MH "Practical Nursing")	Expanders - Apply equivalent subjects<br>Search modes - Boolean/Phrase	7,726
S10	(MH "Nurses+")	Expanders - Apply equivalent subjects<br>Search modes - Boolean/Phrase	242,492
S11	(MH "Nursing Staff, Hospital")	Expanders - Apply equivalent subjects<br>Search modes - Boolean/Phrase	25,146
S12	(MH "Health Educators+")	Expanders - Apply equivalent subjects<br>Search modes - Boolean/Phrase	4,221
S13	(MH "Administrative Personnel+")	Expanders - Apply equivalent subjects<br>Search modes - Boolean/Phrase	40,759
S14	TI (health* N2 provider#) OR AB (health* N2 provider#)	Expanders - Apply equivalent subjects<br>Search modes - Boolean/Phrase	57,827
S15	TI (hospital* N2 provider#) OR AB (hospital* N2 provider#)	Expanders - Apply equivalent subjects<br>Search modes - Boolean/Phrase	1,829
S16	TI (health* N2 staff#) OR AB (health* N2 staff#)	Expanders - Apply equivalent subjects<br>Search modes - Boolean/Phrase	8,994
S17	TI (hospital* N2 staff#) OR AB (hospital* N2 staff#)	Expanders - Apply equivalent subjects<br>Search modes - Boolean/Phrase	6,079
S18	TI (health* N2 personnel#) OR AB (health* N2 personnel#)	Expanders - Apply equivalent subjects<br>Search modes - Boolean/Phrase	5,454
S19	TI (hospital* N2 personnel#) OR AB (hospital* N2 personnel#)	Expanders - Apply equivalent subjects<br>Search modes - Boolean/Phrase	1,157
S20	TI (health* N2 employe*) OR AB (health* N2 employe*)	Expanders - Apply equivalent subjects<br>Search modes - Boolean/Phrase	7,148
S21	TI (hospital* N2 employe*) OR AB (hospital* N2 employe*)	Expanders - Apply equivalent subjects<br>Search modes - Boolean/Phrase	2,073
S22	TI (health* N2 profession*) OR AB (health* N2 profession*)	Expanders - Apply equivalent subjects<br>Search modes - Boolean/Phrase	101,185
S23	TI (hospital* N2 profession*) OR AB (hospital* N2 profession*)	Expanders - Apply equivalent subjects<br>Search modes - Boolean/Phrase	1,997
S24	TI (health* N2 occupation#) OR AB (health* N2 occupation#)	Expanders - Apply equivalent subjects<br>Search modes - Boolean/Phrase	1,023
S25	TI (hospital* N2 occupation#) OR AB (hospital* N2 occupation#)	Expanders - Apply equivalent subjects<br>Search modes - Boolean/Phrase	59

S26	TI (health* N2 practitioner#) OR AB (health* N2 practitioner#)	Expanders - Apply equivalent subjects Search modes - Boolean/Phrase	12,235
S27	TI (hospital* N2 practitioner#) OR AB (hospital* N2 practitioner#)	Expanders - Apply equivalent subjects Search modes - Boolean/Phrase	933
S28	TI (health* N2 worker#) OR AB (health* N2 worker#)	Expanders - Apply equivalent subjects Search modes - Boolean/Phrase	34,243
S29	TI (hospital* N2 worker#) OR AB (hospital* N2 worker#)	Expanders - Apply equivalent subjects Search modes - Boolean/Phrase	1,604
S30	TI nurs* OR AB nurs*	Expanders - Apply equivalent subjects Search modes - Boolean/Phrase	642,418
S31	TI (patient care# N2 team#) OR AB (patient care# N2 team#)	Expanders - Apply equivalent subjects Search modes - Boolean/Phrase	589
S32	TI (health* N2 educator#) OR AB (health* N2 educator#)	Expanders - Apply equivalent subjects Search modes - Boolean/Phrase	3,780
S33	TI (social N2 worker#) OR AB (social N2 worker#)	Expanders - Apply equivalent subjects Search modes - Boolean/Phrase	14,909
S34	TI (support* N2 (worker# or staff#)) OR AB (support* N2 (worker# or staff#))	Expanders - Apply equivalent subjects Search modes - Boolean/Phrase	9,698
S35	TI clinician# OR AB clinician#	Expanders - Apply equivalent subjects Search modes - Boolean/Phrase	123,667
S36	TI doctor# OR AB doctor#	Expanders - Apply equivalent subjects Search modes - Boolean/Phrase	65,516
S37	TI physician# OR AB physician#	Expanders - Apply equivalent subjects Search modes - Boolean/Phrase	169,785
S38	TI practitioner# OR AB practitioner#	Expanders - Apply equivalent subjects Search modes - Boolean/Phrase	108,225
S39	TI surgeon# OR AB surgeon#	Expanders - Apply equivalent subjects Search modes - Boolean/Phrase	63,550
S40	(MH "Caregivers")	Expanders - Apply equivalent subjects Search modes - Boolean/Phrase	43,020
S41	TI (caregiver# or care-giver# or carer or carers) OR AB (caregiver# or care-giver# or carer or carers)	Expanders - Apply equivalent subjects Search modes - Boolean/Phrase	79,928
S42	S1 OR S2 OR S3 OR S4 OR S5 OR S6 OR S7 OR S8 OR S9 OR S10 OR S11 OR S12 OR S13 OR S14 OR S15 OR S16 OR S17 OR S18 OR S19 OR S20 OR S21 OR S22 OR S23 OR S24 OR S25 OR S26 OR S27 OR S28 OR S29 OR S30 OR S31 OR S32 OR S33 OR S34 OR S35 OR S36 OR S37 OR S38 OR S39 OR S40 OR S41	Expanders - Apply equivalent subjects Search modes - Boolean/Phrase	2,200,271

S43	(MH "Computer User Training")	Expanders - Apply equivalent subjects Search modes - Boolean/Phrase	798
S44	TI (computer user training*) OR AB (computer user training*)	Expanders - Apply equivalent subjects Search modes - Boolean/Phrase	0
S45	(MH "Computer Assisted Instruction")	Expanders - Apply equivalent subjects Search modes - Boolean/Phrase	8,293
S46	TI (computer assisted instruction* or computerised programmed instruction* or computerised self instruction program* or computerized programmed instruction* or computerized self instruction program*) OR AB (computer assisted instruction* or computerised programmed instruction* or computerised self instruction program* or computerized programmed instruction* or computerized self instruction program*)	Expanders - Apply equivalent subjects Search modes - Boolean/Phrase	372
S47	(MH "Webcasts")	Expanders - Apply equivalent subjects Search modes - Boolean/Phrase	913
S48	TI (interactive tutorial or interactive tutorials or webcast* or "web cast*") OR AB (interactive tutorial or interactive tutorials or webcast* or "web cast*")	Expanders - Apply equivalent subjects Search modes - Boolean/Phrase	317
S49	(MH "Staff Development")	Expanders - Apply equivalent subjects Search modes - Boolean/Phrase	29,808
S50	TI (inservice or "in service" or "on the job training" or "training on the job") OR AB (inservice or "in service" or "on the job training" or "training on the job")	Expanders - Apply equivalent subjects Search modes - Boolean/Phrase	8,154
S51	(MH "Patient Simulation") OR (MH "Simulations") OR (MH "Vignettes")	Expanders - Apply equivalent subjects Search modes - Boolean/Phrase	28,633
S52	TI (interactive learning or simulation* or simulat*) OR AB (interactive learning or simulation* or simulat*)	Expanders - Apply equivalent subjects Search modes - Boolean/Phrase	68,732
S53	MH "Education, Post-RN") OR (MH "Education, Continuing") OR (MH "Education, Nursing, Continuing") OR (MH "Health Personnel/ED")	Expanders - Apply equivalent subjects Search modes - Boolean/Phrase	39,193
S54	TI (continuing education or continuing nursing education or "post basic nursing education" or "post registration nursing education") OR AB (continuing education or continuing nursing education or "post basic nursing education" or "post registration nursing education")	Expanders - Apply equivalent subjects Search modes - Boolean/Phrase	15,608

S55	(MH "Education, Health Sciences") OR (MH "Education, Allied Health+") OR (MH "Education, Medical+") OR (MH "Education, Nursing+") OR (MH "Education, Pharmacy") OR (MH "Education, Podiatry")	Expanders - Apply equivalent subjects Search modes - Boolean/Phrase	159,945
S56	S43 OR S44 OR S45 OR S46 OR S47 OR S48 OR S49 OR S50 OR S51 OR S52 OR S53 OR S54 OR S55	Expanders - Apply equivalent subjects Search modes - Boolean/Phrase	300,414
S57	(MH "Professional-Patient Relations") OR (MH "Nurse-Patient Relations") OR (MH "Physician-Patient Relations") OR (MH "Interpersonal Relations")	Expanders - Apply equivalent subjects Search modes - Boolean/Phrase	162,559
S58	TI (nurse* patient relation* or patient nurs* relation* or professional* patient relation* or patient professional* relation* or doctor patient relation* or patient doctor relation* or physician patient relation* or patient physician relation* or patient healthcare professional relation* or patient health care professional relation*)	Expanders - Apply equivalent subjects Search modes - Boolean/Phrase	1,105
S59	AB (nurse* patient relation* or patient nurs* relation* or professional* patient relation* or patient professional* relation* or doctor patient relation* or patient doctor relation* or physician patient relation* or patient physician relation* or patient healthcare professional relation* or patient health care professional relation*)	Expanders - Apply equivalent subjects Search modes - Boolean/Phrase	4,131
S60	TI (interpersonal relation* or "inter personal relation*" or social relation*) OR AB (interpersonal relation* or "inter personal relation*" or social relation*)	Expanders - Apply equivalent subjects Search modes - Boolean/Phrase	10,728
S61	TI (interpersonal care or "inter personal care") OR AB (interpersonal care or "inter personal care")	Expanders - Apply equivalent subjects Search modes - Boolean/Phrase	132
S62	TI (interpersonal interaction# or "inter personal interaction#") OR AB (interpersonal interaction# or "inter personal interaction#")	Expanders - Apply equivalent subjects Search modes - Boolean/Phrase	715
S63	(MH "Empathy")	Expanders - Apply equivalent subjects Search modes - Boolean/Phrase	13,189
S64	(MH "Compassion")	Expanders - Apply equivalent subjects Search modes - Boolean/Phrase	3,186

S65	TI (caring or compassion* or empathy or empathetic or sympathy or sympathetic) OR AB (caring or compassion* or empathy or empathetic or sympathy or sympathetic)	Expanders - Apply equivalent subjects Search modes - Boolean/Phrase	78,944
S66	(MH "Humanism")	Expanders - Apply equivalent subjects Search modes - Boolean/Phrase	2,349
S67	TI "humanistic care" OR AB "humanistic care"	Expanders - Apply equivalent subjects Search modes - Boolean/Phrase	198
S68	(MH "Patient Centered Care")	Expanders - Apply equivalent subjects Search modes - Boolean/Phrase	36,110
S69	TI (patient centered care or patient centered nursing or patient centred care or patient centred nursing or patient focused care) OR AB (patient centered care or patient centered nursing or patient centred care or patient centred nursing or patient focused care)	Expanders - Apply equivalent subjects Search modes - Boolean/Phrase	5,988
S70	TI (interpersonal communication* or "inter personal communication*") OR AB (interpersonal communication* or "inter personal communication*")	Expanders - Apply equivalent subjects Search modes - Boolean/Phrase	1,220
S71	TI rapport OR AB rapport	Expanders - Apply equivalent subjects Search modes - Boolean/Phrase	4,902
S72	(MH "Professionalism")	Expanders - Apply equivalent subjects Search modes - Boolean/Phrase	8,267
S73	TI professionalism OR AB professionalism	Expanders - Apply equivalent subjects Search modes - Boolean/Phrase	6,049
S74	TI etiquette OR AB etiquette	Expanders - Apply equivalent subjects Search modes - Boolean/Phrase	640
S75	TI (webside manner# or web side manner# or virtual manner#) OR AB (webside manner# or web side manner# or virtual manner#)	Expanders - Apply equivalent subjects Search modes - Boolean/Phrase	14
S76	TI (bedside manner# or bed side manner#) OR AB (bedside manner# or bed side manner#)	Expanders - Apply equivalent subjects Search modes - Boolean/Phrase	175
S77	(MH "Communication") OR (MH "Communication Skills")	Expanders - Apply equivalent subjects Search modes - Boolean/Phrase	104,367
S78	TI (communication skill# or communication technique#) OR AB (communication skill# or communication technique#)	Expanders - Apply equivalent subjects Search modes - Boolean/Phrase	9,398

S79	TI (relation* N1 (center* or centr*) N1 (care or caring)) OR AB (relation* N1 (center* or centr*) N1 (care or caring))	Expanders - Apply equivalent subjects Search modes - Boolean/Phrase	207
S80	S57 OR S58 OR S59 OR S60 OR S61 OR S62 OR S63 OR S64 OR S65 OR S66 OR S67 OR S68 OR S69 OR S70 OR S71 OR S72 OR S73 OR S74 OR S75 OR S76 OR S77 OR S78 OR S79	Expanders - Apply equivalent subjects Search modes - Boolean/Phrase	368,673
S81	S42 AND S56 AND S80	Expanders - Apply equivalent subjects Search modes - Boolean/Phrase	19,971
S82	MH "Literature Review")	Expanders - Apply equivalent subjects Search modes - Boolean/Phrase	9,020
S83	TX (medline OR medlars OR pubmed OR grateful med OR CINAHL OR scisearch OR psychinfo OR psycinfo OR psychlit OR psyclit OR handsearch* OR "hand search*" OR "manual* search*" OR "electronic database*" OR "bibliographic database*" OR embase OR lilacs OR scopus OR "web of science")	Expanders - Apply equivalent subjects Search modes - Boolean/Phrase	273,412
S84	S82 AND S83	Expanders - Apply equivalent subjects Search modes - Boolean/Phrase	2,411
S85	TI ("meta-analysis") OR AB ("meta-analysis")	Expanders - Apply equivalent subjects Search modes - Boolean/Phrase	90,651
S86	(MH "Meta Analysis")	Expanders - Apply equivalent subjects Search modes - Boolean/Phrase	68,613
S87	TI (systematic review*) OR AB (systematic review*)	Expanders - Apply equivalent subjects Search modes - Boolean/Phrase	137,714
S88	JN cochrane database*	Expanders - Apply equivalent subjects Search modes - Boolean/Phrase	6,023
S89	S84 OR S85 OR S86 OR S87 OR S88	Expanders - Apply equivalent subjects Search modes - Boolean/Phrase	187,792
S90	S81 AND S89	Expanders - Apply equivalent subjects Search modes - Boolean/Phrase	285
S91	S81 AND S89	Limiters - Published Date: 20170101-20231231 Expanders - Apply equivalent subjects Search modes - Boolean/Phrase	190
S92	S81 AND S89	Limiters - Published Date: 20170101-20231231; English Language Expanders - Apply equivalent subjects Search modes - Boolean/Phrase	182

Recommendation Question #3: Should the implementation of interdisciplinary peer champion models in health-service organizations be recommended or not to facilitate education for health providers on the use of digital health technologies?

Dates searched: January 1, 2017 – August 3, 2022

Databases searched:
- MEDLINE
- MEDLINE Epub Ahead of Print and In-Process
- Embase
- Emcare Nursing
- Cochrane Central Register of Controlled Trials
- Cochrane Database of Systematic Reviews
- APA PsychInfo
- Cumulative Index to Nursing and Allied Health (CINAHL)
- IEEE Explore

Database: Medline
Search strategy:

#	Searches	Results
1	nurses/ or nurse administrators/ or nurse practitioners/ or family nurse practitioners/ or pediatric nurse practitioners/ or nurse specialists/ or nurse anesthetists/ or nurse clinicians/ or nurse midwives/ or nurses, pediatric/ or nurses, neonatal/ or nurses, community health/ or nurses, public health/	94366
2	nursing staff/ or nursing staff, hospital/	69180
3	nursing care/ or cardiovascular nursing/ or critical care nursing/ or developmental disability nursing/ or emergency nursing/ or geriatric nursing/ or holistic nursing/ or home nursing/ or "hospice and palliative care nursing"/ or maternal-child nursing/ or medical-surgical nursing/ or nephrology nursing/ or neuroscience nursing/ or nursing, practical/ or obstetric nursing/ or occupational health nursing/ or oncology nursing/ or orthopedic nursing/ or pediatric nursing/ or perioperative nursing/ or primary nursing/ or psychiatric nursing/ or rehabilitation nursing/ or trauma nursing/	128463
4	primary care nursing/	556
5	specialties, nursing/ or advanced practice nursing/ or cardiovascular nursing/ or community health nursing/ or home health nursing/ or parish nursing/ or critical care nursing/ or developmental disability nursing/ or emergency nursing/ or family nursing/ or forensic nursing/ or geriatric nursing/ or holistic nursing/ or "hospice and palliative care nursing"/ or maternal-child nursing/ or neonatal nursing/ or medical-surgical nursing/ or midwifery/ or military nursing/ or nephrology nursing/ or neuroscience nursing/ or obstetric nursing/ or occupational health nursing/ or oncology nursing/ or orthopedic nursing/ or pediatric nursing/ or perioperative nursing/ or operating room nursing/ or postanesthesia nursing/ or psychiatric nursing/ or public health nursing/ or	163272

	"radiologic and imaging nursing"/ or rehabilitation nursing/ or rural nursing/ or school nursing/ or transcultural nursing/ or trauma nursing/	
6	Nursing, Practical/	3443
7	Nurse's Role/	42490
8	nursing/ or nursing, private duty/ or nursing, supervisory/ or office nursing/ or telenursing/ or travel nursing/	60744
9	nursing process/ or nursing assessment/ or nursing diagnosis/	38429
10	nursing services/ or home care services/ or home health nursing/ or nursing service, hospital/	51579
11	Nursing, Team/	2595
12	Nursing Faculty Practice/	615
13	(nurse or nurses or nursing).tw,kf.	451598
14	exp health personnel/	588616
15	(health personnel or health employee* or healthcare employee* or health care employee* or healthcare professional* or health care professional* or healthcare provider* or health care provider* or healthcare personnel or health care personnel or healthcare worker* or health care worker* or medical staff).tw,kf.	156714
16	(doctor or doctors or hospital registrar* or physician* or allergist* or anesthesiologist* or cardiologist* or dermatologist* or endocrinologist* or gastroenterologist* or general practitioner* or geriatrician* or hospitalist* or nephrologist* or neurologist* or oncologist* or ophthalmologist* or osteopathic physician* or otolaryngologist* or pathologist* or paediatrician* or pediatrician* or neonatologist* or pharmacist or pharmacists or physiatrist* or pulmonologist* or radiologist* or rheumatologist* or surgeon* or neurosurgeon* or urologist* or psychotherapist*).tw,kf.	932975
17	(occupational therapist* or physical therapist*).tw,kf.	11351
18	Nutritionists/	1640
19	(dietician or dietitian or nutritionist or dieticians or dietitians or nutritionists).tw,kf.	10634
20	patient care team/	68929
21	(health team or health teams or healthcare team* or health care team* or interdisciplinary care team* or medical care team or medical care teams or multidisciplinary care team* or patient care team or patient care teams).tw,kf.	11843
22	Social Workers/	971
23	(case worker or case workers or caseworker or caseworkers or social worker or social workers).tw,kf.	10575
24	(respiratory care practitioner or respiratory care practitioners or respiratory therapist or respiratory therapists).tw,kf.	1372
25	(logopedist or logopedists or "speech and language pathologist" or "speech and language pathologist*" or speech language pathologist or speech language pathologists or speech therapist or speech therapists or speech language therapist or speech language therapists).tw,kf.	3967
26	or/1-25	1903674
27	telemedicine/	34446

28	(telemedicine or telecardiology or teledermatology or telediagnosis or telediagnoses or telehealth or telemonitoring or telenephrology or teleneurology or telepsychology or teleradiology or teleradiotherap* or telesurger* or teletherap* or videoconsult*).tw,kf.	27206
29	("tele medicine" or "tele cardiology" or "tele dermatology" or "tele diagnosis" or "tele diagnoses" or "tele monitoring" or "tele nephrology" or "tele neurology" or "tele psychology" or "tele radiology" or "tele radiotherap*" or "tele surger*" or "tele therap*" or "video consult*").tw,kf.	923
30	("e-health" or ehealth or "tele health").tw,kf.	7465
31	(telenurs* or "tele nurs*").tw,kf.	266
32	remote consultation/	5572
33	("cyber consult*" or cyberconsult* or econsult* or "e−consult*" or "email based consult*" or "internet consult*" or "internet based consult*" or "online consult*" or "tele consult*" or "telephone based consult*" or "telephone consult*" or "virtual consult*" or "web consult*" or "webbased consult*" or "web based consult*").tw,kf.	1750
34	telerehabilitation/	835
35	("e−rehabilitation" or remote rehabilitation or tele rehabilitation or virtual rehabilitation).tw,kf.	388
36	medical records systems, computerized/	19138
37	(computerised medical record system* or computerized medical record system*).tw,kf.	96
38	electronic health records/	25627
39	(electronic health record* or computerised patient record* or computerized patient record* or electronic medical record* or electronic patient record* or ehr or emr).tw,kf.	45849
40	health smart cards/	63
41	(smart card or smart cards).tw,kf.	299
42	medical order entry systems/	2427
43	medical order entry system*.tw,kf.	88
44	hospital information systems/	11056
45	(hospital information system* or patient health record information system*).tw,kf.	2736
46	ambulatory care information systems/	1171
47	ambulatory care information system*.tw,kf.	15
48	electronic prescribing/	1152
49	("e−prescribing" or "e−prescription*" or electronic prescription*).tw,kf.	557
50	("c.p.o.e. system" or computer* order entry or computer* physician order entry or computer* provider order entry or computer* order entry or computer* prescriber order entry or computer* prescribing order entry or computer* prescription order entry or "cpoe").tw,kf.	1901
51	operating room information systems/	528
52	(operating room information system* or operating room information management system*).tw,kf.	29

53	Point-of-Care Systems/	15665
54	(bedside computing or bedside technolog* or point of care system* or point of care technolog* or point of care information system*).tw,kf.	757
55	bedside information system*.tw,kf.	10
56	(management information system* or management information).tw,kf.	1960
57	clinical pharmacy information systems/	1198
58	clinical pharmacy information system*.tw,kf.	20
59	database management systems/	7724
60	(database management system* or database management tool or database management tools or database manager system* or database managing system* or data base manager system* or data base management software or data base management system* or data base management tool or data base management tools).tw,kf.	593
61	decision support systems, management/	967
62	decision support system*.tw,kf.	6396
63	Mobile Applications/	10397
64	(mobile app or mobile apps or mobile application* or mobile health app or mobile health apps or mobile health application* or portable software app or portable software apps or portable software application* or tablet app or tablet apps or tablet application*).tw,kf.	6137
65	cell phone/	9712
66	(cell phone or cell phones or cellphone or cellphones or cellular phone or cellular phones or cellular telephone or cellular telephones or mobile phone or mobile phones or mobile telephone or mobile telephones).tw,kf.	12459
67	smartphone/	8056
68	(smartphone or smartphones or smart phones or smart phones).tw,kf.	13978
69	text messaging/	4190
70	(text messaging or texting).tw,kf.	2921
71	videoconferencing/	2259
72	(video conference* or video conferencing or videoconferenc*).tw,kf.	3650
73	webcast/	1103
74	webcasts as topic/	409
75	(webcast* or web cast*).tw,kf.	243
76	Wireless Technology/	4411
77	(wireless communication* or wireless technol*).tw,kf.	1478
78	"Cell Phone Use"/	351
79	computers, handheld/	3998
80	(hand held computer* or handheld computer* or palm PC or palmtop or personal data assistant* or personal digital assistant or pocket computer* or pocket sized computer*).tw,kf.	1361
81	internet/	79626
82	internet.tw,kf.	52627

83	internet-based intervention/	988
84	(internet based intervention* or online based intervention* or online intervention* or web intervention* or web based intervention*).tw,kf.	2634
85	smart technolog*.tw,kf.	288
86	wearable electronic devices/	6465
87	(wearable computer or wearable computers or wearable electronic device*).tw,kf.	305
88	fitness trackers/	1035
89	(activity tracker* or fitness tracker* or smart watch or smart watches or pedometer*).tw,kf.	3474
90	smart glasses/	147
91	("ar glasses" or "ar head mounted device" or "ar head mounted display" or "ar head worn display" or "ar headset" or "ar hud" or "arhmd" or "hmd ar" or "optical see through head mounted display" or "ost hmd" or "see through hmd" or "google glasses" or "smartglasses" or "smart glasses").tw,kf.	203
92	(augmented reality glasses or augmented reality head mounted device or augmented reality head mounted display or augmented reality head up display or augmented reality head worn display or head mounted display augmented reality).tw,kf.	45
93	artificial intelligence/	33041
94	(artificial intelligence or machine intelligence).tw,kf.	16213
95	computer heuristics/	52
96	computer heuristic*.tw,kf.	0
97	expert systems/	3475
98	(expert system* or knowledge based system*).tw,kf.	3268
99	fuzzy logic/	5116
100	(fuzzy logic or fuzzy model or fuzzy models).tw,kf.	1823
101	machine learning/	26926
102	(machine learning or learning machine*).tw,kf.	45558
103	deep learning/	12148
104	(deep learning or hierarchical learning).tw,kf.	21257
105	unsupervised machine learning/	712
106	unsupervised machine learning.tw,kf.	587
107	natural language processing/	5657
108	natural language processing.tw,kf.	4404
109	neural networks, computer/	41498
110	("ann approach*" or "ann model" or "ann method*" or "ann training" or artificial neural network or computer neural network or computer neural networks).tw,kf.	7420
111	robotics/	25168
112	(robotics or nanorobotics or robot or robots).tw,kf.	25495
113	biomedical technology/	7177
114	(biomedical technology or bio medical technology).tw,kf.	387
115	informatics/	1170

116	informatics.tw,kf.	14690
117	medical informatics/	12818
118	(clinical information science or clinical information technology or health information science or health information technology or medical computer science or medical information science or medical information technology).tw,kf.	3282
119	medical informatics computing/	761
120	nursing informatics/	1607
121	health information exchange/	1063
122	health information exchange.tw,kf.	1018
123	medical informatics applications/	2550
124	decision making, computer-assisted/	2876
125	(decision support system* or decision support techniques).tw,kf.	6756
126	diagnosis, computer-assisted/	23922
127	(automatic diagnosis or computer assisted diagnosis or computer diagnosis or automatic diagnoses or computer assisted diagnoses or computer diagnoses).tw,kf.	1313
128	image interpretation, computer-assisted/	47671
129	computer assisted image interpretation.tw,kf.	21
130	radiographic image interpretation, computer-assisted/	15746
131	computer assisted radiographic image interpretation*.tw,kf.	16
132	therapy, computer-assisted/	6961
133	computer assisted therap*.tw,kf.	86
134	drug therapy, computer-assisted/	1690
135	computer assisted drug therap*.tw,kf.	16
136	decision support systems, clinical/	9167
137	clinical decision support system*.tw,kf.	2410
138	information systems/	19342
139	(information system or information systems).tw,kf.	33930
140	online systems/	8530
141	(online system or online systems).tw,kf.	569
142	user-computer interface/	39137
143	(computer interface* or computer user interface*).tw,kf.	5493
144	Social Media/	13926
145	(social media or social medium or Facebook or Flickr or Instagram or LinkedIn or MySpace or Pinterest or Reddit or "Sina Weibo" or Snapchat or online social network* or social networking platform* or social networking site* or social networking website* or social platform* or TikTok or Tumblr or Twitter or "web 2.0" or "web 2.0s" or WeChat or WhatsApp or YouTube).tw,kf.	23236
146	Virtual Reality/	4660
147	(virtual reality or virtual realities).tw,kf.	11072
148	Augmented Reality/	874
149	(augmented realities or augmented reality or mixed realities or mixed reality).tw,kf.	2555

150	holography/	2140
151	(hologram* or holograph*).tw,kf.	2914
152	Printing, Three-Dimensional/	10196
153	("3d printing*" or "3 d printing*" or "3 dimensional printing*" or "three dimensional printing*").tw,kf.	8138
154	(chatbot or chatbots or "chat bot" or "chat bots").tw,kf.	286
155	"virtual care".tw,kf.	562
156	(closed loop medicines system* or closed loop medication system* or closed loop medicines process* or closed loop medication process*).tw,kf.	2
157	(bedside station or bedside stations or bed side station or bed side stations or bedside terminal or bedside terminals or bed side terminal or bed side terminals).tw,kf.	55
158	predictive analytic*.tw,kf.	591
159	or/27-158	656103
160	26 and 159	120231
161	(champion or champions).tw,kf.	4380
162	(change agent or change agents).tw,kf.	990
163	(formal leader or formal leaders or informal leader or informal leaders or opinion leader or opinion leaders).tw,kf.	1525
164	(superuser or superusers or super user or super users).tw,kf.	86
165	(boundary spanner or boundary spanners).tw,kf.	69
166	(advocate or advocates).tw,kf.	30769
167	(supporter or supporters).tw,kf.	3062
168	mentors/	12500
169	Mentoring/	3373
170	(mentor or mentors).tw,kf.	8485
171	(coach or coaches).tw,kf.	8264
172	Peer Group/ or Peer Influence/	23959
173	("peer to peer" or peer influence).tw,kf.	2527
174	((peer or peers) adj3 collaborat*).tw,kf.	348
175	interpersonal influence*.tw,kf.	212
176	(innovator or innovators).tw,kf.	2034
177	(ambassador or ambassadors).tw,kf.	520
178	or/161-177	93154
179	160 and 178	2228
180	limit 179 to english language	2195
181	limit 180 to yr="2017 -Current"	1075
182	remove duplicates from 181	1069

Database: MEDLINE Epub Ahead of Print and In-Process
Search strategy:

#	Searches	Results
1	(nurse or nurses or nursing).tw,kf.	45295
2	(health personnel or health employee* or healthcare employee* or health care employee* or healthcare professional* or health care professional* or healthcare provider* or health care provider* or healthcare personnel or health care personnel or healthcare worker* or health care worker* or medical staff).tw,kf.	29833
3	(doctor or doctors or hospital registrar* or physician* or allergist* or anesthesiologist* or cardiologist* or dermatologist* or endocrinologist* or gastroenterologist* or general practitioner* or geriatrician* or hospitalist* or nephrologist* or neurologist* or oncologist* or ophthalmologist* or osteopathic physician* or otolaryngologist* or pathologist* or paediatrician* or pediatrician* or neonatologist* or pharmacist or pharmacists or physiatrist* or pulmonologist* or radiologist* or rheumatologist* or surgeon* or neurosurgeon* or urologist* or psychotherapist*).tw,kf.	138905
4	(occupational therapist* or physical therapist*).tw,kf.	2243
5	(dietician or dietitian or nutritionist or dieticians or dietitians or nutritionists).tw,kf.	1525
6	(health team or health teams or healthcare team* or health care team* or interdisciplinary care team* or medical care team or medical care teams or multidisciplinary care team* or patient care team or patient care teams).tw,kf.	2154
7	(case worker or case workers or caseworker or caseworkers or social worker or social workers).tw,kf.	1326
8	(respiratory care practitioner or respiratory care practitioners or respiratory therapist or respiratory therapists).tw,kf.	231
9	(logopedist or logopedists or "speech and language pathologist" or "speech and language pathologist*" or speech language pathologist or speech language pathologists or speech therapist or speech therapists or speech language therapist or speech language therapists).tw,kf.	530
10	or/1-9	201141
11	(telemedicine or telecardiology or teledermatology or telediagnosis or telediagnoses or telehealth or telemonitoring or telenephrology or teleneurology or telepsychology or teleradiology or teleradiotherap* or telesurger* or teletherap* or videoconsult*).tw,kf.	7212
12	("tele medicine" or "tele cardiology" or "tele dermatology" or "tele diagnosis" or "tele diagnoses" or "tele monitoring" or "tele nephrology" or "tele neurology" or "tele psychology" or "tele radiology" or "tele radiotherap*" or "tele surger*" or "tele therap*" or "video consult*").tw,kf.	307
13	("e-health" or ehealth or "tele health").tw,kf.	2428
14	(telenurs* or "tele nurs*").tw,kf.	57
15	("cyber consult*" or cyberconsult* or econsult* or "e−consult*" or "email based consult*" or "internet consult*" or "internet based consult*" or "online consult*" or "tele consult*" or "telephone based consult*" or "telephone consult*" or "virtual consult*" or "web consult*" or "webbased consult*" or "web based consult*").tw,kf.	484
16	("e−rehabilitation" or remote rehabilitation or tele rehabilitation or virtual rehabilitation).tw,kf.	135
17	(computerised medical record system* or computerized medical record system*).tw,kf.	16

18	(electronic health record* or computerised patient record* or computerized patient record* or electronic medical record* or electronic patient record* or ehr or emr).tw,kf.	9978
19	(smart card or smart cards).tw,kf.	59
20	medical order entry system*.tw,kf.	26
21	(hospital information system* or patient health record information system*).tw,kf.	404
22	ambulatory care information system*.tw,kf.	6
23	("e−prescribing" or "e−prescription*" or electronic prescription*).tw,kf.	95
24	("c.p.o.e. system" or computer* order entry or computer* physician order entry or computer* provider order entry or computer* order entry or computer* prescriber order entry or computer* prescribing order entry or computer* prescription order entry or "cpoe").tw,kf.	220
25	(operating room information system* or operating room information management system*).tw,kf.	3
26	(bedside computing or bedside technolog* or point of care system* or point of care technolog* or point of care information system*).tw,kf.	163
27	bedside information system*.tw,kf.	0
28	(management information system* or management information).tw,kf.	293
29	clinical pharmacy information system*.tw,kf.	10
30	(database management system* or database management tool or database management tools or database manager system* or database managing system* or data base manager system* or data base management software or data base management system* or data base management tool or data base management tools).tw,kf.	97
31	decision support system*.tw,kf.	1305
32	(mobile app or mobile apps or mobile application* or mobile health app or mobile health apps or mobile health application* or portable software app or portable software apps or portable software application* or tablet app or tablet apps or tablet application*).tw,kf.	3127
33	(cell phone or cell phones or cellphone or cellphones or cellular phone or cellular phones or cellular telephone or cellular telephones or mobile phone or mobile phones or mobile telephone or mobile telephones).tw,kf.	4479
34	(smartphone or smartphones or smart phones or smart phones).tw,kf.	6065
35	(text messaging or texting).tw,kf.	838
36	(video conference* or video conferencing or videoconferenc*).tw,kf.	1025
37	(webcast* or web cast*).tw,kf.	55
38	(wireless communication* or wireless technol*).tw,kf.	1508
39	(hand held computer* or handheld computer* or palm PC or palmtop or personal data assistant* or personal digital assistant or pocket computer* or pocket sized computer*).tw,kf.	124
40	internet.tw,kf.	14833
41	(internet based intervention* or online based intervention* or online intervention* or web intervention* or web based intervention*).tw,kf.	984
42	smart technolog*.tw,kf.	136

43	(wearable computer or wearable computers or wearable electronic device*).tw,kf.	361
44	(activity tracker* or fitness tracker* or smart watch or smart watches or pedometer*).tw,kf.	811
45	("ar glasses" or "ar head mounted device" or "ar head mounted display" or "ar head worn display" or "ar headset" or "ar hud" or "arhmd" or "hmd ar" or "optical see through head mounted display" or "ost hmd" or "see through hmd" or "google glasses" or "smartglasses" or "smart glasses").tw,kf.	116
46	(augmented reality glasses or augmented reality head mounted device or augmented reality head mounted display or augmented reality head up display or augmented reality head worn display or head mounted display augmented reality).tw,kf.	16
47	(artificial intelligence or machine intelligence).tw,kf.	9001
48	computer heuristic*.tw,kf.	0
49	(expert system* or knowledge based system*).tw,kf.	467
50	(fuzzy logic or fuzzy model or fuzzy models).tw,kf.	1032
51	(machine learning or learning machine*).tw,kf.	23483
52	(deep learning or hierarchical learning).tw,kf.	12952
53	unsupervised machine learning.tw,kf.	343
54	natural language processing.tw,kf.	1773
55	("ann approach*" or "ann model" or "ann method*" or "ann training" or artificial neural network or computer neural network or computer neural networks).tw,kf.	2802
56	(robotics or nanorobotics or robot or robots).tw,kf.	11962
57	(biomedical technology or bio medical technology).tw,kf.	102
58	informatics.tw,kf.	3419
59	(clinical information science or clinical information technology or health information science or health information technology or medical computer science or medical information science or medical information technology).tw,kf.	674
60	health information exchange.tw,kf.	211
61	(decision support system* or decision support techniques).tw,kf.	1435
62	(automatic diagnosis or computer assisted diagnosis or computer diagnosis or automatic diagnoses or computer assisted diagnoses or computer diagnoses).tw,kf.	247
63	image interpretation, computer-assisted/	1
64	computer assisted image interpretation.tw,kf.	4
65	computer assisted radiographic image interpretation*.tw,kf.	10
66	computer assisted therap*.tw,kf.	25
67	computer assisted drug therap*.tw,kf.	1
68	clinical decision support system*.tw,kf.	528
69	(information system or information systems).tw,kf.	5303
70	(online system or online systems).tw,kf.	169
71	(computer interface* or computer user interface*).tw,kf.	1591
72	(social media or social medium or Facebook or Flickr or Instagram or LinkedIn or MySpace or Pinterest or Reddit or "Sina Weibo" or Snapchat or online social network* or social networking platform* or social networking site* or social networking website*	9732

	or social platform* or TikTok or Tumblr or Twitter or "web 2.0" or "web 2.0s" or WeChat or WhatsApp or YouTube).tw,kf.	
73	(virtual reality or virtual realities).tw,kf.	3550
74	(augmented realities or augmented reality or mixed realities or mixed reality).tw,kf.	1390
75	(hologram* or holograph*).tw,kf.	9193
76	("3d printing*" or "3 d printing*" or "3 dimensional printing*" or "three dimensional printing*").tw,kf.	6211
77	(chatbot or chatbots or "chat bot" or "chat bots").tw,kf.	226
78	"virtual care".tw,kf.	295
79	(closed loop medicines system* or closed loop medication system* or closed loop medicines process* or closed loop medication process*).tw,kf.	0
80	(bedside station or bedside stations or bed side station or bed side stations or bedside terminal or bedside terminals or bed side terminal or bed side terminals).tw,kf.	1
81	predictive analytic*.tw,kf.	277
82	or/11-81	131558
83	10 and 82	16836
84	(champion or champions).tw,kf.	1357
85	(change agent or change agents).tw,kf.	153
86	(formal leader or formal leaders or informal leader or informal leaders or opinion leader or opinion leaders).tw,kf.	247
87	(superuser or superusers or super user or super users).tw,kf.	27
88	(boundary spanner or boundary spanners).tw,kf.	12
89	(advocate or advocates).tw,kf.	5158
90	(supporter or supporters).tw,kf.	670
91	(mentor or mentors).tw,kf.	1679
92	(coach or coaches).tw,kf.	2601
93	("peer to peer" or peer influence).tw,kf.	688
94	((peer or peers) adj3 collaborat*).tw,kf.	83
95	interpersonal influence*.tw,kf.	44
96	(innovator or innovators).tw,kf.	408
97	(ambassador or ambassadors).tw,kf.	148
98	or/84-97	12982
99	83 and 98	319
100	limit 99 to english language	319
101	limit 100 to yr="2017 -Current"	258

Database: Embase
Search strategy:

#	Searches	Results

1	nurse/ or expert nurse/ or licensed practical nurse/ or nurse consultant/ or practical nurse/ or registered nurse/ or staff nurse/	154224
2	nursing/ or practical nursing/ or telenursing/ or travel nursing/	236859
3	advanced practice nurse/	1013
4	nurse administrator/ or charge nurse/ or head nurse/ or nurse manager/	15481
5	nurse practitioner/ or acute care nurse practitioner/ or adult nurse practitioner/ or emergency nurse practitioner/ or family nurse practitioner/ or gerontologic nurse practitioner/ or infection control practitioner/ or neonatal nurse practitioner/ or pediatric nurse practitioner/	28694
6	nurse specialist/ or clinical nurse specialist/ or neonatal nurse/ or nurse anesthetist/ or nurse midwife/ or oncology nurse/ or pediatric nurse/	15218
7	nursing staff/	76745
8	nursing care/ or visiting nursing service/	38268
9	nursing discipline/ or acquired immune deficiency syndrome nursing/ or addictions nursing/ or ambulatory care nursing/ or anesthesia nursing/ or burn nursing/ or camp nursing/ or cardiovascular nursing/ or dermatology nursing/ or emergency nursing/ or enterostomal therapy nursing/ or family nursing/ or flight nursing/ or forensic nursing/ or gastroenterology nursing/ or genetics nursing/ or gynecologic nursing/ or hospice nursing/ or intravenous nursing/ or learning disability nursing/ or military nursing/ or nephrology nursing/ or neuroscience nursing/ or nurse midwifery/ or obstetrical nursing/ or occupational health nursing/ or ophthalmic nursing/ or orthopedic nursing/ or palliative nursing/ or parish nursing/ or perinatal nursing/ or perioperative nursing/ or postanesthesia nursing/ or prison nursing/ or radiology nursing/ or rehabilitation nursing/ or respiratory nursing/ or rural health nursing/ or school health nursing/ or urologic nursing/	46201
10	community health nursing/ or community psychiatric nursing/	27005
11	community health nursing/ or community psychiatric nursing/	27005
12	geriatric nursing/ or psychogeriatric nursing/	13103
13	intensive care nursing/ or newborn intensive care nursing/ or pediatric intensive care nursing/	3100
14	newborn nursing/	3924
15	oncology nursing/ or pediatric oncology nursing/	8475
16	pediatric nursing/	13215
17	psychiatric nursing/	17005
18	surgical nursing/ or plastic surgical nursing/	284
19	advanced practice nursing/	2168
20	practical nursing/	159
21	nursing role/	677
22	nursing process/	7106
23	nursing assessment/	27412
24	nursing diagnosis/	4743
25	team nursing/	444

26	(nurse or nurses or nursing).tw,kw.	591830
27	exp health care personnel/	1891206
28	(health personnel or health employee* or healthcare employee* or health care employee* or healthcare professional* or health care professional* or healthcare provider* or health care provider* or healthcare personnel or health care personnel or healthcare worker* or health care worker* or medical staff).tw,kw.	244437
29	(doctor or doctors or hospital registrar* or physician* or allergist* or anesthesiologist* or cardiologist* or dermatologist* or endocrinologist* or gastroenterologist* or general practitioner* or geriatrician* or hospitalist* or nephrologist* or neurologist* or oncologist* or ophthalmologist* or osteopathic physician* or otolaryngologist* or pathologist* or paediatrician* or pediatrician* or neonatologist* or pharmacist or pharmacists or physiatrist* or pulmonologist* or radiologist* or rheumatologist* or surgeon* or neurosurgeon* or urologist* or psychotherapist*).tw,kw.	1667807
30	(occupational therapist* or physical therapist*).tw,kw.	21322
31	(health team or health teams or healthcare team* or health care team* or interdisciplinary care team* or medical care team or medical care teams or multidisciplinary care team* or patient care team or patient care teams).tw,kw.	19866
32	dietitian/	15342
33	(dietician or dietitian or nutritionist or dieticians or dietitians or nutritionists).tw,kw.	22086
34	social worker/	15571
35	(case worker or case workers or caseworker or caseworkers or social worker or social workers).tw,kw.	20300
36	respiratory therapist/	2830
37	(respiratory care practitioner or respiratory care practitioners or respiratory therapist or respiratory therapists).tw,kw.	3254
38	speech language pathologist/	3058
39	(logopedist or logopedists or "speech and language pathologist" or "speech and language pathologist*" or speech language pathologist or speech language pathologists or speech therapist or speech therapists or speech language therapist or speech language therapists).tw,kw.	6805
40	or/1-39	3279844
41	telemedicine/ or telecardiology/ or teledermatology/ or telediagnosis/ or telemonitoring/ or telenephrology/ or teleneurology/ or telepsychology/ or teleradiology/ or teleradiotherapy/ or telesurgery/ or teletherapy/ or video consultation/	47820
42	(telemedicine or telecardiology or teledermatology or telediagnosis or telediagnoses or telehealth or telemonitoring or telenephrology or teleneurology or telepsychology or teleradiology or teleradiotherap* or telesurger* or teletherap* or videoconsult*).tw,kw.	44914
43	("tele medicine" or "tele cardiology" or "tele dermatology" or "tele diagnosis" or "tele diagnoses" or "tele monitoring" or "tele nephrology" or "tele neurology" or "tele psychology" or "tele radiology" or "tele radiotherap*" or "tele surger*" or "tele therap*" or "video consult*").tw,kw.	2080
44	telehealth/	14232

45	("e-health" or ehealth or "tele health").tw,kw.	11006
46	telenursing/	339
47	(telenurs* or "tele nurs*").tw,kw.	349
48	teleconsultation/ or electronic consultation/	14038
49	("cyber consult*" or cyberconsult* or econsult* or "e−consult*" or "email based consult*" or "internet consult*" or "internet based consult*" or "online consult*" or "tele consult*" or "telephone based consult*" or "telephone consult*" or "virtual consult*" or "web consult*" or "webbased consult*" or "web based consult*").tw,kw.	3870
50	telerehabilitation/	1900
51	("e−rehabilitation" or remote rehabilitation or tele rehabilitation or virtual rehabilitation).tw,kw.	719
52	electronic medical record system/	2097
53	(computerised medical record system* or computerized medical record system*).tw,kw.	171
54	electronic health record/ or electronic medical record/ or electronic patient record/	103544
55	(electronic health record* or computerised patient record* or computerized patient record* or electronic medical record* or electronic patient record* or ehr or emr).tw,kw.	105800
56	smart card/	274
57	(smart card or smart cards).tw,kw.	456
58	physician order entry system/	331
59	medical order entry system*.tw,kw.	156
60	hospital information system/	20886
61	(hospital information system* or patient health record information system*).tw,kw.	4678
62	ambulatory care information system*.tw,kw.	39
63	electronic prescribing/	3823
64	("e−prescribing" or "e−prescription*" or electronic prescription*).tw,kw.	1416
65	computerized provider order entry/	2161
66	("c.p.o.e. system" or computer* order entry or computer* physician order entry or computer* provider order entry or computer* order entry or computer* prescriber order entry or computer* prescribing order entry or computer* prescription order entry or "cpoe").tw,kw.	3110
67	operating room information system/	39
68	(operating room information system* or operating room information management system*).tw,kw.	57
69	"point of care system"/	3370
70	(bedside computing or bedside technolog* or point of care system* or point of care technolog* or point of care information system*).tw,kw.	1257
71	bedside information system/	16
72	bedside information system*.tw,kw.	11
73	information system/	41287
74	(management information system* or management information).tw,kw.	2982

75	medical information system/	22766
76	clinical pharmacy information system*.tw,kw.	36
77	database management system/	653
78	(database management system* or database management tool or database management tools or database manager system* or database managing system* or data base manager system* or data base management software or data base management system* or data base management tool or data base management tools).tw,kw.	928
79	decision support system/	25559
80	decision support system*.tw,kw.	9246
81	mobile application/ or mobile health application/	20932
82	(mobile app or mobile apps or mobile application* or mobile health app or mobile health apps or mobile health application* or portable software app or portable software apps or portable software application* or tablet app or tablet apps or tablet application*).tw,kw.	11116
83	mobile phone/	20418
84	(cell phone or cell phones or cellphone or cellphones or cellular phone or cellular phones or cellular telephone or cellular telephones or mobile phone or mobile phones or mobile telephone or mobile telephones).tw,kw.	19926
85	smartphone/	21299
86	(smartphone or smartphones or smart phone or smart phones).tw,kw.	26526
87	text messaging/	6801
88	(text messaging or texting).tw,kw.	4612
89	videoconferencing/	7295
90	(video conference* or video conferencing or videoconferenc*).tw,kw.	6517
91	webcast/	422
92	(webcast* or web cast*).tw,kw.	489
93	wireless communication/	6759
94	(wireless communication* or wireless technol*).tw,kw.	3077
95	"cell phone use"/	1465
96	personal digital assistant/	1727
97	(hand held computer* or handheld computer* or palm PC or palmtop or personal data assistant* or personal digital assistant or pocket computer* or pocket sized computer*).tw,kw.	1781
98	internet/	118180
99	internet.tw,kw.	87020
100	web-based intervention/	1860
101	(internet based intervention* or online based intervention* or online intervention* or web intervention* or web based intervention*).tw,kw.	4081
102	smart technolog*.tw,kw.	500
103	wearable computer/	1007
104	(wearable computer or wearable computers or wearable electronic device*).tw,kw.	548

105	smart watch/ or activity tracker/	2172
106	(activity tracker* or fitness tracker* or smart watch or smart watches or pedometer*).tw,kw.	5824
107	smart glasses/	213
108	("ar glasses" or "ar head mounted device" or "ar head mounted display" or "ar head worn display" or "ar headset" or "ar hud" or "arhmd" or "hmd ar" or "optical see through head mounted display" or "ost hmd" or "see through hmd" or "google glasses" or "smartglasses" or "smart glasses").tw,kw.	377
109	(augmented reality glasses or augmented reality head mounted device or augmented reality head mounted display or augmented reality head up display or augmented reality head worn display or head mounted display augmented reality).tw,kw.	73
110	artificial intelligence/	44391
111	(artificial intelligence or machine intelligence).tw,kw.	29539
112	computer heuristics/	372
113	computer heuristic*.tw,kw.	1
114	expert system/	5647
115	(expert system* or knowledge based system*).tw,kw.	5174
116	fuzzy logic/	4661
117	(fuzzy logic or fuzzy model or fuzzy models).tw,kw.	3539
118	machine learning/	65473
119	(machine learning or learning machine*).tw,kw.	79956
120	deep learning/	27376
121	(deep learning or hierarchical learning).tw,kw.	38617
122	unsupervised machine learning/	1783
123	unsupervised machine learning.tw,kw.	1171
124	natural language processing/	8228
125	natural language processing.tw,kw.	7188
126	artificial neural network/	44155
127	("ann approach*" or "ann model" or "ann method*" or "ann training" or artificial neural network or computer neural network or computer neural networks).tw,kw.	12250
128	robotics/ or nanorobotics/	44566
129	(robotics or nanorobotics or robot or robots).tw,kw.	48549
130	medical technology/	36849
131	(biomedical technology or bio medical technology).tw,kw.	637
132	information science/	13315
133	informatics.tw,kw.	19210
134	medical informatics/	22345
135	(clinical information science or clinical information technology or health information science or health information technology or medical computer science or medical information science or medical information technology).tw,kw.	4166
136	nursing informatics/	1670

137	health information exchange.tw,kw.	1304
138	(decision support system* or decision support techniques).tw,kw.	9970
139	computer assisted diagnosis/	43067
140	(automatic diagnosis or computer assisted diagnosis or computer diagnosis or automatic diagnoses or computer assisted diagnoses or computer diagnoses).tw,kw.	2140
141	computer assisted image interpretation.tw,kw.	49
142	computer assisted radiographic image interpretation*.tw,kw.	30
143	computer assisted therapy/	4816
144	computer assisted therap*.tw,kw.	161
145	computer assisted drug therapy/	935
146	computer assisted drug therap*.tw,kw.	36
147	clinical decision support system/	4838
148	clinical decision support system*.tw,kw.	3623
149	information system/	41287
150	(information system or information systems).tw,kw.	49943
151	online system/	29690
152	(online system or online systems).tw,kw.	1090
153	computer interface/	34806
154	(computer interface* or computer user interface*).tw,kw.	7794
155	social media/	37588
156	(social media or social medium or Facebook or Flickr or Instagram or LinkedIn or MySpace or Pinterest or Reddit or "Sina Weibo" or Snapchat or online social network* or social networking platform* or social networking site* or social networking website* or social platform* or TikTok or Tumblr or Twitter or "web 2.0" or "web 2.0s" or WeChat or WhatsApp or YouTube).tw,kw.	41751
157	virtual reality/	22845
158	(virtual reality or virtual realities).tw,kw.	18403
159	augmented reality/	1474
160	(augmented realities or augmented reality or mixed realities or mixed reality).tw,kw.	4554
161	holography/	4361
162	(hologram* or holograph*).tw,kw.	7219
163	three dimensional printing/	18816
164	("3d printing*" or "3 d printing*" or "3 dimensional printing*" or "three dimensional printing*").tw,kw.	14841
165	(chatbot or chatbots or "chat bot" or "chat bots").tw,kw.	502
166	"virtual care".tw,kw.	998
167	(closed loop medicines system* or closed loop medication system* or closed loop medicines process* or closed loop medication process*).tw,kw.	7
168	(bedside station or bedside stations or bed side station or bed side stations or bedside terminal or bedside terminals or bed side terminal or bed side terminals).tw,kw.	57
169	predictive analytic?.tw,kw.	1070

170	or/41-169	1016835
171	40 and 170	239803
172	(champion or champions).tw,kw.	8417
173	(change agent or change agents).tw,kw.	1333
174	(formal leader or formal leaders or informal leader or informal leaders or opinion leader or opinion leaders).tw,kw.	2390
175	(superuser or superusers or super user or super users).tw,kw.	250
176	(boundary spanner or boundary spanners).tw,kw.	83
177	(advocate or advocates).tw,kw.	49019
178	(supporter or supporters).tw,kw.	4684
179	mentor/	9020
180	mentoring/	5515
181	(mentor or mentors).tw,kw.	13413
182	(coach or coaches).tw,kw.	12898
183	Peer Group/	28344
184	("peer to peer" or peer influence).tw,kw.	4122
185	((peer or peers) adj3 collaborat*).tw,kw.	523
186	interpersonal influence*.tw,kw.	296
187	(innovator or innovators).tw,kw.	3544
188	(ambassador or ambassadors).tw,kw.	987
189	or/172-188	132931
190	171 and 189	5440
191	limit 190 to english language	5392
192	limit 191 to yr="2017 -Current"	3224
193	limit 192 to (books or chapter or conference abstract or conference paper or "conference review" or editorial or letter)	1420
194	192 not 193	1804

Database: Emcare Nursing
Search strategy:

#	Searches	Results
1	nurse/ or expert nurse/ or licensed practical nurse/ or nurse consultant/ or practical nurse/ or registered nurse/ or staff nurse/	146685
2	nursing/ or practical nursing/ or telenursing/ or travel nursing/	99789
3	advanced practice nurse/	2300
4	nurse administrator/ or charge nurse/ or head nurse/ or nurse manager/	8211
5	nurse practitioner/ or acute care nurse practitioner/ or adult nurse practitioner/ or emergency nurse practitioner/ or family nurse practitioner/ or gerontologic nurse	15608

	practitioner/ or infection control practitioner/ or neonatal nurse practitioner/ or pediatric nurse practitioner/	
6	nurse specialist/ or clinical nurse specialist/ or neonatal nurse/ or nurse anesthetist/ or nurse midwife/ or oncology nurse/ or pediatric nurse/	6354
7	nursing staff/	26910
8	nursing care/ or visiting nursing service/	18332
9	nursing discipline/ or acquired immune deficiency syndrome nursing/ or addictions nursing/ or ambulatory care nursing/ or anesthesia nursing/ or burn nursing/ or camp nursing/ or cardiovascular nursing/ or dermatology nursing/ or emergency nursing/ or enterostomal therapy nursing/ or family nursing/ or flight nursing/ or forensic nursing/ or gastroenterology nursing/ or genetics nursing/ or gynecologic nursing/ or hospice nursing/ or intravenous nursing/ or learning disability nursing/ or military nursing/ or nephrology nursing/ or neuroscience nursing/ or nurse midwifery/ or obstetrical nursing/ or occupational health nursing/ or ophthalmic nursing/ or orthopedic nursing/ or palliative nursing/ or parish nursing/ or perinatal nursing/ or perioperative nursing/ or postanesthesia nursing/ or prison nursing/ or radiology nursing/ or rehabilitation nursing/ or respiratory nursing/ or rural health nursing/ or school health nursing/ or urologic nursing/	15019
10	community health nursing/ or community psychiatric nursing/	5317
11	community health nursing/ or community psychiatric nursing/	5317
12	geriatric nursing/ or psychogeriatric nursing/	2384
13	intensive care nursing/ or newborn intensive care nursing/ or pediatric intensive care nursing/	2047
14	newborn nursing/	2251
15	oncology nursing/ or pediatric oncology nursing/	2581
16	pediatric nursing/	2354
17	psychiatric nursing/	4356
18	surgical nursing/ or plastic surgical nursing/	465
19	advanced practice nursing/	1618
20	practical nursing/	190
21	nursing role/	1914
22	nursing process/	1765
23	nursing assessment/	4732
24	nursing diagnosis/	1731
25	team nursing/	184
26	(nurse or nurses or nursing).tw,kw.	297956
27	exp health care personnel/	823051
28	(health personnel or health employee* or healthcare employee* or health care employee* or healthcare professional* or health care professional* or healthcare provider* or health care provider* or healthcare personnel or health care personnel or healthcare worker* or health care worker* or medical staff).tw,kw.	113390
29	(doctor or doctors or hospital registrar* or physician* or allergist* or anesthesiologist* or cardiologist* or dermatologist* or endocrinologist* or gastroenterologist* or	466345

	general practitioner* or geriatrician* or hospitalist* or nephrologist* or neurologist* or oncologist* or ophthalmologist* or osteopathic physician* or otolaryngologist* or pathologist* or paediatrician* or pediatrician* or neonatologist* or pharmacist or pharmacists or physiatrist* or pulmonologist* or radiologist* or rheumatologist* or surgeon* or neurosurgeon* or urologist* or psychotherapist*).tw,kw.	
30	(occupational therapist* or physical therapist*).tw,kw.	12755
31	(health team or health teams or healthcare team* or health care team* or interdisciplinary care team* or medical care team or medical care teams or multidisciplinary care team* or patient care team or patient care teams).tw,kw.	10116
32	dietitian/	6360
33	(dietician or dietitian or nutritionist or dieticians or dietitians or nutritionists).tw,kw.	7640
34	social worker/	11064
35	(case worker or case workers or caseworker or caseworkers or social worker or social workers).tw,kw.	12900
36	respiratory therapist/	1256
37	(respiratory care practitioner or respiratory care practitioners or respiratory therapist or respiratory therapists).tw,kw.	1069
38	speech language pathologist/	3472
39	(logopedist or logopedists or "speech and language pathologist" or "speech and language pathologist*" or speech language pathologist or speech language pathologists or speech therapist or speech therapists or speech language therapist or speech language therapists).tw,kw.	4339
40	or/1-39	1172394
41	telemedicine/ or telecardiology/ or teledermatology/ or telediagnosis/ or telemonitoring/ or telenephrology/ or teleneurology/ or telepsychology/ or teleradiology/ or teleradiotherapy/ or telesurgery/ or teletherapy/ or video consultation/	16825
42	(telemedicine or telecardiology or teledermatology or telediagnosis or telediagnoses or telehealth or telemonitoring or telenephrology or teleneurology or telepsychology or teleradiology or teleradiotherap* or telesurger* or teletherap* or videoconsult*).tw,kw.	20331
43	("tele medicine" or "tele cardiology" or "tele dermatology" or "tele diagnosis" or "tele diagnoses" or "tele monitoring" or "tele nephrology" or "tele neurology" or "tele psychology" or "tele radiology" or "tele radiotherap*" or "tele surger*" or "tele therap*" or "video consult*").tw,kw.	737
44	telehealth/	9507
45	("e-health" or ehealth or "tele health").tw,kw.	7333
46	telenursing/	308
47	(telenurs* or "tele nurs*").tw,kw.	351
48	teleconsultation/ or electronic consultation/	5284
49	("cyber consult*" or cyberconsult* or econsult* or "e−consult*" or "email based consult*" or "internet consult*" or "internet based consult*" or "online consult*" or	1274

	"tele consult*" or "telephone based consult*" or "telephone consult*" or "virtual consult*" or "web consult*" or "webbased consult*" or "web based consult*").tw,kw.	
50	telerehabilitation/	797
51	("e−rehabilitation" or remote rehabilitation or tele rehabilitation or virtual rehabilitation).tw,kw.	355
52	electronic medical record system/	539
53	(computerised medical record system* or computerized medical record system*).tw,kw.	108
54	electronic health record/ or electronic medical record/ or electronic patient record/	32253
55	(electronic health record* or computerised patient record* or computerized patient record* or electronic medical record* or electronic patient record* or ehr or emr).tw,kw.	31391
56	smart card/	262
57	(smart card or smart cards).tw,kw.	284
58	physician order entry system/	68
59	medical order entry system*.tw,kw.	151
60	hospital information system/	6877
61	(hospital information system* or patient health record information system*).tw,kw.	1960
62	ambulatory care information system*.tw,kw.	22
63	electronic prescribing/	1321
64	("e−prescribing" or "e−prescription*" or electronic prescription*).tw,kw.	464
65	computerized provider order entry/	1226
66	("c.p.o.e. system" or computer* order entry or computer* physician order entry or computer* provider order entry or computer* order entry or computer* prescriber order entry or computer* prescribing order entry or computer* prescription order entry or "cpoe").tw,kw.	1540
67	operating room information system/	17
68	(operating room information system* or operating room information management system*).tw,kw.	22
69	"point of care system"/	452
70	(bedside computing or bedside technolog* or point of care system* or point of care technolog* or point of care information system*).tw,kw.	497
71	bedside information system*.tw,kw.	6
72	information system/	18523
73	(management information system* or management information).tw,kw.	1211
74	medical information system/	13495
75	clinical pharmacy information system*.tw,kw.	21
76	database management system/	107
77	(database management system* or database management tool or database management tools or database manager system* or database managing system* or data base manager system* or data base management software or data base	282

	management system* or data base management tool or data base management tools).tw,kw.	
78	decision support system/	10859
79	decision support system*.tw,kw.	4454
80	mobile application/ or mobile health application/	6990
81	(mobile app or mobile apps or mobile application* or mobile health app or mobile health apps or mobile health application* or portable software app or portable software apps or portable software application* or tablet app or tablet apps or tablet application*).tw,kw.	5475
82	mobile phone/	7757
83	(cell phone or cell phones or cellphone or cellphones or cellular phone or cellular phones or cellular telephone or cellular telephones or mobile phone or mobile phones or mobile telephone or mobile telephones).tw,kw.	9076
84	smartphone/	4789
85	(smartphone or smartphones or smart phone or smart phones).tw,kw.	10421
86	text messaging/	3167
87	(text messaging or texting).tw,kw.	2668
88	videoconferencing/	3004
89	(video conference* or video conferencing or videoconferenc*).tw,kw.	3146
90	webcast/	84
91	(webcast* or web cast*).tw,kw.	108
92	wireless communication/	1772
93	(wireless communication* or wireless technol*).tw,kw.	919
94	"cell phone use"/	689
95	personal digital assistant/	982
96	(hand held computer* or handheld computer* or palm PC or palmtop or personal data assistant* or personal digital assistant or pocket computer* or pocket sized computer*).tw,kw.	1033
97	internet/	54622
98	internet.tw,kw.	43370
99	web-based intervention/	800
100	(internet based intervention* or online based intervention* or online intervention* or web intervention* or web based intervention*).tw,kw.	2679
101	smart technolog*.tw,kw.	223
102	wearable computer/	49
103	(wearable computer or wearable computers or wearable electronic device*).tw,kw.	202
104	smart watch/ or activity tracker/	327
105	(activity tracker* or fitness tracker* or smart watch or smart watches or pedometer*).tw,kw.	2870
106	smart glasses/	27
107	("ar glasses" or "ar head mounted device" or "ar head mounted display" or "ar head worn display" or "ar headset" or "ar hud" or "arhmd" or "hmd ar" or "optical see	121

	through head mounted display" or "ost hmd" or "see through hmd" or "google glasses" or "smartglasses" or "smart glasses").tw,kw.	
108	(augmented reality glasses or augmented reality head mounted device or augmented reality head mounted display or augmented reality head up display or augmented reality head worn display or head mounted display augmented reality).tw,kw.	24
109	artificial intelligence/	7615
110	(artificial intelligence or machine intelligence).tw,kw.	8431
111	computer heuristics/	97
112	computer heuristic*.tw,kw.	1
113	expert system/	1930
114	(expert system* or knowledge based system*).tw,kw.	1332
115	fuzzy logic/	733
116	(fuzzy logic or fuzzy model or fuzzy models).tw,kw.	960
117	machine learning/	13875
118	(machine learning or learning machine*).tw,kw.	22377
119	deep learning/	5753
120	(deep learning or hierarchical learning).tw,kw.	10680
121	unsupervised machine learning/	300
122	unsupervised machine learning.tw,kw.	273
123	natural language processing/	2602
124	natural language processing.tw,kw.	3255
125	artificial neural network/	7700
126	("ann approach*" or "ann model" or "ann method*" or "ann training" or artificial neural network or computer neural network or computer neural networks).tw,kw.	2844
127	robotics/ or nanorobotics/	10849
128	(robotics or nanorobotics or robot or robots).tw,kw.	10586
129	medical technology/	12680
130	(biomedical technology or bio medical technology).tw,kw.	264
131	information science/	6815
132	informatics.tw,kw.	9683
133	medical informatics/	11628
134	(clinical information science or clinical information technology or health information science or health information technology or medical computer science or medical information science or medical information technology).tw,kw.	2920
135	nursing informatics/	1196
136	health information exchange.tw,kw.	844
137	(decision support system* or decision support techniques).tw,kw.	4806
138	computer assisted diagnosis/	4367
139	(automatic diagnosis or computer assisted diagnosis or computer diagnosis or automatic diagnoses or computer assisted diagnoses or computer diagnoses).tw,kw.	633
140	computer assisted image interpretation.tw,kw.	25

141	computer assisted radiographic image interpretation*.tw,kw.	15
142	computer assisted therapy/	1253
143	computer assisted therap*.tw,kw.	102
144	computer assisted drug therapy/	166
145	computer assisted drug therap*.tw,kw.	16
146	clinical decision support system/	1835
147	clinical decision support system*.tw,kw.	2012
148	information system/	18523
149	(information system or information systems).tw,kw.	20260
150	online system/	11099
151	(online system or online systems).tw,kw.	417
152	computer interface/	7316
153	(computer interface* or computer user interface*).tw,kw.	2435
154	social media/	17061
155	(social media or social medium or Facebook or Flickr or Instagram or LinkedIn or MySpace or Pinterest or Reddit or "Sina Weibo" or Snapchat or online social network* or social networking platform* or social networking site* or social networking website* or social platform* or TikTok or Tumblr or Twitter or "web 2.0" or "web 2.0s" or WeChat or WhatsApp or YouTube).tw,kw.	22978
156	virtual reality/	10864
157	(virtual reality or virtual realities).tw,kw.	8849
158	augmented reality/	432
159	(augmented realities or augmented reality or mixed realities or mixed reality).tw,kw.	1732
160	holography/	595
161	(hologram* or holograph*).tw,kw.	1073
162	three dimensional printing/	3505
163	("3d printing*" or "3 d printing*" or "3 dimensional printing*" or "three dimensional printing*").tw,kw.	3422
164	(chatbot or chatbots or "chat bot" or "chat bots").tw,kw.	287
165	"virtual care".tw,kw.	463
166	(closed loop medicines system* or closed loop medication system* or closed loop medicines process* or closed loop medication process*).tw,kw.	2
167	(bedside station or bedside stations or bed side station or bed side stations or bedside terminal or bedside terminals or bed side terminal or bed side terminals).tw,kw.	8
168	predictive analytic?.tw,kw.	419
169	or/41-168	340652
170	40 and 169	95399
171	(champion or champions).tw,kw.	3466
172	(change agent or change agents).tw,kw.	879
173	(formal leader or formal leaders or informal leader or informal leaders or opinion leader or opinion leaders).tw,kw.	1018

174	(superuser or superusers or super user or super users).tw,kw.	83
175	(boundary spanner or boundary spanners).tw,kw.	73
176	(advocate or advocates).tw,kw.	18738
177	(supporter or supporters).tw,kw.	2207
178	mentor/	3857
179	mentoring/	2694
180	(mentor or mentors).tw,kw.	6440
181	(coach or coaches).tw,kw.	9960
182	Peer Group/	12944
183	("peer to peer" or peer influence).tw,kw.	2426
184	((peer or peers) adj3 collaborat*).tw,kw.	306
185	interpersonal influence*.tw,kw.	273
186	(innovator or innovators).tw,kw.	950
187	(ambassador or ambassadors).tw,kw.	355
188	or/171-187	60893
189	170 and 188	1972
190	limit 189 to english language	1954
191	limit 190 to yr="2017 -Current"	1033
192	limit 191 to (books or chapter or conference abstract or conference paper or "conference review" or editorial or letter)	38
193	191 not 192	995

Database: Cochrane Central Register of Controlled Trials
Search strategy:

#	Searches	Results
1	nurses/ or nurse administrators/ or nurse practitioners/ or family nurse practitioners/ or pediatric nurse practitioners/ or nurse specialists/ or nurse anesthetists/ or nurse clinicians/ or nurse midwives/ or nurses, pediatric/ or nurses, neonatal/ or nurses, community health/ or nurses, public health/	1326
2	nursing staff/ or nursing staff, hospital/	682
3	nursing care/ or cardiovascular nursing/ or critical care nursing/ or developmental disability nursing/ or emergency nursing/ or geriatric nursing/ or holistic nursing/ or home nursing/ or "hospice and palliative care nursing"/ or maternal-child nursing/ or medical-surgical nursing/ or nephrology nursing/ or neuroscience nursing/ or nursing, practical/ or obstetric nursing/ or occupational health nursing/ or oncology nursing/ or orthopedic nursing/ or pediatric nursing/ or perioperative nursing/ or primary nursing/ or psychiatric nursing/ or rehabilitation nursing/ or trauma nursing/	1644
4	primary care nursing/	35
5	specialties, nursing/ or advanced practice nursing/ or cardiovascular nursing/ or community health nursing/ or home health nursing/ or parish nursing/ or critical care	2247

	nursing/ or developmental disability nursing/ or emergency nursing/ or family nursing/ or forensic nursing/ or geriatric nursing/ or holistic nursing/ or "hospice and palliative care nursing"/ or maternal-child nursing/ or neonatal nursing/ or medical-surgical nursing/ or midwifery/ or military nursing/ or nephrology nursing/ or neuroscience nursing/ or obstetric nursing/ or occupational health nursing/ or oncology nursing/ or orthopedic nursing/ or pediatric nursing/ or perioperative nursing/ or operating room nursing/ or postanesthesia nursing/ or psychiatric nursing/ or public health nursing/ or "radiologic and imaging nursing"/ or rehabilitation nursing/ or rural nursing/ or school nursing/ or transcultural nursing/ or trauma nursing/	
6	Nursing, Practical/	10
7	Nurse's Role/	386
8	nursing/ or nursing, private duty/ or nursing, supervisory/ or office nursing/ or telenursing/ or travel nursing/	123
9	nursing process/ or nursing assessment/ or nursing diagnosis/	568
10	nursing services/ or home care services/ or home health nursing/ or nursing service, hospital/	1968
11	Nursing, Team/	22
12	Nursing Faculty Practice/	4
13	(nurse or nurses or nursing).tw,kw.	45200
14	exp health personnel/	10409
15	(health personnel or health employee* or healthcare employee* or health care employee* or healthcare professional* or health care professional* or healthcare provider* or health care provider* or healthcare personnel or health care personnel or healthcare worker* or health care worker* or medical staff).tw,kw.	15119
16	(doctor or doctors or hospital registrar* or physician* or allergist* or anesthesiologist* or cardiologist* or dermatologist* or endocrinologist* or gastroenterologist* or general practitioner* or geriatrician* or hospitalist* or nephrologist* or neurologist* or oncologist* or ophthalmologist* or osteopathic physician* or otolaryngologist* or pathologist* or paediatrician* or pediatrician* or neonatologist* or pharmacist or pharmacists or physiatrist* or pulmonologist* or radiologist* or rheumatologist* or surgeon* or neurosurgeon* or urologist* or psychotherapist*).tw,kw.	118146
17	(occupational therapist* or physical therapist*).tw,kw.	3171
18	patient care team/	1754
19	(health team or health teams or healthcare team* or health care team* or interdisciplinary care team* or medical care team or medical care teams or multidisciplinary care team* or patient care team or patient care teams).tw,kw.	1311
20	Nutritionists/	56
21	(dietician or dietitian or nutritionist or dieticians or dietitians or nutritionists).tw,kw.	3694
22	Social Workers/	29
23	(case worker or case workers or caseworker or caseworkers or social worker or social workers).tw,kw.	1337
24	(respiratory care practitioner or respiratory care practitioners or respiratory therapist or respiratory therapists).tw,kw.	295

25	(logopedist or logopedists or "speech and language pathologist" or "speech and language pathologist*" or speech language pathologist or speech language pathologists or speech therapist or speech therapists or speech language therapist or speech language therapists).tw,kw.	456
26	or/1-25	174800
27	telemedicine/	2734
28	(telemedicine or telecardiology or teledermatology or telediagnosis or telediagnoses or telehealth or telemonitoring or telenephrology or teleneurology or telepsychology or teleradiology or teleradiotherap* or telesurger* or teletherap* or videoconsult*).tw,kw.	6097
29	("tele medicine" or "tele cardiology" or "tele dermatology" or "tele diagnosis" or "tele diagnoses" or "tele monitoring" or "tele nephrology" or "tele neurology" or "tele psychology" or "tele radiology" or "tele radiotherap*" or "tele surger*" or "tele therap*" or "video consult*").tw,kw.	280
30	("e-health" or ehealth or "tele health").tw,kw.	1383
31	(telenurs* or "tele nurs*").tw,kw.	156
32	remote consultation/	390
33	("cyber consult*" or cyberconsult* or econsult* or "e−consult*" or "email based consult*" or "internet consult*" or "internet based consult*" or "online consult*" or "tele consult*" or "telephone based consult*" or "telephone consult*" or "virtual consult*" or "web consult*" or "webbased consult*" or "web based consult*").tw,kw.	529
34	telerehabilitation/	169
35	("e−rehabilitation" or remote rehabilitation or tele rehabilitation or virtual rehabilitation).tw,kw.	277
36	medical records systems, computerized/	200
37	(computerised medical record system* or computerized medical record system*).tw,kw.	7
38	electronic health records/	458
39	(electronic health record* or computerised patient record* or computerized patient record* or electronic medical record* or electronic patient record* or ehr or emr).tw,kw.	6096
40	health smart cards/	1
41	(smart card or smart cards).tw,kw.	17
42	medical order entry systems/	71
43	medical order entry system*.tw,kw.	0
44	hospital information systems/	47
45	(hospital information system* or patient health record information system*).tw,kw.	123
46	ambulatory care information systems/	25
47	ambulatory care information system*.tw,kw.	1
48	electronic prescribing/	25
49	("e−prescribing" or "e−prescription*" or electronic prescription*).tw,kw.	51

50	("c.p.o.e. system" or computer* order entry or computer* physician order entry or computer* provider order entry or computer* order entry or computer* prescriber order entry or computer* prescribing order entry or computer* prescription order entry or "cpoe").tw,kw.	133
51	operating room information systems/	3
52	(operating room information system* or operating room information management system*).tw,kw.	2
53	Point-of-Care Systems/	473
54	(bedside computing or bedside technolog* or point of care system* or point of care technolog* or point of care information system*).tw,kw.	91
55	bedside information system*.tw,kw.	1
56	(management information system* or management information).tw,kw.	138
57	clinical pharmacy information systems/	21
58	clinical pharmacy information system*.tw,kw.	3
59	database management systems/	14
60	(database management system* or database management tool or database management tools or database manager system* or database managing system* or data base manager system* or data base management software or data base management system* or data base management tool or data base management tools).tw,kw.	15
61	decision support systems, management/	8
62	decision support system*.tw,kw.	1687
63	Mobile Applications/	1095
64	(mobile app or mobile apps or mobile application* or mobile health app or mobile health apps or mobile health application* or portable software app or portable software apps or portable software application* or tablet app or tablet apps or tablet application*).tw,kw.	3409
65	cell phone/	786
66	(cell phone or cell phones or cellphone or cellphones or cellular phone or cellular phones or cellular telephone or cellular telephones or mobile phone or mobile phones or mobile telephone or mobile telephones).tw,kw.	4275
67	smartphone/	664
68	(smartphone or smartphones or smart phone or smart phones).tw,kw.	6168
69	text messaging/	1127
70	(text messaging or texting).tw,kw.	2355
71	videoconferencing/	220
72	(video conference* or video conferencing or videoconferenc*).tw,kw.	1446
73	webcast/	0
74	webcasts as topic/	27
75	(webcast* or web cast*).tw,kw.	16
76	Wireless Technology/	48
77	(wireless communication* or wireless technol*).tw,kw.	142

78	"Cell Phone Use"/	12
79	computers, handheld/	310
80	(hand held computer* or handheld computer* or palm PC or palmtop or personal data assistant* or personal digital assistant or pocket computer* or pocket sized computer*).tw,kw.	260
81	internet/	4224
82	internet.tw,kw.	11145
83	internet-based intervention/	355
84	(internet based intervention* or online based intervention* or online intervention* or web intervention* or web based intervention*).tw,kw.	2886
85	smart technolog*.tw,kw.	25
86	wearable electronic devices/	138
87	(wearable computer or wearable computers or wearable electronic device*).tw,kw.	16
88	fitness trackers/	150
89	(activity tracker* or fitness tracker* or smart watch or smart watches or pedometer*).tw,kw.	2538
90	smart glasses/	8
91	("ar glasses" or "ar head mounted device" or "ar head mounted display" or "ar head worn display" or "ar headset" or "ar hud" or "arhmd" or "hmd ar" or "optical see through head mounted display" or "ost hmd" or "see through hmd" or "google glasses" or "smartglasses" or "smart glasses").tw,kw.	44
92	(augmented reality glasses or augmented reality head mounted device or augmented reality head mounted display or augmented reality head up display or augmented reality head worn display or head mounted display augmented reality).tw,kw.	8
93	artificial intelligence/	251
94	(artificial intelligence or machine intelligence).tw,kw.	959
95	computer heuristic*.tw,kw.	4
96	expert systems/	60
97	(expert system* or knowledge based system*).tw,kw.	195
98	fuzzy logic/	40
99	(fuzzy logic or fuzzy model or fuzzy models).tw,kw.	53
100	machine learning/	176
101	(machine learning or learning machine*).tw,kw.	2029
102	deep learning/	60
103	(deep learning or hierarchical learning).tw,kw.	781
104	unsupervised machine learning/	1
105	unsupervised machine learning.tw,kw.	30
106	natural language processing/	15
107	natural language processing.tw,kw.	218
108	neural networks, computer/	141

109	("ann approach*" or "ann model" or "ann method*" or "ann training" or artificial neural network or computer neural network or computer neural networks).tw,kw.	353
110	robotics/	748
111	(robotics or nanorobotics or robot or robots).tw,kw.	3797
112	biomedical technology/	22
113	(biomedical technology or bio medical technology).tw,kw.	377
114	informatics/	2
115	informatics.tw,kw.	558
116	medical informatics/	84
117	(clinical information science or clinical information technology or health information science or health information technology or medical computer science or medical information science or medical information technology).tw,kw.	213
118	medical informatics computing/	0
119	nursing informatics/	10
120	health information exchange/	9
121	health information exchange.tw,kw.	42
122	medical informatics applications/	23
123	medical informatics applications.tw,kw.	3
124	decision making, computer-assisted/	135
125	(decision support system* or decision support techniques).tw,kw.	1719
126	diagnosis, computer-assisted/	735
127	(automatic diagnosis or computer assisted diagnosis or computer diagnosis or automatic diagnoses or computer assisted diagnoses or computer diagnoses).tw,kw.	194
128	image interpretation, computer-assisted/	995
129	computer assisted image interpretation.tw,kw.	0
130	radiographic image interpretation, computer-assisted/	406
131	computer assisted radiographic image interpretation*.tw,kw.	0
132	therapy, computer-assisted/	1370
133	computer assisted therap*.tw,kw.	262
134	drug therapy, computer-assisted/	153
135	computer assisted drug therap*.tw,kw.	22
136	decision support systems, clinical/	452
137	clinical decision support system*.tw,kw.	410
138	information systems/	65
139	(information system or information systems).tw,kw.	1770
140	online systems/	161
141	(online system or online systems).tw,kw.	705
142	user-computer interface/	1335
143	(computer interface* or computer user interface*).tw,kw.	582
144	Social Media/	272

145	(social media or social medium or Facebook or Flickr or Instagram or LinkedIn or MySpace or Pinterest or Reddit or "Sina Weibo" or Snapchat or online social network* or social networking platform* or social networking site* or social networking website* or social platform* or TikTok or Tumblr or Twitter or "web 2.0" or "web 2.0s" or WeChat or WhatsApp or YouTube).tw,kw.	3505
146	Virtual Reality/	504
147	(virtual reality or virtual realities).tw,kw.	4657
148	Augmented Reality/	26
149	(augmented realities or augmented reality or mixed realities or mixed reality).tw,kw.	351
150	holography/	5
151	(hologram* or holograph*).tw,kw.	75
152	Printing, Three-Dimensional/	112
153	("3d printing*" or "3 d printing*" or "3 dimensional printing*" or "three dimensional printing*").tw,kw.	425
154	(chatbot or chatbots or "chat bot" or "chat bots").tw,kw.	109
155	"virtual care".tw,kw.	54
156	(closed loop medicines system* or closed loop medication system* or closed loop medicines process* or closed loop medication process*).tw,kw.	0
157	(bedside station or bedside stations or bed side station or bed side stations or bedside terminal or bedside terminals or bed side terminal or bed side terminals).tw,kw.	0
158	predictive analytic*.tw,kw.	64
159	or/27-158	65803
160	26 and 159	16488
161	(champion or champions).tw,kw.	632
162	(change agent or change agents).tw,kw.	114
163	(formal leader or formal leaders or informal leader or informal leaders or opinion leader or opinion leaders).tw,kw.	258
164	(superuser or superusers or super user or super users).tw,kw.	14
165	(boundary spanner or boundary spanners).tw,kw.	3
166	(advocate or advocates).tw,kw.	1759
167	(supporter or supporters).tw,kw.	383
168	mentors/	246
169	Mentoring/	290
170	(mentor or mentors).tw,kw.	1025
171	(coach or coaches).tw,kw.	2807
172	peer group/ or peer influence/	1559
173	("peer to peer" or peer influence).tw,kw.	354
174	((peer or peers) adj3 collaborat*).tw,kw.	39
175	interpersonal influence*.tw,kw.	12
176	(innovator or innovators).tw,kw.	447
177	(ambassador or ambassadors).tw,kw.	80

178	or/161-177	9315
179	160 and 178	527
180	limit 179 to english language	526
181	limit 180 to yr="2017 -Current"	307

Database: Cochrane Database of Systematic Reviews
Search strategy:

#	Searches	Results
1	(nurse or nurses or nursing).mp.	3065
2	(health personnel or health employee* or healthcare employee* or health care employee* or healthcare professional* or health care professional* or healthcare provider* or health care provider* or healthcare personnel or health care personnel or healthcare worker* or health care worker* or medical staff).mp.	2180
3	(doctor or doctors or hospital registrar* or physician* or allergist* or anesthesiologist* or cardiologist* or dermatologist* or endocrinologist* or gastroenterologist* or general practitioner* or geriatrician* or hospitalist* or nephrologist* or neurologist* or oncologist* or ophthalmologist* or osteopathic physician* or otolaryngologist* or pathologist* or paediatrician* or pediatrician* or neonatologist* or pharmacist or pharmacists or physiatrist* or pulmonologist* or radiologist* or rheumatologist* or surgeon* or neurosurgeon* or urologist* or psychotherapist*).mp.	5647
4	(occupational therapist* or physical therapist*).mp.	259
5	(health team or health teams or healthcare team* or health care team* or interdisciplinary care team* or medical care team or medical care teams or multidisciplinary care team* or patient care team or patient care teams).mp.	186
6	(dietician or dietitian or nutritionist or dieticians or dietitians or nutritionists).mp.	196
7	(case worker or case workers or caseworker or caseworkers or social worker or social workers).mp.	231
8	(respiratory care practitioner or respiratory care practitioners or respiratory therapist or respiratory therapists).mp.	50
9	(logopedist or logopedists or "speech and language pathologist" or "speech and language pathologist*" or speech language pathologist or speech language pathologists or speech therapist or speech therapists or speech language therapist or speech language therapists).mp.	45
10	or/1-9	7187
11	(telemedicine or telecardiology or teledermatology or telediagnosis or telediagnoses or telehealth or telemonitoring or telenephrology or teleneurology or telepsychology or teleradiology or teleradiotherap* or telesurger* or teletherap* or videoconsult*).mp.	182
12	("tele medicine" or "tele cardiology" or "tele dermatology" or "tele diagnosis" or "tele diagnoses" or "tele monitoring" or "tele nephrology" or "tele neurology" or "tele	39

	psychology" or "tele radiology" or "tele radiotherap*" or "tele surger*" or "tele therap*" or "video consult*").mp.	
13	("e-health" or ehealth or "tele health").mp.	115
14	(telenurs* or "tele nurs*").mp.	26
15	("cyber consult*" or cyberconsult* or econsult* or "e–consult*" or "email based consult*" or "internet consult*" or "internet based consult*" or "online consult*" or "tele consult*" or "telephone based consult*" or "telephone consult*" or "virtual consult*" or "web consult*" or "webbased consult*" or "web based consult*").mp.	67
16	("e–rehabilitation" or remote rehabilitation or tele rehabilitation or virtual rehabilitation).mp.	12
17	(computerised medical record system* or computerized medical record system*).mp.	3
18	(electronic health record* or computerised patient record* or computerized patient record* or electronic medical record* or electronic patient record* or ehr or emr).mp.	99
19	(smart card or smart cards).mp.	2
20	medical order entry system*.mp.	6
21	(hospital information system* or patient health record information system*).mp.	10
22	ambulatory care information system*.mp.	5
23	("e–prescribing" or "e–prescription*" or electronic prescription*).mp.	4
24	("c.p.o.e. system" or computer* order entry or computer* physician order entry or computer* provider order entry or computer* order entry or computer* prescriber order entry or computer* prescribing order entry or computer* prescription order entry or "cpoe").mp.	14
25	(operating room information system* or operating room information management system*).mp.	1
26	(bedside computing or bedside technolog* or point of care system* or point of care technolog* or point of care information system*).mp.	23
27	(management information system* or management information).mp.	89
28	clinical pharmacy information system*.mp.	3
29	(database management system* or database management tool or database management tools or database manager system* or database managing system* or data base manager system* or data base management software or data base management system* or data base management tool or data base management tools).mp.	4
30	decision support system*.mp.	72
31	(mobile app or mobile apps or mobile application* or mobile health app or mobile health apps or mobile health application* or portable software app or portable software apps or portable software application* or tablet app or tablet apps or tablet application*).mp.	64
32	(cell phone or cell phones or cellphone or cellphones or cellular phone or cellular phones or cellular telephone or cellular telephones or mobile phone or mobile phones or mobile telephone or mobile telephones).mp.	210
33	(smartphone or smartphones or smart phone or smart phones).mp.	147
34	(text messaging or texting).mp.	104
35	(video conference* or video conferencing or videoconferenc*).mp.	78
36	(webcast* or web cast*).mp.	19

37	(wireless communication* or wireless technol*).mp.	24
38	(hand held computer* or handheld computer* or palm PC or palmtop or personal data assistant* or personal digital assistant or pocket computer* or pocket sized computer*).mp.	44
39	internet.mp.	906
40	(internet based intervention* or online based intervention* or online intervention* or web intervention* or web based intervention*).mp.	92
41	smart technolog*.mp.	3
42	(wearable computer or wearable computers or wearable electronic device*).mp.	5
43	(activity tracker* or fitness tracker* or smart watch or smart watches or pedometer*).mp.	81
44	("ar glasses" or "ar head mounted device" or "ar head mounted display" or "ar head worn display" or "ar headset" or "ar hud" or "arhmd" or "hmd ar" or "optical see through head mounted display" or "ost hmd" or "see through hmd" or "google glasses" or "smartglasses" or "smart glasses").mp.	0
45	(augmented reality glasses or augmented reality head mounted device or augmented reality head mounted display or augmented reality head up display or augmented reality head worn display or head mounted display augmented reality).mp.	0
46	(artificial intelligence or machine intelligence).mp.	33
47	(expert system* or knowledge based system*).mp.	13
48	(fuzzy logic or fuzzy model or fuzzy models).mp.	7
49	(machine learning or learning machine*).mp.	115
50	(deep learning or hierarchical learning).mp.	4
51	unsupervised machine learning.mp.	1
52	natural language processing.mp.	3
53	("ann approach*" or "ann model" or "ann method*" or "ann training" or artificial neural network or computer neural network or computer neural networks).mp.	2
54	(robotics or nanorobotics or robot or robots).mp.	67
55	(biomedical technology or bio medical technology).mp.	5
56	informatics.mp.	76
57	(clinical information science or clinical information technology or health information science or health information technology or medical computer science or medical information science or medical information technology).mp.	12
58	health information exchange.mp.	1
59	(decision support system* or decision support techniques).mp.	83
60	(automatic diagnosis or computer assisted diagnosis or computer diagnosis or automatic diagnoses or computer assisted diagnoses or computer diagnoses).mp.	17
61	computer assisted image interpretation.mp.	0
62	computer assisted radiographic image interpretation*.mp.	0
63	computer assisted therap*.mp.	20
64	computer assisted drug therap*.mp.	3
65	clinical decision support system*.mp.	20

66	(information system or information systems).mp.	127
67	(online system or online systems).mp.	10
68	(computer interface* or computer user interface*).mp.	29
69	(social media or social medium or Facebook or Flickr or Instagram or LinkedIn or MySpace or Pinterest or Reddit or "Sina Weibo" or Snapchat or online social network* or social networking platform* or social networking site* or social networking website* or social platform* or TikTok or Tumblr or Twitter or "web 2.0" or "web 2.0s" or WeChat or WhatsApp or YouTube).mp.	156
70	(virtual reality or virtual realities).mp.	90
71	(augmented realities or augmented reality or mixed realities or mixed reality).mp.	6
72	(hologram* or holograph*).mp.	1
73	("3d printing*" or "3 d printing*" or "3 dimensional printing*" or "three dimensional printing*").mp.	0
74	(chatbot or chatbots or "chat bot" or "chat bots").mp.	1
75	"virtual care".mp.	0
76	(closed loop medicines system* or closed loop medication system* or closed loop medicines process* or closed loop medication process*).mp.	0
77	(bedside station or bedside stations or bed side station or bed side stations or bedside terminal or bedside terminals or bed side terminal or bed side terminals).mp.	2
78	predictive analytic*.mp.	0
79	or/11-78	1684
80	10 and 79	1392
81	(champion or champions).mp.	81
82	(change agent or change agents).mp.	9
83	(formal leader or formal leaders or informal leader or informal leaders or opinion leader or opinion leaders).mp.	55
84	(superuser or superusers or super user or super users).mp.	1
85	(boundary spanner or boundary spanners).mp.	0
86	(advocate or advocates).mp.	532
87	(supporter or supporters).mp.	69
88	(mentor or mentors).mp.	88
89	(coach or coaches).mp.	108
90	("peer to peer" or peer influence).mp.	31
91	((peer or peers) adj3 collaborat*).mp.	3
92	interpersonal influence*.mp.	1
93	(innovator or innovators).mp.	5
94	(ambassador or ambassadors).mp.	6
95	or/81-94	888
96	80 and 95	280
97	limit 96 to last 5 years	91
98	limit 97 to full systematic reviews	77

Database: APA PsychInfo
Search strategy:

#	Searches	Results
1	nurses/ or psychiatric nurses/ or public health service nurses/	34864
2	nursing/	25401
3	(nurse or nurses or nursing).tw,id.	109373
4	exp Medical Personnel/	90409
5	(health personnel or health employee* or healthcare employee* or health care employee* or healthcare professional* or health care professional* or healthcare provider* or health care provider* or healthcare personnel or health care personnel or healthcare worker* or health care worker* or medical staff).tw,id.	48394
6	(doctor or doctors or hospital registrar* or physician* or allergist* or anesthesiologist* or cardiologist* or dermatologist* or endocrinologist* or gastroenterologist* or general practitioner* or geriatrician* or hospitalist* or nephrologist* or neurologist* or oncologist* or ophthalmologist* or osteopathic physician* or otolaryngologist* or pathologist* or paediatrician* or pediatrician* or neonatologist* or pharmacist or pharmacists or physiatrist* or pulmonologist* or radiologist* or rheumatologist* or surgeon* or neurosurgeon* or urologist* or psychotherapist*).tw,id.	140554
7	(occupational therapist* or physical therapist*).tw,id.	6908
8	(health team or health teams or healthcare team* or health care team* or interdisciplinary care team* or medical care team or medical care teams or multidisciplinary care team* or patient care team or patient care teams).tw,id.	4338
9	(dietician or dietitian or nutritionist or dieticians or dietitians or nutritionists).tw,id.	1594
10	social workers/	13778
11	(case worker or case workers or caseworker or caseworkers or social worker or social workers).tw,id.	28975
12	(respiratory care practitioner or respiratory care practitioners or respiratory therapist or respiratory therapists).tw,id.	111
13	speech therapists/	1470
14	(logopedist or logopedists or "speech and language pathologist" or "speech and language pathologist*" or speech language pathologist or speech language pathologists or speech therapist or speech therapists or speech language therapist or speech language therapists).tw,id.	4327
15	or/1-14	319546
16	telemedicine/ or online therapy/ or teleconsultation/ or telepsychiatry/ or telepsychology/ or telerehabilitation/	10449
17	teleconferencing/	979
18	(telemedicine or telecardiology or teledermatology or telediagnosis or telediagnoses or telehealth or telemonitoring or telenephrology or teleneurology or telepsychology or teleradiology or teleradiotherap* or telesurger* or teletherap* or videoconsult*).tw,id.	5804

19	("tele medicine" or "tele cardiology" or "tele dermatology" or "tele diagnosis" or "tele diagnoses" or "tele monitoring" or "tele nephrology" or "tele neurology" or "tele psychology" or "tele radiology" or "tele radiotherap*" or "tele surger*" or "tele therap*" or "video consult*").tw,id.	178
20	("e-health" or ehealth or "tele health").tw,id.	2701
21	(telenurs* or "tele nurs*").tw,id.	96
22	online therapy/	3702
23	("cyber consult*" or cyberconsult* or econsult* or "e−consult*" or "email based consult*" or "internet consult*" or "internet based consult*" or "online consult*" or "tele consult*" or "telephone based consult*" or "telephone consult*" or "virtual consult*" or "web consult*" or "webbased consult*" or "web based consult*").tw,id.	407
24	("e−rehabilitation" or remote rehabilitation or tele rehabilitation or virtual rehabilitation).tw,id.	111
25	(computerised medical record system* or computerized medical record system*).tw,id.	2
26	electronic health records/	1098
27	(electronic health record* or computerised patient record* or computerized patient record* or electronic medical record* or electronic patient record* or ehr or emr).tw,id.	5666
28	(smart card or smart cards).tw,id.	55
29	medical order entry system*.tw,id.	2
30	(hospital information system* or patient health record information system*).tw,id.	104
31	ambulatory care information system*.tw,id.	0
32	("e−prescribing" or "e−prescription*" or electronic prescription*).tw,id.	63
33	("c.p.o.e. system" or computer* order entry or computer* physician order entry or computer* provider order entry or computer* order entry or computer* prescriber order entry or computer* prescribing order entry or computer* prescription order entry or "cpoe").tw,id.	90
34	(operating room information system* or operating room information management system*).tw,id.	0
35	(bedside computing or bedside technolog* or point of care system* or point of care technolog* or point of care information system*).tw,id.	36
36	bedside information system*.tw,id.	0
37	information systems/	5882
38	(management information system* or management information).tw,id.	822
39	clinical pharmacy information system*.tw,id.	2
40	(database management system* or database management tool or database management tools or database manager system* or database managing system* or data base manager system* or data base management software or data base management system* or data base management tool or data base management tools).tw,id.	95
41	decision support systems/	3594
42	decision support system*.tw,id.	2169
43	mobile applications/	1887

44	(mobile app or mobile apps or mobile application* or mobile health app or mobile health apps or mobile health application* or portable software app or portable software apps or portable software application* or tablet app or tablet apps or tablet application*).tw,id.	2702
45	mobile devices/ or tablet computers/ or mobile phones/	8660
46	(cell phone or cell phones or cellphone or cellphones or cellular phone or cellular phones or cellular telephone or cellular telephones or mobile phone or mobile phones or mobile telephone or mobile telephones).tw,id.	6968
47	smartphones/	2651
48	"smartphone use"/	566
49	(smartphone or smartphones or smart phone or smart phones).tw,id.	6731
50	(text messaging or texting).tw,id.	2399
51	videoconferencing/ or video-based interventions/	938
52	(video conference* or video conferencing or videoconferenc*).tw,id.	2578
53	(webcast* or web cast*).tw,id.	81
54	wireless technologies/	590
55	(wireless communication* or wireless technol*).tw,id.	346
56	(hand held computer* or handheld computer* or palm PC or palmtop or personal data assistant* or personal digital assistant or pocket computer* or pocket sized computer*).tw,id.	776
57	internet/	30496
58	internet.tw,id.	46698
59	(internet based intervention* or online based intervention* or online intervention* or web intervention* or web based intervention*).tw,id.	2399
60	smart technolog*.tw,id.	159
61	wearable devices/	559
62	(wearable computer or wearable computers or wearable electronic device*).tw,id.	86
63	(activity tracker* or fitness tracker* or smart watch or smart watches or pedometer*).tw,id.	1200
64	("ar glasses" or "ar head mounted device" or "ar head mounted display" or "ar head worn display" or "ar headset" or "ar hud" or "arhmd" or "hmd ar" or "optical see through head mounted display" or "ost hmd" or "see through hmd" or "google glasses" or "smartglasses" or "smart glasses").tw,id.	57
65	(augmented reality glasses or augmented reality head mounted device or augmented reality head mounted display or augmented reality head up display or augmented reality head worn display or head mounted display augmented reality).tw,id.	11
66	artificial intelligence/	9935
67	(artificial intelligence or machine intelligence).tw,id.	7110
68	computer heuristic*.tw,id.	3
69	expert systems/	5925
70	(expert system* or knowledge based system*).tw,id.	2372
71	fuzzy logic/	1725

72	(fuzzy logic or fuzzy model or fuzzy models).tw,id.	944
73	machine learning/	10687
74	(machine learning or learning machine*).tw,id.	10663
75	(deep learning or hierarchical learning).tw,id.	2813
76	unsupervised machine learning.tw,id.	122
77	natural language processing/	814
78	natural language processing.tw,id.	1597
79	artificial neural networks/ or deep neural networks/	5086
80	("ann approach*" or "ann model" or "ann method*" or "ann training" or artificial neural network or computer neural network or computer neural networks).tw,id.	1128
81	robotics/ or avatars/ or social robotics/	7731
82	(robotics or nanorobotics or robot or robots).tw,id.	8741
83	(biomedical technology or bio medical technology).tw,id.	71
84	informatics.tw,id.	1752
85	(clinical information science or clinical information technology or health information science or health information technology or medical computer science or medical information science or medical information technology).tw,id.	766
86	health information exchange.tw,id.	130
87	(decision support system* or decision support techniques).tw,id.	2216
88	computer assisted diagnosis/	1629
89	(automatic diagnosis or computer assisted diagnosis or computer diagnosis or automatic diagnoses or computer assisted diagnoses or computer diagnoses).tw,id.	167
90	computer assisted image interpretation.tw,id.	0
91	computer assisted radiographic image interpretation*.tw,id.	0
92	computer assisted therapy/	1190
93	computer assisted therap*.tw,id.	111
94	computer assisted drug therap*.tw,id.	0
95	clinical decision support system*.tw,id.	260
96	information systems/	5882
97	(information system or information systems).tw,id.	8866
98	(online system or online systems).tw,id.	227
99	(computer interface* or computer user interface*).tw,id.	2728
100	social media/ or online social networks/	19799
101	(social media or social medium or Facebook or Flickr or Instagram or LinkedIn or MySpace or Pinterest or Reddit or "Sina Weibo" or Snapchat or online social network* or social networking platform* or social networking site* or social networking website* or social platform* or TikTok or Tumblr or Twitter or "web 2.0" or "web 2.0s" or WeChat or WhatsApp or YouTube).tw,id.	28069
102	virtual reality/ or augmented reality/	10684
103	(virtual reality or virtual realities).tw,id.	8057
104	(augmented realities or augmented reality or mixed realities or mixed reality).tw,id.	1428

105	(hologram* or holograph*).tw,id.	443
106	("3d printing*" or "3 d printing*" or "3 dimensional printing*" or "three dimensional printing*").tw,id.	151
107	(chatbot or chatbots or "chat bot" or "chat bots").tw,id.	274
108	"virtual care".tw,id.	88
109	(closed loop medicines system* or closed loop medication system* or closed loop medicines process* or closed loop medication process*).tw,id.	0
110	(bedside station or bedside stations or bed side station or bed side stations or bedside terminal or bedside terminals or bed side terminal or bed side terminals).tw,id.	1
111	predictive analytic?.tw,id.	206
112	or/16-111	188321
113	15 and 112	14167
114	(champion or champions).tw,id.	2506
115	(change agent or change agents).tw,id.	2379
116	(formal leader or formal leaders or informal leader or informal leaders or opinion leader or opinion leaders).tw,id.	974
117	(superuser or superusers or super user or super users).tw,id.	28
118	(boundary spanner or boundary spanners).tw,id.	267
119	(advocate or advocates).tw,id.	23656
120	(supporter or supporters).tw,id.	3739
121	mentor/	7659
122	(mentor or mentors).tw,id.	11149
123	(coach or coaches).tw,id.	12679
124	peers/	15693
125	("peer to peer" or peer influence).tw,id.	4102
126	((peer or peers) adj3 collaborat*).tw,id.	998
127	Interpersonal Influences/	6174
128	interpersonal influence*.tw,id.	1080
129	(innovator or innovators).tw,id.	1439
130	(ambassador or ambassadors).tw,id.	480
131	or/114-130	84903
132	113 and 131	422
133	limit 132 to english language	408
134	limit 133 to yr="2017 -Current"	184
135	limit 134 to (bibliography or chapter or "column/opinion" or "comment/reply" or dissertation or editorial or encyclopedia entry or interview or letter or review-book or review-media or review-software & other)	58
136	134 not 135	126

Database: Cumulative Index to Nursing and Allied Health (CINAHL)
Search strategy:

#	Query	Limiters/Expanders	Results
S1	(MH "Nursing Manpower+")	Expanders - Apply equivalent subjects<br>Search modes - Boolean/Phrase	268,072
S2	(MH "Nursing Role")	Expanders - Apply equivalent subjects<br>Search modes - Boolean/Phrase	63,415
S3	(MH "Telenursing")	Expanders - Apply equivalent subjects<br>Search modes - Boolean/Phrase	2,288
S4	(MH "Nursing Process")	Expanders - Apply equivalent subjects<br>Search modes - Boolean/Phrase	3,693
S5	(MH "Nursing Assessment")	Expanders - Apply equivalent subjects<br>Search modes - Boolean/Phrase	18,503
S6	(MH "Nursing Diagnosis")	Expanders - Apply equivalent subjects<br>Search modes - Boolean/Phrase	4,765
S7	(MH "Nursing Service")	Expanders - Apply equivalent subjects<br>Search modes - Boolean/Phrase	1,530
S8	(MH "Team Nursing") OR (MH "Nursing Care Delivery Systems") OR (MH "Differentiated Nursing Practice") OR (MH "Functional Nursing") OR (MH "Modular Nursing") OR (MH "Primary Nursing") OR (MH "Total Patient Care Nursing") OR (MH "Nursing Care")	Expanders - Apply equivalent subjects<br>Search modes - Boolean/Phrase	29,873
S9	TI (nurse or nurses or nursing) OR AB (nurse or nurses or nursing)	Expanders - Apply equivalent subjects<br>Search modes - Boolean/Phrase	603,859
S10	(MH "Health Personnel+")	Expanders - Apply equivalent subjects<br>Search modes - Boolean/Phrase	634,387
S11	TI (health personnel or health employee* or healthcare employee* or health care employee* or healthcare professional* or health care professional* or healthcare provider* or health	Expanders - Apply equivalent subjects<br>Search modes - Boolean/Phrase	108,877

	care provider* or healthcare personnel or health care personnel or healthcare worker* or health care worker* or medical staff) OR AB (health personnel or health employee* or healthcare employee* or health care employee* or healthcare professional* or health care professional* or healthcare provider* or health care provider* or healthcare personnel or health care personnel or healthcare worker* or health care worker* or medical staff)		
S12	TI (doctor or doctors or hospital registrar* or physician* or allergist* or anesthesiologist* or cardiologist* or dermatologist* or endocrinologist* or gastroenterologist* or general practitioner* or geriatrician* or hospitalist* or nephrologist* or neurologist* or oncologist* or ophthalmologist* or osteopathic physician* or otolaryngologist* or pathologist* or paediatrician* or pediatrician* or neonatologist* or pharmacist or pharmacists or physiatrist* or pulmonologist* or radiologist* or rheumatologist* or surgeon* or neurosurgeon* or urologist* or psychotherapist*)	Expanders - Apply equivalent subjects<br>Search modes - Boolean/Phrase	111,479
S13	TI (occupational therapist* or physical therapist*) OR AB (occupational therapist* or physical therapist* or doctor or doctors or hospital registrar* or physician* or allergist* or anesthesiologist* or cardiologist* or dermatologist* or endocrinologist* or gastroenterologist* or general practitioner* or geriatrician* or hospitalist* or nephrologist* or neurologist* or oncologist* or ophthalmologist* or osteopathic physician* or otolaryngologist* or pathologist* or paediatrician* or pediatrician* or neonatologist* or pharmacist or pharmacists or physiatrist* or pulmonologist* or radiologist* or rheumatologist* or surgeon* or neurosurgeon* or urologist* or psychotherapist*)	Expanders - Apply equivalent subjects<br>Search modes - Boolean/Phrase	339,581
S14	(MH "Multidisciplinary Care Team")	Expanders - Apply equivalent subjects<br>Search modes - Boolean/Phrase	49,437
S15	TI (health team or health teams or healthcare team* or health care team* or interdisciplinary care team* or medical care team or medical care	Expanders - Apply equivalent subjects<br>Search modes - Boolean/Phrase	10,321

	teams or multidisciplinary care team* or patient care team or patient care teams) OR AB (health team or health teams or healthcare team* or health care team* or interdisciplinary care team* or medical care team or medical care teams or multidisciplinary care team* or patient care team or patient care teams)		
S16	(MH "Dietitians")	Expanders - Apply equivalent subjects<br>Search modes - Boolean/Phrase	5,785
S17	TI (dietician or dietitian or nutritionist or dieticians or dietitians or nutritionists) OR AB (dietician or dietitian or nutritionist or dieticians or dietitians or nutritionists)	Expanders - Apply equivalent subjects<br>Search modes - Boolean/Phrase	8,065
S18	(MH "Social Workers")	Expanders - Apply equivalent subjects<br>Search modes - Boolean/Phrase	10,496
S19	TI (case worker or case workers or caseworker or caseworkers or social worker or social workers) OR AB (case worker or case workers or caseworker or caseworkers or social worker or social workers)	Expanders - Apply equivalent subjects<br>Search modes - Boolean/Phrase	13,364
S20	(MH "Respiratory Therapists")	Expanders - Apply equivalent subjects<br>Search modes - Boolean/Phrase	3,786
S21	TI (respiratory care practitioner or respiratory care practitioners or respiratory therapist or respiratory therapists) OR AB (respiratory care practitioner or respiratory care practitioners or respiratory therapist or respiratory therapists)	Expanders - Apply equivalent subjects<br>Search modes - Boolean/Phrase	2,388
S22	(MH "Speech-Language Pathologists")	Expanders - Apply equivalent subjects<br>Search modes - Boolean/Phrase	8,210
S23	TI (logopedist or logopedists or "speech and language pathologist" or "speech and language pathologist*" or speech language pathologist or speech language pathologists or speech therapist or speech therapists or speech language therapist or speech language therapists) OR AB (logopedist or logopedists or "speech and language pathologist" or "speech and language pathologist*" or speech language pathologist or	Expanders - Apply equivalent subjects<br>Search modes - Boolean/Phrase	5,587

	speech language pathologists or speech therapist or speech therapists or speech language therapist or speech language therapists)		
S24	S1 OR S2 OR S3 OR S4 OR S5 OR S6 OR S7 OR S8 OR S9 OR S10 OR S11 OR S12 OR S13 OR S14 OR S15 OR S16 OR S17 OR S18 OR S19 OR S20 OR S21 OR S22 OR S23	Expanders - Apply equivalent subjects<br>Search modes - Boolean/Phrase	1,466,404
S25	(MH "Telemedicine") OR (MH "Telepathology") OR (MH "Teleradiology") OR (MH "Telenutrition")	Expanders - Apply equivalent subjects<br>Search modes - Boolean/Phrase	15,985
S26	TI (telemedicine or telecardiology or teledermatology or telediagnosis or telediagnoses or telemonitoring or telenephrology or teleneurology or telepsychology or teleradiology or teleradiotherap* or telesurger* or teletherap* or videoconsult*) OR AB (telemedicine or telecardiology or teledermatology or telediagnosis or telediagnoses or telemonitoring or telenephrology or teleneurology or telepsychology or teleradiology or teleradiotherap* or telesurger* or teletherap* or videoconsult*)	Expanders - Apply equivalent subjects<br>Search modes - Boolean/Phrase	9,465
S27	TI ("tele medicine" or "tele cardiology" or "tele dermatology" or "tele diagnosis" or "tele diagnoses" or "tele monitoring" or "tele nephrology" or "tele neurology" or "tele psychology" or "tele radiology" or "tele radiotherap*" or "tele surger*" or "tele therap*" or "video consult*") OR AB ("tele medicine" or "tele cardiology" or "tele dermatology" or "tele diagnosis" or "tele diagnoses" or "tele monitoring" or "tele nephrology" or "tele neurology" or "tele psychology" or "tele radiology" or "tele radiotherap*" or "tele surger*" or "tele therap*" or "video consult*")	Expanders - Apply equivalent subjects<br>Search modes - Boolean/Phrase	451
S28	(MH "Telehealth")	Expanders - Apply equivalent subjects<br>Search modes - Boolean/Phrase	12,466
S29	TI ("e-health" or ehealth or "tele health") OR AB ("e-health" or ehealth or "tele health")	Expanders - Apply equivalent subjects<br>Search modes - Boolean/Phrase	4,444

S30	(MH "Telenursing")	Expanders - Apply equivalent subjects<br>Search modes - Boolean/Phrase	2,288
S31	TI (telenurs* or "tele nurs*") OR AB (telenurs* or "tele nurs*")	Expanders - Apply equivalent subjects<br>Search modes - Boolean/Phrase	265
S32	(MH "Remote Consultation")	Expanders - Apply equivalent subjects<br>Search modes - Boolean/Phrase	2,917
S33	TI ("cyber consult*" or cyberconsult* or econsult* or "e−consult*" or "email based consult*" or "internet consult*" or "internet based consult*" or "online consult*" or "tele consult*" or "telephone based consult*" or "telephone consult*" or "virtual consult*" or "web consult*" or "webbased consult*" or "web based consult*") OR AB ("cyber consult*" or cyberconsult* or econsult* or "e−consult*" or "email based consult*" or "internet consult*" or "internet based consult*" or "online consult*" or "tele consult*" or "telephone based consult*" or "telephone consult*" or "virtual consult*" or "web consult*" or "webbased consult*" or "web based consult*")	Expanders - Apply equivalent subjects<br>Search modes - Boolean/Phrase	1,296
S34	(MH "Telerehabilitation")	Expanders - Apply equivalent subjects<br>Search modes - Boolean/Phrase	489
S35	TI ("e−rehabilitation" or remote rehabilitation or tele rehabilitation or virtual rehabilitation) OR AB ("e−rehabilitation" or remote rehabilitation or tele rehabilitation or virtual rehabilitation)	Expanders - Apply equivalent subjects<br>Search modes - Boolean/Phrase	256
S36	TI (computerised medical record system* or computerized medical record system*) OR AB (computerised medical record system* or computerized medical record system*)	Expanders - Apply equivalent subjects<br>Search modes - Boolean/Phrase	34
S37	(MH "Electronic Health Records")	Expanders - Apply equivalent subjects<br>Search modes - Boolean/Phrase	28,783
S38	TI (electronic health record* or computerised patient record* or computerized patient record* or electronic medical record* or electronic patient record* or ehr or emr) OR AB (electronic	Expanders - Apply equivalent subjects<br>Search modes - Boolean/Phrase	32,077

	health record* or computerised patient record* or computerized patient record* or electronic medical record* or electronic patient record* or ehr or emr)		
S39	(MH "Smart Cards")	Expanders - Apply equivalent subjects<br>Search modes - Boolean/Phrase	173
S40	TI (smart card or smart cards) OR AB (smart card or smart cards)	Expanders - Apply equivalent subjects<br>Search modes - Boolean/Phrase	135
S41	TI (medical order entry system*) OR AB (medical order entry system*)	Expanders - Apply equivalent subjects<br>Search modes - Boolean/Phrase	2
S42	(MH "Hospital Information Systems")	Expanders - Apply equivalent subjects<br>Search modes - Boolean/Phrase	3,428
S43	TI (hospital information system* or patient health record information system*) OR AB (hospital information system* or patient health record information system*)	Expanders - Apply equivalent subjects<br>Search modes - Boolean/Phrase	954
S44	(MH "Ambulatory Care Information Systems")	Expanders - Apply equivalent subjects<br>Search modes - Boolean/Phrase	317
S45	TI (ambulatory care information system*) OR AB (ambulatory care information system*)	Expanders - Apply equivalent subjects<br>Search modes - Boolean/Phrase	4
S46	TI ("e−prescribing" or "e−prescription*" or electronic prescription*) OR AB ("e−prescribing" or "e−prescription*" or electronic prescription*)	Expanders - Apply equivalent subjects<br>Search modes - Boolean/Phrase	794
S47	(MH "Electronic Order Entry")	Expanders - Apply equivalent subjects<br>Search modes - Boolean/Phrase	3,483
S48	TI ("c.p.o.e. system" or "computer* order entry" or "computer* physician order entry" or "computer* provider order entry" or "computer* order entry" or "computer* prescriber order entry" or "computer* prescribing order entry" or "computer* prescription order entry" or "cpoe") OR AB ("c.p.o.e. system" or "computer* order entry" or "computer* physician order entry" or "computer* provider order entry" or "computer* order entry" or "computer* prescriber order	Expanders - Apply equivalent subjects<br>Search modes - Boolean/Phrase	1,372

	entry" or "computer* prescribing order entry" or "computer* prescription order entry" or "cpoe")		
S49	(MH "Operating Room Information Systems")	Expanders - Apply equivalent subjects<br>Search modes - Boolean/Phrase	417
S50	TI (operating room information system* or operating room information management system*) OR AB (operating room information system* or operating room information management system*)	Expanders - Apply equivalent subjects<br>Search modes - Boolean/Phrase	8
S51	(MH "Clinical Information Systems")	Expanders - Apply equivalent subjects<br>Search modes - Boolean/Phrase	7,408
S52	TI (bedside computing or bedside technolog* or point of care system* or point of care technolog* or point of care information system*) OR AB (bedside computing or bedside technolog* or point of care system* or point of care technolog* or point of care information system*)	Expanders - Apply equivalent subjects<br>Search modes - Boolean/Phrase	225
S53	TI (bedside information system*) OR AB (bedside information system*)	Expanders - Apply equivalent subjects<br>Search modes - Boolean/Phrase	2
S54	(MH "Management Information Systems")	Expanders - Apply equivalent subjects<br>Search modes - Boolean/Phrase	1,819
S55	TI (management information system* or management information) OR AB (management information system* or management information)	Expanders - Apply equivalent subjects<br>Search modes - Boolean/Phrase	944
S56	(MH "Clinical Pharmacy Information Systems")	Expanders - Apply equivalent subjects<br>Search modes - Boolean/Phrase	1,182
S57	TI (clinical pharmacy information system*) OR AB (clinical pharmacy information system*)	Expanders - Apply equivalent subjects<br>Search modes - Boolean/Phrase	1
S58	TI (database management system* or database management tool or database management tools or database manager system* or database managing system* or data base manager system* or data base management software or data base management system* or data base management tool or data base management	Expanders - Apply equivalent subjects<br>Search modes - Boolean/Phrase	90

	tools) OR AB (database management system* or database management tool or database management tools or database manager system* or database managing system* or data base manager system* or data base management software or data base management system* or data base management tool or data base management tools)		
S59	(MH "Decision Support Systems, Management")	Expanders - Apply equivalent subjects<br>Search modes - Boolean/Phrase	497
S60	TI (decision support system*) OR AB (decision support system*)	Expanders - Apply equivalent subjects<br>Search modes - Boolean/Phrase	2,712
S61	(MH "Mobile Applications")	Expanders - Apply equivalent subjects<br>Search modes - Boolean/Phrase	11,063
S62	TI (mobile app or mobile apps or mobile application* or mobile health app or mobile health apps or mobile health application* or portable software app or portable software apps or portable software application* or tablet app or tablet apps or tablet application*) OR AB (mobile app or mobile apps or mobile application* or mobile health app or mobile health apps or mobile health application* or portable software app or portable software apps or portable software application* or tablet app or tablet apps or tablet application*)	Expanders - Apply equivalent subjects<br>Search modes - Boolean/Phrase	4,520
S63	(MH "Cellular Phone")	Expanders - Apply equivalent subjects<br>Search modes - Boolean/Phrase	2,173
S64	TI (cell phone or cell phones or cellphone or cellphones or cellular phone or cellular phones or cellular telephone or cellular telephones or mobile phone or mobile phones or mobile telephone or mobile telephones) OR AB (cell phone or cell phones or cellphone or cellphones or cellular phone or cellular phones or cellular telephone or cellular telephones or mobile phone or mobile phones or mobile telephone or mobile telephones)	Expanders - Apply equivalent subjects<br>Search modes - Boolean/Phrase	6,480

S65	(MH "Smartphone")	Expanders - Apply equivalent subjects<br>Search modes - Boolean/Phrase	3,769
S66	TI (smartphone or smartphones or smart phones or smart phones) OR AB (smartphone or smartphones or smart phones or smart phones)	Expanders - Apply equivalent subjects<br>Search modes - Boolean/Phrase	8,733
S67	(MH "Text Messaging")	Expanders - Apply equivalent subjects<br>Search modes - Boolean/Phrase	3,881
S68	TI (text messaging or texting) OR AB (text messaging or texting)	Expanders - Apply equivalent subjects<br>Search modes - Boolean/Phrase	2,432
S69	(MH "Videoconferencing")	Expanders - Apply equivalent subjects<br>Search modes - Boolean/Phrase	2,682
S70	TI (video conference* or video conferencing or videoconferenc*) OR AB (video conference* or video conferencing or videoconferenc*)	Expanders - Apply equivalent subjects<br>Search modes - Boolean/Phrase	2,436
S71	(MH "Webcasts")	Expanders - Apply equivalent subjects<br>Search modes - Boolean/Phrase	802
S72	TI (webcast* or web cast*) OR AB (webcast* or web cast*)	Expanders - Apply equivalent subjects<br>Search modes - Boolean/Phrase	264
S73	TI (wireless communication* or wireless technol*) OR AB (wireless communication* or wireless technol*)	Expanders - Apply equivalent subjects<br>Search modes - Boolean/Phrase	497
S74	(MH "Computers, Hand-Held")	Expanders - Apply equivalent subjects<br>Search modes - Boolean/Phrase	4,665
S75	TI (hand held computer* or handheld computer* or palm PC or palmtop or personal data assistant* or personal digital assistant or pocket computer* or pocket sized computer*) OR AB (hand held computer* or handheld computer* or palm PC or palmtop or personal data assistant* or personal digital assistant or pocket computer* or pocket sized computer*)	Expanders - Apply equivalent subjects<br>Search modes - Boolean/Phrase	1,054
S76	(MH "Internet")	Expanders - Apply equivalent subjects<br>Search modes - Boolean/Phrase	53,895

S77	TI internet OR AB internet	Expanders - Apply equivalent subjects<br>Search modes - Boolean/Phrase	34,915
S78	(MH "Internet-Based Intervention")	Expanders - Apply equivalent subjects<br>Search modes - Boolean/Phrase	481
S79	TI (internet based intervention* or online based intervention* or online intervention* or web intervention* or web based intervention*) OR AB (internet based intervention* or online based intervention* or online intervention* or web intervention* or web based intervention*)	Expanders - Apply equivalent subjects<br>Search modes - Boolean/Phrase	1,838
S80	TI (smart technolog*) OR AB (smart technolog*)	Expanders - Apply equivalent subjects<br>Search modes - Boolean/Phrase	160
S81	TI (wearable computer or wearable computers or wearable electronic device*) OR AB (wearable computer or wearable computers or wearable electronic device*)	Expanders - Apply equivalent subjects<br>Search modes - Boolean/Phrase	37
S82	(MH "Fitness Trackers")	Expanders - Apply equivalent subjects<br>Search modes - Boolean/Phrase	304
S83	TI (activity tracker* or fitness tracker* or smart watch or smart watches or pedometer*) OR AB (activity tracker* or fitness tracker* or smart watch or smart watches or pedometer*)	Expanders - Apply equivalent subjects<br>Search modes - Boolean/Phrase	2,417
S84	(MH "Smart Glasses")	Expanders - Apply equivalent subjects<br>Search modes - Boolean/Phrase	25
S85	TI ("ar glasses" or "ar head mounted device" or "ar head mounted display" or "ar head worn display" or "ar headset" or "ar hud" or "arhmd" or "hmd ar" or "optical see through head mounted display" or "ost hmd" or "see through hmd" or "google glasses" or "smartglasses" or "smart glasses") OR AB ("ar glasses" or "ar head mounted device" or "ar head mounted display" or "ar head worn display" or "ar headset" or "ar hud" or "arhmd" or "hmd ar" or "optical see through head mounted display" or "ost hmd" or "see through hmd" or "google glasses" or "smartglasses" or "smart glasses")	Expanders - Apply equivalent subjects<br>Search modes - Boolean/Phrase	88

S86	(MH "Artificial Intelligence")	Expanders - Apply equivalent subjects<br>Search modes - Boolean/Phrase	7,489
S87	TI (artificial intelligence or machine intelligence) OR AB (artificial intelligence or machine intelligence)	Expanders - Apply equivalent subjects<br>Search modes - Boolean/Phrase	6,172
S88	TI (computer heuristic*) OR AB (computer heuristic*)	Expanders - Apply equivalent subjects<br>Search modes - Boolean/Phrase	0
S89	(MH "Expert Systems")	Expanders - Apply equivalent subjects<br>Search modes - Boolean/Phrase	538
S90	TI (expert system* or knowledge based system*) OR AB (expert system* or knowledge based system*)	Expanders - Apply equivalent subjects<br>Search modes - Boolean/Phrase	568
S91	TI (fuzzy logic or fuzzy model or fuzzy models) OR AB (fuzzy logic or fuzzy model or fuzzy models)	Expanders - Apply equivalent subjects<br>Search modes - Boolean/Phrase	313
S92	(MH "Machine Learning")	Expanders - Apply equivalent subjects<br>Search modes - Boolean/Phrase	3,496
S93	TI (machine learning or learning machine*) OR AB (machine learning or learning machine*)	Expanders - Apply equivalent subjects<br>Search modes - Boolean/Phrase	10,404
S94	(MH "Deep Learning")	Expanders - Apply equivalent subjects<br>Search modes - Boolean/Phrase	1,151
S95	TI (deep learning or hierarchical learning) OR AB (deep learning or hierarchical learning)	Expanders - Apply equivalent subjects<br>Search modes - Boolean/Phrase	3,875
S96	TI (unsupervised machine learning) OR AB (unsupervised machine learning)	Expanders - Apply equivalent subjects<br>Search modes - Boolean/Phrase	152
S97	(MH "Natural Language Processing")	Expanders - Apply equivalent subjects<br>Search modes - Boolean/Phrase	2,664
S98	TI (natural language processing) OR AB (natural language processing)	Expanders - Apply equivalent subjects<br>Search modes - Boolean/Phrase	1,817

S99	(MH "Neural Networks (Computer)")	Expanders - Apply equivalent subjects<br>Search modes - Boolean/Phrase	3,240
S100	TI ("ann approach*" or "ann model" or "ann method*" or "ann training" or artificial neural network or computer neural network or computer neural networks) OR AB ("ann approach*" or "ann model" or "ann method*" or "ann training" or artificial neural network or computer neural network or computer neural networks)	Expanders - Apply equivalent subjects<br>Search modes - Boolean/Phrase	1,436
S101	(MH "Robotics")	Expanders - Apply equivalent subjects<br>Search modes - Boolean/Phrase	8,818
S102	TI (robotics or nanorobotics or robot or robots) OR AB (robotics or nanorobotics or robot or robots)	Expanders - Apply equivalent subjects<br>Search modes - Boolean/Phrase	14,878
S103	TI (biomedical technology or bio medical technology) OR AB (biomedical technology or bio medical technology)	Expanders - Apply equivalent subjects<br>Search modes - Boolean/Phrase	125
S104	(MH "Informatics")	Expanders - Apply equivalent subjects<br>Search modes - Boolean/Phrase	1,385
S105	TI informatics OR AB informatics	Expanders - Apply equivalent subjects<br>Search modes - Boolean/Phrase	12,303
S106	(MH "Medical Informatics") OR (MH "Nursing Informatics") OR (MH "Health Informatics")	Expanders - Apply equivalent subjects<br>Search modes - Boolean/Phrase	12,899
S107	TI (clinical informatics or clinical information science or clinical information technology or health informatics or health information science or health information technology or medical computer science or medical information science or medical informatics or medical information technology) OR AB (clinical informatics or clinical information science or clinical information technology or health informatics or health information science or health information technology or medical computer science or medical information science or medical informatics or medical information technology)	Expanders - Apply equivalent subjects<br>Search modes - Boolean/Phrase	8,980

S108	(MH "Electronic Data Interchange")	Expanders - Apply equivalent subjects<br>Search modes - Boolean/Phrase	3,949
S109	TI (health information exchange) OR AB (health information exchange)	Expanders - Apply equivalent subjects<br>Search modes - Boolean/Phrase	1,042
S110	(MH "Decision Making, Computer Assisted")	Expanders - Apply equivalent subjects<br>Search modes - Boolean/Phrase	1,383
S111	TI (decision support system* or decision support techniques) OR AB (decision support system* or decision support techniques)	Expanders - Apply equivalent subjects<br>Search modes - Boolean/Phrase	2,716
S112	(MH "Diagnosis, Computer Assisted")	Expanders - Apply equivalent subjects<br>Search modes - Boolean/Phrase	4,812
S113	TI (automatic diagnosis or computer assisted diagnosis or computer diagnosis or automatic diagnoses or computer assisted diagnoses or computer diagnoses) OR AB (automatic diagnosis or computer assisted diagnosis or computer diagnosis or automatic diagnoses or computer assisted diagnoses or computer diagnoses)	Expanders - Apply equivalent subjects<br>Search modes - Boolean/Phrase	174
S114	(MH "Image Interpretation, Computer Assisted")	Expanders - Apply equivalent subjects<br>Search modes - Boolean/Phrase	9,493
S115	TI (computer assisted image interpretation) OR AB (computer assisted image interpretation)	Expanders - Apply equivalent subjects<br>Search modes - Boolean/Phrase	1
S116	(MH "Radiographic Image Interpretation, Computer-Assisted")	Expanders - Apply equivalent subjects<br>Search modes - Boolean/Phrase	4,549
S117	TI (computer assisted radiographic image interpretation*) OR AB (computer assisted radiographic image interpretation*)	Expanders - Apply equivalent subjects<br>Search modes - Boolean/Phrase	0
S118	(MH "Therapy, Computer Assisted") OR (MH "Drug Therapy, Computer Assisted")	Expanders - Apply equivalent subjects<br>Search modes - Boolean/Phrase	5,985
S119	TI (computer assisted therap*) OR AB (computer assisted therap*)	Expanders - Apply equivalent subjects<br>Search modes - Boolean/Phrase	37

S120	TI (computer assisted drug therap*) OR AB (computer assisted drug therap*)	Expanders - Apply equivalent subjects<br>Search modes - Boolean/Phrase	4
S121	(MH "Decision Support Systems, Clinical")	Expanders - Apply equivalent subjects<br>Search modes - Boolean/Phrase	6,280
S122	TI (clinical decision support system*) OR AB (clinical decision support system*)	Expanders - Apply equivalent subjects<br>Search modes - Boolean/Phrase	1,304
S123	(MH "Information Systems")	Expanders - Apply equivalent subjects<br>Search modes - Boolean/Phrase	4,920
S124	TI (information system or information systems) OR AB (information system or information systems)	Expanders - Apply equivalent subjects<br>Search modes - Boolean/Phrase	14,347
S125	(MH "Online Systems")	Expanders - Apply equivalent subjects<br>Search modes - Boolean/Phrase	1,880
S126	TI (online system or online systems) OR AB (online system or online systems)	Expanders - Apply equivalent subjects<br>Search modes - Boolean/Phrase	260
S127	(MH "User-Computer Interface")	Expanders - Apply equivalent subjects<br>Search modes - Boolean/Phrase	11,214
S128	TI (computer interface* or computer user interface*) OR AB (computer interface* or computer user interface*)	Expanders - Apply equivalent subjects<br>Search modes - Boolean/Phrase	774
S129	(MH "Social Media") OR (MH "Facebook") OR (MH "Twitter")	Expanders - Apply equivalent subjects<br>Search modes - Boolean/Phrase	20,464
S130	(MH "Online Social Networking")	Expanders - Apply equivalent subjects<br>Search modes - Boolean/Phrase	550
S131	TI (social media or social medium or Facebook or Flickr or Instagram or LinkedIn or MySpace or Pinterest or Reddit or "Sina Weibo" or Snapchat or online social network* or social networking platform* or social networking site* or social networking website* or social platform* or TikTok or Tumblr or Twitter or "web 2.0" or "web 2.0s" or WeChat or WhatsApp or YouTube)	Expanders - Apply equivalent subjects<br>Search modes - Boolean/Phrase	13,252

S132	AB (social media or social medium or Facebook or Flickr or Instagram or LinkedIn or MySpace or Pinterest or Reddit or "Sina Weibo" or Snapchat or online social network* or social networking platform* or social networking site* or social networking website* or social platform* or TikTok or Tumblr or Twitter or "web 2.0" or "web 2.0s" or WeChat or WhatsApp or YouTube)	Expanders - Apply equivalent subjects<br>Search modes - Boolean/Phrase	18,645
S133	(MH "Virtual Reality")	Expanders - Apply equivalent subjects<br>Search modes - Boolean/Phrase	6,754
S134	TI (virtual reality or virtual realities) OR AB (virtual reality or virtual realities)	Expanders - Apply equivalent subjects<br>Search modes - Boolean/Phrase	5,386
S135	(MH "Augmented Reality")	Expanders - Apply equivalent subjects<br>Search modes - Boolean/Phrase	359
S136	TI (augmented realities or augmented reality or mixed realities or mixed reality) OR AB (augmented realities or augmented reality or mixed realities or mixed reality)	Expanders - Apply equivalent subjects<br>Search modes - Boolean/Phrase	1,090
S137	(MH "Holography")	Expanders - Apply equivalent subjects<br>Search modes - Boolean/Phrase	104
S138	TI (hologram* or holograph*) OR AB (hologram* or holograph*)	Expanders - Apply equivalent subjects<br>Search modes - Boolean/Phrase	234
S139	(MH "Printing, Three-Dimensional")	Expanders - Apply equivalent subjects<br>Search modes - Boolean/Phrase	2,344
S140	TI ("3d printing*" or "3 d printing*" or "3 dimensional printing*" or "three dimensional printing*") OR AB ("3d printing*" or "3 d printing*" or "3 dimensional printing*" or "three dimensional printing*")	Expanders - Apply equivalent subjects<br>Search modes - Boolean/Phrase	1,361
S141	TI (chatbot or chatbots or "chat bot" or "chat bots") OR AB (chatbot or chatbots or "chat bot" or "chat bots")	Expanders - Apply equivalent subjects<br>Search modes - Boolean/Phrase	283
S142	TI "virtual care" OR AB "virtual care"	Expanders - Apply equivalent subjects<br>Search modes - Boolean/Phrase	430

S143	TI (closed loop medicines system* or closed loop medication system* or closed loop medicines process* or closed loop medication process*) OR AB (closed loop medicines system* or closed loop medication system* or closed loop medicines process* or closed loop medication process*)	Expanders - Apply equivalent subjects<br>Search modes - Boolean/Phrase	1
S144	TI (bedside station or bedside stations or bed side station or bed side stations or bedside terminal or bedside terminals or bed side terminal or bed side terminals) OR AB (bedside station or bedside stations or bed side station or bed side stations or bedside terminal or bedside terminals or bed side terminal or bed side terminals)	Expanders - Apply equivalent subjects<br>Search modes - Boolean/Phrase	29
S145	TI (predictive analytic OR predictive analytics) OR AB (predictive analytic OR predictive analytics)	Expanders - Apply equivalent subjects<br>Search modes - Boolean/Phrase	394
S146	S25 OR S26 OR S27 OR S28 OR S29 OR S30 OR S31 OR S32 OR S33 OR S34 OR S35 OR S36 OR S37 OR S38 OR S39 OR S40 OR S41 OR S42 OR S43 OR S44 OR S45 OR S46 OR S47 OR S48 OR S49 OR S50 OR S51 OR S52 OR S53 OR S54 OR S55 OR S56 OR S57 OR S58 OR S59 OR S60 OR S61 OR S62 OR S63 OR S64 OR S65 OR S66 OR S67 OR S68 OR S69 OR S70 OR S71 OR S72 OR S73 OR S74 OR S75 OR S76 OR S77 OR S78 OR S79 OR S80 OR S81 OR S82 OR S83 OR S84 OR S85 OR S86 OR S87 OR S88 OR S89 OR S90 OR S91 OR S92 OR S93 OR S94 OR S95 OR S96 OR S97 OR S98 OR S99 OR S100 OR S101 OR S102 OR S103 OR S104 OR S105 OR S106 OR S107 OR S108 OR S109 OR S110 OR S111 OR S112 OR S113 OR S114 OR S115 OR S116 OR S117 OR S118 OR S119 OR S120 OR S121 OR S122 OR S123 OR S124 OR S125 OR S126 OR S127 OR S128 OR S129 OR S130 OR S131 OR S132 OR S133 OR S134 OR S135 OR S136 OR S137 OR S138 OR S139 OR S140 OR S141 OR S142 OR S143 OR S144 OR S145	Expanders - Apply equivalent subjects<br>Search modes - Boolean/Phrase	338,458
S147	S24 AND S146	Expanders - Apply equivalent subjects<br>Search modes - Boolean/Phrase	85,003

S148	TI (champion or champions) OR AB (champion or champions)	Expanders - Apply equivalent subjects<br>Search modes - Boolean/Phrase	4,494
S149	TI (change agent or change agents) OR AB (change agent or change agents)	Expanders - Apply equivalent subjects<br>Search modes - Boolean/Phrase	949
S150	TI (formal leader or formal leaders or informal leader or informal leaders or opinion leader or opinion leaders) OR AB (formal leader or formal leaders or informal leader or informal leaders or opinion leader or opinion leaders)	Expanders - Apply equivalent subjects<br>Search modes - Boolean/Phrase	849
S151	TI (superuser or superusers or super user or super users) OR AB (superuser or superusers or super user or super users)	Expanders - Apply equivalent subjects<br>Search modes - Boolean/Phrase	104
S152	TI (boundary spanner or boundary spanners) OR AB (boundary spanner or boundary spanners)	Expanders - Apply equivalent subjects<br>Search modes - Boolean/Phrase	64
S153	TI (advocate or advocates) OR AB (advocate or advocates)	Expanders - Apply equivalent subjects<br>Search modes - Boolean/Phrase	20,241
S154	TI (supporter or supporters) OR AB (supporter or supporters)	Expanders - Apply equivalent subjects<br>Search modes - Boolean/Phrase	2,019
S155	(MH "Mentorship")	Expanders - Apply equivalent subjects<br>Search modes - Boolean/Phrase	17,362
S156	TI (mentor or mentors) OR AB (mentor or mentors)	Expanders - Apply equivalent subjects<br>Search modes - Boolean/Phrase	7,555
S157	TI (coach or coaches) OR AB (coach or coaches)	Expanders - Apply equivalent subjects<br>Search modes - Boolean/Phrase	7,905
S158	(MH "Peer Group")	Expanders - Apply equivalent subjects<br>Search modes - Boolean/Phrase	15,333
S159	TI ("peer to peer" or peer influence) OR AB ("peer to peer" or peer influence)	Expanders - Apply equivalent subjects<br>Search modes - Boolean/Phrase	1,957
S160	TI ((peer or peers) N2 collaborat*) OR AB ((peer or peers) N2 collaborat*)	Expanders - Apply equivalent subjects<br>Search modes - Boolean/Phrase	305

S161	TI (interpersonal influence*) OR AB (interpersonal influence*)	Expanders - Apply equivalent subjects<br>Search modes - Boolean/Phrase	164
S162	TI (innovator or innovators) OR AB (innovator or innovators)	Expanders - Apply equivalent subjects<br>Search modes - Boolean/Phrase	1,309
S163	TI (ambassador or ambassadors) OR AB (ambassador or ambassadors)	Expanders - Apply equivalent subjects<br>Search modes - Boolean/Phrase	758
S164	S148 OR S149 OR S150 OR S151 OR S152 OR S153 OR S154 OR S155 OR S156 OR S157 OR S158 OR S159 OR S160 OR S161 OR S162 OR S163	Expanders - Apply equivalent subjects<br>Search modes - Boolean/Phrase	73,814
S165	S147 AND S164	Expanders - Apply equivalent subjects<br>Search modes - Boolean/Phrase	1,738
S166	S147 AND S164	Limiters - English Language<br>Expanders - Apply equivalent subjects<br>Search modes - Boolean/Phrase	1,718
S167	S147 AND S164	Limiters - Published Date: 20170101-20221231; English Language<br>Expanders - Apply equivalent subjects<br>Search modes - Boolean/Phrase	955
S168	S147 AND S164	Limiters - Published Date: 20170101-20221231; English Language; Peer Reviewed<br>Expanders - Apply equivalent subjects<br>Search modes - Boolean/Phrase	844

Database: IEEE Explore
Search strategy:

Group 1 Technology Terms – 19 results

(("All Metadata":nurse OR "All Metadata":nursing) OR ("All Metadata":"health personnel" OR "All Metadata":"health employee" OR "All Metadata":"healthcare employee" OR "All Metadata":"health care employee" OR "All Metadata":" "healthcare professional" OR "All Metadata":"health care professional" OR "All Metadata":"healthcare provider" OR "All Metadata":"health care provider" OR "All Metadata":"healthcare personnel" OR "All Metadata":"health care personnel" OR "All

Metadata":"healthcare worker" OR "All Metadata":"health care worker" OR "All Metadata":"medical staff" OR "All Metadata":"health team" OR "All Metadata":"health teams" OR "All Metadata":"healthcare team" OR "All Metadata":"health care team" OR "All Metadata":"interdisciplinary care team" OR "All Metadata":"medical care team" OR "All Metadata":"multidisciplinary care team" OR "All Metadata":"patient care team") OR ("All Metadata":doctor OR "All Metadata":"hospital registrar" OR "All Metadata":physician OR "All Metadata":allergist OR "All Metadata":anesthesiologist OR "All Metadata":cardiologist OR "All Metadata":dermatologist OR "All Metadata":endocrinologist OR "All Metadata":gastroenterologist OR "All Metadata":general practitioner OR "All Metadata":geriatrician OR "All Metadata":hospitalist OR "All Metadata":nephrologist OR "All Metadata":neurologist OR "All Metadata":oncologist OR "All Metadata":ophthalmologist OR "All Metadata":osteopathic physician OR "All Metadata":otolaryngologist OR "All Metadata":pathologist OR "All Metadata":paediatrician OR "All Metadata":pediatrician OR "All Metadata":neonatologist OR "All Metadata":pharmacist OR "All Metadata":physiatrist OR "All Metadata":pulmonologist OR "All Metadata":radiologist OR "All Metadata":rheumatologist OR "All Metadata":surgeon OR "All Metadata":neurosurgeon OR "All Metadata":urologist OR "All Metadata":psychotherapist) OR ("All Metadata":"occupational therapist" OR "All Metadata":"physical therapist" OR "All Metadata":dietician OR "All Metadata":dietitian OR "All Metadata":nutritionist OR "All Metadata":"case worker" OR "All Metadata":caseworker OR "All Metadata":"social worker" OR "All Metadata":"respiratory care practitioner" OR "All Metadata":"respiratory therapist") OR ("All Metadata":logopedist OR "All Metadata":"speech and language pathologist" OR "All Metadata":"speech language pathologist" OR "All Metadata":"speech therapist" OR "All Metadata":"speech language therapist") AND ("All Metadata":telemedicine OR "All Metadata":telecardiology OR "All Metadata":teledermatology OR "All Metadata":telediagnosis OR "All Metadata":telediagnoses OR "All Metadata":telemonitoring OR "All Metadata":telenephrology OR "All Metadata":teleneurology OR "All Metadata":telepsychology OR "All Metadata":teleradiology OR "All Metadata":teleradiotherapy OR "All Metadata":telesurgery OR "All Metadata":teletherapy teletherapies OR "All Metadata":videoconsult OR "All Metadata":videoconsults OR "All Metadata":"tele medicine" OR "All Metadata":"tele cardiology" OR "All Metadata":"tele dermatology" OR "All Metadata":"tele diagnosis" OR "All Metadata":"tele diagnoses" OR "All Metadata":"tele monitoring" OR "All Metadata":"tele nephrology" OR "All Metadata":"tele neurology" OR "All Metadata":"tele psychology" OR "All Metadata":"tele radiology" OR "All Metadata":"tele radiotherapy" OR "All Metadata":"tele surgery" OR "All Metadata":"tele therapy" OR "All Metadata":telehealth OR "All Metadata":"tele health" OR "All Metadata":"e-health" OR "All Metadata":ehealth OR "All Metadata":telenurse OR "All Metadata":"tele nurse" OR "All Metadata":telenursing OR "All Metadata":"tele nursing" OR "All Metadata":"e−rehabilitation" OR "All Metadata":remote rehabilitation OR "All Metadata":"tele rehabilitation" OR "All Metadata":"virtual rehabilitation"))

Search within Results
champion OR "change agent" OR "formal leader" OR "informal leader" OR "opinion leader" OR superuser OR "super user" OR "boundary spanner" OR advocate OR supporter OR mentor OR coach OR coaches OR innovator OR ambassador OR "peer to peer" OR "peer influence" OR "interpersonal influence" OR "peer collaborat*"

Filters Applied
2017-2022
Journals

Group 2 Technology Terms – 16 results

(("All Metadata":"cyber consulting" OR "All Metadata":"cyber consultation" OR "All Metadata":cyberconsult OR "All Metadata":econsult OR "All Metadata":cyberconsultation OR "All Metadata":econsultation OR "All Metadata":"e consult" OR "All Metadata":"e consulting" OR "All Metadata":"e consutlation" OR "All Metadata":"email based consult" OR "All Metadata":"email based consulting" OR "All Metadata":"email based consultation" OR "All Metadata":"online consult" OR "All Metadata":"online consultation" OR "All Metadata":"tele consult" OR "All Metadata":"tele consultation" OR "All Metadata":"telephone based consult" OR "All Metadata":"telephone based consultation" OR "All Metadata":"telephone consult" OR "All Metadata":"telephone consultation" OR "All Metadata":"video consult" OR "All Metadata":"video consultation" OR "All Metadata":"virtual consult" OR "All Metadata":"virtual consultation" OR "All Metadata":"cyber consults" OR "All Metadata":"cyber consultations" OR "All Metadata":cyberconsults OR "All Metadata":econsults OR "All Metadata":cyberconsultations OR "All Metadata":econsultations OR "All Metadata":"e consults" OR "All Metadata":"e consutlations" OR "All Metadata":"email based consults" OR "All Metadata":"email based consultations" OR "All Metadata":"online consults" OR "All Metadata":"online consultations" OR "All Metadata":"tele consults" OR "All Metadata":"tele consultations" OR "All Metadata":"telephone based consults" OR "All Metadata":"telephone based consultations" OR "All Metadata":"telephone consults" OR "All Metadata":"telephone consultations" OR "All Metadata":"video consults" OR "All Metadata":"video consultations" OR "All Metadata":"virtual consults" OR "All Metadata":"virtual consultations" OR "All Metadata":"video conference" OR "All Metadata":"video conferences" OR "All Metadata":video conferencing OR "All Metadata":videoconference OR "All Metadata":videoconferences OR "All Metadata":webcast OR "All Metadata":webcasts OR "All Metadata":webcasting OR "All Metadata":"virtual care") AND ("All Metadata":nurse OR "All Metadata":nursing) OR ("All Metadata":"health personnel" OR "All Metadata":"health employee" OR "All Metadata":"healthcare employee" OR "All Metadata":"health care employee" OR "All Metadata":" "healthcare professional" OR "All Metadata":"health care professional" OR "All Metadata":"healthcare provider" OR "All Metadata":"health care provider" OR "All Metadata":"healthcare personnel" OR "All Metadata":"health care personnel" OR "All Metadata":"healthcare worker" OR "All Metadata":"health care worker" OR "All Metadata":"medical staff" OR "All Metadata":"health team" OR "All Metadata":"health teams" OR "All Metadata":"healthcare team" OR "All Metadata":"health care team" OR "All Metadata":"interdisciplinary care team" OR "All Metadata":"medical care team" OR "All Metadata":"multidisciplinary care team" OR "All Metadata":"patient care team") OR ("All Metadata":doctor OR "All Metadata":"hospital registrar" OR "All Metadata":physician OR "All Metadata":allergist OR "All Metadata":anesthesiologist OR "All Metadata":cardiologist OR "All Metadata":dermatologist OR "All Metadata":endocrinologist OR "All Metadata":gastroenterologist OR "All Metadata":general practitioner OR "All Metadata":geriatrician OR "All Metadata":hospitalist OR "All Metadata":nephrologist OR "All Metadata":neurologist OR "All Metadata":oncologist OR "All Metadata":ophthalmologist OR "All Metadata":osteopathic physician OR "All Metadata":otolaryngologist OR "All Metadata":pathologist OR "All Metadata":paediatrician OR "All Metadata":pediatrician OR "All Metadata":neonatologist OR "All Metadata":pharmacist OR "All Metadata":physiatrist OR "All Metadata":pulmonologist OR "All Metadata":radiologist OR "All Metadata":rheumatologist OR "All Metadata":surgeon OR "All Metadata":neurosurgeon OR "All Metadata":urologist OR "All Metadata":psychotherapist) OR ("All Metadata":"occupational therapist" OR "All Metadata":"physical therapist" OR "All Metadata":dietician OR "All Metadata":dietitian OR "All Metadata":nutritionist OR "All Metadata":"case worker" OR "All Metadata":caseworker OR "All

Metadata":"social worker" OR "All Metadata":"respiratory care practitioner" OR "All Metadata":"respiratory therapist") OR ("All Metadata":logopedist OR "All Metadata":"speech and language pathologist" OR "All Metadata":"speech language pathologist" OR "All Metadata":"speech therapist" OR "All Metadata":"speech language therapist"))

Search within Results
champion OR "change agent" OR "formal leader" OR "informal leader" OR "opinion leader" OR superuser OR "super user" OR "boundary spanner" OR advocate OR supporter OR mentor OR coach OR coaches OR innovator OR ambassador OR "peer to peer" OR "peer influence" OR "interpersonal influence" OR "peer collaborat*"

Filters Applied
2017-2022
Journals

Group 3 Technology Terms - 17 results

(("All Metadata":"medical record" OR "All Metadata":"health record" OR "All Metadata":"patient record" OR "All Metadata":ehr OR "All Metadata":emr OR "All Metadata":"medical records" OR "All Metadata":"health records" OR "All Metadata":"patient records" OR "All Metadata":ehrs OR "All Metadata":emrs OR "All Metadata":"health information" OR "All Metadata":"smart card" OR "All Metadata":"smart cards" OR "All Metadata":"order entry" OR "All Metadata":"information system" OR "All Metadata":"information systems" OR "All Metadata":"information management system" OR "All Metadata":"information management systems" OR "All Metadata":"database management" OR "All Metadata":"decision support" OR "All Metadata":"point of care" OR "All Metadata":bedside OR "All Metadata":"bedside" OR "All Metadata":"electronic prescribing" OR "All Metadata":"e prescribing" OR "All Metadata":"e prescription" OR "All Metadata":"e prescriptions" OR "All Metadata":"electronic prescription" OR "All Metadata":"electronic prescriptions" OR "All Metadata":"closed loop medicines system" OR "All Metadata":"closed loop medicines systems" OR "All Metadata":"closed loop medication system" OR "All Metadata":"closed loop medication systems" OR "All Metadata":"closed loop medicines process" OR "All Metadata":"closed loop medication process") AND ("All Metadata":nurse OR "All Metadata":nursing) OR ("All Metadata":"health personnel" OR "All Metadata":"health employee" OR "All Metadata":"healthcare employee" OR "All Metadata":"health care employee" OR "All Metadata":" "healthcare professional" OR "All Metadata":"health care professional" OR "All Metadata":"healthcare provider" OR "All Metadata":"health care provider" OR "All Metadata":"healthcare personnel" OR "All Metadata":"health care personnel" OR "All Metadata":"healthcare worker" OR "All Metadata":"health care worker" OR "All Metadata":"medical staff" OR "All Metadata":"health team" OR "All Metadata":"health teams" OR "All Metadata":"healthcare team" OR "All Metadata":"health care team" OR "All Metadata":"interdisciplinary care team" OR "All Metadata":"medical care team" OR "All Metadata":"multidisciplinary care team" OR "All Metadata":"patient care team") OR ("All Metadata":doctor OR "All Metadata":"hospital registrar" OR "All Metadata":physician OR "All Metadata":allergist OR "All Metadata":anesthesiologist OR "All Metadata":cardiologist OR "All Metadata":dermatologist OR "All Metadata":endocrinologist OR "All Metadata":gastroenterologist OR "All Metadata":general practitioner OR "All Metadata":geriatrician OR "All Metadata":hospitalist OR "All Metadata":nephrologist OR "All Metadata":neurologist OR "All Metadata":oncologist OR "All Metadata":ophthalmologist OR "All Metadata":osteopathic physician OR "All

Metadata":otolaryngologist OR "All Metadata":pathologist OR "All Metadata":paediatrician OR "All Metadata":pediatrician OR "All Metadata":neonatologist OR "All Metadata":pharmacist OR "All Metadata":physiatrist OR "All Metadata":pulmonologist OR "All Metadata":radiologist OR "All Metadata":rheumatologist OR "All Metadata":surgeon OR "All Metadata":neurosurgeon OR "All Metadata":urologist OR "All Metadata":psychotherapist) OR ("All Metadata":"occupational therapist" OR "All Metadata":"physical therapist" OR "All Metadata":dietician OR "All Metadata":dietitian OR "All Metadata":nutritionist OR "All Metadata":"case worker" OR "All Metadata":caseworker OR "All Metadata":"social worker" OR "All Metadata":"respiratory care practitioner" OR "All Metadata":"respiratory therapist") OR ("All Metadata":logopedist OR "All Metadata":"speech and language pathologist" OR "All Metadata":"speech language pathologist" OR "All Metadata":"speech therapist" OR "All Metadata":"speech language therapist"))

Search within Results
champion OR "change agent" OR "formal leader" OR "informal leader" OR "opinion leader" OR superuser OR "super user" OR "boundary spanner" OR advocate OR supporter OR mentor OR coach OR coaches OR innovator OR ambassador OR "peer to peer" OR "peer influence" OR "interpersonal influence" OR "peer collaborat*"

Filters Applied
2017-2022
Journals

Group 4 Technology Terms – 17 results

(("All Metadata":"mobile app" OR "All Metadata":"mobile apps" OR "All Metadata":"mobile application" OR "All Metadata":"health app" OR "All Metadata":"health apps" OR "All Metadata":"health application" OR "All Metadata":"health applications" OR "All Metadata":"portable software app" OR "All Metadata":"portable software apps" OR "All Metadata":"portable software application" OR "All Metadata":"portable software applications" OR "All Metadata":"tablet app" OR "All Metadata":"tablet apps" OR "All Metadata":"tablet application" OR "All Metadata":"tablet applications" OR "All Metadata":"cell phone" OR "All Metadata":"cell phones" OR "All Metadata":"cellphone" OR "All Metadata":"cellphones" OR "All Metadata":"cellular phone" OR "All Metadata":"cellular phones" OR "All Metadata":"cellular telephone" OR "All Metadata":"cellular telephones" OR "All Metadata":"mobile phone" OR "All Metadata":"mobile phones" OR "All Metadata":"mobile telephone" OR "All Metadata":"mobile telephones" OR "All Metadata":smartphone OR "All Metadata":smartphones OR "All Metadata":"smart phones" OR "All Metadata":"smart phones" OR "All Metadata":"text messaging" OR "All Metadata":texting) AND ("All Metadata":nurse OR "All Metadata":nursing) OR ("All Metadata":"health personnel" OR "All Metadata":"health employee" OR "All Metadata":"healthcare employee" OR "All Metadata":"health care employee" OR "All Metadata":" healthcare professional" OR "All Metadata":"health care professional" OR "All Metadata":"healthcare provider" OR "All Metadata":"health care provider" OR "All Metadata":"healthcare personnel" OR "All Metadata":"health care personnel" OR "All Metadata":"healthcare worker" OR "All Metadata":"health care worker" OR "All Metadata":"medical staff" OR "All Metadata":"health team" OR "All Metadata":"health teams" OR "All Metadata":"healthcare team" OR "All Metadata":"health care team" OR "All Metadata":"interdisciplinary care team" OR "All Metadata":"medical care team" OR "All Metadata":"multidisciplinary care team" OR "All Metadata":"patient care team") OR ("All

Metadata":doctor OR "All Metadata":"hospital registrar" OR "All Metadata":physician OR "All Metadata":allergist OR "All Metadata":anesthesiologist OR "All Metadata":cardiologist OR "All Metadata":dermatologist OR "All Metadata":endocrinologist OR "All Metadata":gastroenterologist OR "All Metadata":general practitioner OR "All Metadata":geriatrician OR "All Metadata":hospitalist OR "All Metadata":nephrologist OR "All Metadata":neurologist OR "All Metadata":oncologist OR "All Metadata":ophthalmologist OR "All Metadata":osteopathic physician OR "All Metadata":otolaryngologist OR "All Metadata":pathologist OR "All Metadata":paediatrician OR "All Metadata":pediatrician OR "All Metadata":neonatologist OR "All Metadata":pharmacist OR "All Metadata":physiatrist OR "All Metadata":pulmonologist OR "All Metadata":radiologist OR "All Metadata":rheumatologist OR "All Metadata":surgeon OR "All Metadata":neurosurgeon OR "All Metadata":urologist OR "All Metadata":psychotherapist) OR ("All Metadata":"occupational therapist" OR "All Metadata":"physical therapist" OR "All Metadata":dietician OR "All Metadata":dietitian OR "All Metadata":nutritionist OR "All Metadata":"case worker" OR "All Metadata":caseworker OR "All Metadata":"social worker" OR "All Metadata":"respiratory care practitioner" OR "All Metadata":"respiratory therapist") OR ("All Metadata":logopedist OR "All Metadata":"speech and language pathologist" OR "All Metadata":"speech language pathologist" OR "All Metadata":"speech therapist" OR "All Metadata":"speech language therapist"))

Search within Results
champion OR "change agent" OR "formal leader" OR "informal leader" OR "opinion leader" OR superuser OR "super user" OR "boundary spanner" OR advocate OR supporter OR mentor OR coach OR coaches OR innovator OR ambassador OR "peer to peer" OR "peer influence" OR "interpersonal influence" OR "peer collaborat*"

Filters Applied
2017-2022
Journals

Group 5 Technology Terms – 101 results

(("All Metadata":"social media" OR "All Metadata":"social medium" OR "All Metadata":Facebook OR "All Metadata":Flickr OR "All Metadata":Instagram OR "All Metadata":LinkedIn OR "All Metadata":MySpace OR "All Metadata":Pinterest OR "All Metadata":Reddit OR "All Metadata":"Sina Weibo" OR "All Metadata":Snapchat OR "All Metadata":"online social network" OR "All Metadata":"social networking platform" OR "All Metadata":"social networking site" OR "All Metadata":"social networking website" OR "All Metadata":"social platform" OR "All Metadata":TikTok OR "All Metadata":Tumblr OR "All Metadata":Twitter OR "All Metadata":"web 2.0" OR "All Metadata":"web 2.0s" OR "All Metadata":WeChat OR "All Metadata":WhatsApp OR "All Metadata":YouTube OR "All Metadata":chatbot OR "All Metadata":chatbots OR "All Metadata":"chat bot" OR "All Metadata":"chat bots" OR "All Metadata":"smart technology" OR "All Metadata":"smart technologies" OR "All Metadata":wearable OR "All Metadata":wearables OR "All Metadata":"activity tracker" OR "All Metadata":"activity trackers" OR "All Metadata":"fitness tracker" OR "All Metadata":"fitness trackers" OR "All Metadata":smartwatch OR "All Metadata":"smart watch" OR "All Metadata":"smart watches" OR "All Metadata":pedometer OR "All Metadata":pedometers OR "All Metadata":smartglasses OR "All Metadata":"smart glasses" OR "All Metadata":"virtual reality" OR "All Metadata":"virtual realities" OR "All Metadata":"augmented realities" OR "All Metadata":"augmented reality" OR "All Metadata":"mixed

realities" OR "All Metadata":"mixed reality" OR "All Metadata":hologram OR "All Metadata":holograms OR "All Metadata":holograph OR "All Metadata":holographs OR "All Metadata":"3d printing" OR "All Metadata":"3 d printing*" OR "All Metadata":"3 dimensional printing" OR "All Metadata":"three dimensional printing") OR ("All Metadata":wireless OR "All Metadata":internet OR "All Metadata":web OR "All Metadata":webbased OR "All Metadata":"web based") AND ("All Metadata":nurse OR "All Metadata":nursing) OR ("All Metadata":"health personnel" OR "All Metadata":"health employee" OR "All Metadata":"healthcare employee" OR "All Metadata":"health care employee" OR "All Metadata":" "healthcare professional" OR "All Metadata":"health care professional" OR "All Metadata":"healthcare provider" OR "All Metadata":"health care provider" OR "All Metadata":"healthcare personnel" OR "All Metadata":"health care personnel" OR "All Metadata":"healthcare worker" OR "All Metadata":"health care worker" OR "All Metadata":"medical staff" OR "All Metadata":"health team" OR "All Metadata":"health teams" OR "All Metadata":"healthcare team" OR "All Metadata":"health care team" OR "All Metadata":"interdisciplinary care team" OR "All Metadata":"medical care team" OR "All Metadata":"multidisciplinary care team" OR "All Metadata":"patient care team") OR ("All Metadata":doctor OR "All Metadata":"hospital registrar" OR "All Metadata":physician OR "All Metadata":allergist OR "All Metadata":anesthesiologist OR "All Metadata":cardiologist OR "All Metadata":dermatologist OR "All Metadata":endocrinologist OR "All Metadata":gastroenterologist OR "All Metadata":general practitioner OR "All Metadata":geriatrician OR "All Metadata":hospitalist OR "All Metadata":nephrologist OR "All Metadata":neurologist OR "All Metadata":oncologist OR "All Metadata":ophthalmologist OR "All Metadata":osteopathic physician OR "All Metadata":otolaryngologist OR "All Metadata":pathologist OR "All Metadata":paediatrician OR "All Metadata":pediatrician OR "All Metadata":neonatologist OR "All Metadata":pharmacist OR "All Metadata":physiatrist OR "All Metadata":pulmonologist OR "All Metadata":radiologist OR "All Metadata":rheumatologist OR "All Metadata":surgeon OR "All Metadata":neurosurgeon OR "All Metadata":urologist OR "All Metadata":psychotherapist) OR ("All Metadata":"occupational therapist" OR "All Metadata":"physical therapist" OR "All Metadata":dietician OR "All Metadata":dietitian OR "All Metadata":nutritionist OR "All Metadata":"case worker" OR "All Metadata":caseworker OR "All Metadata":"social worker" OR "All Metadata":"respiratory care practitioner" OR "All Metadata":"respiratory therapist") OR ("All Metadata":logopedist OR "All Metadata":"speech and language pathologist" OR "All Metadata":"speech language pathologist" OR "All Metadata":"speech therapist" OR "All Metadata":"speech language therapist"))

Search within Results
champion OR "change agent" OR "formal leader" OR "informal leader" OR "opinion leader" OR superuser OR "super user" OR "boundary spanner" OR advocate OR supporter OR mentor OR coach OR coaches OR innovator OR ambassador OR "peer to peer" OR "peer influence" OR "interpersonal influence" OR "peer collaborat*"

Filters Applied
2017-2022
Journals

Group 6 Technology Terms -

(("All Metadata":"artificial intelligence" OR "All Metadata":"machine intelligence" OR "All Metadata":"expert system" OR "All Metadata":"expert systems" OR "All Metadata":"knowledge based

system" OR "All Metadata":"knowledge based systems" OR "All Metadata":"fuzzy logic" OR "All Metadata":"fuzzy model" OR "All Metadata":"fuzzy models" OR "All Metadata":"machine learning" OR "All Metadata":"learning machine" OR "All Metadata":"learning machines" OR "All Metadata":"deep learning" OR "All Metadata":"hierarchical learning" OR "All Metadata":"ann approach" OR "All Metadata":"ann model" OR "All Metadata":"ann method" OR "All Metadata":"ann training" OR "All Metadata":"neural network" OR "All Metadata":"neural networks" OR "All Metadata":robotics OR "All Metadata":nanorobotics OR "All Metadata":robot OR "All Metadata":robots OR "All Metadata":"biomedical technology" OR "All Metadata":"bio medical technology" OR "All Metadata":"biomedical technologies" OR "All Metadata":"bio medical technologies") AND ("All Metadata":nurse OR "All Metadata":nursing) OR ("All Metadata":"health personnel" OR "All Metadata":"health employee" OR "All Metadata":"healthcare employee" OR "All Metadata":"health care employee" OR "All Metadata":" "healthcare professional" OR "All Metadata":"health care professional" OR "All Metadata":"healthcare provider" OR "All Metadata":"health care provider" OR "All Metadata":"healthcare personnel" OR "All Metadata":"health care personnel" OR "All Metadata":"healthcare worker" OR "All Metadata":"health care worker" OR "All Metadata":"medical staff" OR "All Metadata":"health team" OR "All Metadata":"health teams" OR "All Metadata":"healthcare team" OR "All Metadata":"health care team" OR "All Metadata":"interdisciplinary care team" OR "All Metadata":"medical care team" OR "All Metadata":"multidisciplinary care team" OR "All Metadata":"patient care team") OR ("All Metadata":doctor OR "All Metadata":"hospital registrar" OR "All Metadata":physician OR "All Metadata":allergist OR "All Metadata":anesthesiologist OR "All Metadata":cardiologist OR "All Metadata":dermatologist OR "All Metadata":endocrinologist OR "All Metadata":gastroenterologist OR "All Metadata":general practitioner OR "All Metadata":geriatrician OR "All Metadata":hospitalist OR "All Metadata":nephrologist OR "All Metadata":neurologist OR "All Metadata":oncologist OR "All Metadata":ophthalmologist OR "All Metadata":osteopathic physician OR "All Metadata":otolaryngologist OR "All Metadata":pathologist OR "All Metadata":paediatrician OR "All Metadata":pediatrician OR "All Metadata":neonatologist OR "All Metadata":pharmacist OR "All Metadata":physiatrist OR "All Metadata":pulmonologist OR "All Metadata":radiologist OR "All Metadata":rheumatologist OR "All Metadata":surgeon OR "All Metadata":neurosurgeon OR "All Metadata":urologist OR "All Metadata":psychotherapist) OR ("All Metadata":"occupational therapist" OR "All Metadata":"physical therapist" OR "All Metadata":dietician OR "All Metadata":dietitian OR "All Metadata":nutritionist OR "All Metadata":"case worker" OR "All Metadata":caseworker OR "All Metadata":"social worker" OR "All Metadata":"respiratory care practitioner" OR "All Metadata":"respiratory therapist") OR ("All Metadata":logopedist OR "All Metadata":"speech and language pathologist" OR "All Metadata":"speech language pathologist" OR "All Metadata":"speech therapist" OR "All Metadata":"speech language therapist"))

Search within Results
champion OR "change agent" OR "formal leader" OR "informal leader" OR "opinion leader" OR superuser OR "super user" OR "boundary spanner" OR advocate OR supporter OR mentor OR coach OR coaches OR innovator OR ambassador OR "peer to peer" OR "peer influence" OR "interpersonal influence" OR "peer collaborat*"

Filters Applied
2017-2022
Journals

Group 7 Technology Terms - 254 Results

(("All Metadata":informatics OR "All Metadata":"clinical information science" OR "All Metadata":"clinical information technology" OR "All Metadata":"clinical information technologies" OR "All Metadata":"health information science" OR "All Metadata":"health information technology" OR "All Metadata":"health information technologies" OR "All Metadata":"medical computer science" OR "All Metadata":"medical information science" OR "All Metadata":"medical information technology" OR "All Metadata":"medical information technology" OR "All Metadata":"automatic diagnosis" OR "All Metadata":"computer assisted diagnosis" OR "All Metadata":"computer diagnosis" OR "All Metadata":"automatic diagnoses" OR "All Metadata":"computer assisted diagnoses" OR "All Metadata":"computer diagnoses" OR "All Metadata":"information system" OR "All Metadata":"information systems" OR "All Metadata":"online system" OR "All Metadata":"online systems" OR "All Metadata":"computer interface" OR "All Metadata":"computer interefaces" OR "All Metadata":"computer user interface" OR "All Metadata":"computer user interfaces") OR ("All Metadata":"predictive analytics") AND ("All Metadata":nurse OR "All Metadata":nursing) OR ("All Metadata":"health personnel" OR "All Metadata":"health employee" OR "All Metadata":"healthcare employee" OR "All Metadata":"health care employee" OR "All Metadata":" "healthcare professional" OR "All Metadata":"health care professional" OR "All Metadata":"healthcare provider" OR "All Metadata":"health care provider" OR "All Metadata":"healthcare personnel" OR "All Metadata":"health care personnel" OR "All Metadata":"healthcare worker" OR "All Metadata":"health care worker" OR "All Metadata":"medical staff" OR "All Metadata":"health team" OR "All Metadata":"health teams" OR "All Metadata":"healthcare team" OR "All Metadata":"health care team" OR "All Metadata":"interdisciplinary care team" OR "All Metadata":"medical care team" OR "All Metadata":"multidisciplinary care team" OR "All Metadata":"patient care team") OR ("All Metadata":doctor OR "All Metadata":"hospital registrar" OR "All Metadata":physician OR "All Metadata":allergist OR "All Metadata":anesthesiologist OR "All Metadata":cardiologist OR "All Metadata":dermatologist OR "All Metadata":endocrinologist OR "All Metadata":gastroenterologist OR "All Metadata":general practitioner OR "All Metadata":geriatrician OR "All Metadata":hospitalist OR "All Metadata":nephrologist OR "All Metadata":neurologist OR "All Metadata":oncologist OR "All Metadata":ophthalmologist OR "All Metadata":osteopathic physician OR "All Metadata":otolaryngologist OR "All Metadata":pathologist OR "All Metadata":paediatrician OR "All Metadata":pediatrician OR "All Metadata":neonatologist OR "All Metadata":pharmacist OR "All Metadata":physiatrist OR "All Metadata":pulmonologist OR "All Metadata":radiologist OR "All Metadata":rheumatologist OR "All Metadata":surgeon OR "All Metadata":neurosurgeon OR "All Metadata":urologist OR "All Metadata":psychotherapist) OR ("All Metadata":"occupational therapist" OR "All Metadata":"physical therapist" OR "All Metadata":dietician OR "All Metadata":dietitian OR "All Metadata":nutritionist OR "All Metadata":"case worker" OR "All Metadata":caseworker OR "All Metadata":"social worker" OR "All Metadata":"respiratory care practitioner" OR "All Metadata":"respiratory therapist") OR ("All Metadata":logopedist OR "All Metadata":"speech and language pathologist" OR "All Metadata":"speech language pathologist" OR "All Metadata":"speech therapist" OR "All Metadata":"speech language therapist"))

Search within Results
champion OR "change agent" OR "formal leader" OR "informal leader" OR "opinion leader" OR superuser OR "super user" OR "boundary spanner" OR advocate OR supporter OR mentor OR coach OR

coaches OR innovator OR ambassador OR "peer to peer" OR "peer influence" OR "interpersonal influence" OR "peer collaborat*"

Filters Applied
2017-2022
Journals

Indirect Search for Recommendation Question #3: Should the implementation of interdisciplinary peer champion models in health-service organizations be recommended or not to facilitate education for health providers on the use of digital health technologies?

Dates searched: January 1, 2017 – March 27, 2023

Databases searched: MEDLINE, Embase and CINAHL

**Note: The original search strategy was broadened to examine peer champion models in general (not specific to digital health technologies). The search was only applied to MEDLINE, Embase, and CINAHL for feasibility purposes, and limited to systematic reviews in order to obtain the highest quality evidence.*

Database: Medline
Search strategy:

#	Searches	Results
1	exp "Attitude of Health Personnel"/	169181
2	exp Health Personnel/	605251
3	exp Personnel, Hospital/	95098
4	exp Health Occupations/	1843118
5	exp Patient Care Team/	72633
6	Social Work/	16227
7	Social Workers/	1069
8	Nurse's Role/	42735
9	Licensed Practical Nurses/	99
10	exp Nurses/	97637
11	exp Nursing Staff/	69674
12	Nursing Staff, Hospital/	47830

13	exp Nursing/	262877
14	Nursing, Practical/	3444
15	Health Educators/	492
16	exp Administrative Personnel/	42241
17	(health* adj2 (provider? or staff? or personnel? or employe* or profession* or occupation? or practitioner? or worker?)).tw,kf.	318866
18	(hospital* adj2 (provider? or staff? or personnel? or employe* or profession* or occupation? or practitioner? or worker?)).tw,kf.	21251
19	nurs*.tw,kf.	526437
20	(patient care? adj2 team?).tw,kf.	1035
21	(health* adj2 educator?).tw,kf.	3822
22	(social adj2 worker?).tw,kf.	12626
23	(support* adj2 (worker? or staff?)).tw,kf.	8172
24	clinician?.tw,kf.	291257
25	doctor?.tw,kf.	143575
26	physician?.tw,kf.	453388
27	practitioner?.tw,kf.	177401
28	surgeon?.tw,kf.	245306
29	Caregivers/	49061
30	(caregiver? or care-giver? or carer or carers).tw,kf.	106863
31	or/1-30	3649458
32	(champion or champions).tw,kf.	6186
33	(change agent or change agents).tw,kf.	1193
34	(formal leader or formal leaders or informal leader or informal leaders or opinion leader or opinion leaders).tw,kf.	1856
35	(superuser or superusers or super user or super users).tw,kf.	121
36	(boundary spanner or boundary spanners).tw,kf.	85
37	(advocate or advocates).tw,kf.	37776
38	(supporter or supporters).tw,kf.	3941
39	mentors/	12906
40	Mentoring/	3790

41	(mentor or mentors).tw,kf.	10747
42	(coach or coaches).tw,kf.	11734
43	Peer Group/ or Peer Influence/	24551
44	("peer to peer" or peer influence).tw,kf.	3477
45	((peer or peers) adj3 collaborat*).tw,kf.	466
46	interpersonal influence*.tw,kf.	273
47	(innovator or innovators).tw,kf.	2558
48	(ambassador or ambassadors).tw,kf.	718
49	or/32-48 [Champion Terms]	111397
50	31 and 49	45059
51	limit 50 to english language	43740
52	limit 51 to yr="2017 -Current"	17173
53	review/	3126602
54	(medline or medlars or pubmed or grateful med or CINAHL or scisearch or psychinfo or psycinfo or psychlit or psyclit or handsearch* or hand search* or manual* search* or electronic database* or bibliographic database* or embase or lilacs or scopus or "web of science").mp.	374457
55	53 and 54	198448
56	meta-analysis.mp.	270229
57	meta-analysis as topic/	22215
58	meta-analysis/	178060
59	meta-analysis.tw.	226027
60	systematic review*.tw,kf.	281884
61	cochrane database*.jn.	16190
62	or/55-61	512149
63	52 and 62	861

Database: Embase
Search strategy:

#	Searches	Results

1	exp health personnel attitude/	205704
2	exp health care personnel/	2023718
3	exp hospital personnel/	155883
4	exp multidisciplinary team/	26440
5	social work/	30702
6	social worker/	16785
7	licensed practical nurse/	395
8	exp nurse/	222655
9	nursing staff/	77988
10	exp nursing/	418178
11	practical nursing/	169
12	exp health educator/	3531
13	exp administrative personnel/	35399
14	nurse attitude/	43377
15	(health* adj2 (provider? or staff? or personnel? or employe* or profession* or occupation? or practitioner? or worker?)).tw,kw.	415550
16	(hospital* adj2 (provider? or staff? or personnel? or employe* or profession* or occupation? or practitioner? or worker?)).tw,kw.	29750
17	nurs*.tw,kw.	637727
18	(patient care? adj2 team?).tw,kw.	882
19	(health* adj2 educator?).tw,kw.	4442
20	(social adj2 worker?).tw,kw.	21562
21	(support* adj2 (worker? or staff?)).tw,kw.	11746
22	clinician?.tw,kw.	426095
23	doctor?.tw,kw.	216117
24	physician?.tw,kw.	671186
25	practitioner?.tw,kw.	231276
26	surgeon?.tw,kw.	392803
27	caregiver/	110774
28	(caregiver? or care-giver? or carer or carers).tw,kw.	153206
29	or/1-28	3862091

30	(champion or champions).tw,kw.	9219
31	(change agent or change agents).tw,kw.	1406
32	(formal leader or formal leaders or informal leader or informal leaders or opinion leader or opinion leaders).tw,kw.	2543
33	(superuser or superusers or super user or super users).tw,kw.	259
34	(boundary spanner or boundary spanners).tw,kw.	89
35	(advocate or advocates).tw,kw.	52377
36	(supporter or supporters).tw,kw.	5006
37	mentor/	10742
38	mentoring/	6377
39	(mentor or mentors).tw,kw.	14412
40	(coach or coaches).tw,kw.	14102
41	Peer Group/	30003
42	("peer to peer" or peer influence).tw,kw.	4499
43	((peer or peers) adj3 collaborat*).tw,kw.	575
44	interpersonal influence*.tw,kw.	319
45	(innovator or innovators).tw,kw.	3801
46	(ambassador or ambassadors).tw,kw.	1096
47	or/30-46	143251
48	29 and 47	58769
49	limit 48 to english language	57428
50	limit 49 to yr="2017 -Current"	29492
51	"review"/	2942034
52	(medline or medlars or pubmed or grateful med or CINAHL or scisearch or psychinfo or psycinfo or psychlit or psyclit or handsearch* or hand search* or manual* search* or electronic database* or bibliographic database* or embase or lilacs or scopus or web of science).mp.	489920
53	51 and 52	197396
54	meta-analysis.mp.	411794
55	"meta analysis (topic)"/	52343
56	meta analysis/	287663

57	systematic review*.tw,kw.	357703
58	cochrane database*.jn.	23264
59	or/53-58	677092
60	50 and 59	1097

Database: Cumulative Index to Nursing and Allied Health (CINAHL)
Search strategy:

#	Query	Limiters/Expanders	Results
S1	(MH "Attitude of Health Personnel+")	Expanders - Apply equivalent subjects<br>Search modes - Boolean/Phrase	123,789
S2	(MH "Health Personnel+")	Expanders - Apply equivalent subjects<br>Search modes - Boolean/Phrase	642,122
S3	(MH "Personnel, Health Facility+")	Expanders - Apply equivalent subjects<br>Search modes - Boolean/Phrase	45,150
S4	(MH "Health Occupations+")	Expanders - Apply equivalent subjects<br>Search modes - Boolean/Phrase	826,622
S5	(MH "Multidisciplinary Care Team+")	Expanders - Apply equivalent subjects<br>Search modes - Boolean/Phrase	52,145
S6	(MH "Social Work+")	Expanders - Apply equivalent subjects<br>Search modes - Boolean/Phrase	15,488
S7	(MH "Social Workers")	Expanders - Apply equivalent subjects<br>Search modes - Boolean/Phrase	11,309
S8	(MH "Nursing Role")	Expanders - Apply equivalent subjects<br>Search modes - Boolean/Phrase	65,930
S9	(MH "Practical Nurses") OR (MH "Practical Nursing")	Expanders - Apply equivalent subjects<br>Search modes - Boolean/Phrase	7,734
S10	(MH "Nurses+")	Expanders - Apply equivalent subjects<br>Search modes - Boolean/Phrase	242,910

S11	(MH "Nursing Staff, Hospital")	Expanders - Apply equivalent subjects<br>Search modes - Boolean/Phrase	25,324
S12	(MH "Health Educators+")	Expanders - Apply equivalent subjects<br>Search modes - Boolean/Phrase	4,211
S13	(MH "Administrative Personnel+")	Expanders - Apply equivalent subjects<br>Search modes - Boolean/Phrase	40,913
S14	TI (health* N2 provider#) OR AB (health* N2 provider#)	Expanders - Apply equivalent subjects<br>Search modes - Boolean/Phrase	58,152
S15	TI (hospital* N2 provider#) OR AB (hospital* N2 provider#)	Expanders - Apply equivalent subjects<br>Search modes - Boolean/Phrase	1,843
S16	TI (health* N2 staff#) OR AB (health* N2 staff#)	Expanders - Apply equivalent subjects<br>Search modes - Boolean/Phrase	9,057
S17	TI (hospital* N2 staff#) OR AB (hospital* N2 staff#)	Expanders - Apply equivalent subjects<br>Search modes - Boolean/Phrase	6,110
S18	TI (health* N2 personnel#) OR AB (health* N2 personnel#)	Expanders - Apply equivalent subjects<br>Search modes - Boolean/Phrase	5,479
S19	TI (hospital* N2 personnel#) OR AB (hospital* N2 personnel#)	Expanders - Apply equivalent subjects<br>Search modes - Boolean/Phrase	1,164
S20	TI (health* N2 employe*) OR AB (health* N2 employe*)	Expanders - Apply equivalent subjects<br>Search modes - Boolean/Phrase	7,186
S21	TI (hospital* N2 employe*) OR AB (hospital* N2 employe*)	Expanders - Apply equivalent subjects<br>Search modes - Boolean/Phrase	2,082
S22	TI (health* N2 profession*) OR AB (health* N2 profession*)	Expanders - Apply equivalent subjects<br>Search modes - Boolean/Phrase	101,885
S23	TI (hospital* N2 profession*) OR AB (hospital* N2 profession*)	Expanders - Apply equivalent subjects<br>Search modes - Boolean/Phrase	2,012
S24	TI (health* N2 occupation#) OR AB (health* N2 occupation#)	Expanders - Apply equivalent subjects<br>Search modes - Boolean/Phrase	1,027

S25	TI (hospital* N2 occupation#) OR AB (hospital* N2 occupation#)	Expanders - Apply equivalent subjects<br>Search modes - Boolean/Phrase	59
S26	TI (health* N2 practitioner#) OR AB (health* N2 practitioner#)	Expanders - Apply equivalent subjects<br>Search modes - Boolean/Phrase	12,312
S27	TI (hospital* N2 practitioner#) OR AB (hospital* N2 practitioner#)	Expanders - Apply equivalent subjects<br>Search modes - Boolean/Phrase	935
S28	TI (health* N2 worker#) OR AB (health* N2 worker#)	Expanders - Apply equivalent subjects<br>Search modes - Boolean/Phrase	34,552
S29	TI (hospital* N2 worker#) OR AB (hospital* N2 worker#)	Expanders - Apply equivalent subjects<br>Search modes - Boolean/Phrase	1,614
S30	TI nurs* OR AB nurs*	Expanders - Apply equivalent subjects<br>Search modes - Boolean/Phrase	644,212
S31	TI (patient care# N2 team#) OR AB (patient care# N2 team#)	Expanders - Apply equivalent subjects<br>Search modes - Boolean/Phrase	597
S32	TI (health* N2 educator#) OR AB (health* N2 educator#)	Expanders - Apply equivalent subjects<br>Search modes - Boolean/Phrase	3,792
S33	TI (social N2 worker#) OR AB (social N2 worker#)	Expanders - Apply equivalent subjects<br>Search modes - Boolean/Phrase	15,158
S34	TI (support* N2 (worker# or staff#)) OR AB (support* N2 (worker# or staff#))	Expanders - Apply equivalent subjects<br>Search modes - Boolean/Phrase	9,783
S35	TI clinician# OR AB clinician#	Expanders - Apply equivalent subjects<br>Search modes - Boolean/Phrase	124,364
S36	TI doctor# OR AB doctor#	Expanders - Apply equivalent subjects<br>Search modes - Boolean/Phrase	65,773
S37	TI physician# OR AB physician#	Expanders - Apply equivalent subjects<br>Search modes - Boolean/Phrase	170,419
S38	TI practitioner# OR AB practitioner#	Expanders - Apply equivalent subjects<br>Search modes - Boolean/Phrase	108,772

S39	TI surgeon# OR AB surgeon#	Expanders - Apply equivalent subjects<br>Search modes - Boolean/Phrase	63,827
S40	(MH "Caregivers")	Expanders - Apply equivalent subjects<br>Search modes - Boolean/Phrase	43,255
S41	TI (caregiver# or care-giver# or carer or carers) OR AB (caregiver# or care-giver# or carer or carers)	Expanders - Apply equivalent subjects<br>Search modes - Boolean/Phrase	80,512
S42	S1 OR S2 OR S3 OR S4 OR S5 OR S6 OR S7 OR S8 OR S9 OR S10 OR S11 OR S12 OR S13 OR S14 OR S15 OR S16 OR S17 OR S18 OR S19 OR S20 OR S21 OR S22 OR S23 OR S24 OR S25 OR S26 OR S27 OR S28 OR S29 OR S30 OR S31 OR S32 OR S33 OR S34 OR S35 OR S36 OR S37 OR S38 OR S39 OR S40 OR S41	Expanders - Apply equivalent subjects<br>Search modes - Boolean/Phrase	2,207,831
S43	TI (champion or champions) OR AB (champion or champions)	Expanders - Apply equivalent subjects<br>Search modes - Boolean/Phrase	4,836
S44	TI ("change agent" or "change agents") OR AB ("change agent" or "change agents")	Expanders - Apply equivalent subjects<br>Search modes - Boolean/Phrase	1,016
S45	TI ("formal leader" or "formal leaders" or "informal leader" or "informal leaders" or "opinion leader" or "opinion leaders") or "AB ("formal leader" or "formal leaders" or "informal leader" or "informal leaders" or "opinion leader" or "opinion leaders")	Expanders - Apply equivalent subjects<br>Search modes - Boolean/Phrase	164
S46	TI (superuser or superusers or "super user" or "super users") OR AB (superuser or superusers or "super user" or "super users")	Expanders - Apply equivalent subjects<br>Search modes - Boolean/Phrase	115
S47	TI ("boundary spanner" or "boundary spanners") OR AB ("boundary spanner" or "boundary spanners")	Expanders - Apply equivalent subjects<br>Search modes - Boolean/Phrase	68
S48	TI (advocate or advocates) OR AB (advocate or advocates)	Expanders - Apply equivalent subjects<br>Search modes - Boolean/Phrase	21,632
S49	TI (supporter or supporters) OR AB (supporter or supporters)	Expanders - Apply equivalent subjects<br>Search modes - Boolean/Phrase	2,218
S50	(MH "Mentorship")	Expanders - Apply equivalent subjects<br>Search modes - Boolean/Phrase	18,257

S51	TI (mentor or mentors) OR AB (mentor or mentors)	Expanders - Apply equivalent subjects<br>Search modes - Boolean/Phrase	8,090
S52	TI (coach or coaches) OR AB (coach or coaches)	Expanders - Apply equivalent subjects<br>Search modes - Boolean/Phrase	8,479
S53	(MH "Peer Group")	Expanders - Apply equivalent subjects<br>Search modes - Boolean/Phrase	16,301
S54	TI ("peer to peer" or "peer influence") OR AB ("peer to peer" or "peer influence")	Expanders - Apply equivalent subjects<br>Search modes - Boolean/Phrase	1,755
S55	TI ((peer or peers) N2 collaborat*) OR AB ((peer or peers) N2 collaborat*)	Expanders - Apply equivalent subjects<br>Search modes - Boolean/Phrase	327
S56	TI ("interpersonal influence*") OR AB ("interpersonal influence*")	Expanders - Apply equivalent subjects<br>Search modes - Boolean/Phrase	177
S57	TI (innovator or innovators) OR AB (innovator or innovators)	Expanders - Apply equivalent subjects<br>Search modes - Boolean/Phrase	1,389
S58	TI (ambassador or ambassadors) OR AB (ambassador or ambassadors)	Expanders - Apply equivalent subjects<br>Search modes - Boolean/Phrase	823
S59	S43 OR S44 OR S45 OR S46 OR S47 OR S48 OR S49 OR S50 OR S51 OR S52 OR S53 OR S54 OR S55 OR S56 OR S57 OR S58	Expanders - Apply equivalent subjects<br>Search modes - Boolean/Phrase	77,897
S60	S42 AND S59	Expanders - Apply equivalent subjects<br>Search modes - Boolean/Phrase	39,311
S61	(MH "Literature Review")	Expanders - Apply equivalent subjects<br>Search modes - Boolean/Phrase	9,051
S62	TX (medline OR medlars OR pubmed OR grateful med OR CINAHL OR scisearch OR psychinfo OR psycinfo OR psychlit OR psyclit OR handsearch* OR "hand search*" OR "manual* search*" OR "electronic database*" OR "bibliographic database*" OR embase OR lilacs OR scopus OR "web of science")	Expanders - Apply equivalent subjects<br>Search modes - Boolean/Phrase	276,179
S63	S61 AND S62	Expanders - Apply equivalent subjects<br>Search modes - Boolean/Phrase	2,421

S64	TI ("meta-analysis") OR AB ("meta-analysis")	Expanders - Apply equivalent subjects<br>Search modes - Boolean/Phrase	91,838
S65	(MH "Meta Analysis")	Expanders - Apply equivalent subjects<br>Search modes - Boolean/Phrase	69,030
S66	TI ("systematic review*") OR AB ("systematic review*")	Expanders - Apply equivalent subjects<br>Search modes - Boolean/Phrase	139,438
S67	JN cochrane database*	Expanders - Apply equivalent subjects<br>Search modes - Boolean/Phrase	6,023
S68	S63 OR S64 OR S65 OR S66 OR S67	Expanders - Apply equivalent subjects<br>Search modes - Boolean/Phrase	189,878
S69	S59 AND S68	Expanders - Apply equivalent subjects<br>Search modes - Boolean/Phrase	1,280
S70	S59 AND S68	Limiters - Published Date: 20170101-20231231; English Language; Peer Reviewed<br>Expanders - Apply equivalent subjects<br>Search modes - Boolean/Phrase	748

<u>Recommendation Question #4:</u> Should leveraging predictive analytics (e.g., command centers and risk assessment software tools) for nurses providing care in all practice settings be recommended to inform clinical decision-making and improve clinical outcomes?

Dates searched: January 1, 2017 – July 5, 2022

Databases searched:
- MEDLINE
- MEDLINE Epub Ahead of Print and In-Process
- Embase
- Emcare Nursing
- Cochrane Central Register of Controlled Trials
- Cochrane Database of Systematic Reviews
- APA PsychInfo
- Cumulative Index to Nursing and Allied Health (CINAHL)
- IEEE Explore *(search run July 11, 2022)*

Database: Medline
Search strategy:

#	Searches	Results
1	nurses/ or nurse administrators/ or nurse practitioners/ or family nurse practitioners/ or pediatric nurse practitioners/ or nurse specialists/ or nurse anesthetists/ or nurse clinicians/ or nurse midwives/ or nurses, pediatric/ or nurses, neonatal/ or nurses, community health/ or nurses, public health/	94118
2	nursing staff/ or nursing staff, hospital/	69103
3	nursing care/ or cardiovascular nursing/ or critical care nursing/ or developmental disability nursing/ or emergency nursing/ or geriatric nursing/ or holistic nursing/ or home nursing/ or "hospice and palliative care nursing"/ or maternal-child nursing/ or medical-surgical nursing/ or nephrology nursing/ or neuroscience nursing/ or nursing, practical/ or obstetric nursing/ or occupational health nursing/ or oncology nursing/ or orthopedic nursing/ or pediatric nursing/ or perioperative nursing/ or primary nursing/ or psychiatric nursing/ or rehabilitation nursing/ or trauma nursing/	128336
4	primary care nursing/	554
5	specialties, nursing/ or advanced practice nursing/ or cardiovascular nursing/ or community health nursing/ or home health nursing/ or parish nursing/ or critical care nursing/ or developmental disability nursing/ or emergency nursing/ or family nursing/ or forensic nursing/ or geriatric nursing/ or holistic nursing/ or "hospice and palliative care nursing"/ or maternal-child nursing/ or neonatal nursing/ or medical-surgical nursing/ or midwifery/ or military nursing/ or nephrology nursing/ or neuroscience nursing/ or obstetric nursing/ or occupational health nursing/ or oncology nursing/ or orthopedic nursing/ or pediatric nursing/ or perioperative nursing/ or operating room nursing/ or postanesthesia nursing/ or psychiatric nursing/ or public health nursing/ or "radiologic and imaging nursing"/ or rehabilitation nursing/ or rural nursing/ or school nursing/ or transcultural nursing/ or trauma nursing/	163082
6	Nursing, Practical/	3442
7	Nurse's Role/	42459
8	nursing/ or nursing, private duty/ or nursing, supervisory/ or office nursing/ or telenursing/ or travel nursing/	60725
9	nursing process/ or nursing assessment/ or nursing diagnosis/	38420
10	nursing services/ or home care services/ or home health nursing/ or nursing service, hospital/	51509
11	Nursing, Team/	2593
12	Nursing Faculty Practice/	615
13	(nurse or nurses or nursing).tw,kf.	449818
14	or/1-13 [Nurses]	663784
15	(predictive adj2 analy*).tw,kf.	4584
16	predictive model*.tw,kf.	21075
17	predictive monitoring.tw,kf.	47
18	predictive technolog*.tw,kf.	31
19	real time analytic*.tw,kf.	91

20	probability based.tw,kf.	1237
21	predictive algorithm*.tw,kf.	850
22	risk assessment software tool?.tw,kf.	3
23	(command center? or command centre?).tw,kf.	172
24	Risk Assessment/	300351
25	software/	120871
26	24 and 25	994
27	(risk assess* or risks assess* or assess* risk or assess* risks or risk analysis or risks analysis or risk analyses or risks analyses).tw,kf.	91886
28	(software or computer program*).tw,kf.	176594
29	27 and 28	1499
30	exp Machine Learning/	46345
31	(machine learning or transfer learning or learning machine or learning machines).tw,kf.	45740
32	Bayes Theorem/	43738
33	(bayes decision procedure or bayes theorem or bayesian analysis or bayesian approach* or bayesian decision* or bayesian estimation* or bayesian forecast* or byesian modeling or bayesian method* or bayesian prediction* or naive bayesian classifier).tw,kf.	13037
34	Deep Learning/	11650
35	(deep learning or hierarchical learning).tw,kf.	20224
36	clinical decision rules/	870
37	(clinical decision rule or clinical decision rules or clinical prediction rule or clinical prediction rules).tw,kf.	2077
38	(electronic prediction rule or electronic prediction rules).tw,kf.	3
39	early warning score/	318
40	(early warning score or early warning scores or early warning scoring or mew score* or "modified early warning (MEW) score*" or early warning sign score* or "early warning (PEW) score*").tw,kf.	1250
41	early warning system*.tw,kf.	2008
42	decision support techniques/	22242
43	(decision analyses or decision analysis or decision modeling or decision support model or decision support models or decision support technic or decision support technics or decision support technique or decision support techniques).tw,kf.	5962
44	Pattern Recognition, Automated/	26251
45	(automat* adj2 pattern* adj2 analy*).tw,kf.	51
46	(automat* adj2 pattern* adj2 recog*).tw,kf.	133
47	(comput* adj2 pattern?* adj2 analy*).tw,kf.	68
48	(comput* adj2 pattern* adj2 recog*).tw,kf.	147
49	decision making, computer-assisted/ or diagnosis, computer-assisted/ or therapy, computer-assisted/ or drug therapy, computer-assisted/	34378
50	(automatic diagnosis or computer assisted decision making or computer assisted decision making medical decision making or computer assisted diagnoses or computer assisted diagnosis or computer assisted protocol directed therapies or computer assisted protocol	1827

	directed therapy or computer assisted therapies or computer assisted therapy or computer diagnosis or decision support technique*).tw,kf.	
51	(comput* adj2 reasoning*).tw,kf.	71
52	(predict* adj algorithm*).tw,kf.	3822
53	or/15-23,26,29-52	226443
54	14 and 53	3248
55	limit 54 to english language	3101
56	limit 55 to yr="2017 -Current"	1113

Database: MEDLINE Epub Ahead of Print and In-Process
Search strategy:

#	Searches	Results
1	(nurse or nurses or nursing).tw,kf.	44862
2	(predictive adj2 analy*).tw,kf.	1023
3	predictive model*.tw,kf.	5605
4	predictive monitoring.tw,kf.	12
5	predictive technolog*.tw,kf.	12
6	real time analytic*.tw,kf.	30
7	probability based.tw,kf.	248
8	predictive algorithm*.tw,kf.	167
9	risk assessment software tool?.tw,kf.	0
10	(command center? or command centre?).tw,kf.	27
11	(risk assess* or risks assess* or assess* risk or assess* risks or risk analysis or risks analysis or risk analyses or risks analyses).tw,kf.	14411
12	(software or computer program*).tw,kf.	46357
13	11 and 12	308
14	(machine learning or transfer learning or learning machine or learning machines).tw,kf.	23752
15	(bayes decision procedure or bayes theorem or bayesian analysis or bayesian approach* or bayesian decision* or bayesian estimation* or bayesian forecast* or byesian modeling or bayesian method* or bayesian prediction* or naive bayesian classifier).tw,kf.	2909
16	(deep learning or hierarchical learning).tw,kf.	12445
17	(clinical decision rule or clinical decision rules or clinical prediction rule or clinical prediction rules).tw,kf.	319
18	(electronic prediction rule or electronic prediction rules).tw,kf.	0
19	(early warning score or early warning scores or early warning scoring or mew score* or "modified early warning (MEW) score*" or early warning sign score* or "early warning (PEW) score*").tw,kf.	303
20	early warning system*.tw,kf.	452

21	(decision analyses or decision analysis or decision modeling or decision support model or decision support models or decision support technic or decision support technics or decision support technique or decision support techniques).tw,kf.	910
22	(automat* adj2 pattern* adj2 analy*).tw,kf.	11
23	(automat* adj2 pattern* adj2 recog*).tw,kf.	26
24	(comput* adj2 pattern?* adj2 analy*).tw,kf.	9
25	(comput* adj2 pattern* adj2 recog*).tw,kf.	23
26	(automatic diagnosis or computer assisted decision making or computer assisted decision making medical decision making or computer assisted diagnoses or computer assisted diagnosis or computer assisted protocol directed therapies or computer assisted protocol directed therapy or computer assisted therapies or computer assisted therapy or computer diagnosis or decision support technique*).tw,kf.	411
27	(comput* adj2 reasoning*).tw,kf.	22
28	(predict* adj algorithm*).tw,kf.	710
29	or/2-10,13-28	43680
30	1 and 29	259
31	limit 30 to english language	259
32	limit 31 to yr="2017 -Current"	217

Database: Embase
Search strategy:

#	Searches	Results
1	nurse/ or expert nurse/ or licensed practical nurse/ or nurse consultant/ or practical nurse/ or registered nurse/ or staff nurse/	153068
2	nursing/ or practical nursing/ or telenursing/ or travel nursing/	236701
3	advanced practice nurse/	1002
4	nurse administrator/ or charge nurse/ or head nurse/ or nurse manager/	15450
5	nurse practitioner/ or acute care nurse practitioner/ or adult nurse practitioner/ or emergency nurse practitioner/ or family nurse practitioner/ or gerontologic nurse practitioner/ or infection control practitioner/ or neonatal nurse practitioner/ or pediatric nurse practitioner/	28531
6	nurse specialist/ or clinical nurse specialist/ or neonatal nurse/ or nurse anesthetist/ or nurse midwife/ or oncology nurse/ or pediatric nurse/	15107
7	nursing staff/	76644
8	nursing care/ or visiting nursing service/	38191
9	nursing discipline/ or acquired immune deficiency syndrome nursing/ or addictions nursing/ or ambulatory care nursing/ or anesthesia nursing/ or burn nursing/ or camp nursing/ or cardiovascular nursing/ or dermatology nursing/ or emergency nursing/ or enterostomal therapy nursing/ or family nursing/ or flight nursing/ or forensic nursing/ or gastroenterology nursing/ or genetics nursing/ or gynecologic nursing/ or hospice	46145

	nursing/ or intravenous nursing/ or learning disability nursing/ or military nursing/ or nephrology nursing/ or neuroscience nursing/ or nurse midwifery/ or obstetrical nursing/ or occupational health nursing/ or ophthalmic nursing/ or orthopedic nursing/ or palliative nursing/ or parish nursing/ or perinatal nursing/ or perioperative nursing/ or postanesthesia nursing/ or prison nursing/ or radiology nursing/ or rehabilitation nursing/ or respiratory nursing/ or rural health nursing/ or school health nursing/ or urologic nursing/	
10	community health nursing/ or community psychiatric nursing/	26978
11	community health nursing/ or community psychiatric nursing/	26978
12	geriatric nursing/ or psychogeriatric nursing/	13093
13	intensive care nursing/ or newborn intensive care nursing/ or pediatric intensive care nursing/	3109
14	newborn nursing/	3912
15	oncology nursing/ or pediatric oncology nursing/	8453
16	pediatric nursing/	13219
17	psychiatric nursing/	16984
18	surgical nursing/ or plastic surgical nursing/	284
19	advanced practice nursing/	2153
20	practical nursing/	159
21	nursing role/	674
22	nursing process/	7092
23	nursing assessment/	27422
24	nursing diagnosis/	4753
25	team nursing/	438
26	(nurse or nurses or nursing).tw,kw.	589125
27	or/1-26	817290
28	(predictive adj2 analy*).tw,kw.	8250
29	predictive model*.tw,kw.	36286
30	predictive monitoring.tw,kw.	70
31	predictive technolog*.tw,kw.	60
32	real time analytic*.tw,kw.	152
33	probability based.tw,kw.	1872
34	predictive algorithm*.tw,kw.	1597
35	risk assessment software tool?.tw,kw.	3
36	(command center? or command centre?).tw,kw.	272
37	risk assessment/	671411
38	software/	99533
39	37 and 38	3308
40	(risk assess* or risks assess* or assess* risk or assess* risks or risk analysis or risks analysis or risk analyses or risks analyses).tw,kw.	141863
41	(software or computer program*).tw,kw.	363619

42	40 and 41	2977
43	exp machine learning/	316784
44	(machine learning or transfer learning or learning machine or learning machines).tw,kw.	80552
45	Bayes theorem/	39899
46	(bayes decision procedure or bayes theorem or bayesian analysis or bayesian approach* or bayesian decision* or bayesian estimation* or bayesian forecast* or byesian modeling or bayesian method* or bayesian prediction* or naive bayesian classifier).tw,kw.	18314
47	deep learning/	25783
48	(deep learning or hierarchical learning).tw,kw.	36855
49	clinical decision rule/	445
50	(clinical decision rule or clinical decision rules or clinical prediction rule or clinical prediction rules).tw,kw.	3410
51	(electronic prediction rule or electronic prediction rules).tw,kw.	3
52	early warning score/ or modified early warning score/	911
53	(early warning score or early warning scores or early warning scoring or mew score* or "modified early warning (MEW) score*" or early warning sign score* or "early warning (PEW) score*").tw,kw.	2441
54	early warning system*.tw,kw.	3038
55	decision support system/	25367
56	(decision analyses or decision analysis or decision modeling or decision support model or decision support models or decision support technic or decision support technics or decision support technique or decision support techniques).tw,kw.	9116
57	automated pattern recognition/	17371
58	(automat* adj2 pattern* adj2 analy*).tw,kw.	72
59	(automat* adj2 pattern* adj2 recog*).tw,kw.	187
60	(comput* adj2 pattern?* adj2 analy*).tw,kw.	91
61	(comput* adj2 pattern* adj2 recog*).tw,kw.	208
62	decision support system/	25367
63	computer assisted diagnosis/	42977
64	computer assisted therapy/ or computer assisted drug therapy/	5693
65	(automatic diagnosis or computer assisted decision making or computer assisted decision making medical decision making or computer assisted diagnoses or computer assisted diagnosis or computer assisted protocol directed therapies or computer assisted protocol directed therapy or computer assisted therapies or computer assisted therapy or computer diagnosis or decision support technique*).tw,kw.	3160
66	(comput* adj2 reasoning*).tw,kw.	106
67	(predict* adj algorithm*).tw,kw.	6520
68	or/28-36,39,42-67	498155
69	27 and 68	5583
70	limit 69 to english language	5401
71	limit 70 to yr="2017 -Current"	2306

72	limit 71 to (books or chapter or conference abstract or conference paper or "conference review")	737
73	71 not 72	1569

Database: Emcare Nursing
Search strategy:

#	Searches	Results
1	nurse/ or expert nurse/ or licensed practical nurse/ or nurse consultant/ or practical nurse/ or registered nurse/ or staff nurse/	146245
2	nursing/ or practical nursing/ or telenursing/ or travel nursing/	99705
3	advanced practice nurse/	2290
4	nurse administrator/ or charge nurse/ or head nurse/ or nurse manager/	8192
5	nurse practitioner/ or acute care nurse practitioner/ or adult nurse practitioner/ or emergency nurse practitioner/ or family nurse practitioner/ or gerontologic nurse practitioner/ or infection control practitioner/ or neonatal nurse practitioner/ or pediatric nurse practitioner/	15585
6	nurse specialist/ or clinical nurse specialist/ or neonatal nurse/ or nurse anesthetist/ or nurse midwife/ or oncology nurse/ or pediatric nurse/	6329
7	nursing staff/	26879
8	nursing care/ or visiting nursing service/	18297
9	nursing discipline/ or acquired immune deficiency syndrome nursing/ or addictions nursing/ or ambulatory care nursing/ or anesthesia nursing/ or burn nursing/ or camp nursing/ or cardiovascular nursing/ or dermatology nursing/ or emergency nursing/ or enterostomal therapy nursing/ or family nursing/ or flight nursing/ or forensic nursing/ or gastroenterology nursing/ or genetics nursing/ or gynecologic nursing/ or hospice nursing/ or intravenous nursing/ or learning disability nursing/ or military nursing/ or nephrology nursing/ or neuroscience nursing/ or nurse midwifery/ or obstetrical nursing/ or occupational health nursing/ or ophthalmic nursing/ or orthopedic nursing/ or palliative nursing/ or parish nursing/ or perinatal nursing/ or perioperative nursing/ or postanesthesia nursing/ or prison nursing/ or radiology nursing/ or rehabilitation nursing/ or respiratory nursing/ or rural health nursing/ or school health nursing/ or urologic nursing/	14984
10	community health nursing/ or community psychiatric nursing/	5303
11	community health nursing/ or community psychiatric nursing/	5303
12	geriatric nursing/ or psychogeriatric nursing/	2381
13	intensive care nursing/ or newborn intensive care nursing/ or pediatric intensive care nursing/	2039
14	newborn nursing/	2250
15	oncology nursing/ or pediatric oncology nursing/	2575
16	pediatric nursing/	2349
17	psychiatric nursing/	4352

18	surgical nursing/ or plastic surgical nursing/	464
19	advanced practice nursing/	1610
20	practical nursing/	190
21	nursing role/	1908
22	nursing process/	1758
23	nursing assessment/	4730
24	nursing diagnosis/	1727
25	team nursing/	183
26	(nurse or nurses or nursing).tw,kw.	296840
27	or/1-26	354493
28	(predictive adj2 analy*).tw,kw.	1994
29	predictive model*.tw,kw.	9538
30	predictive monitoring.tw,kw.	22
31	predictive technolog*.tw,kw.	13
32	real time analytic*.tw,kw.	35
33	probability based.tw,kw.	614
34	predictive algorithm*.tw,kw.	346
35	risk assessment software tool?.tw,kw.	1
36	(command center? or command centre?).tw,kw.	90
37	risk assessment/	193024
38	software/	19888
39	37 and 38	652
40	(risk assess* or risks assess* or assess* risk or assess* risks or risk analysis or risks analysis or risk analyses or risks analyses).tw,kw.	35677
41	(software or computer program*).tw,kw.	84388
42	40 and 41	729
43	exp machine learning/	60354
44	(machine learning or transfer learning or learning machine or learning machines).tw,kw.	22811
45	Bayes theorem/	7146
46	(bayes decision procedure or bayes theorem or bayesian analysis or bayesian approach* or bayesian decision* or bayesian estimation* or bayesian forecast* or byesian modeling or bayesian method* or bayesian prediction* or naive bayesian classifier).tw,kw.	4742
47	deep learning/	5649
48	(deep learning or hierarchical learning).tw,kw.	10366
49	clinical decision rule/	110
50	(clinical decision rule or clinical decision rules or clinical prediction rule or clinical prediction rules).tw,kw.	1508
51	(electronic prediction rule or electronic prediction rules).tw,kw.	2
52	early warning score/ or modified early warning score/	364

53	(early warning score or early warning scores or early warning scoring or mew score* or "modified early warning (MEW) score*" or early warning sign score* or "early warning (PEW) score*").tw,kw.	1043
54	early warning system*.tw,kw.	943
55	decision support system/	10820
56	(decision analyses or decision analysis or decision modeling or decision support model or decision support models or decision support technic or decision support technics or decision support technique or decision support techniques).tw,kw.	3130
57	automated pattern recognition/	341
58	(automat* adj2 pattern* adj2 analy*).tw,kw.	15
59	(automat* adj2 pattern* adj2 recog*).tw,kw.	47
60	(comput* adj2 pattern?* adj2 analy*).tw,kw.	13
61	(comput* adj2 pattern* adj2 recog*).tw,kw.	20
62	decision support system/	10820
63	computer assisted diagnosis/	4364
64	computer assisted therapy/ or computer assisted drug therapy/	1416
65	(automatic diagnosis or computer assisted decision making or computer assisted decision making medical decision making or computer assisted diagnoses or computer assisted diagnosis or computer assisted protocol directed therapies or computer assisted protocol directed therapy or computer assisted therapies or computer assisted therapy or computer diagnosis or decision support technique*).tw,kw.	1202
66	(comput* adj2 reasoning*).tw,kw.	44
67	(predict* adj algorithm*).tw,kw.	1400
68	or/28-36,39,42-67	110131
69	27 and 68	2739
70	limit 69 to english language	2635
71	limit 70 to yr="2017 -Current"	1100
72	limit 71 to (books or chapter or conference abstract or conference paper or "conference review")	35
73	71 not 72	1065

Database: Cochrane Central Register of Controlled Trials
Search strategy:

#	Searches	Results
1	nurses/ or nurse administrators/ or nurse practitioners/ or family nurse practitioners/ or pediatric nurse practitioners/ or nurse specialists/ or nurse anesthetists/ or nurse clinicians/ or nurse midwives/ or nurses, pediatric/ or nurses, neonatal/ or nurses, community health/ or nurses, public health/	1323
2	nursing staff/ or nursing staff, hospital/	682

3	nursing care/ or cardiovascular nursing/ or critical care nursing/ or developmental disability nursing/ or emergency nursing/ or geriatric nursing/ or holistic nursing/ or home nursing/ or "hospice and palliative care nursing"/ or maternal-child nursing/ or medical-surgical nursing/ or nephrology nursing/ or neuroscience nursing/ or nursing, practical/ or obstetric nursing/ or occupational health nursing/ or oncology nursing/ or orthopedic nursing/ or pediatric nursing/ or perioperative nursing/ or primary nursing/ or psychiatric nursing/ or rehabilitation nursing/ or trauma nursing/	1642
4	primary care nursing/	35
5	specialties, nursing/ or advanced practice nursing/ or cardiovascular nursing/ or community health nursing/ or home health nursing/ or parish nursing/ or critical care nursing/ or developmental disability nursing/ or emergency nursing/ or family nursing/ or forensic nursing/ or geriatric nursing/ or holistic nursing/ or "hospice and palliative care nursing"/ or maternal-child nursing/ or neonatal nursing/ or medical-surgical nursing/ or midwifery/ or military nursing/ or nephrology nursing/ or neuroscience nursing/ or obstetric nursing/ or occupational health nursing/ or oncology nursing/ or orthopedic nursing/ or pediatric nursing/ or perioperative nursing/ or operating room nursing/ or postanesthesia nursing/ or psychiatric nursing/ or public health nursing/ or "radiologic and imaging nursing"/ or rehabilitation nursing/ or rural nursing/ or school nursing/ or transcultural nursing/ or trauma nursing/	2241
6	Nursing, Practical/	10
7	Nurse's Role/	386
8	nursing/ or nursing, private duty/ or nursing, supervisory/ or office nursing/ or telenursing/ or travel nursing/	123
9	nursing process/ or nursing assessment/ or nursing diagnosis/	568
10	nursing services/ or home care services/ or home health nursing/ or nursing service, hospital/	1968
11	Nursing, Team/	22
12	Nursing Faculty Practice/	4
13	(nurse or nurses or nursing).tw,kw.	44769
14	or/1-13 [Nurses]	47591
15	(predictive adj2 analy*).tw,kw.	627
16	predictive model*.tw,kw.	1257
17	predictive monitoring.tw,kw.	1
18	predictive technolog*.tw,kw.	0
19	real time analytic*.tw,kw.	3
20	probability based.tw,kw.	56
21	predictive algorithm*.tw,kw.	101
22	risk assessment software tool?.tw,kw.	0
23	(command center? or command centre?).tw,kw.	1
24	Risk Assessment/	9603
25	software/	1050
26	24 and 25	17

27	(risk assess* or risks assess* or assess* risk or assess* risks or risk analysis or risks analysis or risk analyses or risks analyses).tw,kw.	20953
28	(software or computer program*).tw,kw.	29311
29	27 and 28	430
30	exp Machine Learning/	252
31	(machine learning or transfer learning or learning machine or learning machines).tw,kw.	2050
32	Bayes Theorem/	591
33	(bayes decision procedure or bayes theorem or bayesian analysis or bayesian approach* or bayesian decision* or bayesian estimation* or bayesian forecast* or byesian modeling or bayesian method* or bayesian prediction* or naive bayesian classifier).tw,kw.	1108
34	Deep Learning/	54
35	(deep learning or hierarchical learning).tw,kw.	762
36	clinical decision rules/	20
37	(clinical decision rule or clinical decision rules or clinical prediction rule or clinical prediction rules).tw,kw.	253
38	(electronic prediction rule or electronic prediction rules).tw,kw.	1
39	early warning score/	5
40	(early warning score or early warning scores or early warning scoring or mew score* or "modified early warning (MEW) score*" or early warning sign score* or "early warning (PEW) score*").tw,kw.	193
41	early warning system*.tw,kw.	70
42	decision support techniques/	903
43	(decision analyses or decision analysis or decision modeling or decision support model or decision support models or decision support technic or decision support technics or decision support technique or decision support techniques).tw,kw.	373
44	Pattern Recognition, Automated/	221
45	(automat* adj2 pattern* adj2 analy*).tw,kw.	1
46	(automat* adj2 pattern* adj2 recog*).tw,kw.	16
47	(comput* adj2 pattern?* adj2 analy*).tw,kw.	2
48	(comput* adj2 pattern* adj2 recog*).tw,kw.	7
49	decision making, computer-assisted/ or diagnosis, computer-assisted/ or therapy, computer-assisted/ or drug therapy, computer-assisted/	2350
50	(automatic diagnosis or computer assisted decision making or computer assisted decision making medical decision making or computer assisted diagnoses or computer assisted diagnosis or computer assisted protocol directed therapies or computer assisted protocol directed therapy or computer assisted therapies or computer assisted therapy or computer diagnosis or decision support technique*).tw,kw.	484
51	(comput* adj2 reasoning*).tw,kw.	8
52	(predict* adj algorithm*).tw,kw.	223
53	or/15-23,26,29-52	10983
54	14 and 53	344
55	limit 54 to english language	333

56	limit 55 to yr="2017 -Current"	153

Database: Cochrane Database of Systematic Reviews
Search strategy:

#	Searches	Results
1	(nurse or nurses or nursing).tw,kw.	3053
2	(predictive adj2 analy*).tw,kw.	8
3	predictive model*.tw,kw.	20
4	predictive monitoring.tw,kw.	0
5	predictive technolog*.tw,kw.	0
6	real time analytic*.tw,kw.	0
7	probability based.tw,kw.	4
8	predictive algorithm*.tw,kw.	1
9	risk assessment software tool?.tw,kw.	0
10	(command center? or command centre?).tw,kw.	1
11	(risk assess* or risks assess* or assess* risk or assess* risks or risk analysis or risks analysis or risk analyses or risks analyses).tw,kw.	4587
12	(software or computer program*).tw,kw.	7299
13	11 and 12	3713
14	(machine learning or transfer learning or learning machine or learning machines).tw,kw.	112
15	(bayes decision procedure or bayes theorem or bayesian analysis or bayesian approach* or bayesian decision* or bayesian estimation* or bayesian forecast* or byesian modeling or bayesian method* or bayesian prediction* or naive bayesian classifier).tw,kw.	69
16	(deep learning or hierarchical learning).tw,kw.	4
17	(clinical decision rule or clinical decision rules or clinical prediction rule or clinical prediction rules).tw,kw.	22
18	(electronic prediction rule or electronic prediction rules).tw,kw.	0
19	(early warning score or early warning scores or early warning scoring or mew score* or "modified early warning (MEW) score*" or early warning sign score* or "early warning (PEW) score*").tw,kw.	4
20	early warning system*.tw,kw.	9
21	(decision analyses or decision analysis or decision modeling or decision support model or decision support models or decision support technic or decision support technics or decision support technique or decision support techniques).tw,kw.	75
22	(automat* adj2 pattern* adj2 analy*).tw,kw.	0
23	(automat* adj2 pattern* adj2 recog*).tw,kw.	1
24	(comput* adj2 pattern?* adj2 analy*).tw,kw.	0
25	(comput* adj2 pattern* adj2 recog*).tw,kw.	0

26	(automatic diagnosis or computer assisted decision making or computer assisted decision making medical decision making or computer assisted diagnoses or computer assisted diagnosis or computer assisted protocol directed therapies or computer assisted protocol directed therapy or computer assisted therapies or computer assisted therapy or computer diagnosis or decision support technique*).tw,kw.	66
27	(comput* adj2 reasoning*).tw,kw.	1
28	(predict* adj algorithm*).tw,kw.	2
29	or/2-10,13-28	3869
30	1 and 29	1474
31	limit 30 to last 5 years	581
32	limit 31 to full systematic reviews	515

Database: APA PsychInfo
Search strategy:

#	Searches	Results
1	nurses/ or psychiatric nurses/ or public health service nurses/	34688
2	nursing/	25277
3	(nurse or nurses or nursing).tw,id.	108952
4	or/1-3	109510
5	(predictive adj2 analy*).tw,id.	1109
6	predictive model*.tw,id.	3773
7	predictive monitoring.tw,id.	6
8	predictive technolog*.tw,id.	9
9	real time analytic*.tw,id.	13
10	probability based.id.	33
11	predictive algorithm*.tw,id.	97
12	risk assessment software tool?.tw,id.	0
13	(command center? or command centre?).tw,id.	51
14	risk assessment/	14926
15	computer software/	10684
16	14 and 15	28
17	(risk assess* or risks assess* or assess* risk or assess* risks or risk analysis or risks analysis or risk analyses or risks analyses).tw,id.	16448
18	(software or computer program*).tw,id.	35534
19	17 and 18	153
20	exp machine learning/	12646
21	(machine learning or transfer learning or learning machine or learning machines).tw,id.	11014
22	bayesian analysis/	1148

23	(bayes decision procedure or bayes theorem or bayesian analysis or bayesian approach* or bayesian decision* or bayesian estimation* or bayesian forecast* or byesian modeling or bayesian method* or bayesian prediction* or naive bayesian classifier).tw,id.	3714
24	(deep learning or hierarchical learning).tw,id.	2746
25	(clinical decision rule or clinical decision rules or clinical prediction rule or clinical prediction rules).tw,id.	132
26	(electronic prediction rule or electronic prediction rules).tw,id.	0
27	(early warning score or early warning scores or early warning scoring or mew score* or "modified early warning (MEW) score*" or early warning sign score* or "early warning (PEW) score*").tw,id.	68
28	early warning system*.tw,id.	298
29	decision support systems/	3580
30	(decision analyses or decision analysis or decision modeling or decision support model or decision support models or decision support technic or decision support technics or decision support technique or decision support techniques).tw,id.	1286
31	"pattern recognition (computer science)"/	884
32	(automat* adj2 pattern* adj2 analy*).tw,id.	5
33	(automat* adj2 pattern* adj2 recog*).tw,id.	26
34	(comput* adj2 pattern?* adj2 analy*).tw,id.	20
35	(comput* adj2 pattern* adj2 recog*).tw,id.	51
36	computer assisted therapy/	1188
37	(automatic diagnosis or computer assisted decision making or computer assisted decision making medical decision making or computer assisted diagnoses or computer assisted diagnosis or computer assisted protocol directed therapies or computer assisted protocol directed therapy or computer assisted therapies or computer assisted therapy or computer diagnosis or decision support technique*).tw,id.	328
38	(comput* adj2 reasoning*).tw,id.	140
39	(predict* adj algorithm*).tw,id.	304
40	or/5-13,16,19-39	33457
41	4 and 40	417
42	limit 41 to english language	392
43	limit 42 to yr="2017 -Current"	156

Database: Cumulative Index to Nursing and Allied Health (CINAHL)
Search strategy:

#	Query	Limiters/Expanders	Results
S1	(MH "Nursing Manpower+")	Expanders - Apply equivalent subjects<br>Search modes - Boolean/Phrase	267,228

S2	(MH "Nursing Role")	Expanders - Apply equivalent subjects<br>Search modes - Boolean/Phrase	63,180
S3	(MH "Telenursing")	Expanders - Apply equivalent subjects<br>Search modes - Boolean/Phrase	2,281
S4	(MH "Nursing Process")	Expanders - Apply equivalent subjects<br>Search modes - Boolean/Phrase	3,687
S5	(MH "Nursing Assessment")	Expanders - Apply equivalent subjects<br>Search modes - Boolean/Phrase	18,468
S6	(MH "Nursing Diagnosis")	Expanders - Apply equivalent subjects<br>Search modes - Boolean/Phrase	4,751
S7	(MH "Nursing Service")	Expanders - Apply equivalent subjects<br>Search modes - Boolean/Phrase	1,514
S8	(MH "Team Nursing") OR (MH "Nursing Care Delivery Systems") OR (MH "Differentiated Nursing Practice") OR (MH "Functional Nursing") OR (MH "Modular Nursing") OR (MH "Primary Nursing") OR (MH "Total Patient Care Nursing") OR (MH "Nursing Care")	Expanders - Apply equivalent subjects<br>Search modes - Boolean/Phrase	29,599
S9	TI (nurse or nurses or nursing) OR AB (nurse or nurses or nursing)	Expanders - Apply equivalent subjects<br>Search modes - Boolean/Phrase	601,615
S10	S1 OR S2 OR S3 OR S4 OR S5 OR S6 OR S7 OR S8 OR S9	Expanders - Apply equivalent subjects<br>Search modes - Boolean/Phrase	738,719
S11	TI (predictive N1 analy*) OR AB (predictive N1 analy*)	Expanders - Apply equivalent subjects<br>Search modes - Boolean/Phrase	1,562
S12	TI (predictive model*) OR AB (predictive model*)	Expanders - Apply equivalent subjects<br>Search modes - Boolean/Phrase	5,864
S13	TI (predictive monitoring) OR AB (predictive monitoring)	Expanders - Apply equivalent subjects<br>Search modes - Boolean/Phrase	11

S14	TI (predictive technolog*) OR AB (predictive technolog*)	Expanders - Apply equivalent subjects<br>Search modes - Boolean/Phrase	11
S15	TI (real time analytic*) OR AB (real time analytic*)	Expanders - Apply equivalent subjects<br>Search modes - Boolean/Phrase	29
S16	TI (probability based) OR AB (probability based)	Expanders - Apply equivalent subjects<br>Search modes - Boolean/Phrase	441
S17	TI (predictive algorithm*) OR AB (predictive algorithm*)	Expanders - Apply equivalent subjects<br>Search modes - Boolean/Phrase	248
S18	TI (risk assessment software tool#) OR AB (risk assessment software tool#)	Expanders - Apply equivalent subjects<br>Search modes - Boolean/Phrase	1
S19	TI (command center# or command centre#) OR AB (command center# or command centre#)	Expanders - Apply equivalent subjects<br>Search modes - Boolean/Phrase	105
S20	(MH "Risk Assessment")	Expanders - Apply equivalent subjects<br>Search modes - Boolean/Phrase	132,192
S21	(MH "Software")	Expanders - Apply equivalent subjects<br>Search modes - Boolean/Phrase	31,466
S22	S20 AND S21	Expanders - Apply equivalent subjects<br>Search modes - Boolean/Phrase	385
S23	TI (risk assess* or risks assess* or assess* risk or assess* risks or risk analysis or risks analysis or risk analyses or risks analyses) OR AB (risk assess* or risks assess* or assess* risk or assess* risks or risk analysis or risks analysis or risk analyses or risks analyses)	Expanders - Apply equivalent subjects<br>Search modes - Boolean/Phrase	25,492
S24	TI (software or computer program*) OR AB (software or computer program*)	Expanders - Apply equivalent subjects<br>Search modes - Boolean/Phrase	58,247
S25	S23 AND S24	Expanders - Apply equivalent subjects<br>Search modes - Boolean/Phrase	904

S26	(MH "Machine Learning+")	Expanders - Apply equivalent subjects<br>Search modes - Boolean/Phrase	4,214
S27	TI (machine learning or transfer learning or learning machine or learning machines) OR AB (machine learning or transfer learning or learning machine or learning machines)	Expanders - Apply equivalent subjects<br>Search modes - Boolean/Phrase	10,459
S28	TI (bayes decision procedure or bayes theorem or bayesian analysis or bayesian approach* or bayesian decision* or bayesian estimation* or bayesian forecast* or byesian modeling or bayesian method* or bayesian prediction* or naive bayesian classifier) OR AB (bayes decision procedure or bayes theorem or bayesian analysis or bayesian approach* or bayesian decision* or bayesian estimation* or bayesian forecast* or byesian modeling or bayesian method* or bayesian prediction* or naive bayesian classifier)	Expanders - Apply equivalent subjects<br>Search modes - Boolean/Phrase	2,148
S29	(MH "Deep Learning")	Expanders - Apply equivalent subjects<br>Search modes - Boolean/Phrase	1,069
S30	TI (deep learning or hierarchical learning). OR AB (deep learning or hierarchical learning)	Expanders - Apply equivalent subjects<br>Search modes - Boolean/Phrase	3,752
S31	(MH "Clinical Prediction Rules")	Expanders - Apply equivalent subjects<br>Search modes - Boolean/Phrase	31
S32	TI (clinical decision rule or clinical decision rules or clinical prediction rule or clinical prediction rules) OR AB (clinical decision rule or clinical decision rules or clinical prediction rule or clinical prediction rules)	Expanders - Apply equivalent subjects<br>Search modes - Boolean/Phrase	1,278
S33	TI (electronic prediction rule or electronic prediction rules) OR AB (electronic prediction rule or electronic prediction rules)	Expanders - Apply equivalent subjects<br>Search modes - Boolean/Phrase	1
S34	(MH "Early Warning Score")	Expanders - Apply equivalent subjects<br>Search modes - Boolean/Phrase	107
S35	TI (early warning score or early warning scores or early warning scoring or mew score* or "modified early warning (MEW) score*" or early warning sign score* or "early warning (PEW) score*" or early	Expanders - Apply equivalent subjects<br>Search modes - Boolean/Phrase	1,601

	warning system*) OR AB (early warning score or early warning scores or early warning scoring or mew score* or "modified early warning (MEW) score*" or early warning sign score* or "early warning (PEW) score*" or early warning system*)		
S36	(MH "Decision Support Techniques")	Expanders - Apply equivalent subjects<br>Search modes - Boolean/Phrase	7,572
S37	TI (decision analyses or decision analysis or decision modeling or decision support model or decision support models or decision support technic or decision support technics or decision support technique or decision support techniques) OR AB (decision analyses or decision analysis or decision modeling or decision support model or decision support models or decision support technic or decision support technics or decision support technique or decision support techniques)	Expanders - Apply equivalent subjects<br>Search modes - Boolean/Phrase	1,840
S38	TI (automat* N1 pattern* N1 analy*) OR AB (automat* N1 pattern* N1 analy*)	Expanders - Apply equivalent subjects<br>Search modes - Boolean/Phrase	13
S39	TI (automat* N1 pattern* N1 recog*) OR AB (automat* N1 pattern* N1 recog*)	Expanders - Apply equivalent subjects<br>Search modes - Boolean/Phrase	14
S40	TI (comput* N1 pattern* N1 analy*) OR AB (comput* N1 pattern* N1 analy*)	Expanders - Apply equivalent subjects<br>Search modes - Boolean/Phrase	7
S41	TI (comput* N1 pattern* N1 recog*) OR AB (comput* N1 pattern* N1 recog*)	Expanders - Apply equivalent subjects<br>Search modes - Boolean/Phrase	10
S42	(MH "Decision Making, Computer Assisted") OR (MH "Diagnosis, Computer Assisted") OR (MH "Therapy, Computer Assisted") OR (MH "Drug Therapy, Computer Assisted")	Expanders - Apply equivalent subjects<br>Search modes - Boolean/Phrase	11,918
S43	TI (automatic diagnosis or computer assisted decision making or computer assisted decision making medical decision making or computer assisted diagnoses or computer assisted diagnosis or computer assisted protocol directed therapies or computer assisted protocol directed therapy or computer assisted therapies or computer assisted therapy or computer diagnosis or decision support	Expanders - Apply equivalent subjects<br>Search modes - Boolean/Phrase	219

	technique*) OR AB (automatic diagnosis or computer assisted decision making or computer assisted decision making medical decision making or computer assisted diagnoses or computer assisted diagnosis or computer assisted protocol directed therapies or computer assisted protocol directed therapy or computer assisted therapies or computer assisted therapy or computer diagnosis or decision support technique*)		
S44	TI (comput* N1 reasoning*) OR AB (comput* N1 reasoning*)	Expanders - Apply equivalent subjects<br>Search modes - Boolean/Phrase	20
S45	TI (predict* N1 algorithm) OR AB (predict* N1 algorithm)	Expanders - Apply equivalent subjects<br>Search modes - Boolean/Phrase	1,585
S46	S11 OR S12 OR S13 OR S14 OR S15 OR S16 OR S17 OR S18 OR S19 OR S22 OR S25 OR S26 OR S27 OR S28 OR S29 OR S30 OR S31 OR S32 OR S33 OR S34 OR S35 OR S36 OR S37 OR S38 OR S39 OR S40 OR S41 OR S42 OR S43 OR S44 OR S45	Expanders - Apply equivalent subjects<br>Search modes - Boolean/Phrase	48,385
S47	S10 AND S46	Expanders - Apply equivalent subjects<br>Search modes - Boolean/Phrase	2,104
S48	S10 AND S46	Limiters - English Language<br>Expanders - Apply equivalent subjects<br>Search modes - Boolean/Phrase	2,026
S49	S10 AND S46	Limiters - Published Date: 20170101-20221231; English Language<br>Expanders - Apply equivalent subjects<br>Search modes - Boolean/Phrase	822
S50	S10 AND S46	Limiters - Published Date: 20170101-20221231; English Language; Peer Reviewed<br>Expanders - Apply equivalent subjects<br>Search modes - Boolean/Phrase	753

Database: IEEE Explore

Search strategy:

Note: METADATA - Includes the abstract, index terms, and bibliographic citation data (such as document title, publication title, author, etc.).

OR following search lines:

predict* NEAR/1 analy* OR automat* NEAR/2 pattern NEAR/2 analy* OR automat* NEAR/2 pattern NEAR/2 recog*

comput* NEAR/2 pattern NEAR/2 analy* OR comput* NEAR/2 pattern NEAR/2 recog* OR comput* NEAR/2 pattern NEAR/2 recog*

comput* NEAR/2 reasoning* OR predict* NEAR/1 algorithm*

predictive model* OR predictive monitoring OR predictive technolog* OR real time analytic* OR probability based OR risk assessment software tool OR command center OR command centre

predictive model* OR predictive monitoring OR predictive technolog* OR real time analytic* OR probability based OR predictive algorithm OR risk assessment software tool

bayes decision procedure OR bayes theorem OR bayesian analysis OR bayesian approach* OR bayesian decision OR bayesian estimation OR bayesian forecast OR byesian modeling OR bayesian method* OR bayesian prediction OR naive bayesian classifier

machine learning OR transfer learning OR learning machine OR deep learning OR hierarchical learning OR clinical decision rule OR clinical prediction rule OR electronic prediction rule

early warning score OR early warning scoring OR mew score OR "modified early warning (MEW) score" OR "modified early warning (MEW) scores" OR early warning sign score OR "early warning (PEW) score*" OR "early warning (PEW) scores" OR early warning system*

decision analyses OR decision analysis OR decision modeling OR decision support model OR decision support models OR decision support technic OR decision support technique

automatic diagnosis OR computer assisted decision making OR computer assisted decision making medical decision making OR computer assisted diagnoses OR computer assisted diagnosis OR computer assisted protocol directed therapies OR computer assisted protocol directed therapy OR computer assisted therapies OR computer assisted therapy OR computer diagnosis OR decision support technique

On Results Screen - Search within results

nurse OR nurses OR nursing

Limit results to Journals

Screen Image of complete search with limits

("All Metadata":predict* NEAR/1 analy* OR "All Metadata":automat* NEAR/2 pattern NEAR/2 analy* OR "All Metadata":automat* NEAR/2 pattern NEAR/2 recog*) OR ("All Metadata":comput* NEAR/2 pattern NEAR/2 analy* OR "All Metadata":comput* NEAR/2 pattern NEAR/2 recog* OR "All Metadata":comput* NEAR/2 pattern NEAR/2 recog*) OR ("All Metadata":comput* NEAR/2 reasoning* OR "All Metadata":predict* NEAR/1 algorithm*) OR ("All Metadata":predictive model* OR "All Metadata":predictive monitoring OR "All Metadata":predictive technolog* OR "All Metadata":real time analytic* OR "All Metadata":probability based OR "All Metadata":risk assessment software tool OR "All Metadata":command center OR "All Metadata":command centre) OR ("All Metadata":predictive model* OR "All Metadata":predictive monitoring OR "All Metadata":predictive technolog* OR "All Metadata":real time analytic* OR "All Metadata":probability based OR "All Metadata":predictive algorithm OR "All Metadata":risk assessment software tool) OR ("All Metadata":bayes decision procedure OR "All Metadata":bayes theorem OR "All Metadata":bayesian analysis OR "All Metadata":bayesian approach* OR "All Metadata":bayesian decision OR "All Metadata":bayesian estimation OR "All Metadata":bayesian forecast OR "All Metadata":byesian modeling OR "All Metadata":bayesian method* OR "All Metadata":bayesian prediction OR "All Metadata":naive bayesian classifier) OR ("All Metadata":machine learning OR "All Metadata":transfer learning OR "All Metadata":learning machine OR "All Metadata":deep learning OR "All Metadata":hierarchical learning OR "All Metadata":clinical decision rule OR "All Metadata":clinical prediction rule OR "All Metadata":electronic prediction rule) OR ("All Metadata":early warning score OR "All Metadata":early warning scoring OR "All Metadata":mew score OR "All Metadata":"modified early warning (MEW) score" OR "All Metadata":"modified early warning (MEW) scores" OR "All Metadata":early warning sign score OR "All Metadata":"early warning (PEW) score*" OR "All Metadata":"early warning (PEW) scores" OR "All Metadata":early warning system*) OR ("All Metadata":decision analyses OR "All Metadata":decision analysis OR "All Metadata":decision modeling OR "All Metadata":decision support model OR "All Metadata":decision support models OR "All Metadata":decision support technic OR "All Metadata":decision support technique) OR ("All Metadata":automatic diagnosis OR "All Metadata":computer assisted decision making OR "All Metadata":computer assisted decision making medical decision making OR "All Metadata":computer assisted diagnoses OR "All Metadata":computer assisted diagnosis OR "All Metadata":computer assisted protocol directed therapies OR "All Metadata":computer assisted protocol directed therapy OR "All Metadata":computer assisted therapies OR "All Metadata":computer assisted therapy OR "All Metadata":computer diagnosis OR "All Metadata":decision support technique) ×
nurse OR nurses OR nursing ×

Filters Applied: Journals × 2017 - 2022 ×

Indirect Search for Recommendation Question #4: Should leveraging predictive analytics (e.g., command centers and risk assessment software tools) for nurses providing care in all practice settings be recommended to inform clinical decision-making and improve clinical outcomes?

Dates searched: January 1, 2017 – January 4 and 5, 2023

Databases searched: MEDLINE, Embase and CINAHL

**Note: The original search strategy was broadened to examine all health providers (not just nurses). The search was only applied to MEDLINE, Embase, and CINAHL for feasibility purposes, and limited to systematic reviews in order to obtain the highest quality evidence.*

Database: Medline
Search strategy:

#	Searches	Results
1	exp "Attitude of Health Personnel"/	168744
2	exp Health Personnel/	599121
3	exp Personnel, Hospital/	94889
4	exp Health Occupations/	1834137

5	exp Patient Care Team/	72489
6	Social Work/	16173
7	Social Workers/	1037
8	Nurse's Role/	42651
9	Licensed Practical Nurses/	97
10	exp Nurses/	96894
11	exp Nursing Staff/	69492
12	Nursing Staff, Hospital/	47703
13	exp Nursing/	262340
14	Nursing, Practical/	3444
15	Health Educators/	489
16	exp Administrative Personnel/	42128
17	(health* adj2 provider?).tw,kf.	72423
18	(hospital* adj2 provider?).tw,kf.	1575
19	(health* adj2 staff?).tw,kf.	8420
20	(hospital* adj2 staff?).tw,kf.	8102
21	(health* adj2 personnel?).tw,kf.	13917
22	(hospital* adj2 personnel?).tw,kf.	2881
23	(health* adj2 employe*).tw,kf.	6474
24	(hospital* adj2 employe*).tw,kf.	2464
25	(health* adj2 profession*).tw,kf.	116500
26	(hospital* adj2 profession*).tw,kf.	1375
27	(health* adj2 occupation?).tw,kf.	1180
28	(hospital* adj2 occupation?).tw,kf.	99
29	(health* adj2 practitioner?).tw,kf.	11259
30	(hospital* adj2 practitioner?).tw,kf.	1180
31	(health* adj2 worker?).tw,kf.	55115
32	(hospital* adj2 worker?).tw,kf.	2109
33	nurs*.tw,kf.	471467
34	(patient care? adj2 team?).tw,kf.	840
35	(health* adj2 educator?).tw,kf.	3312

36	(social adj2 worker?).tw,kf.	11030
37	(support* adj2 (worker? or staff?)).tw,kf.	6756
38	clinician?.tw,kf.	238228
39	doctor?.tw,kf.	123332
40	physician?.tw,kf.	394438
41	practitioner?.tw,kf.	152305
42	surgeon?.tw,kf.	203846
43	Caregivers/	48189
44	(caregiver? or care-giver? or carer or carers).tw,kf.	88098
45	or/1-44 [Health Care Personnel]	3362729
46	(predictive adj2 analy*).tw,kf.	4877
47	predictive model*.tw,kf.	22597
48	predictive monitoring.tw,kf.	47
49	predictive technolog*.tw,kf.	32
50	real time analytic*.tw,kf.	95
51	probability based.tw,kf.	1285
52	predictive algorithm*.tw,kf.	902
53	risk assessment software tool?.tw,kf.	3
54	(command center? or command centre?).tw,kf.	184
55	Risk Assessment/	303089
56	software/	122652
57	55 and 56	995
58	(risk assess* or risks assess* or assess* risk or assess* risks or risk analysis or risks analysis or risk analyses or risks analyses).tw,kf.	96097
59	(software or computer program*).tw,kf.	184178
60	58 and 59	1586
61	Artificial Intelligence/	35414
62	(artificial intelligence or computational intelligence or computer reasoning or computer vision system or computer vision systems or machine intelligence).tw,kf.	19387
63	exp Machine Learning/	52784
64	(machine learning or transfer learning or learning machine or learning machines).tw,kf.	53196

65	Bayes Theorem/	46225
66	(bayes decision procedure or bayes theorem or bayesian analysis or bayesian approach* or bayesian decision* or bayesian estimation* or bayesian forecast* or byesian modeling or bayesian method* or bayesian prediction* or naive bayesian classifier).tw,kf.	13476
67	Deep Learning/	13892
68	(deep learning or hierarchical learning).tw,kf.	25322
69	clinical decision rules/	889
70	(clinical decision rule or clinical decision rules or clinical prediction rule or clinical prediction rules).tw,kf.	2142
71	(electronic prediction rule or electronic prediction rules).tw,kf.	3
72	early warning score/	350
73	(early warning score or early warning scores or early warning scoring or mew score* or "modified early warning (MEW) score*" or early warning sign score* or "early warning (PEW) score*").tw,kf.	1345
74	early warning system*.tw,kf.	2124
75	decision support techniques/	22351
76	(decision analyses or decision analysis or decision modeling or decision support model or decision support models or decision support technic or decision support technics or decision support technique or decision support techniques).tw,kf.	6111
77	Pattern Recognition, Automated/	26366
78	(automat* adj2 pattern* adj2 analy*).tw,kf.	50
79	(automat* adj2 pattern* adj2 recog*).tw,kf.	136
80	(comput* adj2 pattern?* adj2 analy*).tw,kf.	71
81	(comput* adj2 pattern* adj2 recog*).tw,kf.	149
82	decision making, computer-assisted/ or diagnosis, computer-assisted/ or therapy, computer-assisted/ or drug therapy, computer-assisted/	34503
83	(automatic diagnosis or computer assisted decision making or computer assisted decision making medical decision making or computer assisted diagnoses or computer assisted diagnosis or computer assisted protocol directed therapies or computer assisted protocol directed therapy or computer assisted therapies or computer assisted therapy or computer diagnosis or decision support technique*).tw,kf.	1911

84	(comput* adj2 reasoning*).tw,kf.	74
85	(predict* adj algorithm*).tw,kf.	4013
86	or/46-54,57,60-85	260181
87	45 and 86	40704
88	limit 87 to english language	38649
89	limit 88 to yr="2017 -Current"	19199
90	review/	2801172
91	(medline or medlars or pubmed or grateful med or CINAHL or scisearch or psychinfo or psycinfo or psychlit or psyclit or handsearch* or hand search* or manual* search* or electronic database* or bibliographic database* or embase or lilacs or scopus or web of science).mp.	298738
92	90 and 91	163246
93	meta-analysis.mp.	225511
94	meta-analysis as topic/	21979
95	meta-analysis/	173023
96	systematic review*.tw,kf.	219557
97	cochrane database*.jn.	15785
98	or/92-97	418021
99	89 and 98	1166

Database: Embase
Search strategy:

#	Searches	Results
1	exp health personnel attitude/	204464
2	exp health care personnel/	1954504
3	exp hospital personnel/	148628
4	exp multidisciplinary team/	23776
5	social work/	30146
6	social worker/	16284
7	licensed practical nurse/	376

8	exp nurse/	216887
9	nursing staff/	77544
10	exp nursing/	415631
11	practical nursing/	166
12	exp health educator/	3410
13	exp administrative personnel/	34859
14	nurse attitude/	43316
15	(health* adj2 provider?).tw,kw.	112571
16	(hospital* adj2 provider?).tw,kw.	2734
17	(health* adj2 staff?).tw,kw.	12984
18	(hospital* adj2 staff?).tw,kw.	12733
19	(health* adj2 personnel?).tw,kw.	14516
20	(hospital* adj2 personnel?).tw,kw.	3881
21	(health* adj2 employe*).tw,kw.	8982
22	(hospital* adj2 employe*).tw,kw.	3630
23	(health* adj2 profession*).tw,kw.	185144
24	(hospital* adj2 profession*).tw,kw.	2403
25	(health* adj2 occupation?).tw,kw.	1499
26	(hospital* adj2 occupation?).tw,kw.	179
27	(health* adj2 practitioner?).tw,kw.	16289
28	(hospital* adj2 practitioner?).tw,kw.	1776
29	(health* adj2 worker?).tw,kw.	79258
30	(hospital* adj2 worker?).tw,kw.	3043
31	nurs*.tw,kw.	626690
32	(patient care? adj2 team?).tw,kw.	861
33	(health* adj2 educator?).tw,kw.	4342
34	(social adj2 worker?).tw,kw.	21154
35	(support* adj2 (worker? or staff?)).tw,kw.	11354
36	clinician?.tw,kw.	408904
37	doctor?.tw,kw.	211722
38	physician?.tw,kw.	655120

39	practitioner?.tw,kw.	226114
40	surgeon?.tw,kw.	381219
41	caregiver/	106071
42	(caregiver? or care-giver? or carer or carers).tw,kw.	147337
43	or/1-42	3757068
44	(predictive adj2 analy*).tw,kw.	8865
45	predictive model*.tw,kw.	39403
46	predictive monitoring.tw,kw.	77
47	predictive technolog*.tw,kw.	64
48	real time analytic*.tw,kw.	162
49	probability based.tw,kw.	1963
50	predictive algorithm*.tw,kw.	1711
51	risk assessment software tool?.tw,kw.	3
52	(command center? or command centre?).tw,kw.	299
53	risk assessment/	700418
54	software/	109045
55	53 and 54	3765
56	(risk assess* or risks assess* or assess* risk or assess* risks or risk analysis or risks analysis or risk analyses or risks analyses).tw,kw.	148913
57	(software or computer program*).tw,kw.	382484
58	56 and 57	3153
59	artificial intelligence/	50376
60	(artificial intelligence or computational intelligence or computer reasoning or computer vision system or computer vision systems or machine intelligence).tw,kw.	35894
61	exp machine learning/	358057
62	(machine learning or transfer learning or learning machine or learning machines).tw,kw.	95321
63	Bayes theorem/	41514
64	(bayes decision procedure or bayes theorem or bayesian analysis or bayesian approach* or bayesian decision* or bayesian estimation* or bayesian forecast* or byesian modeling or bayesian method* or bayesian prediction* or naive bayesian classifier).tw,kw.	19007

65	deep learning/	33372
66	(deep learning or hierarchical learning).tw,kw.	46637
67	clinical decision rule/	551
68	(clinical decision rule or clinical decision rules or clinical prediction rule or clinical prediction rules).tw,kw.	3533
69	(electronic prediction rule or electronic prediction rules).tw,kw.	3
70	early warning score/ or modified early warning score/	1061
71	(early warning score or early warning scores or early warning scoring or mew score* or "modified early warning (MEW) score*" or early warning sign score* or "early warning (PEW) score*").tw,kw.	2617
72	early warning system*.tw,kw.	3245
73	decision support system/	26155
74	(decision analyses or decision analysis or decision modeling or decision support model or decision support models or decision support technic or decision support technics or decision support technique or decision support techniques).tw,kw.	9363
75	automated pattern recognition/	17455
76	(automat* adj2 pattern* adj2 analy*).tw,kw.	72
77	(automat* adj2 pattern* adj2 recog*).tw,kw.	188
78	(comput* adj2 pattern?* adj2 analy*).tw,kw.	94
79	(comput* adj2 pattern* adj2 recog*).tw,kw.	213
80	decision support system/	26155
81	computer assisted diagnosis/	43414
82	computer assisted therapy/ or computer assisted drug therapy/	5718
83	(automatic diagnosis or computer assisted decision making or computer assisted decision making medical decision making or computer assisted diagnoses or computer assisted diagnosis or computer assisted protocol directed therapies or computer assisted protocol directed therapy or computer assisted therapies or computer assisted therapy or computer diagnosis or decision support technique*).tw,kw.	3336
84	(comput* adj2 reasoning*).tw,kw.	118
85	(predict* adj algorithm*).tw,kw.	6900
86	or/44-52,55,58-85	570414
87	43 and 86	64927

88	limit 87 to english language	63246
89	limit 88 to yr="2017 -Current"	40104
90	limit 89 to (books or chapter or conference abstract or conference paper or "conference review")	11592
91	89 not 90	28512
92	"review"/	2870230
93	(medline or medlars or pubmed or grateful med or CINAHL or scisearch or psychinfo or psycinfo or psychlit or psyclit or handsearch* or hand search* or manual* search* or electronic database* or bibliographic database* or embase or lilacs or scopus or web of science).mp.	458235
94	92 and 93	184553
95	meta-analysis.mp.	387361
96	"meta analysis (topic)"/	51567
97	meta analysis/	267084
98	systematic review*.tw,kw.	331134
99	cochrane database*.jn.	22828
100	or/94-99	637208
101	91 and 100	1377

Database: Cumulative Index to Nursing and Allied Health (CINAHL)
Search strategy:

#	Query	Last Run Via	Results
S1	(MH "Attitude of Health Personnel+")	Interface - EBSCOhost Research Databases<br>Search Screen - Advanced Search<br>Database - CINAHL Ultimate	121,776
S2	(MH "Health Personnel+")	Interface - EBSCOhost Research Databases<br>Search Screen - Advanced Search<br>Database - CINAHL Ultimate	637,326
S3	(MH "Personnel, Health Facility+")	Interface - EBSCOhost Research Databases<br>Search Screen - Advanced Search<br>Database - CINAHL Ultimate	44,565

S4	(MH "Health Occupations+")	Interface - EBSCOhost Research Databases<br>Search Screen - Advanced Search<br>Database - CINAHL Ultimate	825,008
S5	(MH "Multidisciplinary Care Team+")	Interface - EBSCOhost Research Databases<br>Search Screen - Advanced Search<br>Database - CINAHL Ultimate	51,589
S6	(MH "Social Work+")	Interface - EBSCOhost Research Databases<br>Search Screen - Advanced Search<br>Database - CINAHL Ultimate	15,285
S7	(MH "Social Workers")	Interface - EBSCOhost Research Databases<br>Search Screen - Advanced Search<br>Database - CINAHL Ultimate	11,127
S8	(MH "Nursing Role")	Interface - EBSCOhost Research Databases<br>Search Screen - Advanced Search<br>Database - CINAHL Ultimate	65,457
S9	(MH "Practical Nurses") OR (MH "Practical Nursing")	Interface - EBSCOhost Research Databases<br>Search Screen - Advanced Search<br>Database - CINAHL Ultimate	7,716
S10	(MH "Nurses+")	Interface - EBSCOhost Research Databases<br>Search Screen - Advanced Search<br>Database - CINAHL Ultimate	241,738
S11	(MH "Nursing Staff, Hospital")	Interface - EBSCOhost Research Databases<br>Search Screen - Advanced Search<br>Database - CINAHL Ultimate	24,943
S12	(MH "Health Educators+")	Interface - EBSCOhost Research Databases<br>Search Screen - Advanced Search<br>Database - CINAHL Ultimate	4,207
S13	(MH "Administrative Personnel+")	Interface - EBSCOhost Research Databases<br>Search Screen - Advanced Search<br>Database - CINAHL Ultimate	40,581
S14	TI (health* N2 provider#) OR AB (health* N2 provider#)	Interface - EBSCOhost Research Databases<br>Search Screen - Advanced Search<br>Database - CINAHL Ultimate	57,388
S15	TI (hospital* N2 provider#) OR AB (hospital* N2 provider#)	Interface - EBSCOhost Research Databases<br>Search Screen - Advanced Search<br>Database - CINAHL Ultimate	1,817
S16	TI (health* N2 staff#) OR AB (health* N2 staff#)	Interface - EBSCOhost Research Databases<br>Search Screen - Advanced Search<br>Database - CINAHL Ultimate	8,911

S17	TI (hospital* N2 staff#) OR AB (hospital* N2 staff#)	Interface - EBSCOhost Research Databases Search Screen - Advanced Search Database - CINAHL Ultimate	6,037
S18	TI (health* N2 personnel#) OR AB (health* N2 personnel#)	Interface - EBSCOhost Research Databases Search Screen - Advanced Search Database - CINAHL Ultimate	5,418
S19	TI (hospital* N2 personnel#) OR AB (hospital* N2 personnel#)	Interface - EBSCOhost Research Databases Search Screen - Advanced Search Database - CINAHL Ultimate	1,154
S20	TI (health* N2 employe*) OR AB (health* N2 employe*)	Interface - EBSCOhost Research Databases Search Screen - Advanced Search Database - CINAHL Ultimate	7,106
S21	TI (hospital* N2 employe*) OR AB (hospital* N2 employe*)	Interface - EBSCOhost Research Databases Search Screen - Advanced Search Database - CINAHL Ultimate	2,065
S22	TI (health* N2 profession*) OR AB (health* N2 profession*)	Interface - EBSCOhost Research Databases Search Screen - Advanced Search Database - CINAHL Ultimate	100,273
S23	TI (hospital* N2 profession*) OR AB (hospital* N2 profession*)	Interface - EBSCOhost Research Databases Search Screen - Advanced Search Database - CINAHL Ultimate	1,973
S24	TI (health* N2 occupation#) OR AB (health* N2 occupation#)	Interface - EBSCOhost Research Databases Search Screen - Advanced Search Database - CINAHL Ultimate	1,011
S25	TI (hospital* N2 occupation#) OR AB (hospital* N2 occupation#)	Interface - EBSCOhost Research Databases Search Screen - Advanced Search Database - CINAHL Ultimate	58
S26	TI (health* N2 practitioner#) OR AB (health* N2 practitioner#)	Interface - EBSCOhost Research Databases Search Screen - Advanced Search Database - CINAHL Ultimate	12,131
S27	TI (hospital* N2 practitioner#) OR AB (hospital* N2 practitioner#)	Interface - EBSCOhost Research Databases Search Screen - Advanced Search Database - CINAHL Ultimate	922
S28	TI (health* N2 worker#) OR AB (health* N2 worker#)	Interface - EBSCOhost Research Databases Search Screen - Advanced Search Database - CINAHL Ultimate	33,851
S29	TI (hospital* N2 worker#) OR AB (hospital* N2 worker#)	Interface - EBSCOhost Research Databases Search Screen - Advanced Search Database - CINAHL Ultimate	1,582

S30	TI nurs* OR AB nurs*	Interface - EBSCOhost Research Databases Search Screen - Advanced Search Database - CINAHL Ultimate	639,557
S31	TI (patient care# N2 team#) OR AB (patient care# N2 team#)	Interface - EBSCOhost Research Databases Search Screen - Advanced Search Database - CINAHL Ultimate	587
S32	TI (health* N2 educator#) OR AB (health* N2 educator#)	Interface - EBSCOhost Research Databases Search Screen - Advanced Search Database - CINAHL Ultimate	3,744
S33	TI (social N2 worker#) OR AB (social N2 worker#)	Interface - EBSCOhost Research Databases Search Screen - Advanced Search Database - CINAHL Ultimate	14,742
S34	TI (support* N2 (worker# or staff#)) OR AB (support* N2 (worker# or staff#))	Interface - EBSCOhost Research Databases Search Screen - Advanced Search Database - CINAHL Ultimate	9,580
S35	TI clinician# OR AB clinician#	Interface - EBSCOhost Research Databases Search Screen - Advanced Search Database - CINAHL Ultimate	122,738
S36	TI doctor# OR AB doctor#	Interface - EBSCOhost Research Databases Search Screen - Advanced Search Database - CINAHL Ultimate	65,207
S37	TI physician# OR AB physician#	Interface - EBSCOhost Research Databases Search Screen - Advanced Search Database - CINAHL Ultimate	168,960
S38	TI practitioner# OR AB practitioner#	Interface - EBSCOhost Research Databases Search Screen - Advanced Search Database - CINAHL Ultimate	107,504
S39	TI surgeon# OR AB surgeon#	Interface - EBSCOhost Research Databases Search Screen - Advanced Search Database - CINAHL Ultimate	63,230
S40	(MH "Caregivers")	Interface - EBSCOhost Research Databases Search Screen - Advanced Search Database - CINAHL Ultimate	42,730
S41	TI (caregiver# or care-giver# or carer or carers) OR AB (caregiver# or care-giver# or carer or carers)	Interface - EBSCOhost Research Databases Search Screen - Advanced Search Database - CINAHL Ultimate	79,187
S42	S1 OR S2 OR S3 OR S4 OR S5 OR S6 OR S7 OR S8 OR S9 OR S10 OR S11 OR S12 OR S13 OR S14 OR S15 OR S16 OR S17 OR S18 OR S19 OR S20 OR S21 OR S22 OR S23 OR S24 OR S25 OR S26 OR S27 OR	Interface - EBSCOhost Research Databases Search Screen - Advanced Search Database - CINAHL Ultimate	2,190,126

	S28 OR S29 OR S30 OR S31 OR S32 OR S33 OR S34 OR S35 OR S36 OR S37 OR S38 OR S39 OR S40 OR S41		
S43	TI (predictive N1 analy*) OR AB (predictive N1 analy*)	Interface - EBSCOhost Research Databases Search Screen - Advanced Search Database - CINAHL Ultimate	1,667
S44	TI (predictive model*) OR AB (predictive model*)	Interface - EBSCOhost Research Databases Search Screen - Advanced Search Database - CINAHL Ultimate	6,318
S45	TI (predictive monitoring) OR AB (predictive monitoring)	Interface - EBSCOhost Research Databases Search Screen - Advanced Search Database - CINAHL Ultimate	11
S46	TI (predictive technolog*) OR AB (predictive technolog*)	Interface - EBSCOhost Research Databases Search Screen - Advanced Search Database - CINAHL Ultimate	11
S47	TI (real time analytic*) OR AB (real time analytic*)	Interface - EBSCOhost Research Databases Search Screen - Advanced Search Database - CINAHL Ultimate	32
S48	TI (probability based) OR AB (probability based)	Interface - EBSCOhost Research Databases Search Screen - Advanced Search Database - CINAHL Ultimate	465
S49	TI (predictive algorithm*) OR AB (predictive algorithm*)	Interface - EBSCOhost Research Databases Search Screen - Advanced Search Database - CINAHL Ultimate	260
S50	TI (risk assessment software tool#) OR AB (risk assessment software tool#)	Interface - EBSCOhost Research Databases Search Screen - Advanced Search Database - CINAHL Ultimate	1
S51	TI (command center# or command centre#) OR AB (command center# or command centre#)	Interface - EBSCOhost Research Databases Search Screen - Advanced Search Database - CINAHL Ultimate	113
S52	(MH "Risk Assessment")	Interface - EBSCOhost Research Databases Search Screen - Advanced Search Database - CINAHL Ultimate	141,470
S53	(MH "Software")	Interface - EBSCOhost Research Databases Search Screen - Advanced Search Database - CINAHL Ultimate	32,440
S54	S52 AND S53	Interface - EBSCOhost Research Databases Search Screen - Advanced Search Database - CINAHL Ultimate	420

S55	TI (risk assess* or risks assess* or assess* risk or assess* risks or risk analysis or risks analysis or risk analyses or risks analyses) OR AB (risk assess* or risks assess* or assess* risk or assess* risks or risk analysis or risks analysis or risk analyses or risks analyses)	Interface - EBSCOhost Research Databases Search Screen - Advanced Search Database - CINAHL Ultimate	26,807
S56	TI (software or computer program*) OR AB (software or computer program*)	Interface - EBSCOhost Research Databases Search Screen - Advanced Search Database - CINAHL Ultimate	61,647
S57	S55 AND S56	Interface - EBSCOhost Research Databases Search Screen - Advanced Search Database - CINAHL Ultimate	929
S58	(MH "Machine Learning+")	Interface - EBSCOhost Research Databases Search Screen - Advanced Search Database - CINAHL Ultimate	5,286
S59	TI (machine learning or transfer learning or learning machine or learning machines) OR AB (machine learning or transfer learning or learning machine or learning machines)	Interface - EBSCOhost Research Databases Search Screen - Advanced Search Database - CINAHL Ultimate	11,924
S60	TI (bayes decision procedure or bayes theorem or bayesian analysis or bayesian approach* or bayesian decision* or bayesian estimation* or bayesian forecast* or byesian modeling or bayesian method* or bayesian prediction* or naive bayesian classifier) OR AB (bayes decision procedure or bayes theorem or bayesian analysis or bayesian approach* or bayesian decision* or bayesian estimation* or bayesian forecast* or byesian modeling or bayesian method* or bayesian prediction* or naive bayesian classifier)	Interface - EBSCOhost Research Databases Search Screen - Advanced Search Database - CINAHL Ultimate	2,264
S61	(MH "Deep Learning")	Interface - EBSCOhost Research Databases Search Screen - Advanced Search Database - CINAHL Ultimate	1,442
S62	TI (deep learning or hierarchical learning). OR AB (deep learning or hierarchical learning)	Interface - EBSCOhost Research Databases Search Screen - Advanced Search Database - CINAHL Ultimate	4,604

S63	(MH "Clinical Prediction Rules")	Interface - EBSCOhost Research Databases Search Screen - Advanced Search Database - CINAHL Ultimate	38
S64	TI (clinical decision rule or clinical decision rules or clinical prediction rule or clinical prediction rules) OR AB (clinical decision rule or clinical decision rules or clinical prediction rule or clinical prediction rules)	Interface - EBSCOhost Research Databases Search Screen - Advanced Search Database - CINAHL Ultimate	1,306
S65	TI (electronic prediction rule or electronic prediction rules) OR AB (electronic prediction rule or electronic prediction rules)	Interface - EBSCOhost Research Databases Search Screen - Advanced Search Database - CINAHL Ultimate	1
S66	(MH "Early Warning Score")	Interface - EBSCOhost Research Databases Search Screen - Advanced Search Database - CINAHL Ultimate	143
S67	TI (early warning score or early warning scores or early warning scoring or mew score* or "modified early warning (MEW) score*" or early warning sign score* or "early warning (PEW) score*" or early warning system*) OR AB (early warning score or early warning scores or early warning scoring or mew score* or "modified early warning (MEW) score*" or early warning sign score* or "early warning (PEW) score*" or early warning system*)	Interface - EBSCOhost Research Databases Search Screen - Advanced Search Database - CINAHL Ultimate	1,715
S68	(MH "Decision Support Techniques")	Interface - EBSCOhost Research Databases Search Screen - Advanced Search Database - CINAHL Ultimate	7,765
S69	TI (decision analyses or decision analysis or decision modeling or decision support model or decision support models or decision support technic or decision support technics or decision support technique or decision support techniques) OR AB (decision analyses or decision analysis or decision modeling or decision support model or decision support models or decision support technic or decision support technics or	Interface - EBSCOhost Research Databases Search Screen - Advanced Search Database - CINAHL Ultimate	1,898

	decision support technique or decision support techniques)		
S70	TI (automat* N1 pattern* N1 analy*) OR AB (automat* N1 pattern* N1 analy*)	Interface - EBSCOhost Research Databases Search Screen - Advanced Search Database - CINAHL Ultimate	13
S71	TI (automat* N1 pattern* N1 recog*) OR AB (automat* N1 pattern* N1 recog*)	Interface - EBSCOhost Research Databases Search Screen - Advanced Search Database - CINAHL Ultimate	14
S72	TI (comput* N1 pattern* N1 analy*) OR AB (comput* N1 pattern* N1 analy*)	Interface - EBSCOhost Research Databases Search Screen - Advanced Search Database - CINAHL Ultimate	8
S73	TI (comput* N1 pattern* N1 recog*) OR AB (comput* N1 pattern* N1 recog*)	Interface - EBSCOhost Research Databases Search Screen - Advanced Search Database - CINAHL Ultimate	10
S74	(MH "Decision Making, Computer Assisted") OR (MH "Diagnosis, Computer Assisted") OR (MH "Therapy, Computer Assisted") OR (MH "Drug Therapy, Computer Assisted")	Interface - EBSCOhost Research Databases Search Screen - Advanced Search Database - CINAHL Ultimate	12,178
S75	TI (automatic diagnosis or computer assisted decision making or computer assisted decision making medical decision making or computer assisted diagnoses or computer assisted diagnosis or computer assisted protocol directed therapies or computer assisted protocol directed therapy or computer assisted therapies or computer assisted therapy or computer diagnosis or decision support technique*) OR AB (automatic diagnosis or computer assisted decision making or computer assisted decision making medical decision making or computer assisted diagnoses or computer assisted diagnosis or computer assisted protocol directed therapies or computer assisted protocol directed therapy or computer assisted therapies or computer assisted therapy or computer diagnosis or decision support technique*)	Interface - EBSCOhost Research Databases Search Screen - Advanced Search Database - CINAHL Ultimate	232

S76	TI (comput* N1 reasoning*) OR AB (comput* N1 reasoning*)	Interface - EBSCOhost Research Databases Search Screen - Advanced Search Database - CINAHL Ultimate	21
S77	TI (predict* N1 algorithm) OR AB (predict* N1 algorithm)	Interface - EBSCOhost Research Databases Search Screen - Advanced Search Database - CINAHL Ultimate	1,697
S78	(MH "Artificial Intelligence")	Interface - EBSCOhost Research Databases Search Screen - Advanced Search Database - CINAHL Ultimate	8,427
S79	TI (artificial intelligence or computational intelligence or computer reasoning or computer vision system or computer vision systems or machine intelligence). OR AB (artificial intelligence or computational intelligence or computer reasoning or computer vision system or computer vision systems or machine intelligence)	Interface - EBSCOhost Research Databases Search Screen - Advanced Search Database - CINAHL Ultimate	7,239
S80	S43 OR S44 OR S45 OR S46 OR S47 OR S48 OR S49 OR S50 OR S51 OR S54 OR S57 OR S58 OR S59 OR S60 OR S61 OR S62 OR S63 OR S64 OR S65 OR S66 OR S67 OR S68 OR S69 OR S70 OR S71 OR S72 OR S73 OR S74 OR S75 OR S76 OR S77 OR S79 OR S80	Interface - EBSCOhost Research Databases Search Screen - Advanced Search Database - CINAHL Ultimate	56,949
S81	S42 AND S80	Interface - EBSCOhost Research Databases Search Screen - Advanced Search Database - CINAHL Ultimate	14,649
S82	(MH "Literature Review")	Interface - EBSCOhost Research Databases Search Screen - Advanced Search Database - CINAHL Ultimate	8,989
S83	TI (medline OR medlars OR pubmed OR grateful med OR CINAHL OR scisearch OR psychinfo OR psycinfo OR psychlit OR psyclit OR handsearch* OR "hand search*" OR "manual* search*" OR "electronic database*" OR "bibliographic database*" OR embase OR lilacs OR scopus OR "web of science") OR AB (medline OR medlars OR pubmed OR grateful med OR CINAHL OR scisearch OR psychinfo OR psycinfo OR psychlit OR psyclit OR handsearch* OR "hand	Interface - EBSCOhost Research Databases Search Screen - Advanced Search Database - CINAHL Ultimate	138,314

	search*" OR "manual* search*" OR "electronic database*" OR "bibliographic database*" OR embase OR lilacs OR scopus OR "web of science")		
S84	S82 AND S83	Interface - EBSCOhost Research Databases Search Screen - Advanced Search Database - CINAHL Ultimate	1,144
S85	TI ("meta-analysis") OR AB ("meta-analysis")	Interface - EBSCOhost Research Databases Search Screen - Advanced Search Database - CINAHL Ultimate	89,292
S86	(MH "Meta Analysis")	Interface - EBSCOhost Research Databases Search Screen - Advanced Search Database - CINAHL Ultimate	68,098
S87	TI (systematic review*) OR AB (systematic review*)	Interface - EBSCOhost Research Databases Search Screen - Advanced Search Database - CINAHL Ultimate	135,597
S88	JN cochrane database*	Interface - EBSCOhost Research Databases Search Screen - Advanced Search Database - CINAHL Ultimate	6,023
S89	S84 OR S85 OR S86 OR S87 OR S88	Interface - EBSCOhost Research Databases Search Screen - Advanced Search Database - CINAHL Ultimate	184,194
S90	S80 AND S89	Interface - EBSCOhost Research Databases Search Screen - Advanced Search Database - CINAHL Ultimate	2,477
S91	S80 AND S89	Interface - EBSCOhost Research Databases Search Screen - Advanced Search Database - CINAHL Ultimate	2,462
S92	S80 AND S89	Interface - EBSCOhost Research Databases Search Screen - Advanced Search Database - CINAHL Ultimate	1,315

- **Recommendation Question #5:** Should a distributive model (versus no distributive model or any other type of change management model) be recommended to integrate digital health competencies into the professional practice roles and responsibilities of nurses at all levels within an organization?

Dates searched: January 1, 2017 – May 17, 2022

Databases searched:

- MEDLINE

- MEDLINE Epub Ahead of Print and In-Process
- Embase
- Emcare Nursing
- Cochrane Central Register of Controlled Trials
- Cochrane Database of Systematic Reviews
- APA PsychInfo
- Cumulative Index to Nursing and Allied Health (CINAHL)
- IEEE Explore *(search run July 14, 2022)*

Database: MEDLINE
Search strategy:

#	Searches	Results
1	nurses/ or nurse administrators/ or nurse practitioners/ or family nurse practitioners/ or pediatric nurse practitioners/ or nurse specialists/ or nurse anesthetists/ or nurse clinicians/ or nurse midwives/ or nurses, pediatric/ or nurses, neonatal/ or nurses, community health/ or nurses, public health/	93806
2	nursing staff/ or nursing staff, hospital/	69010
3	nursing care/ or cardiovascular nursing/ or critical care nursing/ or developmental disability nursing/ or emergency nursing/ or geriatric nursing/ or holistic nursing/ or home nursing/ or "hospice and palliative care nursing"/ or maternal-child nursing/ or medical-surgical nursing/ or nephrology nursing/ or neuroscience nursing/ or nursing, practical/ or obstetric nursing/ or occupational health nursing/ or oncology nursing/ or orthopedic nursing/ or pediatric nursing/ or perioperative nursing/ or primary nursing/ or psychiatric nursing/ or rehabilitation nursing/ or trauma nursing/	128169
4	primary care nursing/	552
5	specialties, nursing/ or advanced practice nursing/ or cardiovascular nursing/ or community health nursing/ or home health nursing/ or parish nursing/ or critical care nursing/ or developmental disability nursing/ or emergency nursing/ or family nursing/ or forensic nursing/ or geriatric nursing/ or holistic nursing/ or "hospice and palliative care nursing"/ or maternal-child nursing/ or neonatal nursing/ or medical-surgical nursing/ or midwifery/ or military nursing/ or nephrology nursing/ or neuroscience nursing/ or obstetric nursing/ or occupational health nursing/ or oncology nursing/ or orthopedic nursing/ or pediatric nursing/ or perioperative nursing/ or operating room nursing/ or postanesthesia nursing/ or psychiatric nursing/ or public health nursing/ or "radiologic and imaging nursing"/ or rehabilitation nursing/ or rural nursing/ or school nursing/ or transcultural nursing/ or trauma nursing/	162806

6	Nursing, Practical/	3442
7	Nurse's Role/	42400
8	nursing/ or nursing, private duty/ or nursing, supervisory/ or office nursing/ or telenursing/ or travel nursing/	60712
9	nursing process/ or nursing assessment/ or nursing diagnosis/	38410
10	nursing services/ or home care services/ or home health nursing/ or nursing service, hospital/	51438
11	Nursing, Team/	2593
12	Nursing Faculty Practice/	614
13	(nurse or nurses or nursing).tw,kf.	447148
14	or/1-13 [Nurses]	660941
15	(distribut* adj1 leader*).tw,kf.	111
16	(distribut* adj1 education).tw,kf.	99
17	(distribut* adj1 learning).tw,kf.	204
18	(distribut* adj1 (model or models)).tw,kf.	3215
19	(distribut* adj1 governance).tw,kf.	6
20	(distribut* adj1 (framework or frameworks or frame work or frame works)).tw,kf.	45
21	((share or shares or shared or sharing) adj1 leader*).tw,kf.	304
22	((share or shares or shared or sharing) adj1 education).tw,kf.	117
23	Shared Governance, Nursing/	14
24	((share or shares or shared or sharing) adj1 governance).tw,kf.	526
25	((share or shares or shared or sharing) adj1 responsibilit*).tw,kf.	1561
26	(participative governance or participatory governance or participative management).tw,kf.	180
27	or/15-26	6317
28	14 and 27	925
29	limit 28 to english language	874
30	limit 29 to yr="2017 -Current"	212

Database: MEDLINE Epub Ahead of Print and In-Process
Search strategy:

#	Searches	Results
1	(nurse or nurses or nursing).tw,kf.	44177
2	(distribut* adj1 leader*).tw,kf.	32
3	(distribut* adj1 education).tw,kf.	11
4	(distribut* adj1 learning).tw,kf.	124
5	(distribut* adj1 (model or models)).tw,kf.	1025
6	(distribut* adj1 governance).tw,kf.	2
7	(distribut* adj1 (framework or frameworks or frame work or frame works)).tw,kf.	36
8	((share or shares or shared or sharing) adj1 leader*).tw,kf.	57
9	((share or shares or shared or sharing) adj1 education).tw,kf.	21
10	((share or shares or shared or sharing) adj1 governance).tw,kf.	37
11	((share or shares or shared or sharing) adj1 responsibilit*).tw,kf.	237
12	(participative governance or participatory governance or participative management).tw,kf.	26
13	or/2-11	1571
14	1 and 13	66
15	limit 14 to english language	65
16	limit 15 to yr="2017 -Current"	44

Database: Embase
Search Strategy:

#	Searches	Results
1	nurse/ or expert nurse/ or licensed practical nurse/ or nurse consultant/ or practical nurse/ or registered nurse/ or staff nurse/	151636
2	nursing/ or practical nursing/ or telenursing/ or travel nursing/	236477
3	advanced practice nurse/	981
4	nurse administrator/ or charge nurse/ or head nurse/ or nurse manager/	15368
5	nurse practitioner/ or acute care nurse practitioner/ or adult nurse practitioner/ or emergency nurse practitioner/ or family nurse practitioner/ or gerontologic nurse practitioner/ or infection control practitioner/ or neonatal nurse practitioner/ or pediatric nurse practitioner/	28350

6	nurse specialist/ or clinical nurse specialist/ or neonatal nurse/ or nurse anesthetist/ or nurse midwife/ or oncology nurse/ or pediatric nurse/	14971
7	nursing staff/	76413
8	nursing care/ or visiting nursing service/	38041
9	nursing discipline/ or acquired immune deficiency syndrome nursing/ or addictions nursing/ or ambulatory care nursing/ or anesthesia nursing/ or burn nursing/ or camp nursing/ or cardiovascular nursing/ or dermatology nursing/ or emergency nursing/ or enterostomal therapy nursing/ or family nursing/ or flight nursing/ or forensic nursing/ or gastroenterology nursing/ or genetics nursing/ or gynecologic nursing/ or hospice nursing/ or intravenous nursing/ or learning disability nursing/ or military nursing/ or nephrology nursing/ or neuroscience nursing/ or nurse midwifery/ or obstetrical nursing/ or occupational health nursing/ or ophthalmic nursing/ or orthopedic nursing/ or palliative nursing/ or parish nursing/ or perinatal nursing/ or perioperative nursing/ or postanesthesia nursing/ or prison nursing/ or radiology nursing/ or rehabilitation nursing/ or respiratory nursing/ or rural health nursing/ or school health nursing/ or urologic nursing/	46055
10	community health nursing/ or community psychiatric nursing/	26954
11	community health nursing/ or community psychiatric nursing/	26954
12	geriatric nursing/ or psychogeriatric nursing/	13089
13	intensive care nursing/ or newborn intensive care nursing/ or pediatric intensive care nursing/	3079
14	newborn nursing/	3908
15	oncology nursing/ or pediatric oncology nursing/	8447
16	pediatric nursing/	13215
17	psychiatric nursing/	16951
18	surgical nursing/ or plastic surgical nursing/	285
19	advanced practice nursing/	2135
20	practical nursing/	159
21	nursing role/	663
22	nursing process/	7080
23	nursing assessment/	27407
24	nursing diagnosis/	4749

25	team nursing/	430
26	(nurse or nurses or nursing).tw,kw.	585246
27	or/1-26	813001
28	(distribut* adj1 leader*).tw,kw.	157
29	(distribut* adj1 education).tw,kw.	178
30	(distribut* adj1 learning).tw,kw.	346
31	(distribut* adj1 (model or models)).tw,kw.	4210
32	(distribut* adj1 governance).tw,kw.	12
33	(distribut* adj1 (framework or frameworks or frame work or frame works)).tw,kw.	89
34	((share or shares or shared or sharing) adj1 leader*).tw,kw.	369
35	((share or shares or shared or sharing) adj1 education).tw,kw.	202
36	nursing shared governance/	3
37	((share or shares or shared or sharing) adj1 governance).tw,kw.	585
38	((share or shares or shared or sharing) adj1 responsibilit*).tw,kw.	2186
39	participatory management/	1065
40	(participative governance or participatory governance or participative management).tw,kw.	240
41	or/28-40	9457
42	27 and 41	1171
43	limit 42 to english language	1116
44	limit 43 to yr="2017 -Current"	289
45	limit 44 to (books or chapter or conference abstract or conference paper or "conference review")	53
46	44 not 45	236

Database: Emcare Nursing
Search strategy:

#	Searches	Results
1	nurse/ or expert nurse/ or licensed practical nurse/ or nurse consultant/ or practical nurse/ or registered nurse/ or staff nurse/	144931
2	nursing/ or practical nursing/ or telenursing/ or travel nursing/	99463

3	advanced practice nurse/	2284
4	nurse administrator/ or charge nurse/ or head nurse/ or nurse manager/	8102
5	nurse practitioner/ or acute care nurse practitioner/ or adult nurse practitioner/ or emergency nurse practitioner/ or family nurse practitioner/ or gerontologic nurse practitioner/ or infection control practitioner/ or neonatal nurse practitioner/ or pediatric nurse practitioner/	15481
6	nurse specialist/ or clinical nurse specialist/ or neonatal nurse/ or nurse anesthetist/ or nurse midwife/ or oncology nurse/ or pediatric nurse/	6246
7	nursing staff/	26804
8	nursing care/ or visiting nursing service/	18173
9	nursing discipline/ or acquired immune deficiency syndrome nursing/ or addictions nursing/ or ambulatory care nursing/ or anesthesia nursing/ or burn nursing/ or camp nursing/ or cardiovascular nursing/ or dermatology nursing/ or emergency nursing/ or enterostomal therapy nursing/ or family nursing/ or flight nursing/ or forensic nursing/ or gastroenterology nursing/ or genetics nursing/ or gynecologic nursing/ or hospice nursing/ or intravenous nursing/ or learning disability nursing/ or military nursing/ or nephrology nursing/ or neuroscience nursing/ or nurse midwifery/ or obstetrical nursing/ or occupational health nursing/ or ophthalmic nursing/ or orthopedic nursing/ or palliative nursing/ or parish nursing/ or perinatal nursing/ or perioperative nursing/ or postanesthesia nursing/ or prison nursing/ or radiology nursing/ or rehabilitation nursing/ or respiratory nursing/ or rural health nursing/ or school health nursing/ or urologic nursing/	14840
10	community health nursing/ or community psychiatric nursing/	5260
11	community health nursing/ or community psychiatric nursing/	5260
12	geriatric nursing/ or psychogeriatric nursing/	2370
13	intensive care nursing/ or newborn intensive care nursing/ or pediatric intensive care nursing/	2028
14	newborn nursing/	2246
15	oncology nursing/ or pediatric oncology nursing/	2559
16	pediatric nursing/	2335
17	psychiatric nursing/	4334
18	surgical nursing/ or plastic surgical nursing/	456
19	advanced practice nursing/	1600

20	practical nursing/	187
21	nursing role/	1899
22	nursing process/	1746
23	nursing assessment/	4721
24	nursing diagnosis/	1716
25	team nursing/	183
26	(nurse or nurses or nursing).tw,kw.	293580
27	or/1-26	351181
28	(distribut* adj1 leader*).tw,kw.	121
29	(distribut* adj1 education).tw,kw.	60
30	(distribut* adj1 learning).tw,kw.	122
31	(distribut* adj1 (model or models)).tw,kw.	962
32	(distribut* adj1 governance).tw,kw.	5
33	(distribut* adj1 (framework or frameworks or frame work or frame works)).tw,kw.	30
34	((share or shares or shared or sharing) adj1 leader*).tw,kw.	292
35	((share or shares or shared or sharing) adj1 education).tw,kw.	101
36	nursing shared governance/	8
37	((share or shares or shared or sharing) adj1 governance).tw,kw.	420
38	((share or shares or shared or sharing) adj1 responsibilit*).tw,kw.	1103
39	participatory management/	956
40	(participative governance or participatory governance or participative management).tw,kw.	101
41	or/28-40	3829
42	27 and 41	739
43	limit 42 to english language	715
44	limit 43 to yr="2017 -Current"	220
45	limit 44 to (books or chapter or conference abstract or conference paper or "conference review" or dissertation)	3
46	44 not 45	217

Database: Cochrane Central Register of Controlled Trials
Search strategy:

#	Searches	Results
1	nurses/ or nurse administrators/ or nurse practitioners/ or family nurse practitioners/ or pediatric nurse practitioners/ or nurse specialists/ or nurse anesthetists/ or nurse clinicians/ or nurse midwives/ or nurses, pediatric/ or nurses, neonatal/ or nurses, community health/ or nurses, public health/	1322
2	nursing staff/ or nursing staff, hospital/	681
3	nursing care/ or cardiovascular nursing/ or critical care nursing/ or developmental disability nursing/ or emergency nursing/ or geriatric nursing/ or holistic nursing/ or home nursing/ or "hospice and palliative care nursing"/ or maternal-child nursing/ or medical-surgical nursing/ or nephrology nursing/ or neuroscience nursing/ or nursing, practical/ or obstetric nursing/ or occupational health nursing/ or oncology nursing/ or orthopedic nursing/ or pediatric nursing/ or perioperative nursing/ or primary nursing/ or psychiatric nursing/ or rehabilitation nursing/ or trauma nursing/	1634
4	primary care nursing/	35
5	specialties, nursing/ or advanced practice nursing/ or cardiovascular nursing/ or community health nursing/ or home health nursing/ or parish nursing/ or critical care nursing/ or developmental disability nursing/ or emergency nursing/ or family nursing/ or forensic nursing/ or geriatric nursing/ or holistic nursing/ or "hospice and palliative care nursing"/ or maternal-child nursing/ or neonatal nursing/ or medical-surgical nursing/ or midwifery/ or military nursing/ or nephrology nursing/ or neuroscience nursing/ or obstetric nursing/ or occupational health nursing/ or oncology nursing/ or orthopedic nursing/ or pediatric nursing/ or perioperative nursing/ or operating room nursing/ or postanesthesia nursing/ or psychiatric nursing/ or public health nursing/ or "radiologic and imaging nursing"/ or rehabilitation nursing/ or rural nursing/ or school nursing/ or transcultural nursing/ or trauma nursing/	2228
6	Nursing, Practical/	10
7	Nurse's Role/	384
8	nursing/ or nursing, private duty/ or nursing, supervisory/ or office nursing/ or telenursing/ or travel nursing/	123
9	nursing process/ or nursing assessment/ or nursing diagnosis/	568
10	nursing services/ or home care services/ or home health nursing/ or nursing service, hospital/	1966
11	Nursing, Team/	22

12	Nursing Faculty Practice/	4
13	(nurse or nurses or nursing).tw,kw.	44542
14	or/1-13 [Nurses]	47360
15	(distribut* adj1 leader*).tw,kw.	2
16	(distribut* adj1 education).tw,kw.	15
17	(distribut* adj1 learning).tw,kw.	12
18	(distribut* adj1 (model or models)).tw,kw.	79
19	(distribut* adj1 governance).tw,kw.	0
20	(distribut* adj1 (framework or frameworks or frame work or frame works)).tw,kw.	0
21	((share or shares or shared or sharing) adj1 leader*).tw,kw.	8
22	((share or shares or shared or sharing) adj1 education).tw,kw.	21
23	((share or shares or shared or sharing) adj1 governance).tw,kw.	6
24	((share or shares or shared or sharing) adj1 responsibilit*).tw,kw.	65
25	(participative governance or participatory governance or participative management).tw,kw.	1
26	or/15-25	209
27	14 and 26	25
28	limit 27 to english language	23
29	limit 28 to yr="2017 -Current"	9

Database: Cochrane Database of Systematic Reviews
Search Strategy:

#	Searches	Results
1	(nurse or nurses or nursing).mp.	3030
2	(distribut* adj1 leader*).mp.	1
3	(distribut* adj1 education).mp.	5
4	(distribut* adj1 learning).mp.	1
5	(distribut* adj1 (model or models)).mp.	4
6	(distribut* adj1 governance).mp.	0
7	(distribut* adj1 (framework or frameworks or frame work or frame works)).mp.	1
8	((share or shares or shared or sharing) adj1 leader*).mp.	3

9	((share or shares or shared or sharing) adj1 education).mp.	3
10	((share or shares or shared or sharing) adj1 governance).mp.	2
11	((share or shares or shared or sharing) adj1 responsibilit*).mp.	14
12	(participative governance or participatory governance or participative management).mp.	1
13	or/2-12	32
14	1 and 13	21
15	limit 14 to last 5 years	5

Database: APA PsycInfo
Search Strategy:

#	Searches	Results
1	nurses/ or psychiatric nurses/ or public health service nurses/	34388
2	nursing/	25083
3	(nurse or nurses or nursing).tw,id.	108162
4	or/1-3	108719
5	(distribut* adj1 leader*).tw,id.	603
6	(distribut* adj1 education).tw,id.	94
7	(distribut* adj1 learning).tw,id.	432
8	(distribut* adj1 (model or models)).tw,id.	531
9	(distribut* adj1 governance).tw,id.	6
10	(distribut* adj1 (framework or frameworks or frame work or frame works)).tw,id.	29
11	((share or shares or shared or sharing) adj1 leader*).tw,id.	984
12	((share or shares or shared or sharing) adj1 education).tw,id.	101
13	((share or shares or shared or sharing) adj1 governance).tw,id.	218
14	((share or shares or shared or sharing) adj1 responsibilit*).tw,id.	1274
15	participative management/	710
16	(participative governance or participatory governance or participative management).tw,id.	313
17	or/5-16	5044
18	4 and 17	223
19	limit 18 to english language	211

20	limit 19 to yr="2017 -Current"	62
21	limit 20 to peer reviewed journal	51

Database: CINAHL
Search Strategy:

#	Query	Limiters/Expanders	Results
S1	(MH "Nursing Manpower+")	Expanders - Apply equivalent subjects Search modes - Boolean/Phrase	265,823
S2	(MH "Nursing Role")	Expanders - Apply equivalent subjects Search modes - Boolean/Phrase	62,889
S3	(MH "Telenursing")	Expanders - Apply equivalent subjects Search modes - Boolean/Phrase	2,272
S4	(MH "Nursing Process")	Expanders - Apply equivalent subjects Search modes - Boolean/Phrase	3,671
S5	(MH "Nursing Assessment")	Expanders - Apply equivalent subjects Search modes - Boolean/Phrase	18,449
S6	(MH "Nursing Diagnosis")	Expanders - Apply equivalent subjects Search modes - Boolean/Phrase	4,740
S7	(MH "Nursing Service")	Expanders - Apply equivalent subjects Search modes - Boolean/Phrase	1,504
S8	(MH "Team Nursing") OR (MH "Nursing Care Delivery Systems") OR (MH "Differentiated Nursing Practice") OR (MH "Functional Nursing") OR (MH "Modular Nursing") OR (MH "Primary Nursing") OR (MH "Total Patient Care Nursing") OR (MH "Nursing Care")	Expanders - Apply equivalent subjects Search modes - Boolean/Phrase	29,381
S9	TI (nurse or nurses or nursing) OR AB (nurse or nurses or nursing)	Expanders - Apply equivalent subjects Search modes - Boolean/Phrase	598,666
S10	S1 OR S2 OR S3 OR S4 OR S5 OR S6 OR S7 OR S8 OR S9	Expanders - Apply equivalent subjects Search modes - Boolean/Phrase	735,282
S11	TI (distribut* leader*) OR AB (distribut* leader*)	Expanders - Apply equivalent subjects Search modes - Boolean/Phrase	84

S12	TI (distribut* education) OR AB (distribut* education)	Expanders - Apply equivalent subjects Search modes - Boolean/Phrase	25
S13	TI (distribut* learning) OR AB (distribut* learning)	Expanders - Apply equivalent subjects Search modes - Boolean/Phrase	81
S14	TI (distribut* model OR distribut* models) OR AB (distribut* model OR distribut* models)	Expanders - Apply equivalent subjects Search modes - Boolean/Phrase	242
S15	TI (distribut* governance) OR AB (distribut* governance)	Expanders - Apply equivalent subjects Search modes - Boolean/Phrase	2
S16	TI (distribut* framework OR distribut* frameworks OR distribut* frame work OR distribut* frame works)) OR AB (distribut* framework OR distribut* frameworks OR distribut* frame work OR distribut* frame works))	Expanders - Apply equivalent subjects Search modes - Boolean/Phrase	5
S17	TI (share leader* OR shares leader* OR shared leader* OR sharing leader*) OR AB (share leader* OR shares leader* OR shared leader* OR sharing leader*)	Expanders - Apply equivalent subjects Search modes - Boolean/Phrase	233
S18	TI (share education OR shares education OR shared education OR sharing education) OR AB (share education OR shares education OR shared education OR sharing education)	Expanders - Apply equivalent subjects Search modes - Boolean/Phrase	48
S19	(MH "Shared Governance") OR (MH "Shared Governance, Nursing")	Expanders - Apply equivalent subjects Search modes - Boolean/Phrase	1,691
S20	TI (share governance OR shares governance OR shared governance OR governance sharing) OR AB (share governance OR shares governance OR shared governance OR governance sharing)	Expanders - Apply equivalent subjects Search modes - Boolean/Phrase	821
S21	TI ((share responsibilit* OR shares responsibilit* OR shared responsibilit* OR responsibilit* sharing) OR AB ((share responsibilit* OR shares responsibilit* OR shared responsibilit* OR responsibilit* sharing)	Expanders - Apply equivalent subjects Search modes - Boolean/Phrase	936

S22	TI (participative governance OR participatory governance OR participative management) OR AB (participative governance OR participatory governance OR participative management)	Expanders - Apply equivalent subjects<br>Search modes - Boolean/Phrase	121
S23	S11 OR S12 OR S13 OR S14 OR S15 OR S16 OR S17 OR S18 OR S19 OR S20 OR S21 OR S22	Expanders - Apply equivalent subjects<br>Search modes - Boolean/Phrase	3,584
S24	S10 AND S23	Expanders - Apply equivalent subjects<br>Search modes - Boolean/Phrase	1,564
S25	S10 AND S23	Limiters - English Language<br>Expanders - Apply equivalent subjects<br>Search modes - Boolean/Phrase	1,526
S26	S10 AND S23	Limiters - Published Date: 20170101-20221231; English Language<br>Expanders - Apply equivalent subjects<br>Search modes - Boolean/Phrase	326
S27	S10 AND S23	Limiters - Published Date: 20170101-20221231; English Language; Peer Reviewed<br>Expanders - Apply equivalent subjects<br>Search modes - Boolean/Phrase	302

Database: IEEE Xplore
Search Strategy:

("All Metadata":distribut* NEAR/1 leader* OR "All Metadata":distribut* NEAR/1 education OR "All Metadata":distribut* NEAR/1 learning) OR ("All Metadata":distribut* NEAR/1 model) OR ("All Metadata":distribut* NEAR/1 governance) OR ("All Metadata":distribut* NEAR/1 framework OR "All Metadata":distribut* NEAR/1 frame work) OR ("All Metadata":share NEAR/1 leader* OR "All Metadata":sharing NEAR/1 leader*) OR ("All Metadata":share NEAR/1 education OR "All Metadata":sharing NEAR/1 education) OR ("All Metadata":share NEAR/1 governance OR "All Metadata":sharing NEAR/1 governance) OR ("All Metadata":share NEAR/1 responsibilit* OR "All Metadata":sharing NEAR/1 responsibilit*) OR ("All Metadata":participative governance OR "All Metadata":participatory governance OR "All Metadata":participative management)

Search Within Results

nurse OR nurses OR nursing

Filters Applied:

Journals

2017 - 2023

Indirect Search for Recommendation Question #5: Should a distributive model (versus no distributive model or any other type of change management model) be recommended to integrate digital health competencies into the professional practice roles and responsibilities of nurses at all levels within an organization?

Dates searched: January 1, 2017 – January 18, 2023

Databases searched: MEDLINE, Embase and CINAHL

**Note: The original search strategy was broadened to examine distributive models in general (not specific to digital health technologies), and all health providers (not just nurses). The search was only applied to MEDLINE, Embase, and CINAHL for feasibility purposes, and limited to systematic reviews in order to obtain the highest quality evidence.*

Database: MEDLINE
Search Strategy:

#	Searches	Results
1	exp "Attitude of Health Personnel"/	168824
2	exp Health Personnel/	600105
3	exp Personnel, Hospital/	94920
4	exp Health Occupations/	1835598
5	exp Patient Care Team/	72522
6	Social Work/	16186
7	Social Workers/	1047
8	Nurse's Role/	42659
9	Licensed Practical Nurses/	97
10	exp Nurses/	97022
11	exp Nursing Staff/	69514
12	Nursing Staff, Hospital/	47719
13	exp Nursing/	262430
14	Nursing, Practical/	3444
15	Health Educators/	489
16	exp Administrative Personnel/	42136
17	(health* adj2 (provider? or staff? or personnel? or employe* or profession* or occupation? or practitioner? or worker?)).tw,kf.	313758
18	(hospital* adj2 (provider? or staff? or personnel? or employe* or profession* or occupation? or practitioner? or worker?)).tw,kf.	21094

19	nurs*.tw,kf.	522469
20	(patient care? adj2 team?).tw,kf.	1017
21	(health* adj2 educator?).tw,kf.	3773
22	(social adj2 worker?).tw,kf.	12502
23	(support* adj2 (worker? or staff?)).tw,kf.	8033
24	clinician?.tw,kf.	286709
25	doctor?.tw,kf.	142096
26	physician?.tw,kf.	449321
27	practitioner?.tw,kf.	175433
28	surgeon?.tw,kf.	242164
29	Caregivers/	48351
30	(caregiver? or care-giver? or carer or carers).tw,kf.	105042
31	or/1-30 [Health Care Personnel]	3621026
32	(distribut* adj1 leader*).tw,kf.	154
33	(distribut* adj1 education).tw,kf.	112
34	(distribut* adj1 learning).tw,kf.	377
35	(distribut* adj1 (model or models)).tw,kf.	4552
36	(distribut* adj1 governance).tw,kf.	8
37	(distribut* adj1 (framework or frameworks or frame work or frame works)).tw,kf.	91
38	((share or shares or shared or sharing) adj1 leader*).tw,kf.	387
39	((share or shares or shared or sharing) adj1 education).tw,kf.	144
40	Shared Governance, Nursing/	14
41	((share or shares or shared or sharing) adj1 governance).tw,kf.	583
42	((share or shares or shared or sharing) adj1 responsibilit*).tw,kf.	1893
43	(participative governance or participatory governance or participative management).tw,kf.	214
44	(collectiv* adj1 leadership).tw,kf.	74
45	(change management adj2 model*).tw,kf.	57
46	Leadership/	46802
47	or/32-46	54871
48	31 and 47	32715
49	review/	3104055
50	(medline or medlars or pubmed or grateful med or CINAHL or scisearch or psychinfo or psycinfo or psychlit or psyclit or handsearch* or hand search* or manual* search* or electronic database* or bibliographic database* or embase or lilacs or scopus or "web of science").mp.	365778
51	49 and 50	193635
52	meta-analysis.mp.	264797
53	meta-analysis as topic/	22052
54	meta-analysis/	174052
55	systematic review*.tw.	271219

56	cochrane database*.jn.	16107
57	or/51-56	499616
58	48 and 57	455
59	limit 58 to english language	443
60	limit 59 to yr="2017 -Current"	271

Database: Embase
Search Strategy:

#	Searches	Results
1	exp health personnel attitude/	204684
2	exp health care personnel/	1960480
3	exp hospital personnel/	149229
4	exp multidisciplinary team/	24102
5	social work/	30207
6	social worker/	16337
7	licensed practical nurse/	379
8	exp nurse/	217453
9	nursing staff/	77610
10	exp nursing/	415892
11	practical nursing/	168
12	exp health educator/	3421
13	exp administrative personnel/	34910
14	nurse attitude/	43333
15	(health* adj2 (provider? or staff? or personnel? or employe* or profession* or occupation? or practitioner? or worker?)).tw,kw.	401307
16	(hospital* adj2 (provider? or staff? or personnel? or employe* or profession* or occupation? or practitioner? or worker?)).tw,kw.	29089
17	nurs*.tw,kw.	627916
18	(patient care? adj2 team?).tw,kw.	864
19	(health* adj2 educator?).tw,kw.	4352
20	(social adj2 worker?).tw,kw.	21207
21	(support* adj2 (worker? or staff?)).tw,kw.	11385
22	clinician?.tw,kw.	410399
23	doctor?.tw,kw.	212115
24	physician?.tw,kw.	656605
25	practitioner?.tw,kw.	226602
26	surgeon?.tw,kw.	381996
27	caregiver/	106513
28	(caregiver? or care-giver? or carer or carers).tw,kw.	147849
29	or/1-28	3766723

30	(distribut* adj1 leader*).tw,kw.	179
31	(distribut* adj1 education).tw,kw.	186
32	(distribut* adj1 learning).tw,kw.	392
33	(distribut* adj1 (model or models)).tw,kw.	4491
34	(distribut* adj1 governance).tw,kw.	13
35	(distribut* adj1 (framework or frameworks or frame work or frame works)).tw,kw.	102
36	((share or shares or shared or sharing) adj1 leader*).tw,kw.	405
37	((share or shares or shared or sharing) adj1 education).tw,kw.	219
38	nursing shared governance/	5
39	((share or shares or shared or sharing) adj1 governance).tw,kw.	605
40	((share or shares or shared or sharing) adj1 responsibilit*).tw,kw.	2305
41	participatory management/	1123
42	(participative governance or participatory governance or participative management).tw,kw.	250
43	(collectiv* adj1 leadership).tw,kw.	128
44	(change management adj2 model*).tw,kw.	67
45	leadership/	87619
46	or/30-45	96969
47	29 and 46	52069
48	"review"/	2875530
49	(medline or medlars or pubmed or grateful med or CINAHL or scisearch or psychinfo or psycinfo or psychlit or psyclit or handsearch* or hand search* or manual* search* or electronic database* or bibliographic database* or embase or lilacs or scopus or web of science).mp.	460680
50	48 and 49	185839
51	meta-analysis.mp.	389331
52	"meta analysis (topic)"/	51673
53	meta analysis/	268683
54	systematic review*.tw.	328491
55	cochrane database*.jn.	22847
56	or/50-55	638357
57	47 and 56	764
58	limit 57 to english language	752
59	limit 58 to yr="2017 -Current"	515
60	limit 59 to (books or chapter or conference abstract or conference paper or "conference review")	89
61	59 not 60	426

Database: CINAHL
Search Strategy:

#	Query	Limiters/Expanders	Results
S1	(MH "Attitude of Health Personnel+")	Expanders - Apply equivalent subjects Search modes - Boolean/Phrase	122,237
S2	(MH "Health Personnel+")	Expanders - Apply equivalent subjects Search modes - Boolean/Phrase	638,588
S3	(MH "Personnel, Health Facility+")	Expanders - Apply equivalent subjects Search modes - Boolean/Phrase	44,698
S4	(MH "Health Occupations+")	Expanders - Apply equivalent subjects Search modes - Boolean/Phrase	826,110
S5	(MH "Multidisciplinary Care Team+")	Expanders - Apply equivalent subjects Search modes - Boolean/Phrase	51,724
S6	(MH "Social Work+")	Expanders - Apply equivalent subjects Search modes - Boolean/Phrase	15,313
S7	(MH "Social Workers")	Expanders - Apply equivalent subjects Search modes - Boolean/Phrase	11,149
S8	(MH "Nursing Role")	Expanders - Apply equivalent subjects Search modes - Boolean/Phrase	65,568
S9	(MH "Practical Nurses") OR (MH "Practical Nursing")	Expanders - Apply equivalent subjects Search modes - Boolean/Phrase	7,720
S10	(MH "Nurses+")	Expanders - Apply equivalent subjects Search modes - Boolean/Phrase	242,123
S11	(MH "Nursing Staff, Hospital")	Expanders - Apply equivalent subjects Search modes - Boolean/Phrase	25,026
S12	(MH "Health Educators+")	Expanders - Apply equivalent subjects Search modes - Boolean/Phrase	4,211
S13	(MH "Administrative Personnel+")	Expanders - Apply equivalent subjects Search modes - Boolean/Phrase	40,664
S14	TI (health* N2 provider#) OR AB (health* N2 provider#)	Expanders - Apply equivalent subjects Search modes - Boolean/Phrase	57,522
S15	TI (hospital* N2 provider#) OR AB (hospital* N2 provider#)	Expanders - Apply equivalent subjects Search modes - Boolean/Phrase	1,823
S16	TI (health* N2 staff#) OR AB (health* N2 staff#)	Expanders - Apply equivalent subjects Search modes - Boolean/Phrase	8,934
S17	TI (hospital* N2 staff#) OR AB (hospital* N2 staff#)	Expanders - Apply equivalent subjects Search modes - Boolean/Phrase	6,050
S18	TI (health* N2 personnel#) OR AB (health* N2 personnel#)	Expanders - Apply equivalent subjects Search modes - Boolean/Phrase	5,430

S19	TI (hospital* N2 personnel#) OR AB (hospital* N2 personnel#)	Expanders - Apply equivalent subjects Search modes - Boolean/Phrase	1,155
S20	TI (health* N2 employe*) OR AB (health* N2 employe*)	Expanders - Apply equivalent subjects Search modes - Boolean/Phrase	7,113
S21	TI (hospital* N2 employe*) OR AB (hospital* N2 employe*)	Expanders - Apply equivalent subjects Search modes - Boolean/Phrase	2,067
S22	TI (health* N2 profession*) OR AB (health* N2 profession*)	Expanders - Apply equivalent subjects Search modes - Boolean/Phrase	100,605
S23	TI (hospital* N2 profession*) OR AB (hospital* N2 profession*)	Expanders - Apply equivalent subjects Search modes - Boolean/Phrase	1,982
S24	TI (health* N2 occupation#) OR AB (health* N2 occupation#)	Expanders - Apply equivalent subjects Search modes - Boolean/Phrase	1,014
S25	TI (hospital* N2 occupation#) OR AB (hospital* N2 occupation#)	Expanders - Apply equivalent subjects Search modes - Boolean/Phrase	58
S26	TI (health* N2 practitioner#) OR AB (health* N2 practitioner#)	Expanders - Apply equivalent subjects Search modes - Boolean/Phrase	12,158
S27	TI (hospital* N2 practitioner#) OR AB (hospital* N2 practitioner#)	Expanders - Apply equivalent subjects Search modes - Boolean/Phrase	925
S28	TI (health* N2 worker#) OR AB (health* N2 worker#)	Expanders - Apply equivalent subjects Search modes - Boolean/Phrase	33,998
S29	TI (hospital* N2 worker#) OR AB (hospital* N2 worker#)	Expanders - Apply equivalent subjects Search modes - Boolean/Phrase	1,594
S30	TI nurs* OR AB nurs*	Expanders - Apply equivalent subjects Search modes - Boolean/Phrase	640,663
S31	TI (patient care# N2 team#) OR AB (patient care# N2 team#)	Expanders - Apply equivalent subjects Search modes - Boolean/Phrase	588
S32	TI (health* N2 educator#) OR AB (health* N2 educator#)	Expanders - Apply equivalent subjects Search modes - Boolean/Phrase	3,756
S33	TI (social N2 worker#) OR AB (social N2 worker#)	Expanders - Apply equivalent subjects Search modes - Boolean/Phrase	14,796
S34	TI (support* N2 (worker# or staff#)) OR AB (support* N2 (worker# or staff#))	Expanders - Apply equivalent subjects Search modes - Boolean/Phrase	9,616
S35	TI clinician# OR AB clinician#	Expanders - Apply equivalent subjects Search modes - Boolean/Phrase	122,996
S36	TI doctor# OR AB doctor#	Expanders - Apply equivalent subjects Search modes - Boolean/Phrase	65,335
S37	TI physician# OR AB physician#	Expanders - Apply equivalent subjects Search modes - Boolean/Phrase	169,229

S38	TI practitioner# OR AB practitioner#	Expanders - Apply equivalent subjects Search modes - Boolean/Phrase	107,730
S39	TI surgeon# OR AB surgeon#	Expanders - Apply equivalent subjects Search modes - Boolean/Phrase	63,346
S40	(MH "Caregivers")	Expanders - Apply equivalent subjects Search modes - Boolean/Phrase	42,829
S41	TI (caregiver# or care-giver# or carer or carers) OR AB (caregiver# or care-giver# or carer or carers)	Expanders - Apply equivalent subjects Search modes - Boolean/Phrase	79,419
S42	S1 OR S2 OR S3 OR S4 OR S5 OR S6 OR S7 OR S8 OR S9 OR S10 OR S11 OR S12 OR S13 OR S14 OR S15 OR S16 OR S17 OR S18 OR S19 OR S20 OR S21 OR S22 OR S23 OR S24 OR S25 OR S26 OR S27 OR S28 OR S29 OR S30 OR S31 OR S32 OR S33 OR S34 OR S35 OR S36 OR S37 OR S38 OR S39 OR S40 OR S41	Expanders - Apply equivalent subjects Search modes - Boolean/Phrase	2,193,996
S43	TI (distribut* leader*) OR AB (distribut* leader*)	Expanders - Apply equivalent subjects Search modes - Boolean/Phrase	104
S44	TI (distribut* education) OR AB (distribut* education)	Expanders - Apply equivalent subjects Search modes - Boolean/Phrase	25
S45	TI (distribut* learning) OR AB (distribut* learning)	Expanders - Apply equivalent subjects Search modes - Boolean/Phrase	92
S46	TI (distribut* model OR distribut* models) OR AB (distribut* model OR distribut* models)	Expanders - Apply equivalent subjects Search modes - Boolean/Phrase	261
S47	TI (distribut* governance) OR AB (distribut* governance)	Expanders - Apply equivalent subjects Search modes - Boolean/Phrase	2
S48	TI (distribut* framework OR distribut* frameworks OR distribut* frame work OR distribut* frame works)) OR AB (distribut* framework OR distribut* frameworks OR distribut* frame work OR distribut* frame works))	Expanders - Apply equivalent subjects Search modes - Boolean/Phrase	5
S49	TI (share leader* OR shares leader* OR shared leader* OR sharing leader*) OR AB (share leader* OR shares leader* OR shared leader* OR sharing leader*)	Expanders - Apply equivalent subjects Search modes - Boolean/Phrase	250
S50	TI (share education OR shares education OR shared education OR sharing education) OR AB (share education OR	Expanders - Apply equivalent subjects Search modes - Boolean/Phrase	52

	shares education OR shared education OR sharing education)		
S51	(MH "Shared Governance") OR (MH "Shared Governance, Nursing") OR (MH "Leadership")	Expanders - Apply equivalent subjects<br>Search modes - Boolean/Phrase	52,191
S52	TI (share governance OR shares governance OR shared governance OR governance sharing) OR AB (share governance OR shares governance OR shared governance OR governance sharing)	Expanders - Apply equivalent subjects<br>Search modes - Boolean/Phrase	855
S53	TI ((share responsibilit* OR shares responsibilit* OR shared responsibilit* OR responsibilit* sharing) OR AB ((share responsibilit* OR shares responsibilit* OR shared responsibilit* OR responsibilit* sharing)	Expanders - Apply equivalent subjects<br>Search modes - Boolean/Phrase	1,001
S54	TI (participative governance OR participatory governance OR participative management) OR AB (participative governance OR participatory governance OR participative management)	Expanders - Apply equivalent subjects<br>Search modes - Boolean/Phrase	144
S55	S43 OR S44 OR S45 OR S46 OR S47 OR S48 OR S49 OR S50 OR S51 OR S52 OR S53 OR S54	Expanders - Apply equivalent subjects<br>Search modes - Boolean/Phrase	53,987
S56	S42 AND S55	Expanders - Apply equivalent subjects<br>Search modes - Boolean/Phrase	34,574
S57	(MH "Literature Review")	Expanders - Apply equivalent subjects<br>Search modes - Boolean/Phrase	9,004
S58	TX (medline OR medlars OR pubmed OR grateful med OR CINAHL OR scisearch OR psychinfo OR psycinfo OR psychlit OR psyclit OR handsearch* OR "hand search*" OR "manual* search*" OR "electronic database*" OR "bibliographic database*" OR embase OR lilacs OR scopus OR "web of science")	Expanders - Apply equivalent subjects<br>Search modes - Boolean/Phrase	271,797
S59	S57 AND S58	Expanders - Apply equivalent subjects<br>Search modes - Boolean/Phrase	2,396
S60	TI ("meta-analysis") OR AB ("meta-analysis")	Expanders - Apply equivalent subjects<br>Search modes - Boolean/Phrase	89,770

S61	(MH "Meta Analysis")	Expanders - Apply equivalent subjects<br>Search modes - Boolean/Phrase	68,272
S62	TI (systematic review*) OR AB (systematic review*)	Expanders - Apply equivalent subjects<br>Search modes - Boolean/Phrase	136,321
S63	JN cochrane database*	Expanders - Apply equivalent subjects<br>Search modes - Boolean/Phrase	6,023
S64	S59 OR S60 OR S61 OR S62 OR S63	Expanders - Apply equivalent subjects<br>Search modes - Boolean/Phrase	186,110
S65	S56 AND S64	Expanders - Apply equivalent subjects<br>Search modes - Boolean/Phrase	229
S66	S56 AND S64	Limiters - English Language<br>Expanders - Apply equivalent subjects<br>Search modes - Boolean/Phrase	221
S67	S56 AND S64	Limiters - Published Date: 20170101-20231231; English Language<br>Expanders - Apply equivalent subjects<br>Search modes - Boolean/Phrase	146
