# Supplementary material for: Developing a Best Practice Guideline for Clinical Practice in a Digital Health Environment: Systematic Reviews Based on the Grading of Recommendations, Assessment, Development, and Evaluation Approach
Source: JMIR Nurs. 2026 Jan 23;9:e74942. doi: 10.2196/74942 (PMC12829893; doi:10.2196/74942)
Supplement: Multimedia Appendix 4 [file nursing-v9-e74942-s004.pdf]

Multimedia Appendix: Recommendation 1.0 Evidence Profile

Recommendation question: Should practical (e.g., hands-on) professional development education focused on the use of digital health technologies within an organization be recommended or not for all nurses?

Recommendation 1.0: The expert panel suggests that health service and academic organizations provide education to nurses and health providers that includes hands-on training for the use of digital health technologies.

Population: All nurses and other health providers (including students entering health professions), and persons receiving care
Intervention: Practical (e.g., hands-on) professional development education (in general, or specific to digital health technologies)
Comparison: Standard education (i.e., no practical component)
Outcomes: Nurse competence [with using technology] (critical)**,** nurse confidence [with using technology] (critical)**,** nurse-person therapeutic relationship (critical), nurse acceptance of technology (critical; not measured)**,** nurse sensitive outcomes (falls, pressure injuries, pain) (critical; not measured)**,** nurse involvement in the technology lifecycle (critical; not measured)**,**

Setting: All practice settings where nurses provide care to persons using digital health technologies (e.g., primary care, community care, acute care, and long-term care)

Bibliography: 1511, 118, 238, 203

Quality assessment							No. of participants		Reported effects/outcomes	Certainty	Reference
№ of studies	Study design	Risk of bias	Inconsistency	Indirectness	Imprecision	Publication Bias	Intervention	Control			
**Nurse competence [with using technology]** *(Measured using a variety of skills instruments)*											
13[a]	RCTs	Very Serious[b]	Not serious[c]	Serious[d]	Not Serious[e]	Undetected			Both systematic reviews demonstrated overall that practical education (e.g., simulation) improved nurse competence compared to standard education.	⊕◯◯◯<br>Very low	
							Simulation training n=164 participants	Other learning strategies<br><br>n=155 participants	Six studies in the meta-analysis demonstrated a large effect in favour of simulation over other learning strategies.[f]<br><br>SMD: −1.09 (CI −1.72 to −0.47)		1511: Hegland et al., 2017
							Virtual reality training for nursing students N=408	Traditional learning programs for nursing students N=408	Seven studies in the review demonstrated that virtual reality technology training moderately enhances nursing students' practical skills, and largely enhances critical thinking compared to traditional education among nursing students.<br><br>**Practical skills**: 4 studies looked at this outcome (SMD=0.52, 95% CI [0.33, 0.71])<br><br>**Critical thinking:** 4 studies looked at this outcome (SMD=0.8, 95% CI [0.15, 1.44])		203: Liu et al., 2023
**Nursing confidence [with using technology]** *(Measured using a variety of self-reported confidence scales)*											
47[g]	RCTs and non-	Very serious[h]	Serious[i]	Not serious	Not serious[j]	Undetected	Simulation-based training (n=1673 participants)	Conventional teaching strategies or	The meta-analysis demonstrated that simulation showed a moderate effect on confidence favouring simulation when compared to other teaching strategies.	⊕◯◯◯<br>Very low	118: Oliveira Silva et al., 2022

Quality assessment							No. of participants		Reported effects/outcomes	Certainty	Reference
№ of studies	Study design	Risk of bias	Inconsistency	Indirectness	Imprecision	Publication Bias	Intervention	Control			
	randomized studies							no intervention or comparator (n=1690 participants)	SMD: 0.71 (95% CI 0.47 to 0.96)		
**Nurse-person therapeutic relationship** (*Measured as caring using a variety of questionnaires)*											
4[k]	RCTs and non-randomized studies	Very serious[l]	Not serious[m]	Very Serious[n]	Serious[o]	None	High-fidelity simulation training (HFS) (n=287 participants)	Other teaching methods (n=281 participants)	The meta-analysis reported that a HFS learning environment fostered a large increase in nursing students' caring compared to other teaching methods.<br><br>SMD 1.40 (95% CI 0.23 to 2.58)	⊕◯◯◯<br>Very low	238: Li et al., 2022
**Nurse acceptance of technology** *(Not measured)*											
N/A											
**Nurse sensitive outcomes (falls, pressure injuries, pain)** *(Not measured)*											
N/A											
**Nurse involvement in the technology lifecycle** *(Not measured)*											
N/A											

Additional Table – Individual Study Details

Reference	Study Design	Country	Intervention Group Details	Control Group Details	Reported Effects/Outcomes	Risk of Bias
**Outcome: Nurse competence [with using technology]**						
Cioffi et al. (2005); Hebbar et al. (2015); Johnson et al. (2012); Keleekai et al. (2016); Rutherford-Hemming et al. (2016); Weiner et al. (2011)	Systematic review and meta-analysis of 6 RCTs	Australia & USA	Simulation-based learning strategies for midwifery students, Registered Nurses, or anaesthesia students, including low-fidelity simulation sessions, high-fidelity simulation with an advanced manikin,	Other learning strategies (i.e., not hands-on), including lectures or didactic training, standard teaching, or online self-study modules.<br><br>n=155	Six studies in the meta-analysis demonstrated a large effect in favour of simulation over other learning strategies.[f]<br><br>SMD: −1.09 (CI −1.72 to −0.47)	Systematic review: LOW<br><br>Individual studies: SERIOUS

*From review 1511 (Hegland et al., 2017)			and actors trained as standardized patients.<br><br>n=164			
4 studies assessed practical skills outcome: Nan Cao (2021); PingWang (2020); Tianxiang Yuan (2019); Xiaoyan Wang (2023)<br><br>4 studies assessed critical thinking outcome: Hanna Lee (2022); Hongmei Zhao (2022); Nan Cao (2021); Xiaoyan Wang (2023)<br><br>*From review 203 (Liu et al., 2023)	Systematic review and meta-analysis of 7 RCTs	China, Korea	The intervention group was comprised of nursing students who were offered education using virtual reality.<br><br>N = 408 (for all studies)	Control group participants were nursing students that were offered traditional teaching opportunities with no virtual reality component. One study used non-immersive VR as the control group (Ping Wang 2020).<br><br>N= 408	**Practical skills:** 4 RCTs included in this meta-analysis examined this outcome, and demonstrated that those who received virtual reality education showed an increase in practical skills compared to those who were offered traditional teaching methods. (SMD=0.52, 95% CI [0.33, 0.71], P <0.001) I^2=10%<br><br>**Critical thinking:** 4 RCTs included in this meta-analysis examined this outcome and found that VR technology compared to the control teaching modality improved critical thinking skills (SMD =0.8 95% CI [0.15, 1.44], I^2=90%).	Systematic Review: Low<br><br>Individual studies: VERY SERIOUS
**Outcome: Nursing confidence [with using technology]**						
Bowling & Underwood (2016); Warren (2015); Blum, Borglund & Parcells (2010); Curtis (2014); Kim & Kim (2015); Senturk Erenel et al. (2021); Topbas et al. (2018); Merriman, Sayt & Ricketts (2014); Choi et al. (2020); AlAmrani et al. (2017); Sanko & Mckay (2017a); DiGiacomo (2017); Stayt et al. (2015); Akalin & Sahin (2020); Tuttle (2009); Valizadeh et al. (2013a); Ahn & Kim (2015a); Branna, White & Bezanson (2008); Huse (2010); Shinnick & Woo (2014); Terzi et al. (2019a); Rivers (2012); Alfes (2011); Ahn & Kim (2015b); Kim, Issenberg & Roh (2020); Liaw et al. (2019); Akhu-Zaheya, Gharaibeh & Alostaz (2013); Mager & Campbell (2013); Ravert (2004); Luebbert & Popkess (2015); Valizadeh et al. (2013b); Lee et al. (2016); Seo & Eom (2021); Basak, Demirtas & Iyigun (2019); Thomas & Mackey (2012); Younghee (2015); Sanko & Mckay	Systematic review and meta-analysis of 47 RCTs and non-randomized studies	Turkey, Saudi Arabia, Brazil, Oman, Norway, Singapore, USA, Jordan, Iran, South Korea	Undergraduate nursing students in any period of their program receiving simulation-based training.<br><br>n=1673	Conventional teaching strategies or no intervention or comparator.<br><br>n=1690	The meta-analysis demonstrated that simulation showed a moderate effect on confidence favouring simulation when compared to other teaching strategies.<br><br>SMD 0.71 (95% CI 0.47 to 0.96)	Systematic review: LOW<br><br>Individual studies: VERY SERIOUS

(2017b); D'Souza et al. (2020); Tan et al. (2017); Abu Sharour (2019b); Abu Sharour (2019a); Tawalbeh & Tubaishat (2014); Tawalbeh (2020).<br><br>*From review 118 (Silva et al., 2022)						
**Outcome: Nurse-person therapeutic relationship**						
Li & Li (2019); Liu et al. (2015); Liu et al. (2020); Wang & Xu (2020)<br><br>*From review 238 (Li et al., 2022)	Systematic review and meta-analysis of 2 RCTs and 2 non-randomized studies	China	Undergraduate nursing students participating in high-fidelity simulation training.<br><br>n=287	Other teaching methods including low-fidelity simulation, case studies, and standardized patients.<br><br>n=281	The meta-analysis reported that a HFS learning environment fostered a large increase in nursing students' caring compared to other teaching methods.<br>SMD 1.40 (95% CI 0.23 to 2.58)	Systematic review: LOW<br><br>Individual studies: VERY SERIOUS

Acronyms
CI = Confidence interval
HFS = high-fidelity simulation
RCT = randomized controlled trial
SMD = standardized mean difference
SR = systematic review
VR = virtual reality

Tools used to measure outcomes
Study 1511: variety of skills instruments including: a self-developed master sheet assessing participants' performance, a 17-point central venous line dressing change checklist, a 120 criteria clinical practice instrument, a 28-item peripheral intravenous catheter insertion skills checklist, The Performance Observation Measurement Tool, and computer data from a manikin.
Study 203: specific tools used to measure practical skills and critical thinking skills were not specified in the review.
Study 118: variety of self-reported confidence scales including: California Critical Thinking Disposition Inventory, Confidence Scale (C-Scale), Student Satisfaction and Self-Confidence in Learning Scale (SSSCL), Knowledge and self-confidence questionnaire (40 items), Self-confidence scale (SCS), Mental Health Nursing Clinical Confidence Scale (MHNCCS), Emergency Response Confidence tool, Heart and Lung Assessment Confidence Scale, Clinical decision-making self-confidence scale, Clinical Decision-Making Self-Confidence Scale, Self-confidence evaluation questionnaire [Prepared and validated by the authors], Self-Confidence Emergency Response Tool (modified version), Self-confidence assessment questionnaire (validated by the authors based on the Lasater Clinical Judgment Rubric), Confidence Level tool (CL), Medication Administration Competence and Confidence Scale, Clinical Self-Confidence Scale, Instrument to assess Confidence and Stress (20 items, developed by the authors), Self-confidence assessment instrument (11 items, prepared by the authors), and Nursing Anxiety and Self-Confidence with Clinical Decision Making (NASC-CDM).
Study 238: Measured as caring using a variety of questionnaires: Caring self-designed questionnaires, and The Nurses Humanistic Care Quality Questionnaire.

Explanations

---

[a] 13 RCTs were included from a systematic review and meta-analysis. 6 RCTs were from Hegland et al., 2017, and 7 RCTs were from Liu et al. (2023).
[b] The reviews were assessed using the ROBIS tool for systematic reviews, and had a low risk of bias. Studies included in the reviews were assessed by the authors using the Cochrane ROB 2.0 tool for RCTs; 8 studies had high risk of bias, 4 studies had unclear risk of bias, and 1 study had low risk of bias; there were concerns noted around allocation concealment, blinding, and incomplete outcome data. We downgraded by 2.
[c] All studies demonstrated a positive direction of effect, however there was high heterogeneity across the studies. We downgraded by 0.5.
[d] The outcomes of 'skills', 'practical skills', and 'critical thinking skills' were slightly different from the original outcome of interest (nurse competence). One study included in Liu et al. (2023) had a slightly different comparator (non-immersive virtual reality). We downgraded by 0.5. Although the intervention didn't focus on practical education on the use of digital health technologies specifically, it was decided that the intervention of 'simulation education' was close enough to the original intervention of interest (i.e. practical professional development education) and there was not enough concern to warrant downgrading further.

[e] The total number of participants was 1135 across both reviews. We did not downgrade.
[f] The review authors didn't specify which is the control group, but based on a negative SMD, and the context in favor of simulation strategies demonstrated in the forest plot, it was determined that the authors assumed the control in this study as ‘simulation based training’ and the intervention group was ‘other strategies’.
[g] 47 RCTs and non-randomized studies were included from a systematic review and meta-analysis (Silva et al., 2022).
[h] The review was assessed using the ROBIS tool for systematic reviews, and had a low risk of bias. Studies included in the review were assessed by the authors using the Cochrane ROB 2.0 tool for RCTs and the ROBINS-I tool for non-randomized studies; 10 studies had a high ROB and 9 studies had a critical ROB; there were concerns noted around missing outcome data, selection of the reported results, confounding, and selection of participants. We downgraded by 2.
[i] There was variability in the direction of effect shown in the studies; most studies demonstrated a positive direction of effect, but some demonstrated no effect. There was high heterogeneity across the studies (I^2=85%). We downgraded by 1.
[j] The total number of participants was greater than the optimal 800 participants (n=3363). We did not downgrade.
[k] Two RCTs and two non-randomized studies were included from a systematic review and meta-analysis (Li et al., 2022).
[l] The review was assessed using the ROBIS tool for systematic reviews, and had a low risk of bias. Studies included in the review were assessed by the authors using the NICE quality appraisal checklist; there were concerns noted around confounding, allocation concealment, blinding, and power. We downgraded by 2.
[m] All studies demonstrated a positive direction of effect, however there was high heterogeneity across the studies (I^2=97%). We downgraded by 0.5.
[n] The outcome of ‘caring’ was slightly different from the original outcome of interest (nurse-person therapeutic relationship). The comparator was different than the original comparison of interest (included other types of simulation strategies). We downgraded by 2.
[o] The total number of participants was less than the optimal 800 participants (n=568). We downgraded by 1.

References

1. Hegland, P. A., Aarlie, H., Strømme, H., & Jamtvedt, G. (2017). Simulation-based training for nurses: Systematic review and meta-analysis. *Nurse education today*, *54*, 6–20. https://doi.org/10.1016/j.nedt.2017.04.004
2. Liu, K., Zhang, W., Li, W. et al. (2023). Effectiveness of virtual reality in nursing education: a systematic review and meta-analysis. *BMC Med Educ,* 23, 710. https://doi.org/10.1186/s12909-023-04662-x
3. Li, Y. Y., Au, M. L., Tong, L. K., Ng, W. I., & Wang, S. C. (2022). High-fidelity simulation in undergraduate nursing education: A meta-analysis. *Nurse education today*, *111*, 105291. https://doi.org/10.1016/j.nedt.2022.105291
4. Oliveira Silva, G., Oliveira, F. S. E., Coelho, A. S. G., Cavalcante, A. M. R. Z., Vieira, F. V. M., Fonseca, L. M. M., Campbell, S. H., & Aredes, N. D. A. (2022). Effect of simulation on stress, anxiety, and self-confidence in nursing students: Systematic review with meta-analysis and meta-regression. *International journal of nursing studies*, *133*, 104282. https://doi.org/10.1016/j.ijnurstu.2022.104282
