# Supplementary material for: Developing a Best Practice Guideline for Clinical Practice in a Digital Health Environment: Systematic Reviews Based on the Grading of Recommendations, Assessment, Development, and Evaluation Approach
Source: JMIR Nurs. 2026 Jan 23;9:e74942. doi: 10.2196/74942 (PMC12829893; doi:10.2196/74942)
Supplement: Multimedia Appendix 5 [file nursing-v9-e74942-s005.pdf]

Multimedia Appendix: Recommendation 2.0 Evidence Profile (Quantitative)

Recommendation question: Should education about relational care and interpersonal communication skills be recommended or not for nurses practicing in virtual care settings and in-person digital health environments?

Recommendation 2.0: The expert panel suggests that health-service and academic organizations provide ongoing education to nurses and health providers that focuses on interpersonal communication skills when using digital health technologies.

Population: All nurses and other health providers (including students entering health professions), and persons receiving care
Intervention: Comprehensive education about relational care and interpersonal communication skills (in general, or specific to digital health environments)
Comparison: Standard education (or no education) about relational care and interpersonal communication skills
Outcomes: Person/ Caregiver/ Family experience or satisfaction (critical)**,** nurse competence [with using technology] (critical), nurse confidence [with using technology] (critical), nurse -person therapeutic relationship (critical), person/ caregiver/ family involvement and engagement in care (critical; not measured)

Setting: All practice settings where nurses provide care to persons using digital health technologies (e.g., primary care, community care, acute care, long-term care, etc.)

Bibliography: 265, 1530, 1963, 2005, 2267, 2490, 2816

Quality assessment							No. of participants		Reported effects/outcomes	Certainty	Reference
**№ of studies**	**Study design**	**Risk of bias**	**Inconsistency**	**Indirectness**	**Imprecision**	**Publication bias**	**Intervention**	**Control**			
**Person/Caregiver/Family experience or satisfaction** *(Measured as information gathering about patient perspectives/concerns and satisfaction using a variety of scales)*											
5[a]	RCTs	Not serious[b]	Not serious[c]	Serious[d]	Serious[e]	Undetected	Interpersonal communication interventions (n=198 participants)	No intervention or usual training (n=207 participants)	After meta-analysis, the pooled effect size for 5 RCTs demonstrated largely improved information gathering about patient perspectives/ concerns, including largely improved satisfaction, in the intervention groups compared to control groups (SMD 1.07, 95% CI 0.61, 1.54).	⨁⨁◯◯ Low	265: Gilligan et al., 2021
**Nurse competence [with using technology]** *(measured as overall communication skills using a variety of scales)*											
18[f]	RCTs, cluster RCTs and quasi-cluster RCTs	Serious[g]	Serious[h]	Not serious[i]	Not serious[j]	Undetected	Interpersonal communication interventions (660 participants)	Interpersonal communication interventions (696 participants)	After meta-analysis, the pooled effect size for the 18 included studies showed a large increase in communication skills in favour of interpersonal communication interventions compared to no intervention or usual training (SMD 0.92, 95% CI 0.53, 1.31).	⨁⨁◯◯ Low	265: Gilligan et al., 2021

Quality assessment							No. of participants		Reported effects/outcomes	Certainty	Reference
№ of studies	Study design	Risk of bias	Inconsistency	Indirectness	Imprecision	Publication bias	Intervention	Control			
**Nurse confidence [with using technology]** *(measured using questionnaires developed by the researchers, and the SET-M in one study. One study measured comfort using technology instead of confidence)*											
5	Non-RCT	Very serious[k]	Not serious[l]	Not serious[m]	Very Serious[n]	Undetected	Interventions included educational training sessions to medical students and nursing students, using videos, interactive teaching stations, simulations, role playing with standardized patients, large groups sessions, and individualized teaching sessions. All studies focused on communications skills when using technology in practice (e.g., telehealth, virtual consults, or using an EHR during a patient consult).<br><br>.n=209 participants	There was no control group, and results were compared pre and post intervention (or post-only).	Five studies reported an improvement in confidence after participants received education about interpersonal communication skills and digital health technologies[o].	⊕◯◯◯ Very low	1530: Gunner et al., 2021; 2005: Riley et al., 2022; 2267: Mahabamunuge et al., 2021; 2490: Newcomb et al., 2020; 2816: Lanier et al., 2017
1	Non-RCT with historical control group	Very Serious[p]	Not Serious	Not Serious[q]	Very Serious[r]	Undetected	A 'patient-centred EHR use' curriculum designed for 2nd year medical students, consisting of a lecture and group OSCE.<br><br>N= 89 (80 completed survey)<br><br>How confident are you using the EHR in a patient-centered manner?<br>21/80 (26%) said moderately confident	3rd year students received no formal training and served as a historical control group by completing the same OSCE individually.<br>N=96 (88 completed survey)<br>How confident are you using the EHR in a patient-centered manner?<br>14/88 (16%) said moderately confident	More students were confidence using the EHR with formal training (compared to no formal training).	⊕◯◯◯ Very low	1963: Lee et al., 2017
**Nurse-person therapeutic relationship** *(measured as relationship building/rapport using a variety of scales)*											
9[s]	RCTs	Not serious[t]	Serious[u]	Not Serious	Not Serious[v]	Undetected	Interpersonal communication interventions (n=405 participants)	Interpersonal communication interventions (n=429 participants)	Communication skills interventions may have very little positive effect, or no effect, on relationship building scores when compared to the usual curriculum or control.<br>SMD 0.18 (95% CI -0.15, 0.51)	⊕⊕⊕◯ Moderate	265: Gilligan et al., 2021

Quality assessment							No. of participants		Reported effects/outcomes	Certainty	Reference
№ of studies	Study design	Risk of bias	Inconsistency	Indirectness	Imprecision	Publication bias	Intervention	Control			
**Person/caregiver/family involvement and engagement in care** *(Not measured)*											
N/A											

Additional Table – Individual Study Details

Reference	Study Design	Country	Intervention Group Details	Control Group Details	Reported Effects/Outcomes	Risk of Bias
**Outcome: Person/Caregiver/Family experience or satisfaction**						
Bosse (2012); Evans (1989); Ho (2008); Lee (2015); Schwartz (2010)<br><br>*From review 265 (Gilligan et al., 2021)	Systematic review and meta-analysis of 5 RCTs	Multiple: Germany, Australia, Taiwan, USA	Interventions that aim to improve medical students' interpersonal communication when undertaking medical consultations, including interventions targeting the communication tasks and skills associated with relationship building, information gathering, and planning and explaining, as well as specific tasks of communication such as listening, using appropriate non-verbal communication, and providing closure.<br><br>n=198	No intervention, or usual training.<br><br>n=207	After meta-analysis, the pooled effect size for 5 RCTs demonstrated largely improved information gathering about patient perspectives/ concerns, including largely improved satisfaction, in the intervention groups compared to control groups (SMD 1.07, 95% CI 0.61, 1.54).	Systematic review: LOW<br><br>Individual studies: NOT SERIOUS
**Outcome: Nurse competence [with using technology]**						
Bosse (2012); Evans (1996); Fillipetto (2006); Gartmeir (2015); Lee (2015); Liu (2016); Lorin (2006); Lupi (2012); Maguire (1977); Maguire (1978); Pirdehghan (2018); Solomon (2004); Spollen (2010); Colletti (2001); Ho (2008); Hobgood (2009); Shaddeau	Systematic review and meta-analysis of 18 RCTs, cluster RCTs and quasi-cluster RCTs	Multiple: Germany, Australia, USA, England, Iran, Taiwan, Canada	Interventions that aim to improve medical students' interpersonal communication when undertaking medical consultations, including interventions targeting the communication tasks and skills associated with relationship building, information gathering, and planning and explaining, as well as specific tasks of communication such as listening, using appropriate non-verbal communication, and providing closure.<br><br>n=660<br><br>**Subgroups:**<br>Assessed by experts (faculty or trained assessors): n=13 studies, 476 participants, SMD 1.21 (0.69, 1.74)<br><br>Assessed by standardized patients: n=5 studies, 184 participants, SMD 0.27 (95% CI -0.07, 0.60)	No intervention, or usual training.<br><br>n=696<br><br>**Subgroups:**<br>Assessed by experts (faculty or trained assessors): n=483 participants<br><br>Assessed by standardized patients: n=213 participants	After meta-analysis, the pooled effect size for the 18 included studies showed a large increase in communication skills in favour of interpersonal communication interventions compared to no intervention or usual training (SMD 0.92, 95% CI 0.53, 1.31).	Systematic review: LOW<br><br>Individual studies: SERIOUS

(2015);<br>Shapiro (2009)<br><br><br>*From review 265 (Gilligan et al., 2021)						
**Outcome: Nurse confidence [with using technology]**						
1530 (Gunner et al., 2021)	Non-RCT	United Kingdom	A two-hour training in video consultation skills for medical students was provided. Training was comprised of an introductory video and three interactive teaching stations focused on 1) setting up technology for a consultation, 2) practicing with a simulated patient, and 3) patient selection and ethics.<br><br>n=40 (but only 34 completed evaluation forms)<br><br>Mean (SD)<br><br>**Pre-session confidence:**<br>Define video consultation: 2.79 (0.76)<br>Identify suitable patients: 2.44 (0.77)<br>Describe consent process: 2.03 (0.89)<br>Describe technical and procedural issues arising during video consultation: 2.26 (0.70)<br>Describe key elements of safe/ effective video consultation: 2.26 (0.70)<br>Assess patient using video consultation: 2.32 (0.83)<br>Discuss ethical issues surrounding video consultation: 2.65 (0.76)<br><br>**Post-session confidence:**<br>Define video consultation: 4.26 (0.44)<br>Identify suitable patients: 4.03 (0.38)<br>Describe consent process: 4.41 (0.49)<br>Describe technical and procedural issues arising during video consultations: 4.24 (0.60)<br>Describe the key elements of safe/ effective video consultation: 4.24 (0.49)<br>Assess patient using video consultation: 3.97 (0.38)<br>Discuss ethical issues surrounding video consultation: 4.09 (0.45)	There was no control group, and results were compared pre and post intervention.	The mean increase in confidence ratings from pre- to post-session was 1.78. No student had a fall in confidence in any area at the end of the session.	CRITICAL
2005 (Riley et al., 2022)	Non-RCT	USA	Nursing students received a telehelath simulation-based learning experience focused heavily on communication skills and building rapport. In groups, participants interacted with a SP using a virtual platform and then debriefed the experience.<br><br>n=95<br><br>The mean score for the response prompt, "I am more confident in communicating with my patient," was 2.95/3.00	There was no control group (post-test only design).	Based on a survey filled out post training, the learning experience appears to have improved students' confidence communicating with patients when using telehealth.	CRITICAL

2267 (Mahabamunuge et al., 2021)	Non-RCT	USA	A telehealth education curriculum designed for medical students consisting of: 1) a supervised telehealth patient encounter via video conference with an attending physician supervising, and 2) a virtual OSCE to mimic a telehealth patient encounter followed by debriefing sessions focused on communication and clinical reasoning.<br><br>n=48<br><br>"This session increased my confidence in navigating a patient interview using a virtual platform"<br><br>Strong agree: 23 (47.9%)<br>Moderately agree: 20 (41.7%)<br>Moderately disagree: 1(2.1%)<br>Strongly disagree: 4(8.3%)	There was no control group (post-test only design).	90% of students strongly or moderately agreed that the virtual OSCE increased their confidence navigating a patient interview when using a virtual platform.	CRITICAL
2490 (Newcomb et al., 2020)	Non-RCT	USA	A 2-hour virtual class created to increase medical students' skills communicating during video consults. The class included an overview of best practices and two 15-minute role play sessions with a SP which included group debriefing.<br><br>Mean confidence level and range of results:<br><br>**Pre-intervention (N=5)**<br>Exploring patient's perception: 4 (3.5-4.5)<br>Sharing information: 4.25 (3.75-4.6)<br>Checking understanding: 4 (3.5-4.5)<br>Exploring concerns/empathy: 4 (3.5-4.5)<br>Clearly establishing a plan: 4.5 (4-4.75)<br><br>**Post-intervention (N=5)**<br>Exploring patient's perception: 4.75 (4.6-5)<br>Sharing information:5<br>Checking understanding: 5<br>Exploring concerns/empathy: 4.75 (4.6-5)<br>Clearly establishing a plan: 4.75 (4.6-5)	There was no control group, and results were compared pre and post intervention.	Based on graph results, all domains of student confidence increased from pre to post intervention.[w]	CRITICAL
2816 (Lanier et al., 2017)	Non-RCT	Switzerland	Residents participated in a training course focused on communication skills and patient-centred EHR use. Training included 2 large group sessions and 2–4 individualized 1-hour coaching sessions based on videotaped clinical encounters. Sessions were supervised by a communication skills teacher.<br><br>Mean (SD)<br><br>**Pre-intervention (N=17)**<br>"I feel comfortable using the EHR during the encounter": 3.00 (1.23)	There was no control group, and results were compared pre and post intervention.	Residents reported feeling slightly more comfortable using the EHR in the consultation after receiving training (mean increase of 0.76 from pre to post intervention).	CRITICAL

			**Post-intervention (N=17)**<br>"I feel comfortable using the EHR during the encounter": 3.76 (1.20)			
1963 (Lee et al., 2017)	Non-RCT	USA	A 'patient-centred EHR use' curriculum designed for 2nd year medical students, consisting of a lecture and group OSCE. During the lecture, students watched a video, engaged in reflective exercises, and learned best practices for communication. During the OSCE, 1 of 4 students interacted with a SP while using the EHR.<br><br>N= 89 (80 completed survey)<br><br>How confident are you using the EHR in a patient-centered manner?<br>21/80 (26%) said moderately confident	3rd year students received no formal training and served as a historical control group by completing the same OSCE individually.<br><br>N=96 (88 completed survey)<br><br>How confident are you using the EHR in a patient-centered manner?<br>14/88 (16%) said moderately confident	More students were confidence using the EHR with formal training (compared to no formal training).	CRITICAL
**Outcome: Nurse-person therapeutic relationship**						
Bosse (2012);<br>Evans (1989);<br>Gartmeir (2015);<br>Liu (2016);<br>Lupi (2012);<br>Hobgood (2009);<br>Legg (2005);<br>Shaddheau (2015);<br>Windish (2005)<br><br>*From review 265 (Gilligan et al., 2021)	Systematic review and meta-analysis of 8 RCTs and 1 cluster RCT	Multiple: Germany, Australia, USA, South Africa	Interventions that aim to improve medical students' interpersonal communication when undertaking medical consultations, including interventions targeting the communication tasks and skills associated with relationship building, information gathering, and planning and explaining, as well as specific tasks of communication such as listening, using appropriate non-verbal communication, and providing closure.<br><br>n=405<br><br>**Subgroups:**<br>Assessed by experts (faculty or trained assessors): n=5 studies, 225 participants<br><br>Assessed by standardized patients: n=4 studies, 180 participants	No intervention, or usual training.<br><br>n=429<br><br>**Subgroups:**<br>Assessed by experts (faculty or trained assessors): n=5 studies, 231 participants<br><br>Assessed by standardized patients: n=4 studies, 198 participants	Communication skills interventions may have very little positive effect, or no effect, on relationship building scores when compared to the usual curriculum or control<br>SMD 0.18 (95% CI -0.15, 0.51)<br><br>**Subgroups**:<br>Assessed by experts SMD 0.03 (95% CI -0.19, 0.26)<br><br>Assessed by standardized patients: SMD 0.39 (95% CI –0.33, 1.12)	Systematic review: LOW<br><br>Individual studies: NOT SERIOUS

Acronyms
CI = confidence interval
HER = Electronic Health Record
OSCE = Observed structured clinical exam
RCT = randomized controlled trial
SMD = standardized mean difference
SD = Standard deviation
SP = Standardized patient

Tools used to measure outcomes

Study 265: Different scales were used by different studies included in the review to measure communication skills, information gathering about patient perspectives/concerns, and relationship building/rapport: total scores varied across communication items or average score on a Likert scale.
Study 1530: Students rated confidence on a 5-point scale (1= not at all confident, 5 = extremely confident)
Study 1963: The SP evaluated OSCE performance using a 5-point Likert scale, the total score could range from 15-80 with higher scores indicating better performance
Confidence was measured on a 5-point scale, ≥4 equaled moderate confidence
Study 2005: Confidence was measured on a 3-point Likert scale using the simulation effectiveness tool-modified (SET-M) (1 = do not agree, 3 = strongly agree)
Study 2267: Confidence was measured on a 4-point Likert scale (1=strongly agree, 4=strongly disagree)
Study 2490: Confidence was measured using a 5-point Likert scale (1= no confidence, 5 = completely confident)
Study 2816: Outcomes were measured on a 5-point Likert Scale (1= completely disagree, 1= completely agree)

Explanations

[a] Five RCTs were included from a systematic review and meta-analysis (Gilligan, 2021).

[b] The review was assessed using the ROBIS tool for systematic reviews, and had a low risk of bias. Studies included in the review were assessed by the authors in accordance with the Cochrane Handbook for Systematic reviews of Interventions; all studies that could be pooled were rated by experts, and none were deemed as having high risk of bias overall. We did not downgrade.

[c] All studies demonstrated a positive direction of effect, however there was high heterogeneity across the studies (I^2=78%). We downgraded by 0.5.

[d] The outcome of 'information gathering about patient perspectives/concerns', including satisfaction, was slightly different from the original outcome of interest (person/caregiver/family experience or satisfaction). We downgraded by 1.

[e] The total number of participants was less than the optimal 800 participants (n=405). We downgraded by 1.

[f] 12 RCTs, 2 cluster RCTs and 4 quasi-cluster RCTs were included from a systematic review and meta-analysis (Gilligan et al., 2021).

[g] The review was assessed using the ROBIS tool for systematic reviews, and had a low risk of bias. Studies included in the review were assessed by the authors in accordance with the Cochrane Handbook for Systematic reviews of Interventions; review authors downgraded by one due to high or unclear risk of bias in several domains across the studies; there were concerns noted around randomization, allocation concealment, blinding of participants and personnel, contamination, and outcome measurement.

[h] Review authors downgraded by 1 due to substantial heterogeneity (I^2=90%) and wide variation in effect estimates and some CIs with no overlap, not explained by subgroup analysis.

[i] The outcome of interest was slightly different from the original outcome of interest (nurse competence vs. communication skills). We downgraded by 0.5.

[j] The total number of participants was more than the optimal 800 participants (n=1356). We did not downgrade.

[k] Studies were assessed using the ROBINS-I tool for non-RCT studies, and there was critical risk of bias related to confounding variables, deviations from the intended interventions, missing data, measurement of outcomes and selection of the reported results. We downgraded by 2.

[l] There was variation in the tools used to measure the outcome. We downgraded by 0.5.

[m] Participants were nursing students in only one study. In the other studies, participants were physicians or medical students. We did not downgrade, due to the very similar nature of the populations.

[n] The total number of participants was less than the optimal 800 participants (n=209). We downgraded by 2.

[o] Given the heterogeneity of the outcomes and outcome measurement tools, a pooled statistical analysis of the results was not possible.

[p] Study was assessed using the ROBINS-I tool for non-RCT studies, and there was critical risk of bias related to confounding variables, missing data, and measurement of outcomes. We downgraded by 2.

[q] Participants were medical students. We did not downgrade, due to the very similar nature of the populations

[r] The total number of participants was less than the optimal 800 participants (n=168). We downgraded by 2.

[s] 8 RCTs and 1 cluster RCT were included from a systematic review and meta-analysis (Gilligan et al., 2021).

[t] The review was assessed using the ROBIS tool for systematic reviews, and had a low risk of bias. Studies included in the review were assessed by the authors in accordance with the Cochrane Handbook for Systematic reviews of Interventions; all studies that could be pooled were rated by experts, and none were deemed as having high risk of bias overall. We did not downgrade.

[u] Review authors downgraded by 1 due to substantial heterogeneity (I^2=81%).

[v] The total number of participants was more than the optimal 800 participants (n=834). We did not downgrade.

[w] Study did not provide numerical results (only a chart is provided). These numbers have been interpreted by RNAO's BPG team.

References

1. Gilligan, C., Powell, M., Lynagh, M. C., Ward, B. M., Lonsdale, C., Harvey, P., James, E. L., Rich, D., Dewi, S. P., Nepal, S., Croft, H. A., & Silverman, J. (2021). Interventions for improving medical students' interpersonal communication in medical consultations. *The Cochrane database of systematic reviews*, *2*(2), CD012418. https://doi.org/10.1002/14651858.CD012418.pub2
2. Gunner, C. K., Eisner, E., Watson, A. J., & Duncan, J. L. (2021). Teaching webside manner: development and initial evaluation of a video consultation skills training module for undergraduate medical students. *Medical education online*, *26*(1), 1954492. https://doi.org/10.1080/10872981.2021.1954492
3. Lanier, C., Dominicé Dao, M., Hudelson, P., Cerutti, B., & Junod Perron, N. (2017). Learning to use electronic health records: can we stay patient-centered? A pre-post intervention study with family medicine residents. *BMC family practice*, *18*(1), 69. https://doi.org/10.1186/s12875-017-0640-2
4. Lee, W. W., Alkureishi, M. L., Wroblewski, K. E., Farnan, J. M., & Arora, V. M. (2017). Incorporating the human touch: piloting a curriculum for patient-centered electronic health record use. *Medical education online*, *22*(1), 1396171. https://doi.org/10.1080/10872981.2017.1396171
5. Mahabamunuge, J., Farmer, L., Pessolano, J., & Lakhi, N. (2021). Implementation and Assessment of a Novel Telehealth Education Curriculum for Undergraduate Medical Students. *Journal of advances in medical education & professionalism*, *9*(3), 127–135. https://doi.org/10.30476/jamp.2021.89447.1375
6. Newcomb, A. B., Duval, M., Bachman, S. L., Mohess, D., Dort, J., & Kapadia, M. R. (2021). Building Rapport and Earning the Surgical Patient's Trust in the Era of Social Distancing: Teaching Patient-Centered Communication During Video Conference Encounters to Medical Students. *Journal of surgical education*, *78*(1), 336–341. https://doi.org/10.1016/j.jsurg.2020.06.018
7. Riley E, McCance C, Ward N, deGravelles P. (2022). Evaluation of a simulation-based learning experience using a prenatal telehealth scenario with prelicensure nursing students. *Teach Learn Nurs.* 17(2):220–4.
