# Supplementary material for: Developing a Best Practice Guideline for Clinical Practice in a Digital Health Environment: Systematic Reviews Based on the Grading of Recommendations, Assessment, Development, and Evaluation Approach
Source: JMIR Nurs. 2026 Jan 23;9:e74942. doi: 10.2196/74942 (PMC12829893; doi:10.2196/74942)
Supplement: Multimedia Appendix 6 [file nursing-v9-e74942-s006.pdf]

RNAO Registered Nurses' Association of Ontario L'Association des infirmières et infirmiers autorisés de l'Ontario
Speaking out for nursing. Speaking out for health.

Multimedia Appendix: Recommendation 3.0 Evidence Profile

Recommendation question: Should the implementation of interdisciplinary peer champion models in health-service organizations be recommended or not to facilitate education for health providers on the use of digital health technologies?

Recommendation 3.0: The expert panel suggests that health service organizations implement interdisciplinary peer champion models to facilitate education for nurses and other health providers on the use of digital health technologies.

Population: Health providers at all levels of an organization, and persons receiving care
Intervention: Interdisciplinary peer champion model (in general, or specific to digital health technologies)
Comparison: No interdisciplinary peer champion model
Outcomes: Health provider competence [with using technology] (critical), health provider adoption of technology (critical), health provider confidence [with using technology] (critical), health provider sensitive outcomes (falls, pressure injuries, pain) (critical), sustainability of education (i.e., knowledge and skills retention) (critical, not measured)

Setting: All practice settings where nurses provide care to persons using digital health technologies (e.g., primary care, community care, acute care, long-term care, etc.)

Bibliography: 984, Kadish et al. (2018), 1073

Quality assessment							No. of participants		Reported effects/outcomes	Certainty	Reference
№ of studies	Study design	Risk of bias	Inconsistency	Indirectness	Imprecision	Publication Bias	Intervention	Control			
**Health provider competence [with using technology]** *(Measured using a self-report survey)*											
1	Non-randomized single arm study	Very serious[a]	Not serious	Not serious	Very serious[b]	Undetected	Physician champions provided educational outreach<br><br>n=1509 received the intervention<br><br>**Pre and Post Intervention**<br>n=88 (who completed both pre and post surveys)<br><br>Familiar with EHR messaging tool:<br>Pre survey: 90%<br>Post survey: 97%<br><br>Know how to send messages with EHR messaging tool:<br>Pre survey: 79%<br>Post survey: 96%	No true control group.	There was a 17% increase in clinicians who knew how to send messages using the EHR tool after the physician champions provided education.	⊕◯◯◯<br>Very Low	984: Walsh et al., 2018
**Health provider adoption of technology** (*Measured using a self-report survey and EHR data*)											
1	Non-randomized single arm	Very serious[a]	Not serious	Not serious[c]	Very serious[b]	Undetected	Physician champions provided educational outreach	No true control group.	The proportion of providers who identified that the EHR communication tool was their preferred mode of communication for semi-urgent patient issues	⊕◯◯◯<br>Very Low	984: Walsh et al., 2018

Quality assessment							No. of participants		Reported effects/outcomes	Certainty	Reference
№ of studies	Study design	Risk of bias	Inconsistency	Indirectness	Imprecision	Publication Bias	Intervention	Control			
	study						n=1509 participants received the intervention<br><br>n=88 (who completed both pre and post surveys)<br><br>EHR messaging tool as the preferred mode of communication for semi-urgent patient issues:<br>Presurvey: 66%<br>Postsurvey: 88%<br><br>**During preintervention period:** Total number of monthly messages increased from 2300 to 4000 per month<br><br>**During intervention period:** Total number of messages used in the EHR messaging tool increased from 4000 to 8400 per month		increased by 22% after receiving the intervention. There was also increased self-reported and observed use of the EHR messaging tool after the intervention was received. The rate of EHR messaging tool messages increased from 167 to 271 per month.		
**Health provider confidence [with using technology]** *(Measured using surveys)*											
1	Non-randomized, single arm study	Very Serious[a]	Not serious	Not serious	Very serious[d]	Undetected	Individually tailored EMR training conducted one-on-one with a credentialed trainer<br><br>n=185 (133 physicians, 42 NPs, 10 physician assistants)<br><br>**Pre-test (n=87)**<br>Providers who strongly agree or agree they feel confident using the EMR (%):<br><br>Overall: 58%<br>Clinical Review: 78%	No true control group.	Providers that completed both surveys reported an increase in confidence across all activities after training. There was a 36% increase in overall confidence (baseline 58% to post-training 94%).	⊕◯◯◯ Very Low	Kadish et al., 2018

Quality assessment							No. of participants		Reported effects/outcomes	Certainty	Reference
№ of studies	Study design	Risk of bias	Inconsistency	Indirectness	Imprecision	Publication Bias	Intervention	Control			
							Documentation: 61%<br>Placing orders: 65%<br>Inbox management: 61%<br><br>**Post-test (n=92)**<br>Overall: 94%<br>Clinical Review: 93%<br>Documentation: 92%<br>Placing orders: 90%<br>Inbox management: 87%				
**Health provider sensitive outcomes (falls, pressure injuries, pain)** (*Measured as resident clinical outcomes: clinical physical function, pressure ulcer prevalence, malnourishment, prevalence of delirium, infections, and comfort in the last week of dying)*											
6[e]	RCTs	Very Serious[f]	Serious[g]	Not Serious	Not Serious[h]	Undetected	Stand-alone or multi-component intervention that used a champion<br>n=7788 residents<br><br>pressure ulcer events = 401<br><br>infections=1150<br><br>delirium events=23<br><br>*(total events across intervention and control groups)*	Studies compared intervention to baseline data (i.e., no intervention).	It is uncertain whether champions, as part of a multi-component intervention may improve health provider sensitive outcomes; there was either no difference (malnutrition, comfort in the last week of life, delirium, infection rate, category II-IV pressure ulcer prevalence) or a slight improvement in the clinical outcomes (physical function, category I-IV pressure ulcer prevalence) for those in the LTC facilities with the champion intervention.<br><br>Clinical Physical Function (unadjusted MD = 4.77 [95% CI: 1.39, 8.15])<br><br>Pressure ulcer prevalence (unadjusted RD = 0.00 [95% CI: − 0.03, 0.02])<br><br>Moderate-severe malnourishment (adjusted OR = 1.6 [95% CI: 0.8, 3.1])<br><br>Prevalence of delirium (unadjusted RD = − 0.03 [95% CI: − 0.10, 0.04])<br><br>Infections (adjusted hazard ratio = 0.99 [95% CI: 0.87,<br>1.12])	⊕◯◯◯<br>Very Low	1073: Hall et al. (2021)

Quality assessment							No. of participants		Reported effects/outcomes	Certainty	Reference
№ of studies	Study design	Risk of bias	Inconsistency	Indirectness	Imprecision	Publication Bias	Intervention	Control			
									Comfort in the last week of dying (adjusted MD = 0.91 [95% CI: − 1.03, 2.85])		
**Sustainability of education (i.e., knowledge and skills retention)** (not measured*)*											
N/A											

Additional Table – Individual Study Details

Reference	Study Design	Country	Intervention Group Details	Control Group Details	Reported Effects/Outcomes	Risk of Bias
**Outcome: Health provider competence [with using technology]**						
Walsh et al. (2018)	Non-randomized single arm study	USA	Physician champions provided educational outreach to 16 academic departments, using 10-minute case-based presentations, customized for each specific clinical division audience. The physician educational outreach intervention aimed to increase knowledge and promote the use of the standard EHR tool for communication between providers regarding patient care.<br><br>n=1509 received the intervention<br><br>n=88 (who completed both pre and post surveys)	There was no true control group and results were compared pre and post intervention.	There was a 17% increase in clinicians who knew how to send messages using the EHR tool after the physician champions provided education.	CRITICAL
**Outcome: Health provider adoption of technology**						
Walsh et al. (2018)	Non-randomized single arm study	USA	Physician champions provided educational outreach to 16 academic departments, using 10-minute case-based presentations, customized for each specific clinical division audience. The physician educational outreach intervention aimed to increase knowledge and promote the use of the standard EHR tool for communication between providers regarding patient care.<br><br>n=1509 participants received the intervention	There was no true control group and results were compared pre and post intervention.	The proportion of providers who identified that the EHR communication tool was their preferred mode of communication for semi-urgent patient issues increased by 22% after receiving the intervention. There was also increased self-reported and observed use of the EHR messaging tool after the intervention was received. The rate of EHR messaging tool messages increased from 167 to 271 per month.	CRITICAL

			n=88 (who completed both pre and post surveys)			
**Outcome: Health provider confidence [with using technology]**						
Kadish et al. (2018)	Non-randomized, single arm study	USA	Physicians, nurse practitioners (NPs), and physician assistants received two hours of individually tailored EMR training conducted one-on-one with a credentialed trainer. Providers were already familiar with the EMR and content was tailored to reflect their workflows and personal challenges in EMR use.<br><br>n=185 (133 physicians, 42 NPs, 10 physician assistants)<br><br>** Results for physicians, NPs and physician assistants were grouped together.*	There was no control group, and results were compared pre and post intervention.	Providers that completed both surveys reported an increase in confidence across all activities after training. There was a 36% increase in overall confidence (baseline 58% to post-training 94%).	CRITICAL
**Outcome: Health provider sensitive outcomes (falls, pressure injuries, pain)**						
Beekman (2013); Chami (2012); Gaskill (2009); Resnick (2011); Siddiqi (2016); Van den Block (2020)<br><br>*From review 1073 (Hall et al. (2021)	Systematic review of RCTs	Multiple: Belgium, France, Australia, UK, USA and one multi-country study.	Stand-alone or multi-component intervention that used a champion (i.e., an internal nursing staff member who had an implementation-related role, had received supplementary training, assumed responsibility for a specific topic area and may have acted as a key contact person with external health providers) to improve staff adherence to guidelines and resident outcomes.<br>n=7788 residents<br><br>pressure ulcer events = 401<br><br>infections=1150<br><br>delirium events=23	No intervention group (no implementation strategies tested), or another intervention (which may or may not have included a champion).	It is uncertain whether champions, as part of a multi-component intervention may improve health provider sensitive outcomes; there was either no difference (malnutrition, comfort in the last week of life, delirium, infection rate, category II-IV pressure ulcer prevalence) or a slight improvement in the clinical outcomes (physical function, category I-IV pressure ulcer prevalence) for those in the LTC facilities with the champion intervention.<br><br>Clinical Physical Function (unadjusted MD = 4.77 [95% CI: 1.39, 8.15])<br><br>Pressure ulcer prevalence (unadjusted RD = 0.00 [95% CI: − 0.03, 0.02])<br><br>Moderate-severe malnourishment (adjusted OR = 1.6 [95% CI: 0.8, 3.1])<br><br>Prevalence of delirium (unadjusted RD = − 0.03 [95% CI: − 0.10, 0.04])<br><br>Infections (adjusted hazard ratio = 0.99 [95% CI: 0.87,<br>1.12])<br><br>Comfort in the last week of dying (adjusted MD = 0.91 [95% CI: − 1.03, 2.85])	Systematic review: LOW<br><br>Individual studies: VERY SERIOUS

Acronyms

EHR = electronic health record

EMR = electronic medical record

SD = standard deviation

SR = systematic review

RCT = randomized controlled trial

RD = risk difference

MD = mean difference

CI = confidence interval

LTC = long-term care

NP = nurse practitioner

Tools used to measure outcomes

Study 984: Two online provider surveys assessed provider-to-provider communication using the EHR tool at baseline and one-year follow-up.

Kadish et al. (2018): The first survey was sent before training and used a 5-point Likert scale to measure confidence in the EMR overall and in five key activities. Immediately after training, a second survey was sent to participants to evaluate the session and to gauge confidence in the same activities. The second survey included similar questions as the first as well as an additional request to provide feedback on the training.

Study 1073: Cochrane Effective Practice and Organization of Care (EPOC)-recommended outcomes such as patient outcomes (resident health outcomes: clinical physical function, pressure ulcer prevalence, malnourishment, prevalence of delirium, infections, and comfort in the last week of dying).

Explanations

---

[a] Study was assessed using the ROBINS-I tool for non-RCT studies, and there was a critical risk of bias related to confounding variables, missing data, measurement of the outcomes, and selection of the reported results. We downgraded by 2.
[b] The total number of participants was far less than the optimal 800 (n=88). We downgraded by 2.
[c] Health provider adoption of technology was measured indirectly using provider preferred mode of communication and the total number of messages used in the EHR messaging tool per month. We downgraded by 0.5.
[d] The total number of participants was far less than the optimal 800 participants (n=185). We downgraded by 2.
[e] Six RCTs were included from a systematic review (Hall et al. 2021)
[f] The review was assessed using the ROBIS tool for systematic reviews, and had a low risk of bias. Studies included in the review were assessed by the authors using the Cochrane ROB tool for RCTs; 5 studies had a high ROB, 1 study had an unclear ROB. We downgraded by 2.
[g] There was variability in the direction of effect shown in the studies; most studies demonstrated a positive direction of effect, but some demonstrated no effect. The authors also noted heterogeneity across the studies. We downgraded by 1.
[h] The total number of participants greater than the optimal 800 participants (n=7788). The total number of events was also greater than the optimal 300 events (n=813). We did not downgrade.

References

1. Hall, A.M., Flodgren, G.M., Richmond, H.L. et al. (2021). Champions for improved adherence to guidelines in long-term care homes: a systematic review. *Implement Sci Commun* 2(85). https://doi.org/10.1186/s43058-021-00185-y
2. Kadish, S. S., Mayer, E. L., Jackman, D. M., Pomerantz, M., Brady, L., Dimitriadis, A., Cleveland, J. L. F., & Wagner, A. J. (2018). Implementation to Optimization: A Tailored, Data-Driven Approach to Improve Provider Efficiency and Confidence in Use of the Electronic Medical Record. *Journal of oncology practice*, *14*(7), e421–e428. https://doi.org/10.1200/JOP.18.00093
3. Walsh, K. E., Secor, J. L., Matsumura, J. S., Schwarze, M. L., Potter, B. E., Newcomer, P., Kim, M. K., & Bartels, C. M. (2018). Secure Provider-to-Provider Communication With Electronic Health Record Messaging: An Educational Outreach Study. *Journal for healthcare quality : official publication of the National Association for Healthcare Quality*, *40*(5), 283–291. https://doi.org/10.1097/JHQ.0000000000000115
