# Supplementary material for: Developing a Best Practice Guideline for Clinical Practice in a Digital Health Environment: Systematic Reviews Based on the Grading of Recommendations, Assessment, Development, and Evaluation Approach
Source: JMIR Nurs. 2026 Jan 23;9:e74942. doi: 10.2196/74942 (PMC12829893; doi:10.2196/74942)
Supplement: Multimedia Appendix 7 [file nursing-v9-e74942-s007.pdf]

Multimedia Appendix: Recommendation 4.0 Evidence Profile (Quantitative)

Recommendation question: Should the use of artificial intelligence-driven predictive analytics software or systems (e.g., command centers and risk assessment software tools) for nurses providing care in all practice settings be recommended or not to inform clinical decision-making and improve clinical outcomes?

Recommendation 4.0: The expert panel suggests that health service organizations implement clinical decision support systems (CDSS) or early warning systems that use artificial intelligence-driven predictive analytics to support nurses' and health providers' clinical decision-making.

Population: All nurses and other health providers, and persons receiving care
Intervention: Use of AI-driven predictive analytics
Comparison: No use of AI-driven predictive analytics
Outcomes: Proactive/ anticipatory care (critical), critical incidents (critical, not measured), failure to rescue (critical), consistent application of evidence-based practice (critical), nurse sensitive outcomes (i.e., falls, pressure injuries, pain) (critical)

Setting: All practice settings where nurses provide care to persons using digital health technologies (e.g., primary care, community care, acute care, and long-term care)
Bibliography: 597, 906, 2030, 226

Quality assessment							No. of participants		Reported effects/outcomes	Certainty	Reference
№ of studies	Study design	Risk of bias	Inconsistency	Indirectness	Imprecision	Publication Bias	Intervention	Control			
**Proactive/anticipatory care** *(Measured using data from EHR or clinical records)*[a]											
13[b]	RCTs and retrospective cohort studies	Very Serious[c]	Serious[d]	Not serious	Not Serious[e]	Undetected	AI-based CDSS<br><br>n=706 (across intervention and control groups)<br><br>Assessment time: 2.778±0.858 minutes<br><br>Automatic detection of patients: 100%<br><br>Time in therapeutic range: 81.6	Non-AI based approaches (i.e., standard care, manual nurse decision, and no AI algorithm-based support.)<br><br>Assessment time: 15 minutes<br><br>Automatic detection of patients: no raw data<br><br>Time in therapeutic range: 80.9	Both systematic reviews demonstrated overall that AI and ML-based prediction tools improved proactive care compared to non-AI or manual approaches.<br><br>Two of the three RCTs in the systematic review demonstrated that the AI-based CDSS improved proactive/anticipatory care, while one RCT demonstrated little to no difference[f].	⊕⊕◯◯<br>Low	597: Cresswell et al. (2020)
							Machine learning-based diagnostic and prognostic prediction models to predict	Manual diagnosis with and without clinical scoring tools.	Ten studies in the review demonstrated that the machine learning models outperformed the manual diagnosis and clinical scoring tools at all prediction times.		226: Frondelius et al. (2023)

Quality assessment							No. of participants		Reported effects/outcomes	Certainty	Reference
№ of studies	Study design	Risk of bias	Inconsistency	Indirectness	Imprecision	Publication Bias	Intervention	Control			
							ventilator associated pneumonia (VAP).<br><br>n=2383 (VAP events across all groups)	n=not specified			
**Failure to Rescue** *(Measured using data from EHR)*[g]											
1	Non-randomized, single arm study	Serious[h]	Not serious	Serious[i]	Very serious[j]	Undetected	Sepsis improvement program using real-time data-driven CDSS<br><br>**Before implementation:**<br>n=566<br><br>Deaths from sepsis: 51<br><br>Sepsis related mortality: 90 deaths per 1000 cases of sepsis<br><br>**After implementation:**<br>n=212<br><br>Deaths from sepsis: 9<br><br>Sepsis related mortality: 42 deaths per 1000 cases of sepsis	There was no control group, and results were compared pre and post intervention.	The primary outcome, sepsis mortality, decreased by 53% (95% CI, 1.06-5.25) after the intervention was implemented. Patients screened using the sepsis CDS system had a 2.1 times lower risk of death (OR: 0.474; 95% CI, 0.228-0.988), compared to patients in the pre-implementation period group.	⊕◯◯◯<br>Very low	2030:<br>Manaktala & Claypool (2017)
**Consistent application of evidence-based practice**[k] *(measured as guideline adherence using CDSS data)*											
5[l]	Non-randomized studies	Very Serious[m]	Not serious	Not serious	Not serious	Undetected	Use of CDSS by physicians<br><br>n=735<br>Mean percentage of guidelines-adherent treatment decisions (across 4 studies): 80.47%	No CDSS or standard care<br><br>n=804<br>Mean percentage of guidelines-adherent treatment decisions (across 4 studies): 69.02%	Four studies reported an increase in adherence to cancer guidelines after implementation of a CDSS ranging from 3.2% to 23.61%. One study showed a 60% reduction in number of deviations from pain management guidelines.	⊕⊕◯◯<br>Low	906:<br>Klarenbeek et al., 2020

Quality assessment							No. of participants		Reported effects/outcomes	Certainty	Reference
№ of studies	Study design	Risk of bias	Inconsistency	Indirectness	Imprecision	Publication Bias	Intervention	Control			
							Percentage of deviations from guideline (1 study, n=50):<br>On hospital admission: 85%<br>At discharge: 14%	Percentage of deviations from guideline (1 study, n=50):<br>On hospital admission: 80%<br>At discharge: 74%			
**Nurse sensitive outcomes (falls, pressure injuries, pain)** *(Measured using data from EHR)*											
2[n]	Non-randomized studies	Very Serious[o]	Not serious	Not serious	Very serious[p]	Undetected	Use of CDSS by physicians<br><br>n=82<br>Mean pain score (NVAS) at hospital admission: 7.4<br><br>Mean pain score (NVAS) over the first 28h: 4.2<br><br>Pain intensity score (NVAS) on day 5 after admission<br><br>At rest: 2<br><br>During physical activity: 4	No CDSS or standard care<br><br>n=80<br>Mean pain score (NVAS) at hospital admission: 6.3<br><br>Mean pain score (NVAS) over the first 28h: 4.9<br><br>Pain intensity score (NVAS) on day 5 after admission<br><br>At rest: 2.4<br><br>During physical activity: 4	There was little to no difference in mean pain scores between the intervention and control groups at any time points.	⨁◯◯◯<br>Very Low	906: Klarenbeek et al., 2020
**Critical incidents** *(Not measured)*											
N/A											

Additional Table – Individual Study Details

Reference	Study Design	Country	Intervention Group Details	Control Group Details	Reported Effects/Outcomes	Risk of Bias
**Outcome: Proactive/anticipatory care**						
Caballero-Ruiz et al. (2017); Finkelstein et al. (2013); Nielsen et al. (2017)<br><br>*From review 597 (Cresswell et al., 2020)	Systematic review of 3 RCTs	Spain, USA, Denmark	**Study 1 (Caballero-Ruiz et al., 2017):** n=450 pregnant women<br>Learning algorithm to manage the treatment of patients with gestational diabetes through telemedicine.<br><br>**Study 2 (Finkelstein et al., 2013):** n=65 lung transplant recipients<br>Computer based Bayesian triage algorithm for automated triaging.<br><br>**Study 3 (Nielsen et al., 2017):** n=191 patients with an indication for warfarin treatment<br>Personalized support for warfarin dosing based on the AI algorithm by health providers in home health settings.	**Study 1:** standard care.<br><br>**Study 2:** Manual nurse decision.<br><br>**Study 3:** No AI algorithm-based support (the dosage suggestion in the placebo arm would equal last week's dose of warfarin)	**Study 1:** Assessment time decreased by almost a third. Face-to-face time was reduced by 88% but overall time remained the same. Automatic detection of 100% of patients who needed insulin therapy and diet adjustment.<br><br>**Study 2:** No differences in outcomes measured.<br><br>**Study 3:** The intervention arm achieved a time in therapeutic (INR, international normalised ratio) range (TTR) of 81.6, while the placebo arm attained a TTR of 80.9 (difference (intervention arm minus placebo arm): 0.67 (95% confidence interval)).	Systematic review: LOW<br><br>Individual studies: VERY SERIOUS
Abujaber et al. (2021); Amador et al. (2022); Calvert et al. (2022); Dos Santos et al. (2021); Paucher et al. (2022); Giang et al. (2021); Liang et al. (2022); Liquet et al. (2012); Pearl et al. (2012); Schurink et al. (2007).<br><br>*From review 226 (Frondelius et al., 2023)	Systematic review and meta-analysis of 10 retrospective cohort studies	Qatar, Brazil, or unspecified	Population: Adults undergoing internal mechanical ventilation in ICU settings.<br><br>Machine learning-based diagnostic and prognostic prediction models using regression (e.g., logistic regression) or non-regression (e.g., random forests, neural networks, and support vector machines) modeling techniques to predict ventilator associated pneumonia (VAP).<br><br>The most common study aims were predicting VAP without its consequences. The timeline for VAP diagnosis and VAP variable extraction varied from the first hour after ICU admission to 24–48 h after initiation of ventilation, and beyond.<br><br>n=2382 VAP events (n of participants not specified)	Comparator: Manual diagnosis with and without clinical scoring tools.	Compared to clinical scoring tools, the ML models outperformed the PIRO (predisposition, insult, response, organ dysfunction) and CPIS (clinical pulmonary infection score) scoring tools at all prediction times.<br><br>The pooled AUROC for VAP and early VAP were 0.88 (95% CI 0.82–0.94, I2 98.4%) and 0.84 (95% CI 0.76–0.91, I2 98.7%), respectively.	Systematic review: LOW<br><br>Individual studies: VERY SERIOUS
**Outcome: Failure to Rescue**						

2030: Manaktala & Claypool (2017)	Non-randomized, single arm study	USA	The sepsis improvement program consisted of a combination of sepsis education, process improvement through change management, and an electronic CDSS. The CDSS conducted real-time surveillance of electronic medical record (EHR) data and delivered alerts to nursing staff's mobile devices at the point of care. The CDSS sent nursing staff four types of alerts: (1) informational prompts; (2) diagnostic alerts that informed nurses about new positive sepsis results or signs of worsening sepsis; (3) advice alerts; and (4) reminder alerts, which ensured that all alerts were acknowledged and that staff were complying with the recommended treatment plans.<br><br>n=212 patients with sepsis after exclusions	There was no control group, and results were compared pre and post intervention.<br><br>n=566 patients in the control period (pre intervention) after exclusions	The primary outcome, sepsis mortality, decreased by 53% (95% CI, 1.06-5.25) after the intervention was implemented.<br>Patients screened using the sepsis CDS system had a 2.1 times lower risk of death (OR: 0.474; 95% CI, 0.228-0.988), compared to patients in the pre-implementation period group.	CRITICAL
**Outcome: Consistent application of evidence-based practice**						
Christ et al. (2018); Rios et al. (2003); Seroussi et al. (2007); Bouaud et al. (2001); Bertsche et al. (2009)<br><br>*From review 906 (Klarenbeek et al., 2020)	Systematic Review of 5 non-randomized studies	Authors located in the Netherlands (no information on countries of included studies)	**Study 1 (Christ et al., 2018):** n=32<br>Decision support system for pain management of opioid-tolerant oncology patients.<br><br>**Study 2 (Rios et al., 2003):** n=270 (breast cancer patients), n=129 (prostate cancer patients)<br>Clinical practice guideline system for treatment planning of breast and prostate cancer.<br><br>**Study 3 (Seroussi et al., 2007):** n=177<br>Decision support system for treatment decisions for breast cancer.<br><br>**Study 4 (Bouaud et al., 2001):** n=127<br>Clinical practice guideline system for treatment decisions for breast cancer.<br><br>**Study 5 (Bertsche et al., 2009):** n=50<br>Decision support system for treatment of tumor-induced pain, for all types of cancer.	**Study 1:** n=30<br>National guidelines (no CDSS)<br><br>**Study 2:** n=320 (breast cancer patients), n=188 (prostate cancer patients)<br>Standard care.<br><br>**Study 3:** n=139<br>Standard care (multi-disciplinary team)<br><br>**Study 4:** n=127<br>Standard care (multi-disciplinary team)<br><br>**Study 5:** n=50<br>Standard care.	**Control vs. Intervention**<br>**Study 1:** Percentage of guidelines-adherent pain regimens: 40% vs. 46.9% (difference of 6.9%)<br><br>**Study 2:** Percentage of guideline adherent treatment decisions<br>Breast cancer: 77.8% vs. 87.1% (difference of 9.3%)<br>Prostate cancer: 86.7% vs. 89.9% (difference of 3.2%)<br><br>**Study 3:** Percentage of guideline adherent treatment decisions: 79.2% vs. 93.4% (difference of 14.2%)<br><br>**Study 4:** Percentage of guideline adherent treatment decisions: 61.42% vs. 85.03% (23.61%)<br><br>**Study 5:** Percentages of deviations from guidelines<br>On hospital admission: 80% vs. 85% (difference of -5%)<br>At discharge from hospital: 74% vs. 14% (difference of 60%)	Systematic review: LOW<br><br>Individual studies: VERY SERIOUS
**Outcome: Nurse sensitive outcomes (falls, pressure injuries, pain)**						
Christ et al. (2018); Bertsche et al. (2009)<br><br>*From review 906	Systematic Review of 2 non-randomized studies	Authors located in the Netherlands (no information on countries of included studies)	**Study 1 (Christ et al., 2018):** n=32<br>CDSS identified patients who require pain assessment, displays patient-specific information and the most recent and maximum pain score.<br><br>**Study 2 (Bertsche et al., 2009):** n=50<br>CDSS generated pain specific recommendations.	**Study 1:** n=30<br>No CDSS (use of national guidelines).<br><br>**Study 2:** n=50<br>No CDSS (standard care).	**Control vs. Intervention**<br>**Study 1:**<br>Mean pain score (NVAS) at hospital admission: 6.3 vs. 7.4<br>Mean pain score (NVAS) over the first 28h: 4.9 vs. 4.2<br><br>**Study 2:**	Systematic review: LOW<br><br>Individual studies: VERY SERIOUS

(Klarenbeek et al., 2020)					Pain intensity score (NVAS) on day 5 after admission<br>At rest: 2.4 vs. 2<br>During physical activity: 4 vs. 4	

Acronyms
AI = artificial intelligence
AUROC = area under the receiver operating characteristics curve
CDSS = clinical decision support system
CI= confidence interval
EHR = electronic health record
ICU = intensive care unit
NLP = natural language processing
OR= odds ratio
PI= pressure injuries
PU = pressure ulcers
SD= standard deviation
NVAS = numerical visual analog scale
VAP = ventilator associated pneumonia

Tools used to measure outcomes

Study 597: Assessment time, automatic detection time of patients who need insulin, triage time, time in therapeutic range of warfarin dosing.
Study 226: All included studies used the ICD-9 code for VAP (997.31) (i.e., the official system of assigning codes to diagnoses and procedures associated with hospital utilization in the United States).
Study 2030: Deaths from sepsis and sepsis related mortality rates (EHR data)
Study 906: Percentage of guideline adherence, mean NVAS at hospital admission, mean NVAS over the first 28 hours, pain intensity score (NVAS) on day 5 after admission.

Explanations

---

[a] Measured as assessment time, automatic detection time of patients who need insulin, triage time, time in therapeutic range, patients transferred to ICU after first elevated eCART score, mortality, or ventilator associated pneumonia.

[b] Three RCTs were included from a systematic review (Cresswell et al., 2020) and ten retrospective cohort studies were included from another systematic review (Frondelius et al., 2023).

[c] Both included reviews were assessed using the ROBIS tool for systematic reviews, and had a low risk of bias. Studies included in one review were assessed by the authors using the CASP checklist for RCTs; 2 studies had a low risk of bias, and one study had a high risk of bias; concerns were noted around lack of details describing the methods, and lack of blinding (Cresswell et al., 2020). Studies included in the second review were assessed by the authors using the PROBAST tool; all 10 studies had high or unclear risk of bias (Frondelius et al., 2023). We downgraded by 2.

[d] In one systematic review, proactive care was measured differently in each of the 3 included studies, with variation in the reported effects (Cresswell et al., 2020). We downgraded by 1.

[e] In one systematic review, the total number of participants was less than the optimal 800 participants (n=706) (Cresswell et al., 2020). In another systematic review, the total number of events was 2383 (Frondelius et al., 2023). We did not downgrade.

[f] The studies in the review lacked detail and raw data; a pooled statistical analysis of the results was not possible.

[g] Measured indirectly as death from sepsis.

[h] The study was assessed using the ROBINS-I tool for non-RCT studies, and had a critical risk of bias due to lack of control for confounding variables, deviations from the intended intervention, and selection of the reported results. We downgraded by 1.5.

[i] The one study measured the outcome of failure to rescue as 'sepsis related mortality'. We downgraded by 0.5.

[j] The total number of events was far less than the optimal 300 (n=60). We downgraded by 2.

[k] Measured as percentage of guidelines-adherent treatment decisions, or deviations from guidelines. Measured indirectly in one non-randomized study using degree of clinical performance scale and a 24-item decision making instrument.

[l] Five non-randomized studies were included from a systematic review (Klarenbeek et al., 2020).

[m] The review was assessed using the ROBIS tool for systematic reviews, and had a low risk of bias. Studies included in the review were assessed by the authors using the ROBINS-I tool for non-RCT studies; all 5 included studies had a critical risk of bias. Concerns were noted around confounding, selection of participants, missing data, measurement of outcomes, and selection in reported results. We downgraded by 2.

[n] Two non-randomized studies were included from a systematic review (Klarenbeek et al., 2020).

[o] The review was assessed using the ROBIS tool for systematic reviews, and had a low risk of bias. Studies included in the review were assessed by the authors using the ROBINS-I tool for non-RCT studies; 2 included studies had a critical risk of bias. Concerns were noted around confounding, selection of participants, missing data, measurement of outcomes, and selection in reported results. We downgraded by 2.

[p] The total number of participants was far less than the optimal 800 participants (n=162). We downgraded by 2.

References

1. Cresswell, K., Callaghan, M., Khan, S., Sheikh, Z., Mozaffar, H., & Sheikh, A. (2020). Investigating the use of data-driven artificial intelligence in computerised decision support systems for health and social care: A systematic review. *Health informatics journal*, *26*(3), 2138–2147. https://doi.org/10.1177/1460458219900452
2. Frondelius T, Atkova I, Miettunen J, et al. Early prediction of ventilator-associated pneumonia with machine learning models: A systematic review and meta-analysis of prediction model performance. Eur J Intern Med. 2023;S0953-6205(23)00406-5. Advance online publication. https://www.ejinme.com/article/S0953-6205(23)00406-5/fulltext
3. Klarenbeek, S. E., Weekenstroo, H. H. A., Sedelaar, J. P. M., Fütterer, J. J., Prokop, M., & Tummers, M. (2020). The Effect of Higher Level Computerized Clinical Decision Support Systems on Oncology Care: A Systematic Review. *Cancers*, *12*(4), 1032. https://doi.org/10.3390/cancers12041032
4. Manaktala, S., & Claypool, S. R. (2017). Evaluating the impact of a computerized surveillance algorithm and decision support system on sepsis mortality. *Journal of the American Medical Informatics Association: JAMIA*, *24*(1), 88–95. https://doi.org/10.1093/jamia/ocw056
